# NOD2 deficiency confers a pro‐tumorigenic macrophage phenotype to promote lung adenocarcinoma progression

**DOI:** 10.1111/jcmm.16790

**Published:** 2021-07-16

**Authors:** Yibei Wang, Ziwei Miao, Xiaoxue Qin, Bo Li, Yun Han

**Affiliations:** ^1^ Department of Thoracic Surgery Shengjing Hospital of China Medical University Shenyang China; ^2^ Department of Developmental Cell Biology Key Laboratory of Medical Cell Biology Ministry of Education China Medical University Shenyang China

**Keywords:** immunotherapy, lung adenocarcinoma, NOD2, tumour immune microenvironment, tumour‐associated macrophages

## Abstract

Nucleotide‐binding and oligomerization domain‐containing protein 2 (NOD2) was a member of the NOD‐like receptor family and played an important role in the innate immune response. Dysregulated NOD2 had been reported to contribute to tumorigenesis and progression. Here, we investigated that decreased NOD2 expressions could affect the phenotypic polarization of tumour‐associated macrophages and thus lead to the poor prognosis of lung adenocarcinoma patients. We clustered the patients by the single‐sample gene set enrichment analysis of tumour microenvironment and 13 prognostic differentially expressed immune‐related genes (PDEIRGs) were obtained based on prognostic analyses. After multiple assessments on the 13 PDEIRGs, NOD2 was considered to be the central immune gene and had a strong effect on suppressing tumour progression. Decreased NOD2 expression could be induced by cancer cells and lead to the phenotypic polarization of macrophages from protective M1 phenotype to pro‐tumorigenic M2 subtype which might be attributed to the down‐regulating of NF‐κB signalling pathway. This study draw attention to the role of inhibited innate immune function mediated by depletion of NOD2 in the TME. Our work also points to a potential strategy of NOD2‐mediated TAM‐targeted immunotherapy.

## 1 INTRODUCTION

Approximately 40% of patients with non‐small‐cell lung cancer have pathological subtype of lung adenocarcinoma (LUAD), which is also the type of lung cancer with higher driver mutations.^1^, ^2^ Although there have been many improvements in surgery, chemotherapy and radiotherapy, the mortality of LUAD has remained almost unchanged over the last few decades.^3^

At present, treatments of LUAD have been transformed from physicians’ empirical use of cytotoxic therapy to a hallmark of personalized medicine, moving from targeted therapy to immunotherapy.^4^ Immunotherapy targeting immune checkpoint blockers (ICBs), including cytotoxic T lymphocyte antigen 4 (CTLA‐4) and programmed cell death protein 1 (PD‐1) or programmed cell death 1 ligand (PD‐L1), has dramatically evolved the curative effect and changed the landscape of LUAD treatments.^5^ However, a significant number of LUAD patients those lack high PD‐L1 expression do not benefit from immunotherapy. Therefore, this amazing result is observed in only a minority of lung cancer patients, and many patients are only dependent on platinum‐based chemotherapy. Unfortunately, the efficacy of the basic chemotherapy is limited, with a median over survival (OS) of approximately one year. There is, therefore, an unmet need to better evaluate the immune status of patients with LUAD for exploring a more powerful clinical management for LUAD.

The characteristics of tumour immune microenvironment (TIME) depend mainly on interactions occurring between the different populations of intra‐tumour immune cells. Recent studies have shed new light on the complex interaction between tumour and host immune cells. The organization of immune cells within solid tumours, including various populations of lymphocytes, dendritic cells, natural killer cells, myeloid‐derived suppressor cells, neutrophils and macrophages, is a major determinant of patient outcome. Among them, macrophages infiltrated into TIME are called tumour‐associated macrophages (TAMs),^6^ which has been known as an accomplice to tumour by inhibiting immune response and promoting tumour growth.^6^, ^7^ Macrophages are one of the innate immunity compartments and have two functional phenotypes. M1 macrophages are induced through lipopolysaccharide (LPS) activation and express the high levels of pro‐inflammatory cytokines. M2 macrophages are induced by IL‐4 and express the different anti‐inflammatory cytokines. It is well known that tumour‐infiltrating macrophages of lung carcinoma are plastic. These cells can have pro‐ or anticancer functional phenotypes. M1 macrophages promote cancer cell elimination in association with the activation of adaptive immune cells, while M2 macrophages induce an immunosuppressive effect in particular through TGF‐β pathway. Therefore, the two phenotypes may have opposite effects on the prognoses of cancer patients.^8^ M1 macrophages function in immune surveillance, whereas M2 macrophages, in the TME, are closely related to bad clinical prognosis in many kinds of human cancers.^9^ Dissection of the roles of TAMs in tumour progression can pave the way to emerging TAM‐targeted therapeutic strategies.^10^

Here, we performed an immunological analysis of the TME (immunoscore) by clustering the immune components of LUAD samples in The Cancer Genome Atlas (TCGA) database and identified that NOD2 might be a suppressor for LUAD. NOD2 is a cytosolic receptor belonging to the NOD‐like receptor (NLR) family that initiates innate immune in response to bacterial peptidoglycan (PGN)‐conserved motifs. It has been reported that macrophages could exhibit a phenotypic polarization by up‐regulating NLR expressions to adapt to their surrounding microenvironment. Our results revealed down‐regulated NOD2 in macrophages induced by lung cancer cells could impel the phenotypic conversion of TAMs from the protective M1 phenotype to the pro‐tumorigenic M2 subtype. These findings provided a clue to develop an optional TAM‐targeted immunotherapeutic strategy for LUAD treatment.

## 2 MATERIALS AND METHODS

### 2.1 Databases and analysis software

Transcriptomic RNA‐seq data and clinical information were downloaded from TCGA (https://portal.gdc.cancer.gov/). Single‐cell RNA‐seq data were downloaded from Gene Expression Omnibus (GEO, GSE131907, https://www.ncbi.nlm.nih.gov/geo/query/acc.cgi?acc=GSE131907). A total of 1811 immune‐related genes (IRGs) were achieved from the ImmPort database (https://www.immport.org/home). The protein–protein interaction (PPI) networks of 13 PDEIRGs were achieved by STRING database (https://string‐db.org/). Gene Set Enrichment Analysis (GSEA) of NOD2 was done by GSEA software (version 4.0.3, https://www.gsea‐msigdb.org). All the data were analysed with R software (version 3.6.2, https://cran.r‐project.org/), R Bioconductor packages (http://www.bioconductor.org/), Perl software (version 5.28, https://www.perl.org/) and GraphPad Prism8 software (https://www.graphpad.com/).

### 2.2 Differentially expressed genes analysis

The differentially expressed genes (DEGs) were obtained from a comparison between HIC and LIC using the R package limma.^11^ The cut‐off values were false discovery rate (FDR) < 0.05 and |log2 fold change (FC)| > 1. Differentially expressed IRGs (DEIRGs) were obtained using the intersection between DEGs of HIC plus LIC and IRGs from ImmPort database.

### 2.3 Function and pathway enrichment analyses

The analyses of function and pathway enrichments of DEGs and DEIRGs were performed by R package cluster profiler^12^ with a strict cut‐off of FDR < 0.05. Gene Ontology (GO) terms and Kyoto Encyclopedia of Genes and Genomes (KEGG) pathways of NOD2 were obtained by GSEA,^13^ and the cut‐off value was FDR < 0.05 based on 10,000 permutations.

### 2.4 The risk score calculation

We cited the regression coefficients (coef) of the multivariate Cox hazard regression analysis and the FPKM (fragments per kilobase million) values of 13 PDEIRGs to create risk a score formula for each patient:

$$\text{Risk}\;\text{score}=2^{\wedge }\left[\Sigma_{i=1}^{n}\text{coef}\left(i\right)*\text{FPKM}\left(i\right)\right]$$

Here, coef (i) represented the regression coefficient of the ith selected PDEIRGs and FPKM (i) represented gene expression value of the ith selected PDEIRGs.

### 2.5 Cell lines and reagents

Human bronchial epithelial cells (HBEpiC), human lung cancer cell lines (A549 cells and NCI‐H1299 cells) and human myeloid leukaemia mononuclear cells (THP‐1) were obtained from American Type Culture Collection (ATCC). NCI‐H1299 and THP‐1 were cultured in RPMI 1640 supplemented with 10% FBS (Thermo Scientific, Waltham, Massachusetts, USA). HBEpiC and A549 were cultured in HycloneDME‐F12 supplemented with 10% FBS. All cells were cultured in 5% CO2 at 37°C. LPS and recombinant protein IL‐4 were from Beyotime Biotechnology (Nanjing, China).

### 2.6 Patients and specimens

All patients who attended Shengjing Hospital of China Medical University (CMU) from 2018 to 2019 were initially diagnosed with LUAD. All experimental protocols were approved by the Ethical Review Board of Shengjing Hospital of CMU and were performed in accordance with the committee guidelines. The written informed consents were obtained from all patients.

### 2.7 Immunohistochemistry

Immunohistochemistry was performed following the protocol provided by Rabbit ABC detection kit (ZSGB‐BIO, Beijing, China). The sections were incubated overnight at 4°C with rabbit anti‐human NOD2 polyclonal antibody (1:800, Proteintech, Wuhan, China) and rabbit anti‐human CD68 polyclonal antibody (1:800, Bioss, Beijing, China).

### 2.8 The cellular co‐culture system in vitro

10^5^ HBEpiC or LUAD cells were firstly seeded on the upper chamber of 6‐transwell polycarbonate membrane with pore sizes of 0.4 μm (Corning Costar Corp., Cambridge, MA). Meanwhile, 10^6^ THP‐1 cells were placed in another 6‐well plate. After co‐culturing overnight, the chamber cultured with HBEpiC or LUAD cells was inserted into the 6‐well plates with THP‐1 cells.

### 2.9 Cell treatment

For macrophage polarization, the THP‐1 cells were pretreated with SiRNA to knock down the level of NOD2; small‐interfering RNA (siRNA)1: 5′‐GCCCUGAUGACAUUUCUCUTT‐3′; siRNA2: 5′‐CCACAUGCAAGAAGUAUAUTT‐3′; siRNA3: 5′‐GCCUGAUGUUGGUCAAGAATT‐3′ were used as human NOD2 (NM_022162.1) target sequences. Non‐silencing siRNA (5′‐UUCUUCGAACGUGUCACGUTT‐3′) was used as the negative control. Then, the cells were co‐cultured with 100 ng/ml LPS or 10 ng/ml recombinant IL‐4 for 24 h, respectively.

### 2.10 Quantitative real‐time PCR (Q‐PCR)

TRIzol reagent (Invitrogen, Thermo Fisher Scientific Inc., Waltham, MA) was used to obtain total RNA according to the manufacturer's instructions. The cDNA was synthesized and amplified using a miRNA 1st Strand cDNA Synthesis Kit and miRNA Universal SYBR qPCR Master Mix kit (Vazyme, Nanjing, China) according to the manufacturer's protocols. The sequences of primers are listed in Table S1. The relative levels of target genes were normalized to GAPDH by 2^ΔΔCt^ method.

### 2.11 Western blot

Protein extraction and Western blot were performed as we previously reported.^14^ The primary antibodies were as follows: rabbit anti‐NOD2 (1: 1000, Proteintech, Wuhan, China) and rabbit anti‐β‐tubulin (1: 1000, Proteintech, Wuhan, China). The activation of the NF‐κB pathway was evaluated by NF‐κB pathway antibody kit (#9936, Cell Signaling Technology, USA). The secondary antibodies were anti‐rabbit IgG, HRP‐linked antibody (1:10,000, Wanleibio, Shenyang, China).

### 2.12 Statistical analyses

For the comparisons of two groups, unpaired Student *t*‐test was used for the variables of normal distribution, and Mann–Whitney *U* test was used to analyse the variables of non‐normal distribution. Non‐parametric test, Kruskal–Wallis test, parametric test and one‐way ANOVA were used for the comparisons of three or more groups.^15^ The Pearson correlation coefficient test was used to estimate the rank correlation among the different variables.^16^ The time‐dependent receiver operating characteristic (ROC) analysis was used to evaluate the accuracy of the prognostic model and calculate area under the curve (AUC).^17^, ^18^ *p* Values, two‐sided, of less than 0.05 were considered statistically significant.

## 3 RESULTS

### 3.1 Tumour immune microenvironments of LUAD patients from TCGA database were evaluated by ssGSEA and three immune clusters were established

**FIGURE 1:**
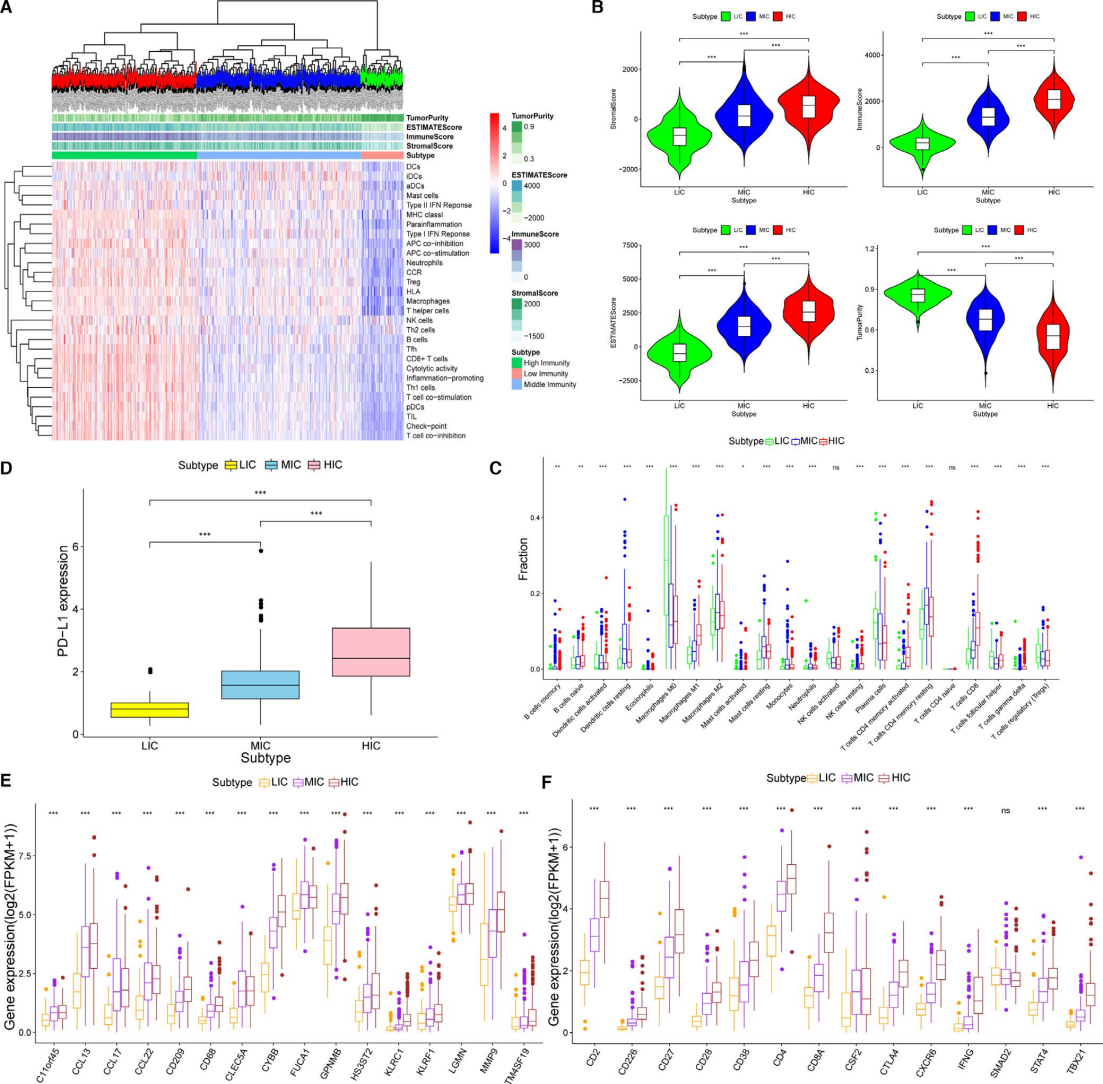
Tumour immune microenvironments of LUAD patients from TCGA database were evaluated by ssGSEA and three immune clusters were established. (A) Immune clustering of the entire included TCGA samples based on immunogenomic profiling of 29 immune signatures. (B) Comparisons of immune scores, stromal scores, ESTIAMTE scores and tumour purity scores of patients in the HIC, MIC and LIC (***p < 0.001). (C) The fraction of TME cells in three immunity clusters (*p < 0.05, **p < 0.01, ***p < 0.001, ns, no significance). (D) The level of PD‐L1 expression in HIC, MIC and LIC (***p < 0.001). (E) The level of marker gene expressions for innate immune in three immunity clusters. The thick line represents the median value. (*p < 0.05, **p < 0.01, ***p < 0.001, ns, no significance). (F) The level of marker gene expressions for specific immune in three immunity clusters. The thick line represents the median value. (*p < 0.05, **p < 0.01, ***p < 0.001, ns, no significance)

To evaluated the TIME of LUAD patients in TCGA cohort, we quantified the enrichment levels of 29 immune‐associated gene sets (Table S2) including different immune cell types, functions, and signalling pathways by ssGSEA and divided the TCGA patients into three immune clusters according to the ssGSEA scores.^19^, ^20^ The three immune clusters were defined as high‐immunity cluster (HIC), middle‐immunity cluster (MIC) and low‐immunity cluster (LIC). Obvious enrichment differences were shown among three clusters (Figure 1A). Additionally, we applied the index of TME scores to confirm the results of ssGSEA clustering. Being consist with ssGSEA scores, HIC patients had the highest ESTIMATE scores including stromal scores and immune scores, whereas LIC patients had the highest tumour purity scores (Figure 1B). Briefly, HIC samples contained the most proportion of immune components, while LIC samples contained the most proportion of tumour components.

Next, to explore the differences of immune cell proportions among three clusters, we used CIBERSORT algorithm to calculate the percentages of 22 kinds of immune cells in TCGA LUAD samples. A box plot was drawn to exhibit the fraction of 22 kinds of immune cells in different clusters (Figure 1C). As shown in the results, there were significant differences of immune cell proportions: HIC and MIC patients had more proportions of both innate immune cells such as macrophages, monocytes and dendritic cells (DCs) and specific immune cells such as B cells and CD8^+^ T cells than LIC patients. Moreover, we calculated the expressions of three sets of marker genes including immunotherapy target gene, PD‐L1 (Figure 1D), genes for innate immune (Figure 1E) and genes for specific immune (Figure 1F). Box plots were plotted to show that the expressions of all the marker genes ascended from LIC to HIC apparently.

In general, TIME of LUAD patients could be clearly distinguished by ssGSEA. There were the most significant differences between HIC and LIC. LIC patients not only have fewer immune cells proportions, but also have lower levels of immune gene expressions.

### 3.2 13 PDEIRGs between HIC and LIC were obtained through Cox regression hazards analyses

**FIGURE 2:**
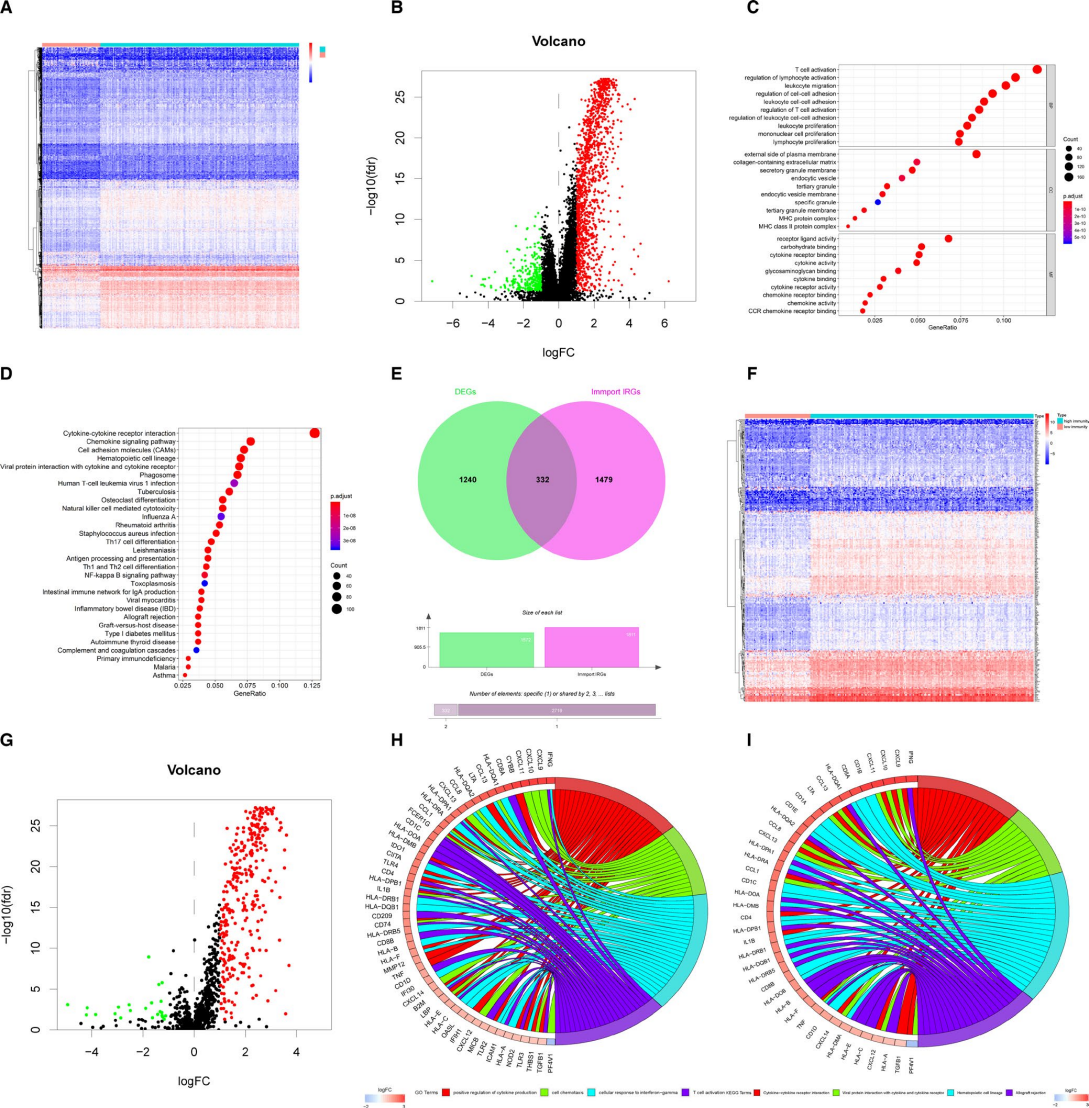
Differential expression analyses and Functional annotations for DEGs and DEIRGs. (A) The heat map shows the DEGs between HIC and LIC determined by R package limma with cut‐off values of FDR < 0.05 and |log2 FC| > 1. Row names were the gene names, and column names were the ID of samples. (B) The volcano plot shows the DEGs between HIC and LIC determined by R package limma with cut‐off values of FDR < 0.05 and |log2 FC| > 1. The red dots represent up‐regulated significant DEGs, and the green dots represent down‐regulated significant DEGs. (C) GO terms enrichment analyses of DEGs in Figure 2A and B. The x‐axis indicates the gene ratio within each GO term. (D) KEGG pathway enrichment analyses of DEGs in Figure 2A and B. The x‐axis indicates the gene ratio within each KEGG pathway. (E) Venn plot shows the obtaining of 332 DEIRGs by intersection between significant DEGs in Figure 2B and 1811 IRGs from ImmPort database. (F) The heat map shows the DEIRGs between HIC and LIC determined by R package limma with cut‐off values of FDR < 0.05 and |log2 FC| > 1. Row names were the gene names, and column names were the ID of samples. (G) The volcano plot shows the DEIRGs between HIC and LIC determined by R package limma with cut‐off values of FDR < 0.05 and |log2 FC| > 1. The red dots represent up‐regulated significant DEIRGs and the green dots represent down‐regulated significant DEIRGs. (H) GO term enrichment analyses of DEIRGs in Figure 2F and G. The x‐axis indicates the gene ratio within each GO term. (I) KEGG pathway enrichment analyses of DEIRGs in Figure 2F and G. The x‐axis indicates the gene ratio within each KEGG pathway

To explore the immune genes that were most closely related to the prognosis of LUAD patients, we filtered all the DEGs between HIC and LIC because of the most remarkable discrepancy between the two groups. To begin with, an obvious different tendency of total transcriptomic gene expressions could be observed in the heat map (Figure 2A). A volcano map (Figure 2B) was plotted to show the 1572 DEGs including 1302 up‐regulated genes and 270 down‐regulated genes (FDR < 0.05, |log_2_ FC| > 1). Further, the enrichment analyses of GO and KEGG were carried out to demonstrate the functional annotation of these DEGs (Figure 2C and D). The DEGs were prominently enriched in both immune‐related GO of immune cell activation and proliferation such as T‐cell activation of biological process (adjust *p* value = 2.09E‐69) and immune‐related pathways such as the chemokine signalling pathway (adjust *p* value = 1.98E‐27) and NF‐κB signalling pathway (adjust *p* value = 1.46E‐17). These again proved the successfully clustering by ssGSEA.

Next, to identify DEIRGs, we utilized 1811 IRGs obtained from the ImmPort Database (Table S3) to intersect with total 1572 DEGs. As a result shown in the venn diagram (Figure 2E),^21^ 332 DEIRGs were gained totally. Compared with genes in the LIC, 309 genes were up‐regulated and 23 genes were down‐regulated (Figure 2F and G). Again, we performed GO and KEGG functional enrichment analyses of 332 DEIRGs (Figure 2H and I). We found that there was a high functional enrichment of cytokine production and cytokine–cytokine receptor interaction. Here, we inferred that cytokine production and the interaction between cytokines and immune cells might play a key role in TIME.

**FIGURE 3:**
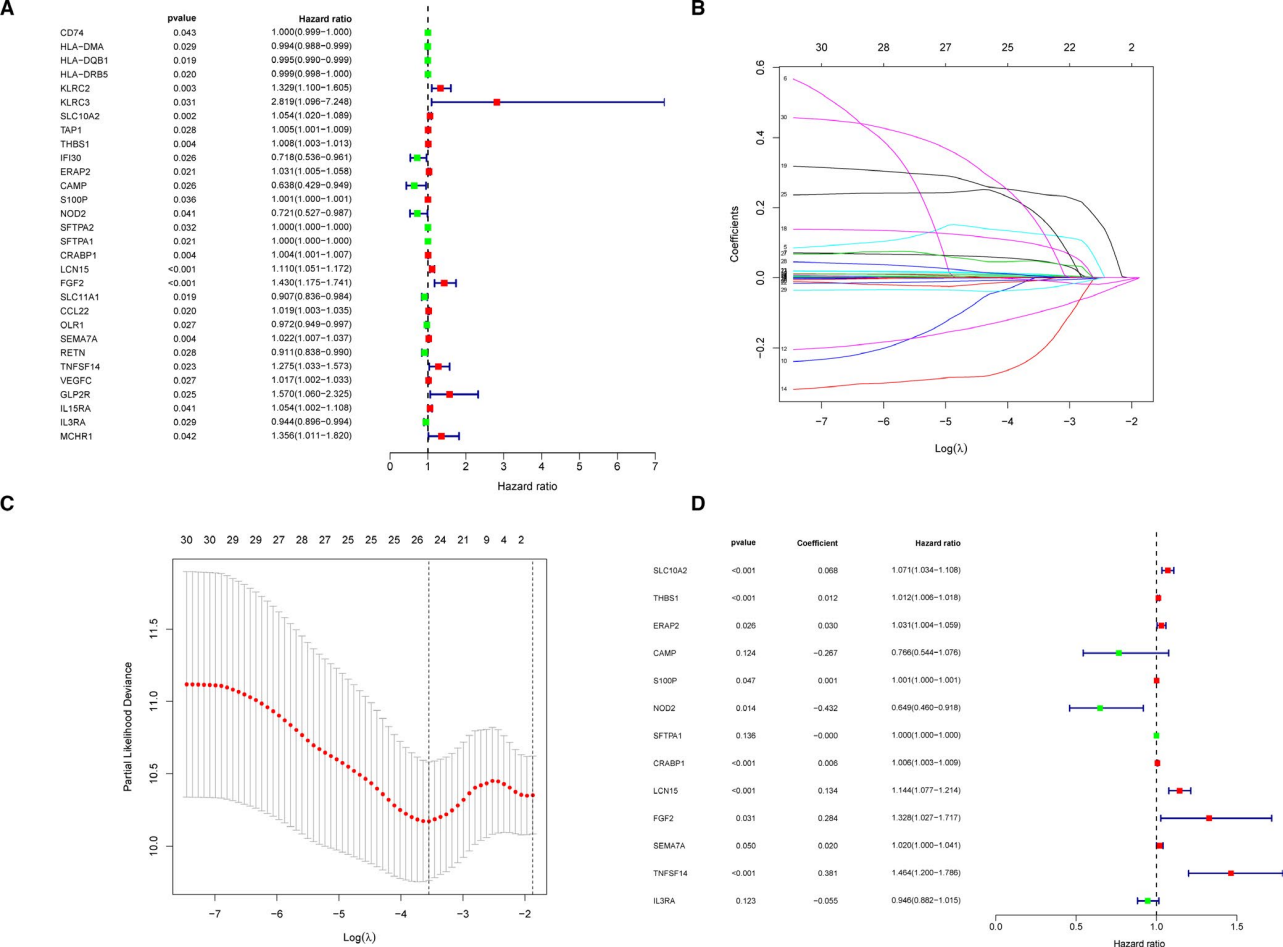
13 PDEIRGs between HIC and LIC were obtained through Cox regression hazards analyses. (A) Thirty candidate PDEIRGs with the significance criteria of *p *< 0.05 were obtained through the univariate Cox analysis. (B and C) Lasso regression analysis of 30 candidate PDEIRGs in Figure 3A. (D) The forest plot shows 13 PDEIRGs determined by the multivariate Cox analysis, and the IPM was built based on the 13 PDEIRGs

To investigate the relationship between expressions of 332 DEIRGs and prognosis of LUAD patients, we involved 332 DEIRGs into Cox regression hazards analyses. Firstly, we applied a univariate Cox hazard regression analysis to screen all the DEIRGs of patients in HIC and LIC. Thirty genes with significant *p* value were established as candidate PDEIRGs (Figure 3A). Then, we utilized the Lasso regression to eliminate five PDEIRGs that might over‐fit the model (λ = 25, Figure 3B and C). Next, the reserved 25 candidate genes were included in the multivariate Cox hazard regression analysis for further assessment. Finally, 13 PDEIRGs were established including genes associated with antigen processing and presentation such as SLC10A2, THBS1, ERAP2 and genes taking part in anti‐microbial such as S100P, NOD2 and LCN15 (Figure 3D).

Furthermore, we used the 13 PDEIRGs to build up an IPM. The risk scores of each patient were calculated according to our formula mentioned before. All the patients were divided into the high‐risk score group and the low‐risk score group (Figure S1A). High‐risk patients had much shorter survival times (Figure S1B). In addition, the expressions of 13 PDEIRGs in two groups were displayed by the heat map (Figure S1C). The Kaplan–Meier curve was used to show that the significantly higher survival rates in the low‐risk group (*p *= 2.116E‐07, Figure S1D). After that, the time‐dependent ROC curve with the AUC value of 0.709 was plotted to confirm the predictive accuracy of the model for LUAD patients (Figure S1E). Finally, we again put the risk scores and clinical characteristics of patients into the univariate and multivariate Cox hazard regression analysis, as shown in Figure S1F and G. Significant *p* values could be found in the index of TNM stage [HR = 1.777 (1.088–2.900), *p *= 0.021] and the risk scores [HR = 1.029 (1.018–1.040), *p *< 0.001]. Our results showed that LUAD patients with high‐risk scores and advanced tumour stages had a low survival rate and poor prognosis.

### 3.3 NOD2 was identified as negative prognostic factor in LUAD and played a role of negative regulation in tumour immune microenvironment

**FIGURE 4:**
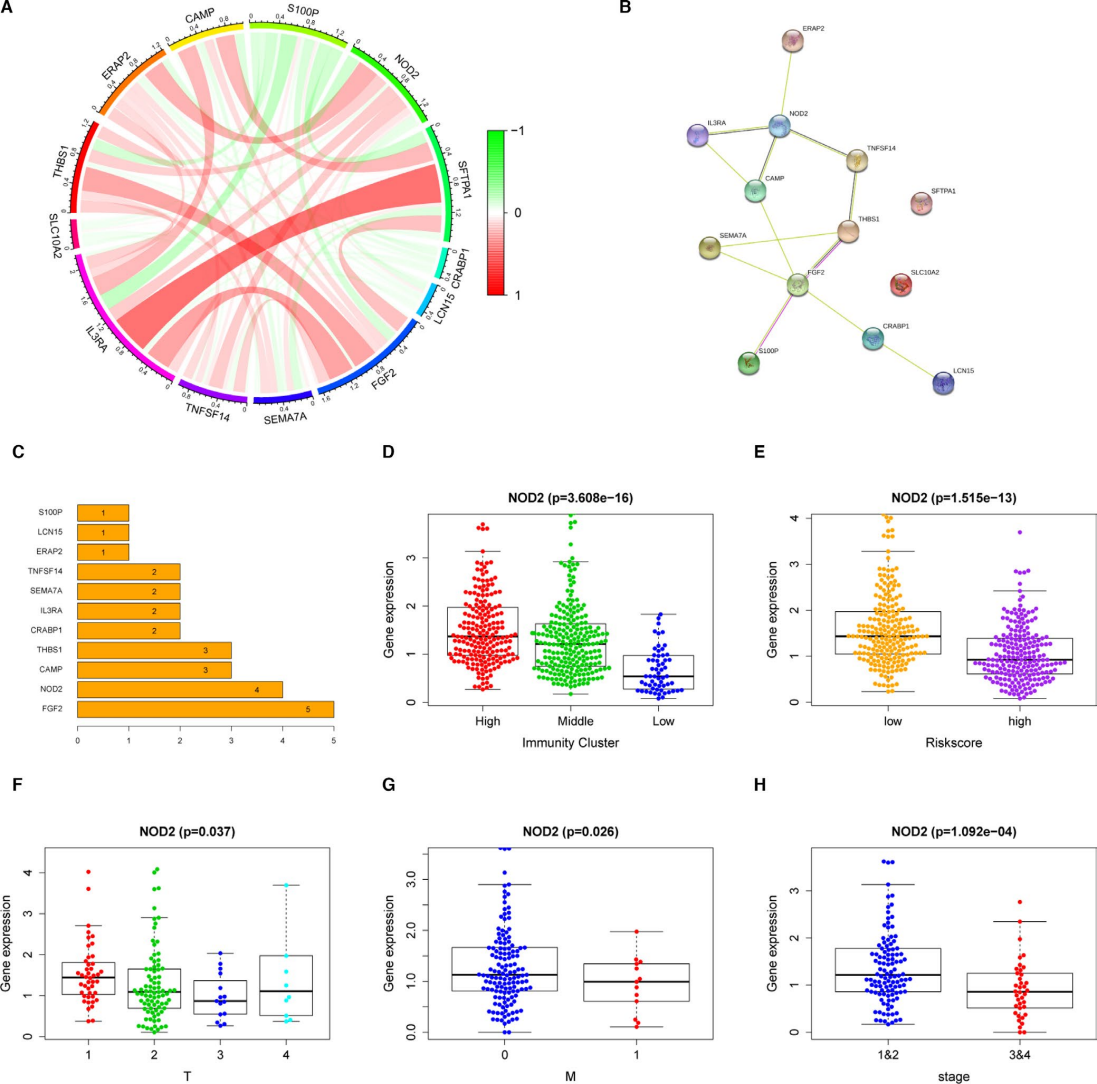
NOD2 was the key immune gene in the TME and acted as a tumour suppressor in LUAD. (A) Gene expression correlations of 13 PDEIRGs. Lines between two genes indicate the correlation value calculated by Pearson coefficient of correlation test. (B and C) PPI networks of 13 PDEIRGs from STRING database (B) and ordered by the number of connected nodes (C). (D) NOD2 expressions in different immunity clusters of the entire TCGA LUAD patients (*n* = 422). (E) NOD2 expressions in the high‐risk group and the low‐risk group of the entire TCGA LUAD patients (*n* = 422). (F) NOD2 expressions of the entire TCGA LUAD patients with different tumour sizes (T stage, *n* = 422). (G) NOD2 expressions of the entire TCGA LUAD patients with and without metastasis (M stage, *n* = 422). (H) NOD2 expressions of the entire TCGA LUAD patients with different TNM stages (*n* = 422)

To ascertain which gene played a key role in TIME, we compared the coefficient values of all 13 PDEIRGs each other. As shown in Figure 3D, NOD2 had the maximum negative coefficient value of −0.432 and HR value of 0.649 (0.460–0.918), suggesting its potent protective role in LUAD. Then, to evaluate the interactions among 13 PDEIRGs, we examined the expression correlations and PPI networks of 13 PDEIRGs (Figure 4A–C). We found that NOD2 was a hub gene because of many interactions with other PDEIRGs. Next, we calculated 13 PDEIRGs expression values to assess the consistency of gene expressions and the clustering method including ssGSEA clustering and risk score clustering. Lower NOD2 gene expression was found in the LIC which was thought to have more tumour components (Figure 4D). Likewise, lower NOD2 gene expression was found in low‐risk score cluster which was thought to have poor prognosis (Figure 4E). After that, since the multivariate Cox analysis had shown that TNM stage was an independent prognostic factor, we estimated the correlations between 13 PDEIRG expressions and clinical TNM stage, including T stage and M stage. Surprisingly, among all the 13 PDEIRGs, only NOD2 had a positive correlation with tumour stage: descended NOD2 expressions were found in larger primary tumours, metastatic tumours and advanced tumours (Figure 4F–H). Collectively, based on our comprehensive assessments, NOD2 was believed to be the central immune gene in the IPM and played a potent role in TIME. Descended expression of NOD2 indicated not only tumour progression but also poor prognosis.

### 3.4 The expressions of NOD2 in macrophages were decreased after co‐culturing with LUAD cells

**FIGURE 5:**
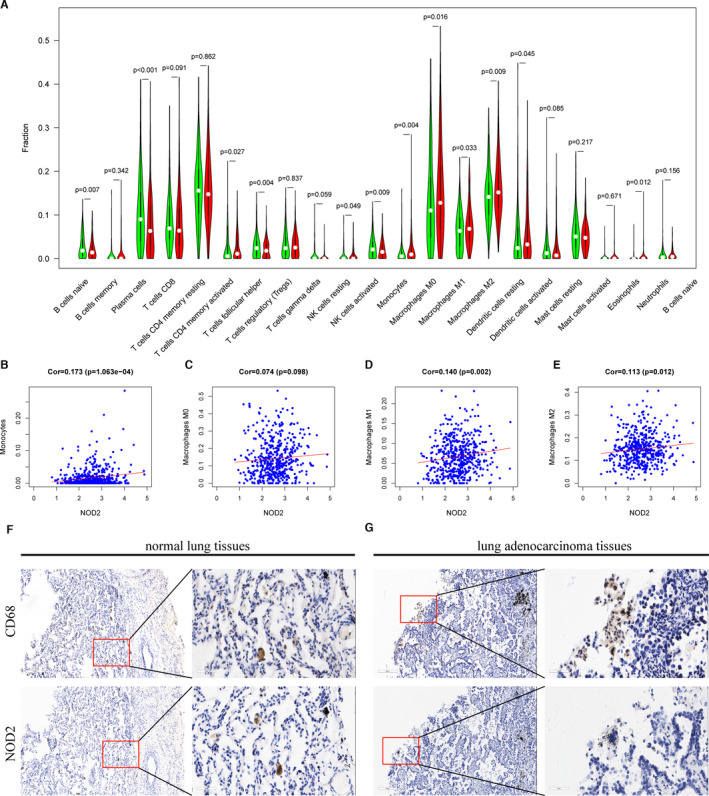
NOD2 was mainly expressed in the macrophages. (A) The fractions of 22 kinds of immune cells of patients with high NOD2 expressions (red) and low NOD2 expressions (green) in the TCGA LUAD cohort (*n* = 422). (B–E) Gene expression correlations between NOD2 and monocytes, macrophages M0, macrophages M1, macrophages M2, respectively, in the TCGA LUAD cohort (*n* = 422). (F and G) Representative pictures of the expressions of CD68 and NOD2 in LUAD patients tested by immunohistochemistry (*n* = 3)

**FIGURE 6:**
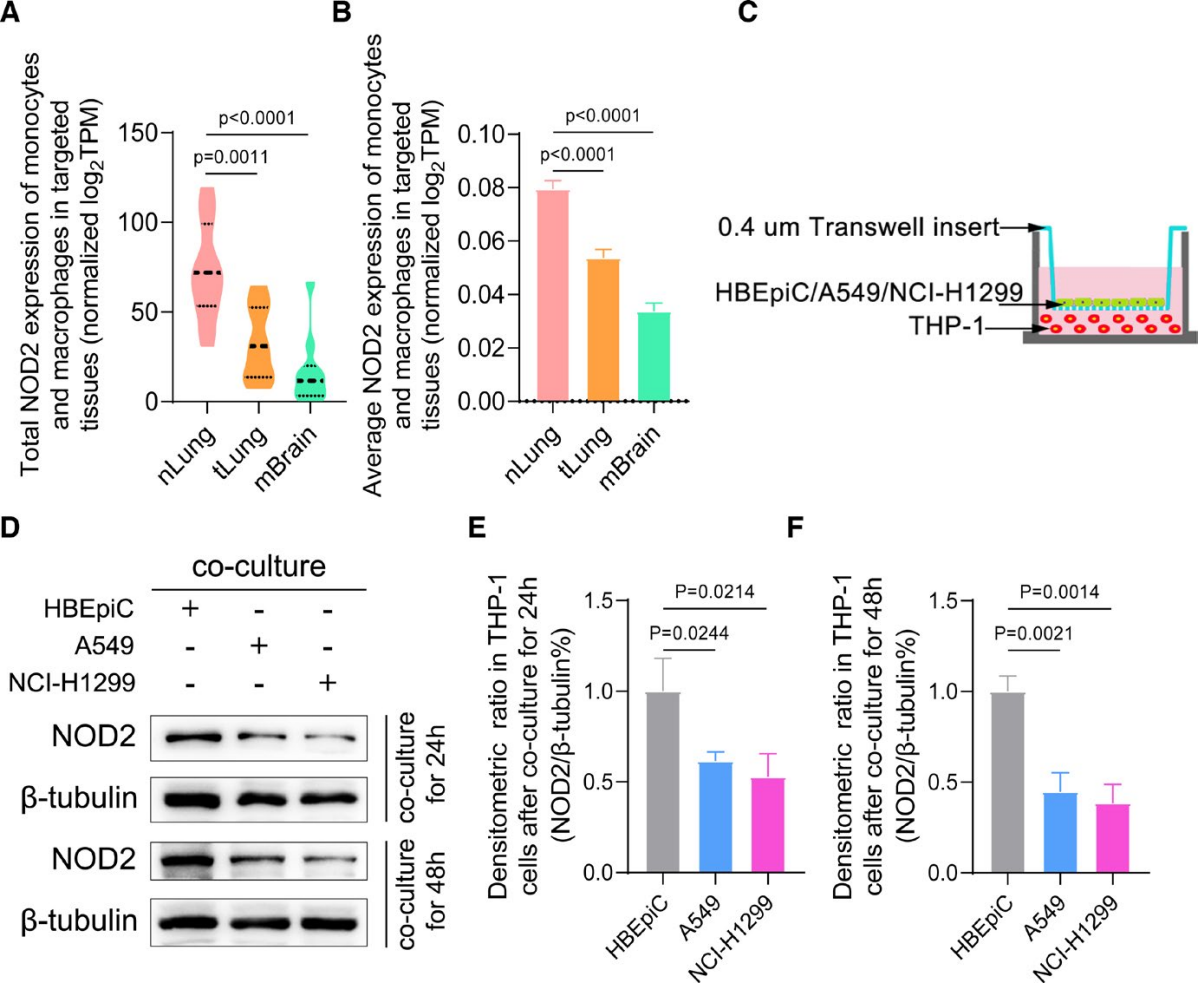
Decreased NOD2 expressions in TAMs were induced by LUAD cells. (A) Total NOD2 expression values of mo‐Mac in nLung, tLung and mBrain samples of GEO cohort. (B) Average NOD2 expression values of mo‐Mac in nLung, tLung and mBrain samples of GEO cohort (mean + SEM). (C) The schemes of the in vitro cellular co‐cultured experiment. (D) The level of NOD2 expressions in the co‐culture system was tested by Western blot. Representative pictures are shown. (E and F): The average densitometric values of NOD2 expressions are shown by histograms normalized to the levels of β‐tubulin (mean + SD). Abbreviations: mBrain, metastatic brain tissue samples; nLung, distant normal lung samples; SD, standard deviation; SEM, standard error of mean; tLung, primary tumour samples; TPM, transcripts per million

Since our previous results suggested that cytokine production and the interaction between cytokines and immune cells might play a key role in TIME (Figure 2H and I), we made further efforts to discuss the relationships between NOD2 and immune cells. A total of 422 TCGA LUAD patients were divided into two groups according to median expression level of NOD2. The proportions of 22 kinds of immune cells of patients in the two groups were statistically analysed. As shown in Figure 5A, decreased NOD2 expressions were found in patients with low monocytes and macrophage fractions (*p *< 0.05). Furthermore, we analyse the correlations between NOD2 expressions and CIBERSORT algorithm results of TAMs. It was proved that NOD2 expressions were positively related to TAMs (Figure 5B–E). Moreover, the results from immunohistochemistry showed that there was the same immune‐positive signal for NOD2 and CD68, a macrophage marker molecule, in tumour tissues (Figure 5F and G). There was an obvious decline in NOD2 expressions in primary lung cancer tissues than those of normal lung tissues. In order to further confirm these results, we used the single‐cell RNA sequencing (scRNA‐Seq) data of 58 LUAD patients from GEO database to make a conjoint analysis.^22^ The NOD2 expression values were calculated in the samples of 11 distant normal lungs, 11 primary tumours and 10 metastatic brain tissues. The decreased total and average NOD2 expressions in TAMs were found in both malignant tissues than in normal tissues (Figure 6A and B). To explore whether the depletion of NOD2 in TAMs was induced by cancer cells, we co‐cultured either HBEpiC or LUAD cells with THP‐1 (Figure 6C). After 24 h and 48 h co‐culture, lower NOD2 expressions were found in THP‐1 co‐cultured with cancer cells (Figure 6D–F). These results suggested that the expressions of NOD2 in monocytes and macrophages were decreased when these monocytes and macrophages were recruited into the TME.

### 3.5 Tumour cells spurred the conversion of the protective M1 phenotype to pro‐tumorigenic M2 subtype by down‐regulating NOD2 expression in TAMs

**FIGURE 7:**
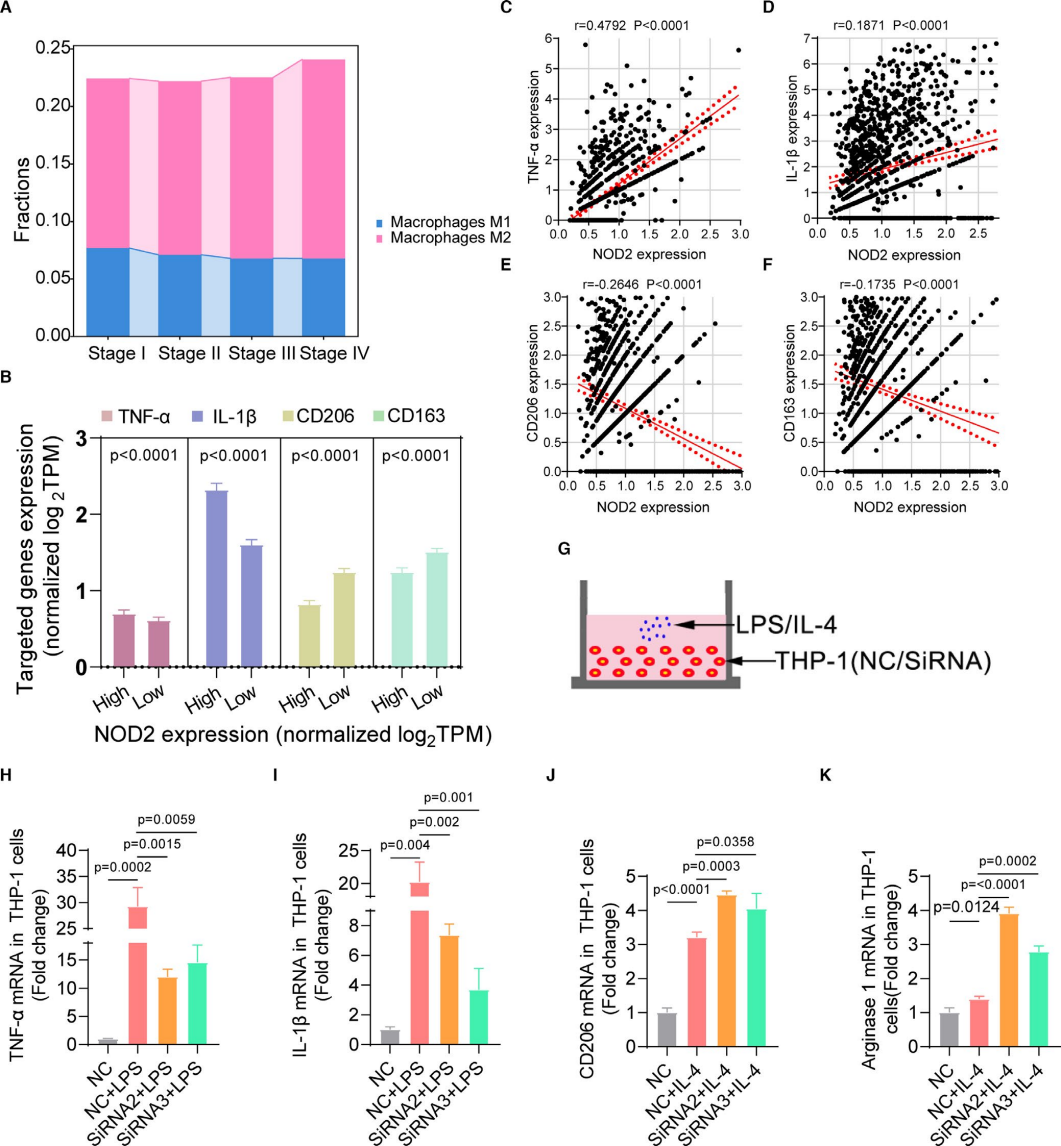
Tumour cells spurred the conversion of the protective M1 phenotype to pro‐tumorigenic M2 subtype by down‐regulating NOD2 expression in TAMs. (A) Average fractions of M1 and M2 macrophages in different TNM stage patients of TCGA cohort. (B) Gene expressions of TNF‐α, IL‐1β, CD206 and CD163 in the high NOD2 expression group and the low NOD2 group in the GEO malignant samples. X‐axis represents the high NOD2 expression group and the low NOD2 group. Y‐axis represents normalized TPM values of targeted genes (mean + SEM). (C–F) The correlations of gene expressions between NOD2 and marker genes of M1 or M2 macrophages in the GEO LUAD cohort. (G) The schemes of the in vitro RNA interfered THP‐1 co‐cultured with LPS or IL‐4. (H–K) Gene expressions of TNF‐α, IL‐1β, CD206 and arginase 1 in RNA interfered THP‐1 co‐cultured with LPS or IL‐4 were tested by Q‐PCR (mean + SD)

It had been well reported that macrophages could be differentiated into M1 or M2 phenotype under microenvironment stimulus.^23^ M1 macrophages had a pro‐inflammatory and antitumour effect, whereas M2 macrophages could secrete cytokines and chemokines to enhance proliferative and pro‐metastatic effect on the tumour cells.^24^ TCGA data showed that there were fewer M1 and more M2 proportions in advanced stage patients than early‐stage patients (Figure 7A), which was consistent with the changeable tendency of NOD2 expressions (Figure 4F). To further explain whether there was a correlation between the phenotypic conversion of macrophages and the depleted expression of NOD2, we divided the monocytes and macrophages in GEO cohort into high NOD2 group and low NOD2 group. As shown in Figure 7B, macrophages with high NOD2 expression secreted more TNF‐α and IL‐1β, M1‐specific markers, while macrophages with low NOD2 expressed more CD206 and CD163, M2‐specific markers. Moreover, NOD2 expressions were positively correlated to M1 markers and negatively correlated to M2 markers (Figure 7C–F). These results provide a clue that lung cancer cells induced the M1‐to‐M2 phenotypic switch by down‐regulating NOD2 expression in macrophages. To further confirm these findings, we first used Q‐PCR to examine the mRNA levels of phenotypic markers in THP‐1 cells within co‐culture system (Figure 6C). After co‐cultured with cancer cells, THP‐1 cells expressed less M1 markers and more M2 markers (Figure S2). Next, after 48 h of transfection with NOD2 siRNAs, the expressions of NOD2 in THP‐1 cells were significantly knocked down (Figure S3). Subsequently, LPS, a M1 stimulus, or IL‐4, a M2 stimulus, was applied for the cellular co‐culture system, respectively (Figure 7G). Compared to the control group, knockdown of NOD2 could inhibit the expression of M1 markers under LPS stimulation. Conversely, the expression of M2 markers was obviously increased after addition of IL‐4 to the cellular co‐culture system (Figure 7H–K). Taken together, our data suggested that the reduction of NOD2 in macrophages might be involved in the phenotypic conversion from the protective M1 phenotype to pro‐tumorigenic M2 subtype.

In addition, to answer the possible molecular mechanism of phenotypic conversion mediated by NOD2, we performed the GSEA using TCGA data. As shown in Figure S4A, NF‐κB signalling pathway was significantly enriched (*p *< 0.05). Since it had been reported that NF‐κB pathway could be activated through CARD‐CARD domain interactions between NOD2 and RIPK2, we again analysed the previous scRNA‐Seq data of TAMs to evaluate the expression values and correlations of RIPK2. Consequently, a sharp decline of RIPK2 expressions in the low NOD2 group and a strong correlation was obtained and shown in Figure S4B and C. Further, we used NF‐κB pathway antibody kit to evaluate the activation of the NF‐κB pathway in LPS‐stimulated NOD2‐silencing THP‐1. After NOD2 expression was knocked down by SiRNA2 and SiRNA3, the phosphorylation of NF‐κB pathway inhibitors, IKKα/β (Ser176/180) and IκBα (Ser32), was significantly decreased. Moreover, the activation of transcription factor p65/RelA (Ser536) was obviously impeded (Figure S4D). It appeared that descended NOD2 in TAMs impeded the formation of the active NOD2‐RIPK2 complex and then down‐regulated the NF‐κB signalling pathway by which means, at least partly, macrophages converted from M1 phenotype to M2 subtype.

## 4 DISCUSSION

Serving as a critical role in cancer immune escape leading to tumour growth and aggressiveness,^25^ the TME signature was a robust biomarker for predicting survivals of tumour patients and providing effective immunotherapy strategies.^26^, ^27^ On the one hand, immune cells could eliminate tumour cells not only by regulating the expression levels of IRGs at certain immune checkpoints,^28^, ^29^ but also by activating IRGs pathways which could turn immune cells from a naive into an activated functional status.^30^ On the other, the cancer cells in the TME could reconstruct IRGs expression patterns by mimicking normal cells to avoid the destruction which was called cancer immunoediting.^31^, ^32^ In this study, we were the first to create the IPM based on the ssGSEA clustering and identified a tumour inhibitor, NOD2. Meanwhile, we elucidated the possible mechanism of TAM phenotypic conversion mediated by deceased NOD2 in promoting tumour development through the combination of integrated multiple omics analyses and molecular biology experiments.

NOD2 was a member of the NLR family and played an important role in both innate and adaptive immune response, apoptosis, autophagy and reactive oxygen species generation.^33^ The unbalanced level of NOD signalling was associated with diseases by breaking immune homeostasis.^34^ It had been previously shown that a lack of NOD2 signalling could increase Crohn's disease susceptibility using mouse models.^35^ Recently, NOD2 had been connected to cancer development and treatment.^36^ Diogo Branquinho et al. reported that NOD2 deficiency increased risk of colitis and colitis‐associated colorectal cancer because of dysbiosis.^37^ Lener's group found that the 3020insC mutation of the NOD2 might be a genetic predisposing factor for aggregations of lung cancer.^38^ NOD2 deficiency in TME could not only induce inflammatory diseases but also resulted in cancers. In accord with previous studies, we reported LUAD patients with decreased NOD2 had a disposition to tumour progression and bad prognosis.

It had been extensively reviewed and demonstrated about the mechanisms of NOD2 in host defence against pathogens.^39^, ^40^, ^41^ However, the role of NOD2 in innate immune cells, especially macrophages, was only partially understood.^41^ An model of NOD2 signalling had been proposed to demonstrate that NOD2 could be activated by the recruitment of the downstream adaptor RIPK2 through homotypic CARD‐CARD interactions to form a large signalling platform.^42^ Triggered NOD2 signalling could drive the innate immune response through the activation of NF‐κB, MAPK and caspase‐1 pathways, which lead to increased production of pro‐inflammatory factors such as TNF‐α, IL‐1β, IL‐6 and IL‐12p40. The innate immune cells including NK cells, inflammatory macrophages and DCs could be recruited and primed by the increased pro‐inflammatory factors.^43^ On the one hand, the recruited innate immune cells killed the pathogens directly by secreting NO, H_2_O_2_, O_2_^−^, etc., or by mediating antibody‐dependent cell‐mediated cytotoxicity (ADCC) and phagocytosis (ADCP). On the other, the innate immune cells also released cytokines and chemokines to drive more innate cells and developed antigen‐presenting function to take part in regulating specific T‐cell immune response.^2^ A recent study had shown a pro‐inflammatory microenvironment in the intestines of both NOD2‐deficient and RIPK2‐deficient increased susceptibility to colon cancer.^34^ Consist with previous researches, our study found that decreased NOD2‐RIPK2 expression in LUAD patients might suppress the NF‐κB pathway and lead to a pro‐tumorigenic TAMs functional status.

Macrophages could be classified into different subtypes according to their extremely plastic phenotypes and highly dynamic functions. The increased proportion of M2 TAMs in the TME facilitated tumour immune escape and chemoresistance development. Immunotherapy strategies targeted at TME especially M2 macrophages have been amply explored including blocking macrophages infiltration,^44^ eliminating predominant M2 TAMs,^45^ reprogramming M2 TAMs into the M1 phenotype^46^ and delivering therapeutics medicated by TAMs.^47^ Recently, Carlos W et al. used mouse tumour model to demonstrate that paclitaxel inhibited tumour progression by reprogramming the phenotypes of TAMs from a M2 pro‐tumour subtype to a M1 antitumour subtype in a TLR4‐dependent manner.^48^ Serving as one of intracellular NTRs, NOD2 contained many similarities to TLRs, membrane‐bound pattern recognition receptors (PRPs), in the processing of recognizing pathogen‐associated motifs and inducing inflammatory signalling cascades.^49^ Nathaniel J. Buteyn et al. found that activation of NOD2 in innate immune cells promoted acute myeloid leukaemia (AML) cell apoptosis and provided a survival advantage.^50^ However, the therapeutic potential of NOD2 in human LUAD had not been fully explored. In this study, descended NOD2 expression‐mediated TAM phenotypic conversion in LUAD patients had been found and investigated by bioinformatics methods and molecular biology experiments. Our results provided clues and theoretical basis for the formation and development of NOD2‐mediated TAM‐targeted immunotherapeutic strategies. As a retrospective research, there were several limitations in our study. Prospective studies should be further conducted and the exact roles and mechanisms of NOD2 on TAMs in the TME should be deeply investigated to provide benefit strategies of TAM‐targeted immunotherapy for LUAD patients.

In summary, we clustered the TIME of LUAD patients by ssGSEA and performed an immune gene prognosis analysis. Our data supported the notion that decreased NOD2 leads to an inhibited innate immune status by mediating TAMs phenotypic conversion in the TME which contributed to tumour progression and poor prognosis of LUAD patients. Notably, this study provides a potential strategy to boost TAM‐targeted immunotherapies in order to bring clinical interest for LUAD patients.

## CONFLICT OF INTEREST

The authors have no conflicts of interests and declare no competing financial interests.

## AUTHOR CONTRIBUTIONS

**Yibei Wang:** Formal analysis (equal); Software (equal); Visualization (equal); Writing‐original draft (equal). **Ziwei Miao:** Software (equal); Visualization (equal). **Xiaoxue Qin:** Formal analysis (equal). **Bo Li:** Funding acquisition (equal); Writing‐original draft (equal). **Yun Han:** Conceptualization (lead); Funding acquisition (equal).

## Supporting information

Figure S1

Figure S2

Figure S3

Figure S4

Table S1

Table S2

Table S3

## Data Availability

The data sets used and/or analysed during the current study are available from the corresponding author on reasonable request.
