# Supplementary material for: NOD2 deficiency confers a pro‐tumorigenic macrophage phenotype to promote lung adenocarcinoma progression
Source: J Cell Mol Med. 2021 Jul 16;25(15):7545–58. doi: 10.1111/jcmm.16790 (PMC8335701; doi:10.1111/jcmm.16790)
Supplement: Supplementary file 1 — Figure S1 [file JCMM-25-7545-s007.docx]

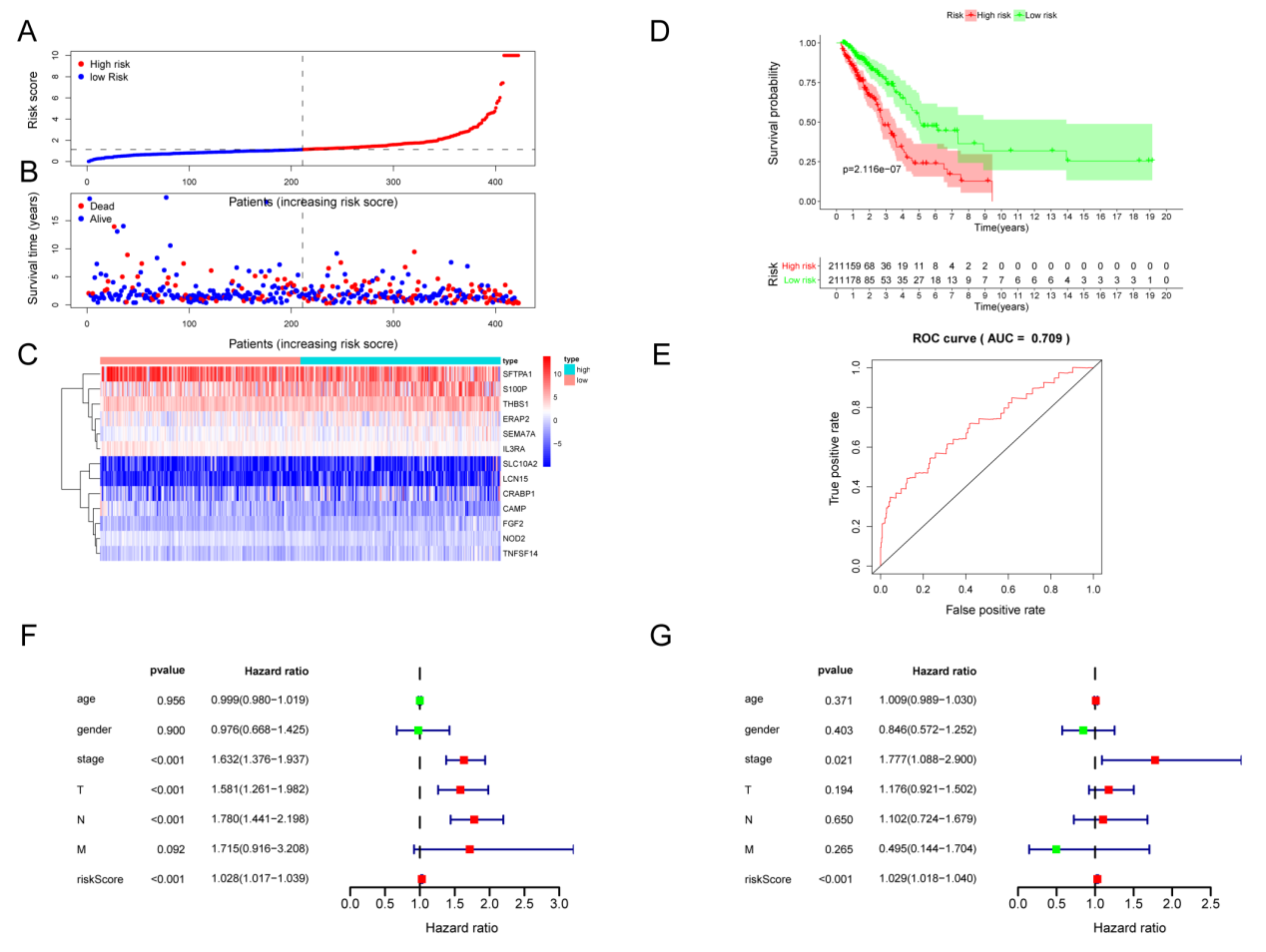


**Figure S1. Evaluation of the prognostic efficacy of the IPM.**

(A) Risk scores distribution of 422 TCGA cohort patients. X axis represented 422 patients and Y axis represented risk scores.

(B) Survival status scatter plots for 422 TCGA cohort patients. X axis represented 422 patients and Y axis represented the survival time.

(C)Heat map showed the expression patterns of 13 PDEIRGs. Row name represented gene names and column name represented patients in high and low risk groups.

(D) Kaplan-Meier curves for patients in high and low risk groups. Log-rank test showed P=2.116E-07.

(E) Time dependent ROC curve analysis of the prognostic model showed AUC=0.709.

(F) Univariate Cox hazard regression analysis of different clinical characters including age, gender, stage, T, N, M status and riskscore.

(G) Multivariate Cox hazard regression analysis of different clinical characters including age, gender, stage, T, N, M status and riskscore.
