# Supplementary material for: NOD2 deficiency confers a pro‐tumorigenic macrophage phenotype to promote lung adenocarcinoma progression
Source: J Cell Mol Med. 2021 Jul 16;25(15):7545–58. doi: 10.1111/jcmm.16790 (PMC8335701; doi:10.1111/jcmm.16790)
Supplement: Supplementary file 2 — Figure S2 [file JCMM-25-7545-s004.docx]

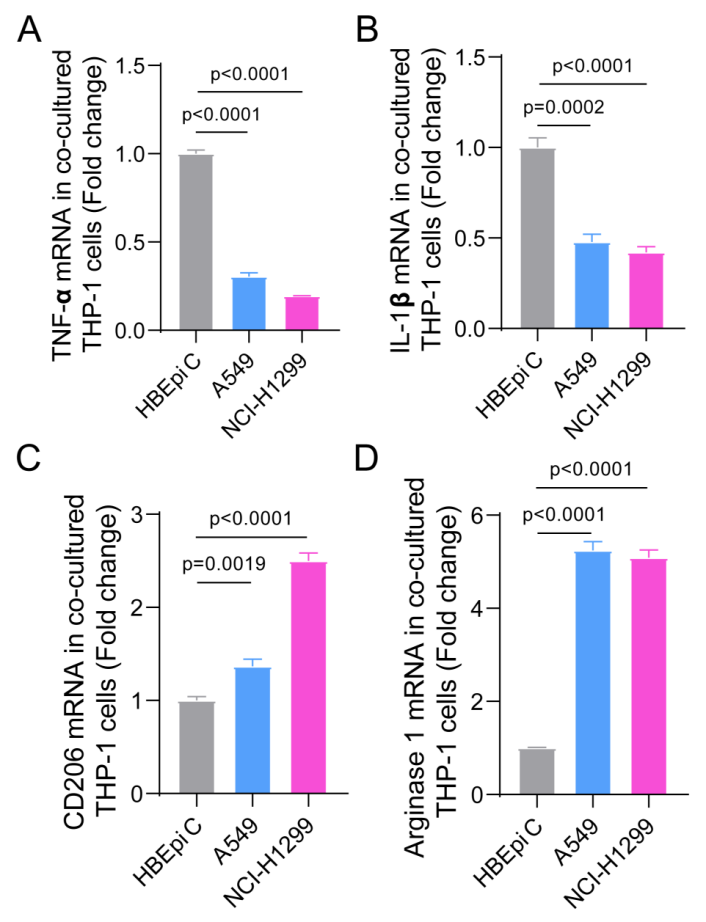


**Figure S2. NOD2 expressions of THP-1 decreased after co-cultured with LUAD cells.**

(A-D) Gene expressions of TNF-α, IL-1β, CD206 and Arginase 1 in THP-1 in the co-culture system were tested by Q-PCR. (Mean+SD).
