# Supplementary material for: NOD2 deficiency confers a pro‐tumorigenic macrophage phenotype to promote lung adenocarcinoma progression
Source: J Cell Mol Med. 2021 Jul 16;25(15):7545–58. doi: 10.1111/jcmm.16790 (PMC8335701; doi:10.1111/jcmm.16790)
Supplement: Supplementary file 3 — Figure S3 [file JCMM-25-7545-s005.docx]

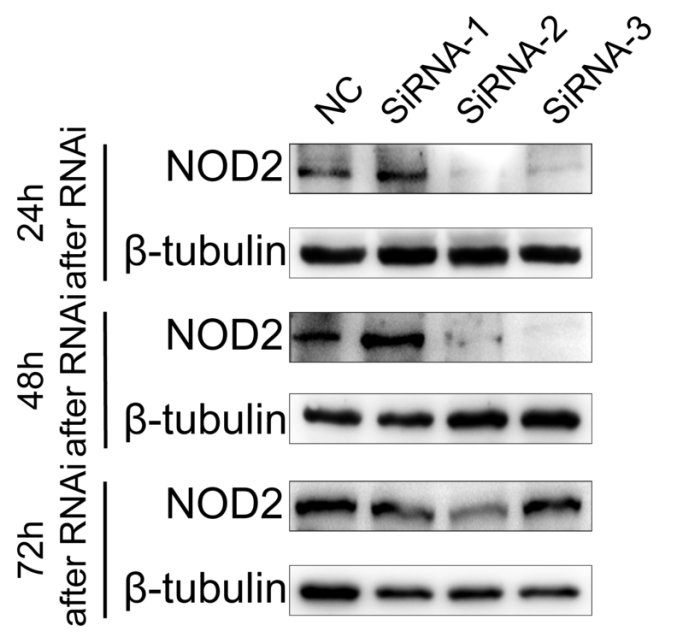


**Figure S3. The levels of NOD2 expression after RNA interfering were tested by western blot at indicated time point.**
