# Supplementary material for: NOD2 deficiency confers a pro‐tumorigenic macrophage phenotype to promote lung adenocarcinoma progression
Source: J Cell Mol Med. 2021 Jul 16;25(15):7545–58. doi: 10.1111/jcmm.16790 (PMC8335701; doi:10.1111/jcmm.16790)
Supplement: Supplementary file 4 — Figure S4 [file JCMM-25-7545-s002.docx]

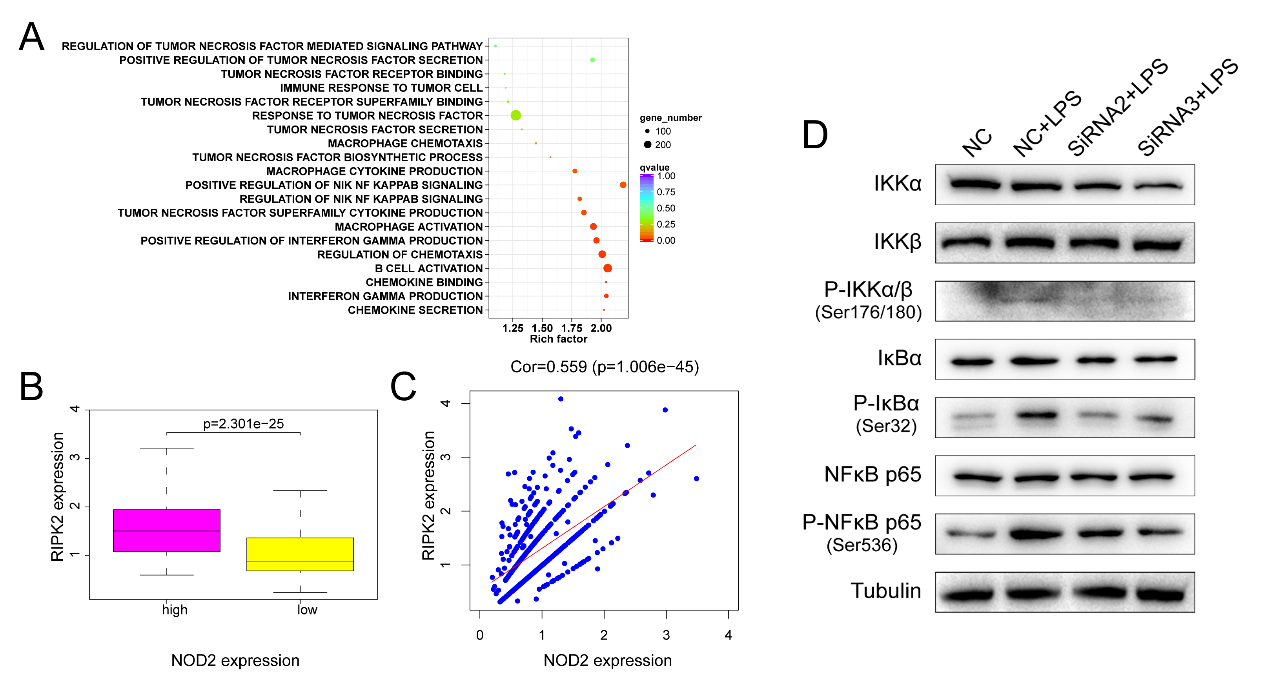


**Figure S4. Possible molecular mechanism of phenotypic conversion mediated by NOD2**

(A) GO terms of NOD2 GSEA.

(B) Gene expressions of NF-κB and RIPK2 in the high NOD2 expression group and the low NOD2 group in the GEO malignant samples. X axis represented the high NOD2 expression group and the low NOD2 group. Y axis represented TPM values of gene expressions.

(C) The gene expression correlation between NOD2 and RIPK2 in the GEO LUAD cohort.

(D) Evaluation of the canonical NF-κB pathway in LPS-stimulated NOD2-silencing THP-1 cell line.
