# Supplementary material for: NOD2 deficiency confers a pro‐tumorigenic macrophage phenotype to promote lung adenocarcinoma progression
Source: J Cell Mol Med. 2021 Jul 16;25(15):7545–58. doi: 10.1111/jcmm.16790 (PMC8335701; doi:10.1111/jcmm.16790)
Supplement: Supplementary file 5 — Table S1 [file JCMM-25-7545-s003.docx]

**Supplementary Table**

Table S1. The sequences of primers for quantitative real-time PCR.

| **Target** | **5’-3’ Oligonucleotide sequence,**  **forward, reverse** | **Genbank Accession No.** |
| --- | --- | --- |
| Human  IL-1β | GCTACGAATCTCCGACCAC  ACATAAGCCTCGTTATCCCA | NM_000576.3 |
| Human TNF-α, | GCATCGCCGTCTCCTACCAG  CGCTGAGTCGGTCACCCTTC | NM_000594.4 |
| Human arginase 1 | AAGCCTATTGACTACCTTAACCC ATGCCATTAACTATGAGATTTATATCGG | [NM_000045.4](http://www.ncbi.nlm.nih.gov/entrez/query.fcgi?cmd=Search&amp;db=Nucleotide&amp;term=NM_007482) |
| Human CD206 | ATACCTGCGACAGTAAACGA TTGCAGTATGTCTCCGCTTC | NM_002438.4 |
| Human GAPDH | AGGCTGGGGCTCATTTGCAG  TGGTGGTGCAGGAGGCATTG | NM_002046.7 |
