# Supplementary material for: NOD2 deficiency confers a pro‐tumorigenic macrophage phenotype to promote lung adenocarcinoma progression
Source: J Cell Mol Med. 2021 Jul 16;25(15):7545–58. doi: 10.1111/jcmm.16790 (PMC8335701; doi:10.1111/jcmm.16790)
Supplement: Supplementary file 6 — Table S2 [file JCMM-25-7545-s001.docx]

**Supplementary Table**

Table S2. The list of 29 immune-associated gene sets.

| **Names** | **Gene sets** | | | | | | | | | |
| --- | --- | --- | --- | --- | --- | --- | --- | --- | --- | --- |
| aDCs | CD83 | LAMP3 | CCL1 |  |  |  |  |  |  |  |
| APC-co-inhibition | C10orf54 | CD274 | LGALS9 | PDCD1LG2 | PVRL3 |  |  |  |  |  |
| APC-co-stimulation | CD40 | CD58 | CD70 | ICOSLG | SLAMF1 | TNFSF14 | TNFSF15 | TNFSF18 | TNFSF4 | TNFSF8 |
|  | TNFSF9 |  |  |  |  |  |  |  |  |  |
| B-cells | BACH2 | BANK1 | BLK | BTLA | CD79A | CD79B | FCRL1 | FCRL3 | HVCN1 | RALGPS2 |
| CCR | CCL16 | TPO | TGFBR2 | CXCL2 | CCL14 | TGFBR3 | IL11RA | CCL11 | IL4I1 | IL33 |
|  | CXCL10 | BMPER | BMP8A | CXCL11 | IL21R | IL17B | TNFRSF9 | ILF2 | CX3CR1 | CCR8 |
|  | TNFSF12 | CSF3 | TNFSF4 | BMP3 | CX3CL1 | BMP5 | CXCR2 | TNFRSF10D | BMP2 | CXCL14 |
|  | CCL28 | CXCL3 | BMP6 | CCL21 | CXCL9 | CCL23 | IL6 | TNFRSF18 | IL17RD | IL17D |
|  | IL27 | CCL7 | IL1R1 | CXCR4 | CXCR2P1 | TGFB1I1 | IFNGR1 | IL9R | IL1RAPL1 | IL11 |
|  | CSF1 | IL20RA | IL25 | TNFRSF4 | IL18 | ILF3 | CCL20 | TNFRSF12A | IL6ST | CXCL13 |
|  | IL12B | TNFRSF8 | IL6R | BMPR2 | IFNE | IL1RAPL2 | IL3RA | BMP4 | CCL24 | TNFSF13B |
|  | CCR4 | IL2RA | IL32 | TNFRSF10C | IL22RA1 | BMPR1A | CXCR5 | CXCR3 | IFNA8 | IL17REL |
|  | IFNB1 | IFNAR1 | TNFRSF1B | CCL17 | IFNL1 | IL16 | IL1RL1 | ILK | CCL25 | ILDR2 |
|  | IL36RN | IL34 | TGFB1 | IFNG | IL19 | ILKAP | BMP2K | CCR10 | ILDR1 | EPO |
|  | CCR7 | IL17C | IL23A | CCR5 | IL7 | EPOR | CCL13 | IL2RG | IL31RA | TNFAIP6 |
|  | IFNL2 | BMP1 | IL12RB1 | TNFAIP8 | IL4R | TNFRSF6B | TNFAIP8L1 | TNFRSF10B | IFNL3 | CCL5 |
|  | CXCL6 | CXCL1 | CCR3 | TNFSF11 | CSF1R | IL21 | IL1RAP | IL12RB2 | CCL1 | IL17RA |
|  | CCR1 | IL1RN | TNFRSF11B | TNFRSF14 | IL13 | IL2RB | BMP8B | CCL2 | IL24 | IL18RAP |
|  | TGFBI | TNFSF10 | TNFRSF11A | CXCL5 | IL5RA | TNFSF9 | IL1RL2 | TNFRSF13C | IL36G | IL15RA |
|  | TNFRSF21 | CXCL8 | IL22RA2 | TNFAIP8L2 | IL18R1 | IFNLR1 | CXCR6 | CCL3L3 | TNFRSF1A | IL17RE |
|  | IFNGR2 | IL17RC | TNFAIP8L3 | ILVBL | TGFBRAP1 | CCL4L1 | CSF2RA | CCRN4L | CCL26 | TNFAIP1 |
|  | CCRL2 | IFNA10 | TNFRSF17 | IFNA13 | IL20 | IL18BP | CCL3L1 | TNFSF12-TNFSF13 | IL5 | IL23R |
|  | IL26 | TNF | TGFA | CSF2 | IL1F10 | CXCL17 | TNFSF13 | IFNA4 | IL37 | IL12A |
|  | IL7R | IFNA1 | IL1A | IL4 | IL2 | CCL22 | CSF3R | IL10 | IFNK | TGFB2 |
|  | IL1R2 | IL1B | IL17F | IL27RA | IL15 | TNFSF8 | IL36B | XCL1 | CXCL16 | TNFRSF19 |
|  | IL3 | CCL3 | IFNA2 | BMPR1B | IFNA21 | TNFSF18 | CCL8 | IL17RB | TNFRSF25 | IL22 |
|  | IL10RB | IFNAR2 | CCL18 | IFNA16 | CSF2RB | IL36A | TNFAIP3 | IL13RA2 | IL13RA1 | CCR9 |
|  | TNFRSF10A | IFNA7 | IFNW1 | XCL2 | TNFSF14 | CCR2 | BMP15 | BMP10 | CCL15-CCL14 | TGFBR1 |
|  | IFNA5 | BMP7 | IFNA14 | IL20RB | IL10RA | IFNA17 | CCR6 | TGFB3 | CCL15 | CCL4 |
|  | CCL27 | TNFRSF13B | TNFAIP2 | IL31 | IL17A | TNFSF15 | CCL19 | IFNA6 | IL9 | CXCR1 |
|  | CXCL12 |  |  |  |  |  |  |  |  |  |
| CD8+-T-cells | CD8A |  |  |  |  |  |  |  |  |  |
| Check-point | IDO1 | LAG3 | CTLA4 | TNFRSF9 | ICOS | CD80 | PDCD1LG2 | TIGIT | CD70 | TNFSF9 |
|  | ICOSLG | KIR3DL1 | CD86 | PDCD1 | LAIR1 | TNFRSF8 | TNFSF15 | TNFRSF14 | IDO2 | CD276 |
|  | CD40 | TNFRSF4 | TNFSF14 | HHLA2 | CD244 | CD274 | HAVCR2 | CD27 | BTLA | LGALS9 |
|  | TMIGD2 | CD28 | CD48 | TNFRSF25 | CD40LG | ADORA2A | VTCN1 | CD160 | CD44 | TNFSF18 |
|  | TNFRSF18 | BTNL2 | C10orf54 | CD200R1 | TNFSF4 | CD200 | NRP1 |  |  |  |
| Cytolytic-activity | PRF1 | GZMA |  |  |  |  |  |  |  |  |
| DCs | CCL17 | CCL22 | CD209 | CCL13 |  |  |  |  |  |  |
| HLA | HLA-E | HLA-DPB2 | HLA-C | HLA-J | HLA-DQB1 | HLA-DQB2 | HLA-DQA2 | HLA-DQA1 | HLA-A | HLA-DMA |
|  | HLA-DOB | HLA-DRB1 | HLA-H | HLA-B | HLA-DRB5 | HLA-DOA | HLA-DPB1 | HLA-DRA | HLA-DRB6 | HLA-L |
|  | HLA-F | HLA-G | HLA-DMB | HLA-DPA1 |  |  |  |  |  |  |
| iDCs | CD1A | CD1E |  |  |  |  |  |  |  |  |
| Inflammation-promoting | CCL5 | CD19 | CD8B | CXCL10 | CXCL13 | CXCL9 | GNLY | GZMB | IFNG | IL12A |
|  | IL12B | IRF1 | PRF1 | STAT1 | TBX21 |  |  |  |  |  |
| Macrophages | C11orf45 | CD68 | CLEC5A | CYBB | FUCA1 | GPNMB | HS3ST2 | LGMN | MMP9 | TM4SF19 |
| Mast-cells | CMA1 | MS4A2 | TPSAB1 |  |  |  |  |  |  |  |
| MHC-class-I | B2M | HLA-A | TAP1 |  |  |  |  |  |  |  |
| Neutrophils | EVI2B | HSD17B11 | KDM6B | MEGF9 | MNDA | NLRP12 | PADI4 | SELL | TRANK1 | VNN3 |
| NK-cells | KLRC1 | KLRF1 |  |  |  |  |  |  |  |  |
| Parainflammation | CXCL10 | PLAT | CCND1 | LGMN | PLAUR | AIM2 | MMP7 | ICAM1 | MX2 | CXCL9 |
|  | ANXA1 | TLR2 | PLA2G2D | ITGA2 | MX1 | HMOX1 | CD276 | TIRAP | IL33 | PTGES |
|  | TNFRSF12A | SCARB1 | CD14 | BLNK | IFIT3 | RETNLB | IFIT2 | ISG15 | OAS2 | REL |
|  | OAS3 | CD44 | PPARG | BST2 | OAS1 | NOX1 | PLA2G2A | IFIT1 | IFITM3 | IL1RN |
| pDCs | CLEC4C | CXCR3 | GZMB | IL3RA | IRF7 | IRF8 | LILRA4 | PHEX | PLD4 | PTCRA |
| T-cell-co-inhibition | BTLA | C10orf54 | CD160 | CD244 | CD274 | CTLA4 | HAVCR2 | LAG3 | LAIR1 | TIGIT |
| T-cell-co-stimulation | CD2 | CD226 | CD27 | CD28 | CD40LG | ICOS | SLAMF1 | TNFRSF18 | TNFRSF25 | TNFRSF4 |
|  | TNFRSF8 | TNFRSF9 | TNFSF14 |  |  |  |  |  |  |  |
| T-helper-cells | CD4 |  |  |  |  |  |  |  |  |  |
| Tfh | PDCD1 | CXCL13 | CXCR5 |  |  |  |  |  |  |  |
| Th1-cells | IFNG | TBX21 | CTLA4 | STAT4 | CD38 | IL12RB2 | LTA | CSF2 |  |  |
| Th2-cells | PMCH | LAIR2 | SMAD2 | CXCR6 | GATA3 | IL26 |  |  |  |  |
| TIL | ITM2C | CD38 | THEMIS2 | GLYR1 | ICOS | F5 | TIGIT | KLRD1 | IRF4 | PRKCQ |
| FCRL5 | SIRPG | LPXN | IL2RG | CCL5 | LCK | TRAF3IP3 | CD86 | MAL | LILRB1 | DOK2 |
|  | CD6 | PAG1 | LAX1 | PLEK | PIK3CD | SLAMF1 | XCL1 | GPR171 | XCL2 | TBX21 |
|  | CD2 | CD53 | KLHL6 | SLAMF6 | CD40 | SIT1 | TNFRSF4 | CD79A | CD247 | LCP2 |
|  | CD3D | CD27 | SH2D1A | FYB | ARHGAP30 | ACAP1 | CST7 | CD3G | IL2RB | CD3E |
|  | FCRL3 | CORO1A | ITK | TCL1A | CYBB | CSF2RB | IKZF1 | NCF4 | DOCK2 | CCR2 |
|  | PTPRC | PLAC8 | NCKAP1L | IL7R | 6-Sep | CD28 | STAT4 | CD8A | LY9 | CD48 |
|  | HCST | PTPRCAP | SASH3 | ARHGAP25 | LAT | TRAT1 | IL10RA | PAX5 | CCR7 | DOCK11 |
|  | PARVG | SPNS1 | CD52 | HCLS1 | ARHGAP9 | GIMAP6 | PRKCB | MS4A1 | GPR18 | TBC1D10C |
|  | GVINP1 | P2RY8 | EVI2B | VAMP5 | KLRK1 | SELL | MPEG1 | MS4A6A | ARHGAP15 | MFNG |
|  | GZMK | SELPLG | TARP | GIMAP7 | FAM65B | INPP5D | ITGA4 | MZB1 | GPSM3 | STK10 |
|  | CLEC2D | IL16 | NLRC3 | GIMAP5 | GIMAP4 | IFFO1 | CFH | PVRIG | CFHR1 |  |
| Treg | IL12RB2 | TMPRSS6 | CTSC | LAPTM4B | TFRC | RNF145 | NETO2 | ADAT2 | CHST2 | CTLA4 |
|  | NFE2L3 | LIMA1 | IL1R2 | ICOS | HSDL2 | HTATIP2 | FKBP1A | TIGIT | CCR8 | LTA |
|  | SLC35F2 | IL21R | AHCYL1 | SOCS2 | ETV7 | BCL2L1 | RRAGB | ACSL4 | CHRNA6 | BATF |
|  | LAX1 | ADPRH | TNFRSF4 | ANKRD10 | CD274 | CASP1 | LY75 | NPTN | SSTR3 | GRSF1 |
|  | CSF2RB | TMEM184C | NDFIP2 | ZBTB38 | ERI1 | TRAF3 | NAB1 | HS3ST3B1 | LAYN | JAK1 |
|  | VDR | LEPROT | GCNT1 | PTPRJ | IKZF2 | CSF1 | ENTPD1 | TNFRSF18 | METTL7A | KSR1 |
|  | SSH1 | CADM1 | IL1R1 | ACP5 | CHST7 | THADA | CD177 | NFAT5 | ZNF282 | MAGEH1 |
| Type-I-IFN-Reponse | DDX4 | IFIT1 | IFIT2 | IFIT3 | IRF7 | ISG20 | MX1 | MX2 | RSAD2 | TNFSF10 |
| Type-II-IFN-Reponse | GPR146 | SELP | AHR |  |  |  |  |  |  |  |
