# Supplementary material for: NOD2 deficiency confers a pro‐tumorigenic macrophage phenotype to promote lung adenocarcinoma progression
Source: J Cell Mol Med. 2021 Jul 16;25(15):7545–58. doi: 10.1111/jcmm.16790 (PMC8335701; doi:10.1111/jcmm.16790)
Supplement: Supplementary file 7 — Table S3 [file JCMM-25-7545-s006.docx]

**Supplementary Table**

Table S3.The 1811 IRGs information downloaded from Immport database.

| Symbol | ID | Name | Synonyms | Chromosome | Category |
| --- | --- | --- | --- | --- | --- |
| AZGP1 | 563 | alpha-2-glycoprotein 1, zinc-binding | ZA2G\|ZAG | 7 | Antigen Processing and Presentation |
| B2M | 567 | beta-2-microglobulin | - | 15 | Antigen Processing and Presentation |
| CALR | 811 | calreticulin | CRT\|FLJ26680\|RO\|SSA\|cC1qR | 19 | Antigen Processing and Presentation |
| CANX | 821 | calnexin | CNX\|FLJ26570\|IP90\|P90 | 5 | Antigen Processing and Presentation |
| CD1A | 909 | CD1a molecule | CD1\|FCB6\|HTA1\|R4\|T6 | 1 | Antigen Processing and Presentation |
| CD1B | 910 | CD1b molecule | CD1\|CD1A\|MGC125990\|MGC125991\|R1 | 1 | Antigen Processing and Presentation |
| CD1C | 911 | CD1c molecule | BDCA1\|CD1\|CD1A\|R7 | 1 | Antigen Processing and Presentation |
| CD1D | 912 | CD1d molecule | CD1A\|MGC34622\|R3 | 1 | Antigen Processing and Presentation |
| CD1E | 913 | CD1e molecule | CD1A\|R2 | 1 | Antigen Processing and Presentation |
| CD4 | 920 | CD4 molecule | CD4mut | 12 | Antigen Processing and Presentation |
| CD8A | 925 | CD8a molecule | CD8\|Leu2\|MAL\|p32 | 2 | Antigen Processing and Presentation |
| CD8B | 926 | CD8b molecule | CD8B1\|LYT3\|Leu2\|Ly3\|MGC119115 | 2 | Antigen Processing and Presentation |
| CD74 | 972 | CD74 molecule, major histocompatibility complex, class II invariant chain | DHLAG\|HLADG\|Ia-GAMMA | 5 | Antigen Processing and Presentation |
| CREB1 | 1385 | cAMP responsive element binding protein 1 | CREB\|MGC9284 | 2 | Antigen Processing and Presentation |
| CTSB | 1508 | cathepsin B | APPS\|CPSB | 8 | Antigen Processing and Presentation |
| CTSE | 1510 | cathepsin E | CATE | 1 | Antigen Processing and Presentation |
| CTSL1 | 1514 | cathepsin L1 | CATL\|CTSL\|FLJ31037\|MEP | 9 | Antigen Processing and Presentation |
| CTSS | 1520 | cathepsin S | MGC3886 | 1 | Antigen Processing and Presentation |
| FCER1G | 2207 | Fc fragment of IgE, high affinity I, receptor for; gamma polypeptide | FCRG | 1 | Antigen Processing and Presentation |
| FCGRT | 2217 | Fc fragment of IgG, receptor, transporter, alpha | FCRN\|alpha-chain | 19 | Antigen Processing and Presentation |
| PDIA3 | 2923 | protein disulfide isomerase family A, member 3 | ER60\|ERp57\|ERp60\|ERp61\|GRP57\|GRP58\|HsT17083\|P58\|PI-PLC | 15 | Antigen Processing and Presentation |
| HFE | 3077 | hemochromatosis | HFE1\|HH\|HLA-H\|MGC103790\|MVCD7\|dJ221C16.10.1 | 6 | Antigen Processing and Presentation |
| HLA-A | 3105 | major histocompatibility complex, class I, A | FLJ26655\|HLAA | 6 | Antigen Processing and Presentation |
| HLA-B | 3106 | major histocompatibility complex, class I, B | AS\|HLA-B-7301\|HLA-B73\|HLAB\|HLAC\|MGC111087\|SPDA1 | 6 | Antigen Processing and Presentation |
| HLA-C | 3107 | major histocompatibility complex, class I, C | D6S204\|FLJ27082\|HLA-Cw\|HLA-Cw12\|HLA-JY3\|HLC-C\|PSORS1 | 6 | Antigen Processing and Presentation |
| HLA-DMA | 3108 | major histocompatibility complex, class II, DM alpha | D6S222E\|DMA\|HLADM\|RING6 | 6 | Antigen Processing and Presentation |
| HLA-DMB | 3109 | major histocompatibility complex, class II, DM beta | D6S221E\|RING7 | 6 | Antigen Processing and Presentation |
| HLA-DOA | 3111 | major histocompatibility complex, class II, DO alpha | HLA-DNA\|HLA-DZA\|HLADZ | 6 | Antigen Processing and Presentation |
| HLA-DOB | 3112 | major histocompatibility complex, class II, DO beta | DOB | 6 | Antigen Processing and Presentation |
| HLA-DPA1 | 3113 | major histocompatibility complex, class II, DP alpha 1 | HLA-DP1A\|HLADP\|HLASB | 6 | Antigen Processing and Presentation |
| HLA-DPB1 | 3115 | major histocompatibility complex, class II, DP beta 1 | DPB1\|HLA-DP1B | 6 | Antigen Processing and Presentation |
| HLA-DQA1 | 3117 | major histocompatibility complex, class II, DQ alpha 1 | CD\|CELIAC1\|DQ-A1\|FLJ27088\|FLJ27328\|GSE\|HLA-DQA\|MGC149527 | 6 | Antigen Processing and Presentation |
| HLA-DQA2 | 3118 | major histocompatibility complex, class II, DQ alpha 2 | HLA-DXA | 6 | Antigen Processing and Presentation |
| HLA-DQB1 | 3119 | major histocompatibility complex, class II, DQ beta 1 | CELIAC1\|HLA-DQB\|IDDM1 | 6 | Antigen Processing and Presentation |
| HLA-DRA | 3122 | major histocompatibility complex, class II, DR alpha | HLA-DRA1 | 6 | Antigen Processing and Presentation |
| HLA-DRB1 | 3123 | major histocompatibility complex, class II, DR beta 1 | DRB1\|FLJ75017\|FLJ76359\|HLA-DR1B\|HLA-DRB\|HLA-DRB1*\|SS1 | 6 | Antigen Processing and Presentation |
| HLA-DRB3 | 3125 | major histocompatibility complex, class II, DR beta 3 | HLA-DR3B\|HLA-DR52\|MGC117330 | 6 | Antigen Processing and Presentation |
| HLA-DRB4 | 3126 | major histocompatibility complex, class II, DR beta 4 | DRB4\|HLA-DR4B | 6 | Antigen Processing and Presentation |
| HLA-DRB5 | 3127 | major histocompatibility complex, class II, DR beta 5 | FLJ76359\|HLA-DRB | 6 | Antigen Processing and Presentation |
| HLA-E | 3133 | major histocompatibility complex, class I, E | DKFZp686P19218\|EA1.2\|EA2.1\|HLA-6.2\|MHC\|QA1 | 6 | Antigen Processing and Presentation |
| HLA-F | 3134 | major histocompatibility complex, class I, F | CDA12\|HLA-5.4\|HLA-CDA12\|HLAF | 6 | Antigen Processing and Presentation |
| HLA-G | 3135 | major histocompatibility complex, class I, G | MHC-G | 6 | Antigen Processing and Presentation |
| HLA-H | 3136 | major histocompatibility complex, class I, H (pseudogene) | HLAHP | 6 | Antigen Processing and Presentation |
| MR1 | 3140 | major histocompatibility complex, class I-related | HLALS | 1 | Antigen Processing and Presentation |
| HSPA1A | 3303 | heat shock 70kDa protein 1A | FLJ54303\|FLJ54370\|FLJ54392\|FLJ54408\|FLJ75127\|HSP70-1\|HSP70-1A\|HSP70I\|HSP72\|HSPA1\|HSPA1B | 6 | Antigen Processing and Presentation |
| HSPA1B | 3304 | heat shock 70kDa protein 1B | FLJ54328\|HSP70-1B\|HSP70-2\|HSPA1A | 6 | Antigen Processing and Presentation |
| HSPA1L | 3305 | heat shock 70kDa protein 1-like | HSP70-1L\|HSP70-HOM\|HSP70T\|hum70t | 6 | Antigen Processing and Presentation |
| HSPA2 | 3306 | heat shock 70kDa protein 2 | HSP70-2\|HSP70-3 | 14 | Antigen Processing and Presentation |
| HSPA4 | 3308 | heat shock 70kDa protein 4 | APG-2\|HS24/P52\|MGC131852\|RY\|hsp70\|hsp70RY | 5 | Antigen Processing and Presentation |
| HSPA5 | 3309 | heat shock 70kDa protein 5 (glucose-regulated protein, 78kDa) | BIP\|FLJ26106\|GRP78\|MIF2 | 9 | Antigen Processing and Presentation |
| HSPA6 | 3310 | heat shock 70kDa protein 6 (HSP70B') | - | 1 | Antigen Processing and Presentation |
| HSPA8 | 3312 | heat shock 70kDa protein 8 | HSC54\|HSC70\|HSC71\|HSP71\|HSP73\|HSPA10\|LAP1\|MGC131511\|MGC29929\|NIP71 | 11 | Antigen Processing and Presentation |
| HSP90AA1 | 3320 | heat shock protein 90kDa alpha (cytosolic), class A member 1 | FLJ31884\|HSP86\|HSP89A\|HSP90A\|HSP90N\|HSPC1\|HSPCA\|HSPCAL1\|HSPCAL4\|HSPN\|Hsp89\|Hsp90\|LAP2 | 14 | Antigen Processing and Presentation |
| HSP90AB1 | 3326 | heat shock protein 90kDa alpha (cytosolic), class B member 1 | D6S182\|FLJ26984\|HSP90-BETA\|HSP90B\|HSPC2\|HSPCB | 6 | Antigen Processing and Presentation |
| ICAM1 | 3383 | intercellular adhesion molecule 1 | BB2\|CD54\|P3.58 | 19 | Antigen Processing and Presentation |
| IFNA1 | 3439 | interferon, alpha 1 | IFL\|IFN\|IFN-ALPHA\|IFNA13\|IFNA@\|MGC138207\|MGC138505\|MGC138507 | 9 | Antigen Processing and Presentation |
| IFNA2 | 3440 | interferon, alpha 2 | IFNA\|INFA2\|MGC125764\|MGC125765 | 9 | Antigen Processing and Presentation |
| IFNA4 | 3441 | interferon, alpha 4 | INFA4\|MGC142200 | 9 | Antigen Processing and Presentation |
| IFNA5 | 3442 | interferon, alpha 5 | INFA5 | 9 | Antigen Processing and Presentation |
| IFNA6 | 3443 | interferon, alpha 6 | - | 9 | Antigen Processing and Presentation |
| IFNA7 | 3444 | interferon, alpha 7 | IFNA-J | 9 | Antigen Processing and Presentation |
| IFNA8 | 3445 | interferon, alpha 8 | - | 9 | Antigen Processing and Presentation |
| IFNA10 | 3446 | interferon, alpha 10 | MGC119878\|MGC119879 | 9 | Antigen Processing and Presentation |
| IFNA13 | 3447 | interferon, alpha 13 | - | 9 | Antigen Processing and Presentation |
| IFNA14 | 3448 | interferon, alpha 14 | LEIF2H\|MGC125756\|MGC125757 | 9 | Antigen Processing and Presentation |
| IFNA16 | 3449 | interferon, alpha 16 | - | 9 | Antigen Processing and Presentation |
| IFNA17 | 3451 | interferon, alpha 17 | IFNA\|INFA\|LEIF2C1 | 9 | Antigen Processing and Presentation |
| IFNA21 | 3452 | interferon, alpha 21 | MGC126687\|MGC126689 | 9 | Antigen Processing and Presentation |
| IFNG | 3458 | interferon, gamma | IFG\|IFI | 12 | Antigen Processing and Presentation |
| KIR2DL1 | 3802 | killer cell immunoglobulin-like receptor, two domains, long cytoplasmic tail, 1 | CD158A\|KIR-K64\|KIR221\|NKAT\|NKAT1\|p58.1 | 19 | Antigen Processing and Presentation |
| KIR2DL2 | 3803 | killer cell immunoglobulin-like receptor, two domains, long cytoplasmic tail, 2 | CD158B1\|CD158b\|NKAT6\|p58.2 | 19 | Antigen Processing and Presentation |
| KIR2DL3 | 3804 | killer cell immunoglobulin-like receptor, two domains, long cytoplasmic tail, 3 | CD158B2\|CD158b\|GL183\|KIR-023GB\|KIR-K7b\|KIR-K7c\|KIRCL23\|MGC129943\|NKAT\|NKAT2\|NKAT2A\|NKAT2B\|p58 | 19 | Antigen Processing and Presentation |
| KIR2DL4 | 3805 | killer cell immunoglobulin-like receptor, two domains, long cytoplasmic tail, 4 | CD158D\|G9P\|KIR103\|KIR103AS | 19 | Antigen Processing and Presentation |
| KIR2DS1 | 3806 | killer cell immunoglobulin-like receptor, two domains, short cytoplasmic tail, 1 | CD158H\|CD158a\|p50.1 | 19 | Antigen Processing and Presentation |
| KIR2DS3 | 3808 | killer cell immunoglobulin-like receptor, two domains, short cytoplasmic tail, 3 | NKAT7 | 19 | Antigen Processing and Presentation |
| KIR2DS4 | 3809 | killer cell immunoglobulin-like receptor, two domains, short cytoplasmic tail, 4 | CD158I\|KIR1D\|KIR412\|KKA3\|MGC120019\|MGC125315\|MGC125317\|NKAT8 | 19 | Antigen Processing and Presentation |
| KIR2DS5 | 3810 | killer cell immunoglobulin-like receptor, two domains, short cytoplasmic tail, 5 | CD158G\|NKAT9 | 19 | Antigen Processing and Presentation |
| KIR3DL1 | 3811 | killer cell immunoglobulin-like receptor, three domains, long cytoplasmic tail, 1 | CD158E1\|KIR\|MGC119726\|MGC119728\|MGC126589\|MGC126591\|NKAT3\|NKB1\|NKB1B | 19 | Antigen Processing and Presentation |
| KIR3DL2 | 3812 | killer cell immunoglobulin-like receptor, three domains, long cytoplasmic tail, 2 | CD158K\|MGC125321\|NKAT4\|NKAT4B\|p140 | 19 | Antigen Processing and Presentation |
| KLRC1 | 3821 | killer cell lectin-like receptor subfamily C, member 1 | CD159A\|MGC13374\|MGC59791\|NKG2\|NKG2A | 12 | Antigen Processing and Presentation |
| KLRC2 | 3822 | killer cell lectin-like receptor subfamily C, member 2 | CD159c\|MGC138244\|NKG2-C\|NKG2C | 12 | Antigen Processing and Presentation |
| KLRC3 | 3823 | killer cell lectin-like receptor subfamily C, member 3 | NKG2-E\|NKG2E | 12 | Antigen Processing and Presentation |
| KLRD1 | 3824 | killer cell lectin-like receptor subfamily D, member 1 | CD94 | 12 | Antigen Processing and Presentation |
| LTA | 4049 | lymphotoxin alpha (TNF superfamily, member 1) | LT\|TNFB\|TNFSF1 | 6 | Antigen Processing and Presentation |
| CIITA | 4261 | class II, major histocompatibility complex, transactivator | C2TA\|CIITAIV\|MHC2TA\|NLRA | 16 | Antigen Processing and Presentation |
| MICA | 4276 | MHC class I polypeptide-related sequence A | FLJ60820\|MGC111087\|PERB11.1 | 6 | Antigen Processing and Presentation |
| MICB | 4277 | MHC class I polypeptide-related sequence B | PERB11.2 | 6 | Antigen Processing and Presentation |
| NFYA | 4800 | nuclear transcription factor Y, alpha | CBF-A\|CBF-B\|FLJ11236\|HAP2\|NF-YA | 6 | Antigen Processing and Presentation |
| NFYB | 4801 | nuclear transcription factor Y, beta | CBF-A\|CBF-B\|HAP3\|NF-YB | 12 | Antigen Processing and Presentation |
| NFYC | 4802 | nuclear transcription factor Y, gamma | CBF-C\|CBFC\|DKFZp667G242\|FLJ45775\|H1TF2A\|HAP5\|HSM\|NF-YC | 1 | Antigen Processing and Presentation |
| LGMN | 5641 | legumain | AEP\|LGMN1\|PRSC1 | 14 | Antigen Processing and Presentation |
| PSMB8 | 5696 | proteasome (prosome, macropain) subunit, beta type, 8 (large multifunctional peptidase 7) | D6S216\|D6S216E\|LMP7\|MGC1491\|PSMB5i\|RING10\|beta5i | 6 | Antigen Processing and Presentation |
| PSMC1 | 5700 | proteasome (prosome, macropain) 26S subunit, ATPase, 1 | MGC24583\|MGC8541\|P26S4\|S4\|p56 | 14 | Antigen Processing and Presentation |
| PSMC2 | 5701 | proteasome (prosome, macropain) 26S subunit, ATPase, 2 | MGC3004\|MSS1\|Nbla10058\|S7 | 7 | Antigen Processing and Presentation |
| PSMC3 | 5702 | proteasome (prosome, macropain) 26S subunit, ATPase, 3 | MGC8487\|TBP1 | 11 | Antigen Processing and Presentation |
| PSMC4 | 5704 | proteasome (prosome, macropain) 26S subunit, ATPase, 4 | MGC13687\|MGC23214\|MGC8570\|MIP224\|S6\|TBP7 | 19 | Antigen Processing and Presentation |
| PSMC5 | 5705 | proteasome (prosome, macropain) 26S subunit, ATPase, 5 | S8\|SUG-1\|SUG1\|TBP10\|TRIP1\|p45\|p45/SUG | 17 | Antigen Processing and Presentation |
| PSMC6 | 5706 | proteasome (prosome, macropain) 26S subunit, ATPase, 6 | CADP44\|MGC12520\|P44\|SUG2\|p42 | 14 | Antigen Processing and Presentation |
| PSMD1 | 5707 | proteasome (prosome, macropain) 26S subunit, non-ATPase, 1 | MGC133040\|MGC133041\|P112\|Rpn2\|S1 | 2 | Antigen Processing and Presentation |
| PSMD2 | 5708 | proteasome (prosome, macropain) 26S subunit, non-ATPase, 2 | MGC14274\|P97\|Rpn1\|S2\|TRAP2 | 3 | Antigen Processing and Presentation |
| PSMD3 | 5709 | proteasome (prosome, macropain) 26S subunit, non-ATPase, 3 | P58\|RPN3\|S3 | 17 | Antigen Processing and Presentation |
| PSMD4 | 5710 | proteasome (prosome, macropain) 26S subunit, non-ATPase, 4 | AF\|AF-1\|ASF\|MCB1\|Rpn10\|S5A\|pUB-R5 | 1 | Antigen Processing and Presentation |
| PSMD5 | 5711 | proteasome (prosome, macropain) 26S subunit, non-ATPase, 5 | KIAA0072\|MGC23145\|S5B | 9 | Antigen Processing and Presentation |
| PSMD7 | 5713 | proteasome (prosome, macropain) 26S subunit, non-ATPase, 7 | MOV34\|P40\|Rpn8\|S12 | 16 | Antigen Processing and Presentation |
| PSMD8 | 5714 | proteasome (prosome, macropain) 26S subunit, non-ATPase, 8 | HIP6\|HYPF\|MGC1660\|Nin1p\|Rpn12\|S14\|p31 | 19 | Antigen Processing and Presentation |
| PSMD10 | 5716 | proteasome (prosome, macropain) 26S subunit, non-ATPase, 10 | dJ889N15.2\|p28 | X | Antigen Processing and Presentation |
| PSMD11 | 5717 | proteasome (prosome, macropain) 26S subunit, non-ATPase, 11 | MGC3844\|Rpn6\|S9\|p44.5 | 17 | Antigen Processing and Presentation |
| PSMD13 | 5719 | proteasome (prosome, macropain) 26S subunit, non-ATPase, 13 | HSPC027\|Rpn9\|S11\|p40.5 | 11 | Antigen Processing and Presentation |
| PSME1 | 5720 | proteasome (prosome, macropain) activator subunit 1 (PA28 alpha) | IFI5111\|MGC8628\|PA28A\|PA28alpha\|REGalpha | 14 | Antigen Processing and Presentation |
| PSME1 | 5720 | proteasome (prosome, macropain) activator subunit 1 (PA28 alpha) | IFI5111\|MGC8628\|PA28A\|PA28alpha\|REGalpha | 14 | Antigen Processing and Presentation |
| PSME2 | 5721 | proteasome (prosome, macropain) activator subunit 2 (PA28 beta) | PA28B\|PA28beta\|REGbeta | 14 | Antigen Processing and Presentation |
| PSME2 | 5721 | proteasome (prosome, macropain) activator subunit 2 (PA28 beta) | PA28B\|PA28beta\|REGbeta | 14 | Antigen Processing and Presentation |
| RELB | 5971 | v-rel reticuloendotheliosis viral oncogene homolog B | I-REL\|IREL | 19 | Antigen Processing and Presentation |
| RFX5 | 5993 | regulatory factor X, 5 (influences HLA class II expression) | - | 1 | Antigen Processing and Presentation |
| RFXAP | 5994 | regulatory factor X-associated protein | - | 13 | Antigen Processing and Presentation |
| SLC10A2 | 6555 | solute carrier family 10 (sodium/bile acid cotransporter family), member 2 | ASBT\|ISBT\|NTCP2 | 13 | Antigen Processing and Presentation |
| TAP1 | 6890 | transporter 1, ATP-binding cassette, sub-family B (MDR/TAP) | ABC17\|ABCB2\|APT1\|D6S114E\|FLJ26666\|FLJ41500\|PSF1\|RING4\|TAP1*0102N\|TAP1N | 6 | Antigen Processing and Presentation |
| TAP2 | 6891 | transporter 2, ATP-binding cassette, sub-family B (MDR/TAP) | ABC18\|ABCB3\|APT2\|D6S217E\|PSF2\|RING11 | 6 | Antigen Processing and Presentation |
| TAPBP | 6892 | TAP binding protein (tapasin) | NGS17\|TAPA\|TPN\|TPSN\|tapasin | 6 | Antigen Processing and Presentation |
| THBS1 | 7057 | thrombospondin 1 | THBS\|THBS-1\|TSP\|TSP-1\|TSP1 | 15 | Antigen Processing and Presentation |
| SHFM1 | 7979 | split hand/foot malformation (ectrodactyly) type 1 | DSS1\|ECD\|SEM1\|SHFD1\|SHSF1\|Shfdg1 | 7 | Antigen Processing and Presentation |
| KLRC4 | 8302 | killer cell lectin-like receptor subfamily C, member 4 | FLJ17759\|FLJ78582\|NKG2-F\|NKG2F | 12 | Antigen Processing and Presentation |
| AP3B1 | 8546 | adaptor-related protein complex 3, beta 1 subunit | ADTB3\|ADTB3A\|HPS\|HPS2\|PE | 5 | Antigen Processing and Presentation |
| RFXANK | 8625 | regulatory factor X-associated ankyrin-containing protein | ANKRA1\|BLS\|F14150_1\|MGC138628\|RFX-B | 19 | Antigen Processing and Presentation |
| PSMD6 | 9861 | proteasome (prosome, macropain) 26S subunit, non-ATPase, 6 | KIAA0107\|Rpn7\|S10\|SGA-113M\|p44S10 | 3 | Antigen Processing and Presentation |
| PSME3 | 10197 | proteasome (prosome, macropain) activator subunit 3 (PA28 gamma; Ki) | Ki\|PA28-gamma\|PA28G\|REG-GAMMA | 17 | Antigen Processing and Presentation |
| PSMD14 | 10213 | proteasome (prosome, macropain) 26S subunit, non-ATPase, 14 | PAD1\|POH1\|rpn11 | 2 | Antigen Processing and Presentation |
| CLEC4M | 10332 | C-type lectin domain family 4, member M | CD209L\|CD299\|DC-SIGN2\|DC-SIGNR\|DCSIGNR\|HP10347\|L-SIGN\|LSIGN\|MGC129964\|MGC47866 | 19 | Antigen Processing and Presentation |
| IFI30 | 10437 | interferon, gamma-inducible protein 30 | GILT\|IFI-30\|IP30\|MGC32056 | 19 | Antigen Processing and Presentation |
| PROCR | 10544 | protein C receptor, endothelial (EPCR) | CCCA\|CCD41\|CD201\|EPCR\|MGC23024\|bA42O4.2 | 20 | Antigen Processing and Presentation |
| ADRM1 | 11047 | adhesion regulating molecule 1 | ARM1\|GP110\|MGC29536\|Rpn13 | 20 | Antigen Processing and Presentation |
| KIAA0368 | 23392 | KIAA0368 | ECM29\|FLJ22036\|KIAA1962\|RP11-386D8.2 | 9 | Antigen Processing and Presentation |
| TRPC4AP | 26133 | transient receptor potential cation channel, subfamily C, member 4 associated protein | C20orf188\|TRRP4AP\|TRUSS | 20 | Antigen Processing and Presentation |
| CD209 | 30835 | CD209 molecule | CDSIGN\|CLEC4L\|DC-SIGN\|DC-SIGN1\|MGC129965 | 19 | Antigen Processing and Presentation |
| UBXN1 | 51035 | UBX domain protein 1 | 2B28\|UBXD10 | 11 | Antigen Processing and Presentation |
| ERAP1 | 51752 | endoplasmic reticulum aminopeptidase 1 | A-LAP\|ALAP\|APPILS\|ARTS-1\|ARTS1\|ERAAP\|ERAAP1\|KIAA0525\|PILS-AP\|PILSAP | 5 | Antigen Processing and Presentation |
| TAPBPL | 55080 | TAP binding protein-like | FLJ10143\|TAPBP-R\|TAPBPR | 12 | Antigen Processing and Presentation |
| KIR2DL5A | 57292 | killer cell immunoglobulin-like receptor, two domains, long cytoplasmic tail, 5A | CD158F\|KIR2DL5\|KIR2DL5.1\|KIR2DL5.3 | 19 | Antigen Processing and Presentation |
| ERAP2 | 64167 | endoplasmic reticulum aminopeptidase 2 | FLJ23633\|FLJ23701\|FLJ23807\|L-RAP\|LRAP | 5 | Antigen Processing and Presentation |
| ULBP3 | 79465 | UL16 binding protein 3 | RAET1N | 6 | Antigen Processing and Presentation |
| ULBP2 | 80328 | UL16 binding protein 2 | N2DL2\|RAET1H | 6 | Antigen Processing and Presentation |
| ULBP1 | 80329 | UL16 binding protein 1 | RAET1I | 6 | Antigen Processing and Presentation |
| KIR3DL3 | 115653 | killer cell immunoglobulin-like receptor, three domains, long cytoplasmic tail, 3 | CD158Z\|KIR3DL7\|KIR44\|KIRC1 | 19 | Antigen Processing and Presentation |
| RAET1E | 135250 | retinoic acid early transcript 1E | LETAL\|MGC125308\|MGC125309\|RAET1E2\|ULBP4\|bA350J20.7 | 6 | Antigen Processing and Presentation |
| RAET1L | 154064 | retinoic acid early transcript 1L | - | 6 | Antigen Processing and Presentation |
| UBR1 | 197131 | ubiquitin protein ligase E3 component n-recognin 1 | JBS\|MGC142065\|MGC142067 | 15 | Antigen Processing and Presentation |
| RAET1G | 353091 | retinoic acid early transcript 1G | ULBP5 | 6 | Antigen Processing and Presentation |
| PDIA2 | 64714 | protein disulfide isomerase family A, member 2 | PDA2\|PDI\|PDIP\|PDIR | 16 | Antigen Processing and Presentation |
| HAMP | 57817 | hepcidin antimicrobial peptide | HEPC\|HEPCIDIN\|HFE2B\|LEAP-1\|LEAP1\|PLTR | 19 | Antimicrobials |
| PI3 | 5266 | peptidase inhibitor 3, skin-derived | ESI\|MGC13613\|SKALP\|WAP3\|WFDC14\|cementoin | 20 | Antimicrobials |
| CAMP | 820 | cathelicidin antimicrobial peptide | CAP18\|CRAMP\|FALL-39\|FALL39\|HSD26\|LL37 | 3 | Antimicrobials |
| DEFB4 | 1673 | defensin, beta 4 | DEFB-2\|DEFB102\|DEFB2\|HBD-2\|SAP1 | 8 | Antimicrobials |
| PPBP | 5473 | pro-platelet basic protein (chemokine (C-X-C motif) ligand 7) | B-TG1\|Beta-TG\|CTAP-III\|CTAP3\|CTAPIII\|CXCL7\|LA-PF4\|LDGF\|MDGF\|NAP-2\|PBP\|SCYB7\|TC1\|TC2\|TGB\|TGB1\|THBGB\|THBGB1 | 4 | Antimicrobials |
| REG3G | 130120 | regenerating islet-derived 3 gamma | MGC118998\|MGC118999\|MGC119001\|PAP1B\|PAPIB\|REG-III\|UNQ429 | 2 | Antimicrobials |
| CXCL14 | 9547 | chemokine (C-X-C motif) ligand 14 | BMAC\|BRAK\|KS1\|Kec\|MGC10687\|MIP-2g\|NJAC\|SCYB14\|bolekine | 5 | Antimicrobials |
| CXCL16 | 58191 | chemokine (C-X-C motif) ligand 16 | CXCLG16\|SR-PSOX\|SRPSOX | 17 | Antimicrobials |
| SLPI | 6590 | secretory leukocyte peptidase inhibitor | ALK1\|ALP\|BLPI\|HUSI\|HUSI-I\|MPI\|WAP4\|WFDC4 | 20 | Antimicrobials |
| IL8 | 3576 | interleukin 8 | CXCL8\|GCP-1\|GCP1\|LECT\|LUCT\|LYNAP\|MDNCF\|MONAP\|NAF\|NAP-1\|NAP1 | 4 | Antimicrobials |
| CXCL10 | 3627 | chemokine (C-X-C motif) ligand 10 | C7\|IFI10\|INP10\|IP-10\|SCYB10\|crg-2\|gIP-10\|mob-1 | 4 | Antimicrobials |
| CXCL9 | 4283 | chemokine (C-X-C motif) ligand 9 | CMK\|Humig\|MIG\|SCYB9\|crg-10 | 4 | Antimicrobials |
| CXCL5 | 6374 | chemokine (C-X-C motif) ligand 5 | ENA-78\|SCYB5 | 4 | Antimicrobials |
| CXCL11 | 6373 | chemokine (C-X-C motif) ligand 11 | H174\|I-TAC\|IP-9\|IP9\|MGC102770\|SCYB11\|SCYB9B\|b-R1 | 4 | Antimicrobials |
| CXCL6 | 6372 | chemokine (C-X-C motif) ligand 6 (granulocyte chemotactic protein 2) | CKA-3\|GCP-2\|GCP2\|SCYB6 | 4 | Antimicrobials |
| CXCL1 | 2919 | chemokine (C-X-C motif) ligand 1 (melanoma growth stimulating activity, alpha) | FSP\|GRO1\|GROa\|MGSA\|MGSA-a\|NAP-3\|SCYB1 | 4 | Antimicrobials |
| CXCL12 | 6387 | chemokine (C-X-C motif) ligand 12 (stromal cell-derived factor 1) | PBSF\|SCYB12\|SDF-1a\|SDF-1b\|SDF1\|SDF1A\|SDF1B\|TLSF-a\|TLSF-b\|TPAR1 | 10 | Antimicrobials |
| CXCL13 | 10563 | chemokine (C-X-C motif) ligand 13 | ANGIE\|ANGIE2\|BCA-1\|BCA1\|BLC\|BLR1L\|SCYB13 | 4 | Antimicrobials |
| CXCL2 | 2920 | chemokine (C-X-C motif) ligand 2 | CINC-2a\|GRO2\|GROb\|MGSA-b\|MIP-2a\|MIP2\|MIP2A\|SCYB2 | 4 | Antimicrobials |
| PF4 | 5196 | platelet factor 4 | CXCL4\|MGC138298\|SCYB4 | 4 | Antimicrobials |
| XCL1 | 6375 | chemokine (C motif) ligand 1 | ATAC\|LPTN\|LTN\|SCM-1\|SCM-1a\|SCM1\|SCYC1 | 1 | Antimicrobials |
| CXCL3 | 2921 | chemokine (C-X-C motif) ligand 3 | CINC-2b\|GRO3\|GROg\|MIP-2b\|MIP2B\|SCYB3 | 4 | Antimicrobials |
| DEFB103A | 55894 | defensin, beta 103A | DEFB103\|DEFB3\|HBD-3\|HBD3\|HBP-3\|HBP3 | 8 | Antimicrobials |
| CCL13 | 6357 | chemokine (C-C motif) ligand 13 | CKb10\|MCP-4\|MGC17134\|NCC-1\|NCC1\|SCYA13\|SCYL1 | 17 | Antimicrobials |
| CCL1 | 6346 | chemokine (C-C motif) ligand 1 | I-309\|P500\|SCYA1\|SISe\|TCA3 | 17 | Antimicrobials |
| DEFB1 | 1672 | defensin, beta 1 | BD1\|DEFB-1\|DEFB101\|HBD1\|MGC51822 | 8 | Antimicrobials |
| CCL8 | 6355 | chemokine (C-C motif) ligand 8 | HC14\|MCP-2\|MCP2\|SCYA10\|SCYA8 | 17 | Antimicrobials |
| ELANE | 1991 | elastase, neutrophil expressed | ELA2\|GE\|HLE\|HNE\|NE\|PMN-E | 19 | Antimicrobials |
| DEFB103B | 414325 | defensin, beta 103B | - | 8 | Antimicrobials |
| DEFA3 | 1668 | defensin, alpha 3, neutrophil-specific | DEF3\|HNP-3\|HNP3\|HP-3 | 8 | Antimicrobials |
| DEFA1 | 1667 | defensin, alpha 1 | DEF1\|DEFA2\|HNP-1\|HP-1\|MGC138393\|MRS | 8 | Antimicrobials |
| TMSB10 | 9168 | thymosin beta 10 | MIG12\|TB10 | 2 | Antimicrobials |
| DEFA6 | 1671 | defensin, alpha 6, Paneth cell-specific | DEF6\|HD-6 | 8 | Antimicrobials |
| DEFA5 | 1670 | defensin, alpha 5, Paneth cell-specific | DEF5\|HD-5\|MGC129728 | 8 | Antimicrobials |
| DEFA4 | 1669 | defensin, alpha 4, corticostatin | DEF4\|HNP-4\|HP-4\|HP4\|MGC120099\|MGC138296 | 8 | Antimicrobials |
| LCN2 | 3934 | lipocalin 2 | 24p3\|NGAL | 9 | Antimicrobials |
| LCN1 | 3933 | lipocalin 1 (tear prealbumin) | MGC71975\|PMFA\|TP\|VEGP | 9 | Antimicrobials |
| COLEC10 | 10584 | collectin sub-family member 10 (C-type lectin) | CLL1\|MGC118794\|MGC118795 | 8 | Antimicrobials |
| BPI | 671 | bactericidal/permeability-increasing protein | - | 20 | Antimicrobials |
| S100A9 | 6280 | S100 calcium binding protein A9 | 60B8AG\|CAGB\|CFAG\|CGLB\|L1AG\|LIAG\|MAC387\|MIF\|MRP14\|NIF\|P14 | 1 | Antimicrobials |
| S100A8 | 6279 | S100 calcium binding protein A8 | 60B8AG\|CAGA\|CFAG\|CGLA\|CP-10\|L1Ag\|MA387\|MIF\|MRP8\|NIF\|P8 | 1 | Antimicrobials |
| DCD | 117159 | dermcidin | AIDD\|DCD-1\|DSEP\|HCAP\|MGC71930\|PIF | 12 | Antimicrobials |
| LCN6 | 158062 | lipocalin 6 | LCN5\|UNQ643\|hLcn5 | 9 | Antimicrobials |
| S100A12 | 6283 | S100 calcium binding protein A12 | CAAF1\|CAGC\|CGRP\|ENRAGE\|MRP6\|p6 | 1 | Antimicrobials |
| HTN3 | 3347 | histatin 3 | HIS2\|HTN2\|HTN5 | 4 | Antimicrobials |
| LCN8 | 138307 | lipocalin 8 | EP17\|LCN5 | 9 | Antimicrobials |
| LOC728358 | 728358 | defensin, alpha 1 | - | 8 | Antimicrobials |
| CCR10 | 2826 | chemokine (C-C motif) receptor 10 | GPR2 | 17 | Antimicrobials |
| CELA1 | 1990 | chymotrypsin-like elastase family, member 1 | ELA1 | 12 | Antimicrobials |
| DEFB106A | 245909 | defensin, beta 106A | BD-6\|DEFB-6\|DEFB106\|MGC118938\|MGC118939\|MGC118940\|MGC118941\|MGC133011\|MGC133012 | 8 | Antimicrobials |
| PENK | 5179 | proenkephalin | - | 8 | Antimicrobials |
| BPIL2 | 254240 | bactericidal/permeability-increasing protein-like 2 | - | 22 | Antimicrobials |
| MMP12 | 4321 | matrix metallopeptidase 12 (macrophage elastase) | HME\|MGC138506\|MME | 11 | Antimicrobials |
| BPIL3 | 128859 | bactericidal/permeability-increasing protein-like 3 | LPLUNC6 | 20 | Antimicrobials |
| LEAP2 | 116842 | liver expressed antimicrobial peptide 2 | LEAP-2 | 5 | Antimicrobials |
| SFTPD | 6441 | surfactant protein D | COLEC7\|PSP-D\|SFTP4\|SP-D | 10 | Antimicrobials |
| LCN9 | 392399 | lipocalin 9 | 9230102I19Rik | 9 | Antimicrobials |
| BPIL1 | 80341 | bactericidal/permeability-increasing protein-like 1 | C20orf184\|LPLUNC2\|RYSR\|dJ726C3.2 | 20 | Antimicrobials |
| PTGDS | 5730 | prostaglandin D2 synthase 21kDa (brain) | LPGDS\|PDS\|PGD2\|PGDS\|PGDS2 | 9 | Antimicrobials |
| TMSB4X | 7114 | thymosin beta 4, X-linked | FX\|PTMB4\|TB4X\|TMSB4 | X | Antimicrobials |
| PGLYRP1 | 8993 | peptidoglycan recognition protein 1 | MGC126894\|MGC126896\|PGLYRP\|PGRP\|PGRP-S\|PGRPS\|TAG7\|TNFSF3L | 19 | Antimicrobials |
| ZC3HAV1 | 56829 | zinc finger CCCH-type, antiviral 1 | DKFZp686F2052\|DKFZp686H1869\|DKFZp686O19171\|FLB6421\|FLJ13288\|MGC48898\|ZAP\|ZC3H2\|ZC3HDC2 | 7 | Antimicrobials |
| TMSB15A | 11013 | thymosin beta 15a | TMSB15\|TMSL8\|TMSNB\|Tb15\|TbNB | X | Antimicrobials |
| S100B | 6285 | S100 calcium binding protein B | NEF\|S100\|S100beta | 21 | Antimicrobials |
| S100A13 | 6284 | S100 calcium binding protein A13 | - | 1 | Antimicrobials |
| S100A6 | 6277 | S100 calcium binding protein A6 | 2A9\|5B10\|CABP\|CACY\|PRA | 1 | Antimicrobials |
| DEFB119 | 245932 | defensin, beta 119 | DEFB-19\|DEFB-20\|DEFB120\|ESC42-RELA\|ESC42-RELB\|MGC71893 | 20 | Antimicrobials |
| DEFB107A | 245910 | defensin, beta 107A | BD-7\|DEFB-7\|DEFB107 | 8 | Antimicrobials |
| DEFB105A | 245908 | defensin, beta 105A | BD-5\|DEFB-5\|DEFB105 | 8 | Antimicrobials |
| SERPIND1 | 3053 | serpin peptidase inhibitor, clade D (heparin cofactor), member 1 | D22S673\|HC2\|HCF2\|HCII\|HLS2\|LS2 | 22 | Antimicrobials |
| DEFB129 | 140881 | defensin, beta 129 | C20orf87\|DEFB-29\|DEFB29\|bA530N10.3\|hBD-29 | 20 | Antimicrobials |
| DEFB127 | 140850 | defensin, beta 127 | C20orf73\|DEF-27\|DEFB-27\|DEFB27\|bA530N10.2\|hBD-27 | 20 | Antimicrobials |
| S100P | 6286 | S100 calcium binding protein P | MIG9 | 4 | Antimicrobials |
| S100A7 | 6278 | S100 calcium binding protein A7 | PSOR1\|S100A7c | 1 | Antimicrobials |
| DEFB104A | 140596 | defensin, beta 104A | BD-4\|DEFB-4\|DEFB104\|DEFB4\|MGC118942\|MGC118944\|MGC118945\|hBD-4 | 8 | Antimicrobials |
| DEFB126 | 81623 | defensin, beta 126 | C20orf8\|DEFB-26\|DEFB26\|ESP13.2\|bA530N10.1\|hBD-26 | 20 | Antimicrobials |
| DEFB106B | 503841 | defensin, beta 106B | - | 8 | Antimicrobials |
| DEFB104B | 503618 | defensin, beta 104B | - | 8 | Antimicrobials |
| DEFB107B | 503614 | defensin, beta 107B | HsT21816 | 8 | Antimicrobials |
| PGLYRP3 | 114771 | peptidoglycan recognition protein 3 | MGC149197\|PGRP-Ialpha\|PGRPIA | 1 | Antimicrobials |
| PGLYRP2 | 114770 | peptidoglycan recognition protein 2 | HMFT0141\|PGLYRPL\|PGRP-L\|PGRPL\|TAGL-like\|tagL\|tagL-alpha\|tagl-beta | 19 | Antimicrobials |
| S100A10 | 6281 | S100 calcium binding protein A10 | 42C\|ANX2L\|ANX2LG\|CAL1L\|CLP11\|Ca[1]\|GP11\|MGC111133\|P11\|p10 | 1 | Antimicrobials |
| S100A2 | 6273 | S100 calcium binding protein A2 | CAN19\|MGC111539\|S100L | 1 | Antimicrobials |
| DEFB125 | 245938 | defensin, beta 125 | DEFB-25\|MGC57449 | 20 | Antimicrobials |
| DEFB123 | 245936 | defensin, beta 123 | DEFB-23\|ESC42-RELD | 20 | Antimicrobials |
| DEFB105B | 504180 | defensin, beta 105B | - | 8 | Antimicrobials |
| DEFB132 | 400830 | defensin, beta 132 | DEFB32\|UNQ827 | 20 | Antimicrobials |
| C20orf185 | 359710 | chromosome 20 open reading frame 185 | LPLUNC3\|RYA3\|dJ726C3.4 | 20 | Antimicrobials |
| LCN12 | 286256 | lipocalin 12 | MGC34753\|MGC48935 | 9 | Antimicrobials |
| PGLYRP4 | 57115 | peptidoglycan recognition protein 4 | PGLYRPIbeta\|PGRP-Ibeta\|PGRPIB\|SBBI67 | 1 | Antimicrobials |
| S100A11 | 6282 | S100 calcium binding protein A11 | MLN70\|S100C | 1 | Antimicrobials |
| S100A5 | 6276 | S100 calcium binding protein A5 | S100D | 1 | Antimicrobials |
| S100A3 | 6274 | S100 calcium binding protein A3 | S100E | 1 | Antimicrobials |
| S100A1 | 6271 | S100 calcium binding protein A1 | S100\|S100-alpha\|S100A | 1 | Antimicrobials |
| DEFB128 | 245939 | defensin, beta 128 | DEFB-28\|DEFB28\|hBD-28 | 20 | Antimicrobials |
| DEFB108B | 245911 | defensin, beta 108B | - | 11 | Antimicrobials |
| HTN1 | 3346 | histatin 1 | HIS1 | 4 | Antimicrobials |
| LMBR1L | 55716 | limb region 1 homolog (mouse)-like | FLJ10494\|FLJ36251\|KIAA1174\|LIMR | 12 | Antimicrobials |
| S100A7A | 338324 | S100 calcium binding protein A7A | NICE-2\|S100A15\|S100A7L1\|S100A7f | 1 | Antimicrobials |
| DEFB118 | 117285 | defensin, beta 118 | C20orf63\|DEFB-18\|ESC42\|dJ1018D12.3 | 20 | Antimicrobials |
| COLEC12 | 81035 | collectin sub-family member 12 | CLP1\|NSR2\|SCARA4\|SRCL | 18 | Antimicrobials |
| TMSB4Y | 9087 | thymosin beta 4, Y-linked | MGC26307\|TB4Y | Y | Antimicrobials |
| DEFB131 | 644414 | defensin, beta 131 | DEFB-31 | 4 | Antimicrobials |
| DEFB134 | 613211 | defensin, beta 134 | MGC163333\|MGC163335 | 8 | Antimicrobials |
| DEFB130 | 245940 | defensin, beta 130 | DEFB-30 | 8 | Antimicrobials |
| DEFB124 | 245937 | defensin, beta 124 | DEFB-24 | 20 | Antimicrobials |
| DEFB121 | 245934 | defensin, beta 121 | DEFB-21\|ESC42-RELC | 20 | Antimicrobials |
| DEFB116 | 245930 | defensin, beta 116 | DEFB-16 | 20 | Antimicrobials |
| DEFB115 | 245929 | defensin, beta 115 | DEFB-15 | 20 | Antimicrobials |
| DEFB114 | 245928 | defensin, beta 114 | DEFB-14 | 6 | Antimicrobials |
| DEFB113 | 245927 | defensin, beta 113 | DEFB-13 | 6 | Antimicrobials |
| DEFB112 | 245915 | defensin, beta 112 | DEFB-12 | 6 | Antimicrobials |
| DEFB110 | 245913 | defensin, beta 110 | DEFB-10 | 6 | Antimicrobials |
| TMSB15B | 286527 | thymosin beta 15B | MGC39900\|Tbeta15b | X | Antimicrobials |
| DEFB133 | 403339 | defensin, beta 133 | - | 12 | Antimicrobials |
| S100Z | 170591 | S100 calcium binding protein Z | Gm625\|S100-zeta | 5 | Antimicrobials |
| MAVS | 57506 | mitochondrial antiviral signaling protein | CARDIF\|DKFZp547C224\|DKFZp666M015\|FLJ27482\|FLJ35386\|FLJ38051\|FLJ41962\|IPS-1\|IPS1\|KIAA1271\|MGC3260\|VISA | 20 | Antimicrobials |
| TMSL3 | 7117 | thymosin-like 3 | - | 4 | Antimicrobials |
| S100A14 | 57402 | S100 calcium binding protein A14 | BCMP84\|S100A15 | 1 | Antimicrobials |
| LCN10 | 414332 | lipocalin 10 | - | 9 | Antimicrobials |
| S100A16 | 140576 | S100 calcium binding protein A16 | AAG13\|DT1P1A7\|MGC17528\|S100F | 1 | Antimicrobials |
| DEFB137 | 613210 | beta-defensin 137 | DEFB136 | 8 | Antimicrobials |
| DEFB136 | 613209 | beta-defensin 136 | DEFB135 | 8 | Antimicrobials |
| DEFB117 | 245931 | defensin, beta 117 | DEFB-17 | 20 | Antimicrobials |
| DEFB111 | 245914 | defensin, beta 111 | DEFB-11 | 6 | Antimicrobials |
| ZC3HAV1L | 92092 | zinc finger CCCH-type, antiviral 1-like | C7orf39\|MGC14289 | 7 | Antimicrobials |
| S100A7L2 | 645922 | S100 calcium binding protein A7-like 2 | S100a7b | 1 | Antimicrobials |
| LOC731414 | 731414 | similar to S100 calcium-binding protein A10 | - | 17 | Antimicrobials |
| LOC730963 | 730963 | similar to Neutrophil defensin 4 precursor (HNP-4) (HP-4) (Defensin, alpha 4) | - | 8 | Antimicrobials |
| COLEC2 | 50639 | collectin sub-family member 2 | MBL | - | Antimicrobials |
| DEFB4P | 728454 | defensin, beta 4, pseudogene | - | 8 | Antimicrobials |
| C20orf186 | 149954 | chromosome 20 open reading frame 186 | LPLUNC4\|RY2G5\|dJ726C3.5 | 20 | Antimicrobials |
| IFNAR1 | 3454 | interferon (alpha, beta and omega) receptor 1 | AVP\|IFN-alpha-REC\|IFNAR\|IFNBR\|IFRC | 21 | Antimicrobials |
| AZU1 | 566 | azurocidin 1 | AZAMP\|AZU\|CAP37\|HBP\|HUMAZUR\|NAZC | 19 | Antimicrobials |
| LOC729523 | 729523 | similar to beta-defensin 130 | - | 11 | Antimicrobials |
| LOC100130154 | 100130154 | similar to thymosin, beta 10 | - | 2 | Antimicrobials |
| LOC100134379 | 100134379 | similar to bactericidal/permeability-increasing protein | - | Un | Antimicrobials |
| LOC100134289 | 100134289 | similar to bactericidal permeability increasing protein (BPI) | - | 20 | Antimicrobials |
| LOC100129216 | 100129216 | similar to beta-defensin 131 | - | 11 | Antimicrobials |
| DEFA1A3 | 613253 | defensin, alpha 1 and alpha 3, variable copy number locus | DEFA1\|DEFA3\|DEFT1P | 8 | Antimicrobials |
| LOC100131433 | 100131433 | similar to thymosin, beta 10 | - | 9 | Antimicrobials |
| LCN1L1 | 286310 | lipocalin 1-like 1 | bA430N14.2 | 9 | Antimicrobials |
| S100G | 795 | S100 calcium binding protein G | CABP1\|CABP9K\|CALB3\|MGC138379 | X | Antimicrobials |
| LOC648637 | 648637 | similar to Neutrophil defensin 4 precursor (HNP-4) (HP-4) (Defensin, alpha 4) | - | 8 | Antimicrobials |
| LOC100130969 | 100130969 | similar to lipocalin-like protein | - | 9 | Antimicrobials |
| LOC100133267 | 100133267 | similar to beta-defensin 130 | - | 8 | Antimicrobials |
| LOC100133128 | 100133128 | Beta-defensin 108B-like | - | 4 | Antimicrobials |
| LOC100128174 | 100128174 | similar to beta-defensin 131 | - | 8 | Antimicrobials |
| TCHHL1 | 126637 | trichohyalin-like 1 | S100A17\|THHL1\|basalin | 1 | Antimicrobials |
| TINAGL1 | 64129 | tubulointerstitial nephritis antigen-like 1 | ARG1\|LCN7\|LIECG3\|TINAGRP | 1 | Antimicrobials |
| IFNGR1 | 3459 | interferon gamma receptor 1 | CD119\|FLJ45734\|IFNGR | 6 | Antimicrobials |
| SLC22A17 | 51310 | solute carrier family 22, member 17 | BOCT\|BOIT\|NGALR\|hBOIT | 14 | Antimicrobials |
| WFIKKN1 | 117166 | WAP, follistatin/kazal, immunoglobulin, kunitz and netrin domain containing 1 | C16orf12\|MGC126651\|MGC126655\|RJD2\|WFIKKN | 16 | Antimicrobials |
| WFDC2 | 10406 | WAP four-disulfide core domain 2 | HE4\|MGC57529\|WAP5\|dJ461P17.6 | 20 | Antimicrobials |
| IL6 | 3569 | interleukin 6 (interferon, beta 2) | BSF2\|HGF\|HSF\|IFNB2\|IL-6 | 7 | Antimicrobials |
| UMODL1 | 89766 | uromodulin-like 1 | - | 21 | Antimicrobials |
| TGFB1 | 7040 | transforming growth factor, beta 1 | CED\|DPD1\|TGFB\|TGFbeta | 19 | Antimicrobials |
| PF4V1 | 5197 | platelet factor 4 variant 1 | CXCL4L1\|CXCL4V1\|PF4-ALT\|PF4A\|SCYB4V1 | 4 | Antimicrobials |
| MMP9 | 4318 | matrix metallopeptidase 9 (gelatinase B, 92kDa gelatinase, 92kDa type IV collagenase) | CLG4B\|GELB\|MMP-9 | 20 | Antimicrobials |
| KAL1 | 3730 | Kallmann syndrome 1 sequence | ADMLX\|HHA\|KAL\|KALIG-1\|KMS | X | Antimicrobials |
| TLR4 | 7099 | toll-like receptor 4 | ARMD10\|CD284\|TOLL\|hToll | 9 | Antimicrobials |
| IFNG | 3458 | interferon, gamma | IFG\|IFI | 12 | Antimicrobials |
| SPAG11B | 10407 | sperm associated antigen 11B | EP2\|EP2C\|EP2D\|HE2\|HE2C\|MGC61846\|SPAG11 | 8 | Antimicrobials |
| A2M | 2 | alpha-2-macroglobulin | CPAMD5\|DKFZp779B086\|FWP007\|S863-7 | 12 | Antimicrobials |
| CTSL1 | 1514 | cathepsin L1 | CATL\|CTSL\|FLJ31037\|MEP | 9 | Antimicrobials |
| NFKB1 | 4790 | nuclear factor of kappa light polypeptide gene enhancer in B-cells 1 | DKFZp686C01211\|EBP-1\|KBF1\|MGC54151\|NF-kappa-B\|NFKB-p105\|NFKB-p50\|p105\|p50 | 4 | Antimicrobials |
| APOBEC3G | 60489 | apolipoprotein B mRNA editing enzyme, catalytic polypeptide-like 3G | ARP9\|CEM15\|FLJ12740\|MDS019\|bK150C2.7\|dJ494G10.1 | 22 | Antimicrobials |
| FABP6 | 2172 | fatty acid binding protein 6, ileal | I-15P\|I-BABP\|I-BALB\|I-BAP\|ILBP\|ILBP3\|ILLBP | 5 | Antimicrobials |
| NOD2 | 64127 | nucleotide-binding oligomerization domain containing 2 | ACUG\|BLAU\|CARD15\|CD\|CLR16.3\|IBD1\|NLRC2\|NOD2B\|PSORAS1 | 16 | Antimicrobials |
| MBL2 | 4153 | mannose-binding lectin (protein C) 2, soluble (opsonic defect) | COLEC1\|HSMBPC\|MBL\|MBP\|MBP1\|MGC116832\|MGC116833 | 10 | Antimicrobials |
| SFTPA1B | 6435 | surfactant protein A1B | AC068139.6\|MGC133365\|PSAP\|PSPA\|SFTP1\|SFTPA1 | 10 | Antimicrobials |
| RBP1 | 5947 | retinol binding protein 1, cellular | CRABP-I\|CRBP\|CRBP1\|CRBPI\|RBPC | 3 | Antimicrobials |
| TLR2 | 7097 | toll-like receptor 2 | CD282\|TIL4 | 4 | Antimicrobials |
| SLC40A1 | 30061 | solute carrier family 40 (iron-regulated transporter), member 1 | FPN1\|HFE4\|IREG1\|MST079\|MSTP079\|MTP1\|SLC11A3 | 2 | Antimicrobials |
| PLAU | 5328 | plasminogen activator, urokinase | ATF\|UPA\|URK\|u-PA | 10 | Antimicrobials |
| IL1B | 3553 | interleukin 1, beta | IL-1\|IL1-BETA\|IL1F2 | 2 | Antimicrobials |
| PAEP | 5047 | progestagen-associated endometrial protein | GD\|GdA\|GdF\|GdS\|MGC138509\|MGC142288\|PAEG\|PEP\|PP14 | 9 | Antimicrobials |
| HFE2 | 148738 | hemochromatosis type 2 (juvenile) | HFE2A\|HJV\|JH\|MGC23953\|RGMC | 1 | Antimicrobials |
| MUC5AC | 4586 | mucin 5AC, oligomeric mucus/gel-forming | MUC5 | 11 | Antimicrobials |
| CTSS | 1520 | cathepsin S | MGC3886 | 1 | Antimicrobials |
| OBP2A | 29991 | odorant binding protein 2A | OBP\|OBP2C\|OBPIIa\|hOBPIIa | 9 | Antimicrobials |
| PLTP | 5360 | phospholipid transfer protein | HDLCQ9 | 20 | Antimicrobials |
| MX1 | 4599 | myxovirus (influenza virus) resistance 1, interferon-inducible protein p78 (mouse) | IFI-78K\|IFI78\|MX\|MxA | 21 | Antimicrobials |
| DDX58 | 23586 | DEAD (Asp-Glu-Ala-Asp) box polypeptide 58 | DKFZp434J1111\|DKFZp686N19181\|FLJ13599\|RIG-I | 9 | Antimicrobials |
| IL29 | 282618 | interleukin 29 (interferon, lambda 1) | IFNL1\|IL-29 | 19 | Antimicrobials |
| IRF3 | 3661 | interferon regulatory factor 3 | - | 19 | Antimicrobials |
| SFTPA2 | 729238 | surfactant protein A2 | COLEC5\|MGC189761\|SP-A2\|SPA2\|SPAII | 10 | Antimicrobials |
| SFTPA2B | 6436 | surfactant protein A2B | AC068139.3\|SFTPA2\|SP-2A\|SP-A1\|SP-A2\|SPAII | 10 | Antimicrobials |
| LPA | 4018 | lipoprotein, Lp(a) | AK38\|APOA\|LP | 6 | Antimicrobials |
| LBP | 3929 | lipopolysaccharide binding protein | MGC22233 | 20 | Antimicrobials |
| RBP4 | 5950 | retinol binding protein 4, plasma | - | 10 | Antimicrobials |
| SFTPA1 | 653509 | surfactant protein A1 | COLEC4\|FLJ51913\|SFTP1\|SP-A\|SP-A1 | 10 | Antimicrobials |
| NOX4 | 50507 | NADPH oxidase 4 | KOX\|KOX-1\|RENOX | 11 | Antimicrobials |
| LTF | 4057 | lactotransferrin | GIG12\|HLF2\|LF | 3 | Antimicrobials |
| IFNB1 | 3456 | interferon, beta 1, fibroblast | IFB\|IFF\|IFNB\|MGC96956 | 9 | Antimicrobials |
| RBP5 | 83758 | retinol binding protein 5, cellular | CRBP-III\|CRBP3\|CRBPIII | 12 | Antimicrobials |
| FABP7 | 2173 | fatty acid binding protein 7, brain | B-FABP\|BLBP\|DKFZp547J2313\|FABPB\|MRG | 6 | Antimicrobials |
| FABP5 | 2171 | fatty acid binding protein 5 (psoriasis-associated) | E-FABP\|EFABP\|PA-FABP\|PAFABP | 8 | Antimicrobials |
| FABP3 | 2170 | fatty acid binding protein 3, muscle and heart (mammary-derived growth inhibitor) | FABP11\|H-FABP\|MDGI\|O-FABP | 1 | Antimicrobials |
| FABP2 | 2169 | fatty acid binding protein 2, intestinal | FABPI\|I-FABP\|MGC133132 | 4 | Antimicrobials |
| FABP4 | 2167 | fatty acid binding protein 4, adipocyte | A-FABP\|aP2 | 8 | Antimicrobials |
| R3HDML | 140902 | R3H domain containing-like | MGC129564\|dJ881L22.3 | 20 | Antimicrobials |
| C20orf71 | 128861 | chromosome 20 open reading frame 71 | MGC44525\|SPLUNC3 | 20 | Antimicrobials |
| C20orf114 | 92747 | chromosome 20 open reading frame 114 | LPLUNC1\|MGC14597 | 20 | Antimicrobials |
| OASL | 8638 | 2'-5'-oligoadenylate synthetase-like | TRIP14\|p59OASL | 12 | Antimicrobials |
| CRABP2 | 1382 | cellular retinoic acid binding protein 2 | CRABP-II\|RBP6 | 1 | Antimicrobials |
| CRABP1 | 1381 | cellular retinoic acid binding protein 1 | CRABP\|CRABP-I\|CRABPI\|RBP5 | 15 | Antimicrobials |
| RBP7 | 116362 | retinol binding protein 7, cellular | CRBP4\|CRBPIV\|MGC70641 | 1 | Antimicrobials |
| DUOX1 | 53905 | dual oxidase 1 | LNOX1\|MGC138840\|MGC138841\|NOXEF1\|THOX1 | 15 | Antimicrobials |
| OBP2B | 29989 | odorant binding protein 2B | MGC119022\|hOBPIIb | 9 | Antimicrobials |
| RBP2 | 5948 | retinol binding protein 2, cellular | CRABP-II\|CRBP2\|CRBPII\|RBPC2 | 3 | Antimicrobials |
| LCN15 | 389812 | lipocalin 15 | PRO6093\|UNQ2541 | 9 | Antimicrobials |
| CETP | 1071 | cholesteryl ester transfer protein, plasma | HDLCQ10 | 16 | Antimicrobials |
| FABP12 | 646486 | fatty acid binding protein 12 | - | 8 | Antimicrobials |
| FABP9 | 646480 | fatty acid binding protein 9, testis | PERF\|PERF15\|T-FABP | 8 | Antimicrobials |
| PLUNC | 51297 | palate, lung and nasal epithelium associated | LPLUNC3\|LUNX\|NASG\|SPLUNC1\|SPURT\|bA49G10.5 | 20 | Antimicrobials |
| LCNL1 | 401562 | lipocalin-like 1 | FLJ45224 | 9 | Antimicrobials |
| C8G | 733 | complement component 8, gamma polypeptide | C8C\|MGC142186 | 9 | Antimicrobials |
| SPAG11A | 653423 | sperm associated antigen 11A | HE2 | 8 | Antimicrobials |
| PI15 | 51050 | peptidase inhibitor 15 | CRISP8\|DKFZp686F0366\|P24TI\|P25TI | 8 | Antimicrobials |
| NOX1 | 27035 | NADPH oxidase 1 | GP91-2\|MOX1\|NOH-1\|NOH1 | X | Antimicrobials |
| PMP2 | 5375 | peripheral myelin protein 2 | FABP8\|M-FABP\|MP2\|P2 | 8 | Antimicrobials |
| APOD | 347 | apolipoprotein D | - | 3 | Antimicrobials |
| ORM2 | 5005 | orosomucoid 2 | AGP-B\|AGP-B'\|AGP2 | 9 | Antimicrobials |
| ORM1 | 5004 | orosomucoid 1 | AGP-A\|AGP1\|ORM | 9 | Antimicrobials |
| TNF | 7124 | tumor necrosis factor (TNF superfamily, member 2) | DIF\|TNF-alpha\|TNFA\|TNFSF2 | 6 | Antimicrobials |
| CTSG | 1511 | cathepsin G | CG\|MGC23078 | 14 | Antimicrobials |
| PRTN3 | 5657 | proteinase 3 | ACPA\|AGP7\|C-ANCA\|MBT\|P29\|PR-3 | 19 | Antimicrobials |
| MAPK1 | 5594 | mitogen-activated protein kinase 1 | ERK\|ERK2\|ERT1\|MAPK2\|P42MAPK\|PRKM1\|PRKM2\|p38\|p40\|p41\|p41mapk | 22 | Antimicrobials |
| PML | 5371 | promyelocytic leukemia | MYL\|PP8675\|RNF71\|TRIM19 | 15 | Antimicrobials |
| AEN | 64782 | apoptosis enhancing nuclease | FLJ12484\|FLJ12562\|ISG20L1\|pp12744 | 15 | Antimicrobials |
| CYBB | 1536 | cytochrome b-245, beta polypeptide | CGD\|GP91-1\|GP91-PHOX\|GP91PHOX\|NOX2\|p91-PHOX | X | Antimicrobials |
| C20orf70 | 140683 | chromosome 20 open reading frame 70 | PSP\|SPLUNC2\|bA49G10.1 | 20 | Antimicrobials |
| ISG20 | 3669 | interferon stimulated exonuclease gene 20kDa | CD25\|HEM45 | 15 | Antimicrobials |
| BCL3 | 602 | B-cell CLL/lymphoma 3 | BCL4\|D19S37 | 19 | Antimicrobials |
| ISG20L2 | 81875 | interferon stimulated exonuclease gene 20kDa-like 2 | FLJ12671 | 1 | Antimicrobials |
| NOX5 | 79400 | NADPH oxidase, EF-hand calcium binding domain 5 | MGC149776\|MGC149777\|NOX5A\|NOX5B | 15 | Antimicrobials |
| NOX3 | 50508 | NADPH oxidase 3 | GP91-3 | 6 | Antimicrobials |
| DUOX2 | 50506 | dual oxidase 2 | LNOX2\|NOXEF2\|P138-TOX\|THOX2 | 15 | Antimicrobials |
| TLR3 | 7098 | toll-like receptor 3 | CD283 | 4 | Antimicrobials |
| TFRC | 7037 | transferrin receptor (p90, CD71) | CD71\|TFR\|TFR1\|TRFR | 3 | Antimicrobials |
| IFIH1 | 64135 | interferon induced with helicase C domain 1 | Hlcd\|IDDM19\|MDA-5\|MDA5\|MGC133047 | 2 | Antimicrobials |
| LRP1 | 4035 | low density lipoprotein-related protein 1 (alpha-2-macroglobulin receptor) | A2MR\|APOER\|APR\|CD91\|FLJ16451\|IGFBP3R\|LRP\|MGC88725\|TGFBR5 | 12 | Antimicrobials |
| TRIM5 | 85363 | tripartite motif-containing 5 | RNF88\|TRIM5alpha | 11 | Antimicrobials |
| IDO1 | 3620 | indoleamine 2,3-dioxygenase 1 | CD107B\|IDO\|INDO | 8 | Antimicrobials |
| GDF15 | 9518 | growth differentiation factor 15 | GDF-15\|MIC-1\|MIC1\|NAG-1\|PDF\|PLAB\|PTGFB | 19 | Antimicrobials |
| NEDD4 | 4734 | neural precursor cell expressed, developmentally down-regulated 4 | KIAA0093\|MGC176705\|NEDD4-1\|RPF1 | 15 | Antimicrobials |
| ADIPOQ | 9370 | adiponectin, C1Q and collagen domain containing | ACDC\|ACRP30\|ADIPQTL1\|ADPN\|APM-1\|APM1\|GBP28\|adiponectin | 3 | Antimicrobials |
| STAT3 | 6774 | signal transducer and activator of transcription 3 (acute-phase response factor) | APRF\|FLJ20882\|HIES\|MGC16063 | 17 | Antimicrobials |
| STAT1 | 6772 | signal transducer and activator of transcription 1, 91kDa | DKFZp686B04100\|ISGF-3\|STAT91 | 2 | Antimicrobials |
| IL28A | 282616 | interleukin 28A (interferon, lambda 2) | IFNL2\|IL-28A | 19 | Antimicrobials |
| SOCS3 | 9021 | suppressor of cytokine signaling 3 | ATOD4\|CIS3\|Cish3\|MGC71791\|SOCS-3\|SSI-3\|SSI3 | 17 | Antimicrobials |
| SEMG1 | 6406 | semenogelin I | MGC14719\|SEMG\|SGI | 20 | Antimicrobials |
| TNFSF10 | 8743 | tumor necrosis factor (ligand) superfamily, member 10 | APO2L\|Apo-2L\|CD253\|TL2\|TRAIL | 3 | Antimicrobials |
| CCL20 | 6364 | chemokine (C-C motif) ligand 20 | CKb4\|LARC\|MIP-3a\|MIP3A\|SCYA20\|ST38 | 2 | Antimicrobials |
| SOCS1 | 8651 | suppressor of cytokine signaling 1 | CIS1\|CISH1\|JAB\|SOCS-1\|SSI-1\|SSI1\|TIP3 | 16 | Antimicrobials |
| RNASEL | 6041 | ribonuclease L (2',5'-oligoisoadenylate synthetase-dependent) | DKFZp781D08126\|MGC104972\|MGC133329\|PRCA1\|RNS4 | 1 | Antimicrobials |
| IRF1 | 3659 | interferon regulatory factor 1 | IRF-1\|MAR | 5 | Antimicrobials |
| IL15 | 3600 | interleukin 15 | IL-15\|MGC9721 | 4 | Antimicrobials |
| APOBEC3F | 200316 | apolipoprotein B mRNA editing enzyme, catalytic polypeptide-like 3F | ARP8\|BK150C2.4.MRNA\|KA6\|MGC74891 | 22 | Antimicrobials |
| RARRES3 | 5920 | retinoic acid receptor responder (tazarotene induced) 3 | HRASLS4\|MGC8906\|PLA1/2-3\|RIG1\|TIG3 | 11 | Antimicrobials |
| CHIT1 | 1118 | chitinase 1 (chitotriosidase) | CHI3\|CHIT\|FLJ00314\|MGC125322 | 1 | Antimicrobials |
| IFNA1 | 3439 | interferon, alpha 1 | IFL\|IFN\|IFN-ALPHA\|IFNA13\|IFNA@\|MGC138207\|MGC138505\|MGC138507 | 9 | Antimicrobials |
| CD40 | 958 | CD40 molecule, TNF receptor superfamily member 5 | Bp50\|CDW40\|MGC9013\|TNFRSF5\|p50 | 20 | Antimicrobials |
| TLR7 | 51284 | toll-like receptor 7 | - | X | Antimicrobials |
| PPIA | 5478 | peptidylprolyl isomerase A (cyclophilin A) | CYPA\|CYPH\|MGC117158\|MGC12404\|MGC23397 | 7 | Antimicrobials |
| HFE | 3077 | hemochromatosis | HFE1\|HH\|HLA-H\|MGC103790\|MVCD7\|dJ221C16.10.1 | 6 | Antimicrobials |
| ZYX | 7791 | zyxin | ESP-2\|HED-2 | 7 | Antimicrobials |
| NLRX1 | 79671 | NLR family member X1 | CLR11.3\|DLNB26\|FLJ21478\|MGC131937\|MGC21025\|NOD26\|NOD5\|NOD9 | 11 | Antimicrobials |
| PGC | 5225 | progastricsin (pepsinogen C) | - | 6 | Antimicrobials |
| VEGFA | 7422 | vascular endothelial growth factor A | MGC70609\|MVCD1\|VEGF\|VEGF-A\|VPF | 6 | Antimicrobials |
| IKBKE | 9641 | inhibitor of kappa light polypeptide gene enhancer in B-cells, kinase epsilon | IKK-i\|IKKE\|IKKI\|KIAA0151\|MGC125294\|MGC125295\|MGC125297 | 1 | Antimicrobials |
| ISG15 | 9636 | ISG15 ubiquitin-like modifier | G1P2\|IFI15\|UCRP | 1 | Antimicrobials |
| DHX58 | 79132 | DEXH (Asp-Glu-X-His) box polypeptide 58 | D11LGP2\|D11lgp2e\|LGP2 | 17 | Antimicrobials |
| TNFAIP3 | 7128 | tumor necrosis factor, alpha-induced protein 3 | A20\|MGC104522\|MGC138687\|MGC138688\|OTUD7C\|TNFA1P2 | 6 | Antimicrobials |
| TFR2 | 7036 | transferrin receptor 2 | HFE3\|MGC126368\|TFRC2 | 7 | Antimicrobials |
| FCN2 | 2220 | ficolin (collagen/fibrinogen domain containing lectin) 2 (hucolin) | EBP-37\|FCNL\|P35\|ficolin-2 | 9 | Antimicrobials |
| MUC4 | 4585 | mucin 4, cell surface associated | HSA276359 | 3 | Antimicrobials |
| F2R | 2149 | coagulation factor II (thrombin) receptor | CF2R\|HTR\|PAR1\|TR | 5 | Antimicrobials |
| ELN | 2006 | elastin | FLJ38671\|FLJ43523\|SVAS\|WBS\|WS | 7 | Antimicrobials |
| IL27 | 246778 | interleukin 27 | IL-27\|IL-27A\|IL27p28\|IL30\|MGC71873\|p28 | 16 | Antimicrobials |
| MAPT | 4137 | microtubule-associated protein tau | DDPAC\|FLJ31424\|FTDP-17\|MAPTL\|MGC138549\|MSTD\|MTBT1\|MTBT2\|PPND\|TAU | 17 | Antimicrobials |
| LYZ | 4069 | lysozyme (renal amyloidosis) | LZM\|lysozyme | 12 | Antimicrobials |
| CCL5 | 6352 | chemokine (C-C motif) ligand 5 | D17S136E\|MGC17164\|RANTES\|SCYA5\|SISd\|TCP228 | 17 | Antimicrobials |
| LEP | 3952 | leptin | FLJ94114\|OB\|OBS | 7 | Antimicrobials |
| CYLD | 1540 | cylindromatosis (turban tumor syndrome) | CDMT\|CYLD1\|CYLDI\|EAC\|FLJ20180\|FLJ31664\|FLJ78684\|HSPC057\|KIAA0849\|MFT\|MFT1\|SBS\|TEM\|USPL2 | 16 | Antimicrobials |
| KLKB1 | 3818 | kallikrein B, plasma (Fletcher factor) 1 | KLK3\|PPK | 4 | Antimicrobials |
| CST4 | 1472 | cystatin S | MGC71923 | 20 | Antimicrobials |
| CSRP1 | 1465 | cysteine and glycine-rich protein 1 | CRP\|CRP1\|CSRP\|CYRP\|D1S181E\|DKFZp686M148 | 1 | Antimicrobials |
| MAPK14 | 1432 | mitogen-activated protein kinase 14 | CSBP1\|CSBP2\|CSPB1\|EXIP\|Mxi2\|PRKM14\|PRKM15\|RK\|SAPK2A\|p38\|p38ALPHA | 6 | Antimicrobials |
| JUN | 3725 | jun oncogene | AP-1\|AP1\|c-Jun | 1 | Antimicrobials |
| ITGAV | 3685 | integrin, alpha V (vitronectin receptor, alpha polypeptide, antigen CD51) | CD51\|DKFZp686A08142\|MSK8\|VNRA | 2 | Antimicrobials |
| IRF5 | 3663 | interferon regulatory factor 5 | SLEB10 | 7 | Antimicrobials |
| CCR6 | 1235 | chemokine (C-C motif) receptor 6 | BN-1\|CD196\|CKR-L3\|CKR6\|CKRL3\|CMKBR6\|DCR2\|DRY-6\|GPR-CY4\|GPR29\|GPRCY4\|STRL22 | 6 | Antimicrobials |
| IL12B | 3593 | interleukin 12B (natural killer cell stimulatory factor 2, cytotoxic lymphocyte maturation factor 2, p40) | CLMF\|CLMF2\|IL-12B\|NKSF\|NKSF2 | 5 | Antimicrobials |
| TLR8 | 51311 | toll-like receptor 8 | CD288\|MGC119599\|MGC119600 | X | Antimicrobials |
| GNLY | 10578 | granulysin | 519\|D2S69E\|LAG-2\|LAG2\|NKG5\|TLA519 | 2 | Antimicrobials |
| CD81 | 975 | CD81 molecule | S5.7\|TAPA1\|TSPAN28 | 11 | Antimicrobials |
| EIF2AK2 | 5610 | eukaryotic translation initiation factor 2-alpha kinase 2 | EIF2AK1\|MGC126524\|PKR\|PRKR | 2 | Antimicrobials |
| APOM | 55937 | apolipoprotein M | G3a\|HSPC336\|MGC22400\|NG20 | 6 | Antimicrobials |
| CACYBP | 27101 | calcyclin binding protein | GIG5\|MGC87971\|PNAS-107\|RP1-102G20.6\|S100A6BP\|SIP | 1 | Antimicrobials |
| NOD1 | 10392 | nucleotide-binding oligomerization domain containing 1 | CARD4\|CLR7.1\|NLRC1 | 7 | Antimicrobials |
| MAPK8 | 5599 | mitogen-activated protein kinase 8 | JNK\|JNK1\|JNK1A2\|JNK21B1/2\|PRKM8\|SAPK1 | 10 | Antimicrobials |
| MAPK3 | 5595 | mitogen-activated protein kinase 3 | ERK1\|HS44KDAP\|HUMKER1A\|MGC20180\|P44ERK1\|P44MAPK\|PRKM3 | 16 | Antimicrobials |
| BST2 | 684 | bone marrow stromal cell antigen 2 | CD317 | 19 | Antimicrobials |
| BPHL | 670 | biphenyl hydrolase-like (serine hydrolase) | BPH-RP\|MCNAA\|MGC125930\|MGC41865\|VACVASE | 6 | Antimicrobials |
| PLA2G2A | 5320 | phospholipase A2, group IIA (platelets, synovial fluid) | MOM1\|PLA2\|PLA2B\|PLA2L\|PLA2S\|PLAS1\|sPLA2 | 1 | Antimicrobials |
| GRN | 2896 | granulin | GEP\|GP88\|PCDGF\|PEPI\|PGRN | 17 | Antimicrobials |
| NEWENTRY | 192343 | Record to support submission of GeneRIFs for a gene not in Entrez Gene (human; man). | - | - | Antimicrobials |
| PDGFRA | 5156 | platelet-derived growth factor receptor, alpha polypeptide | CD140A\|MGC74795\|PDGFR2\|Rhe-PDGFRA | 4 | Antimicrobials |
| GNAI1 | 2770 | guanine nucleotide binding protein (G protein), alpha inhibiting activity polypeptide 1 | Gi | 7 | Antimicrobials |
| WNT5A | 7474 | wingless-type MMTV integration site family, member 5A | hWNT5A | 3 | Antimicrobials |
| FURIN | 5045 | furin (paired basic amino acid cleaving enzyme) | FUR\|PACE\|PCSK3\|SPC1 | 15 | Antimicrobials |
| ADAR | 103 | adenosine deaminase, RNA-specific | ADAR1\|DRADA\|DSH\|DSRAD\|G1P1\|IFI-4\|IFI4\|K88dsRBP\|p136 | 1 | Antimicrobials |
| TYK2 | 7297 | tyrosine kinase 2 | JTK1 | 19 | Antimicrobials |
| NOS2 | 4843 | nitric oxide synthase 2, inducible | HEP-NOS\|INOS\|NOS\|NOS2A | 17 | Antimicrobials |
| TRAF3 | 7187 | TNF receptor-associated factor 3 | CAP-1\|CD40bp\|CRAF1\|LAP1 | 14 | Antimicrobials |
| TPT1 | 7178 | tumor protein, translationally-controlled 1 | FLJ27337\|HRF\|TCTP\|p02 | 13 | Antimicrobials |
| TPM2 | 7169 | tropomyosin 2 (beta) | AMCD1\|DA1\|DA2B\|TMSB | 9 | Antimicrobials |
| NEO1 | 4756 | neogenin homolog 1 (chicken) | DKFZp547A066\|DKFZp547B146\|HsT17534\|IGDCC2\|NGN | 15 | Antimicrobials |
| AHNAK | 79026 | AHNAK nucleoprotein | AHNAKRS\|MGC5395 | 11 | Antimicrobials |
| TLR1 | 7096 | toll-like receptor 1 | CD281\|DKFZp547I0610\|DKFZp564I0682\|KIAA0012\|MGC104956\|MGC126311\|MGC126312\|TIL\|rsc786 | 4 | Antimicrobials |
| TK2 | 7084 | thymidine kinase 2, mitochondrial | - | 16 | Antimicrobials |
| PRDX2 | 7001 | peroxiredoxin 2 | MGC4104\|NKEFB\|PRP\|PRX2\|PRXII\|TDPX1\|TSA | 19 | Antimicrobials |
| MX2 | 4600 | myxovirus (influenza virus) resistance 2 (mouse) | MXB | 21 | Antimicrobials |
| FGF2 | 2247 | fibroblast growth factor 2 (basic) | BFGF\|FGFB\|HBGF-2 | 4 | Antimicrobials |
| FGA | 2243 | fibrinogen alpha chain | Fib2\|MGC119422\|MGC119423\|MGC119425 | 4 | Antimicrobials |
| TCF7L2 | 6934 | transcription factor 7-like 2 (T-cell specific, HMG-box) | TCF-4\|TCF4 | 10 | Antimicrobials |
| F2RL1 | 2150 | coagulation factor II (thrombin) receptor-like 1 | GPR11\|PAR2 | 5 | Antimicrobials |
| DAK | 26007 | dihydroxyacetone kinase 2 homolog (S. cerevisiae) | DKFZp586B1621\|MGC5621 | 11 | Antimicrobials |
| MSR1 | 4481 | macrophage scavenger receptor 1 | CD204\|SCARA1\|SR-A\|phSR1\|phSR2 | 8 | Antimicrobials |
| NFKBIZ | 64332 | nuclear factor of kappa light polypeptide gene enhancer in B-cells inhibitor, zeta | FLJ30225\|FLJ34463\|IKBZ\|INAP\|MAIL | 3 | Antimicrobials |
| LMBR1 | 64327 | limb region 1 homolog (mouse) | ACHP\|C7orf2\|DIF14\|FLJ11665\|PPD2\|TPT | 7 | Antimicrobials |
| SPINLW1 | 57119 | serine peptidase inhibitor-like, with Kunitz and WAP domains 1 (eppin) | EPPIN\|EPPIN1\|EPPIN2\|EPPIN3\|WAP7\|WFDC7\|dJ461P17.2 | 20 | Antimicrobials |
| SRC | 6714 | v-src sarcoma (Schmidt-Ruppin A-2) viral oncogene homolog (avian) | ASV\|SRC1\|c-SRC\|p60-Src | 20 | Antimicrobials |
| MPO | 4353 | myeloperoxidase | - | 17 | Antimicrobials |
| ELAVL1 | 1994 | ELAV (embryonic lethal, abnormal vision, Drosophila)-like 1 (Hu antigen R) | ELAV1\|HUR\|Hua\|MelG | 19 | Antimicrobials |
| ROBO3 | 64221 | roundabout, axon guidance receptor, homolog 3 (Drosophila) | FLJ21044\|HGPPS\|HGPS\|RBIG1\|RIG1 | 11 | Antimicrobials |
| SP1 | 6667 | Sp1 transcription factor | - | 12 | Antimicrobials |
| SOD1 | 6647 | superoxide dismutase 1, soluble | ALS\|ALS1\|IPOA\|SOD\|homodimer | 21 | Antimicrobials |
| PDF | 64146 | peptide deformylase (mitochondrial) | - | 16 | Antimicrobials |
| DLL4 | 54567 | delta-like 4 (Drosophila) | MGC126344\|hdelta2 | 15 | Antimicrobials |
| ECD | 11319 | ecdysoneless homolog (Drosophila) | GCR2\|HSGT1 | 10 | Antimicrobials |
| SLC11A1 | 6556 | solute carrier family 11 (proton-coupled divalent metal ion transporters), member 1 | LSH\|NRAMP\|NRAMP1 | 2 | Antimicrobials |
| DMBT1 | 1755 | deleted in malignant brain tumors 1 | GP340\|MGC164738\|muclin | 10 | Antimicrobials |
| TMEM173 | 340061 | transmembrane protein 173 | FLJ38577\|MITA\|MPYS\|STING | 5 | Antimicrobials |
| SKIV2L | 6499 | superkiller viralicidic activity 2-like (S. cerevisiae) | 170A\|DDX13\|HLP\|SKI2\|SKI2W\|SKIV2 | 6 | Antimicrobials |
| SEMG2 | 6407 | semenogelin II | SGII | 20 | Antimicrobials |
| LTA | 4049 | lymphotoxin alpha (TNF superfamily, member 1) | LT\|TNFB\|TNFSF1 | 6 | Antimicrobials |
| DES | 1674 | desmin | CMD1I\|CSM1\|CSM2\|FLJ12025\|FLJ39719\|FLJ41013\|FLJ41793 | 2 | Antimicrobials |
| DCK | 1633 | deoxycytidine kinase | MGC117410\|MGC138632 | 4 | Antimicrobials |
| DAXX | 1616 | death-domain associated protein | BING2\|DAP6\|EAP1\|MGC126245\|MGC126246 | 6 | Antimicrobials |
| TNFRSF10A | 8797 | tumor necrosis factor receptor superfamily, member 10a | APO2\|CD261\|DR4\|MGC9365\|TRAILR-1\|TRAILR1 | 8 | Antimicrobials |
| TNFRSF10B | 8795 | tumor necrosis factor receptor superfamily, member 10b | CD262\|DR5\|KILLER\|KILLER/DR5\|TRAIL-R2\|TRAILR2\|TRICK2\|TRICK2A\|TRICK2B\|TRICKB\|ZTNFR9 | 8 | Antimicrobials |
| EED | 8726 | embryonic ectoderm development | HEED\|WAIT1 | 11 | Antimicrobials |
| CCL4 | 6351 | chemokine (C-C motif) ligand 4 | ACT2\|AT744.1\|G-26\|LAG1\|MGC104418\|MGC126025\|MGC126026\|MIP-1-beta\|MIP1B\|MIP1B1\|SCYA2\|SCYA4 | 17 | Antimicrobials |
| LIMS1 | 3987 | LIM and senescent cell antigen-like domains 1 | PINCH\|PINCH1 | 2 | Antimicrobials |
| LALBA | 3906 | lactalbumin, alpha- | MGC138521\|MGC138523 | 12 | Antimicrobials |
| APOBEC3H | 164668 | apolipoprotein B mRNA editing enzyme, catalytic polypeptide-like 3H | ARP10\|dJ742C19.2 | 22 | Antimicrobials |
| TMPRSS6 | 164656 | transmembrane protease, serine 6 | IRIDA | 22 | Antimicrobials |
| SPINK5 | 11005 | serine peptidase inhibitor, Kazal type 5 | DKFZp686K19184\|FLJ21544\|FLJ97536\|FLJ97596\|FLJ99794\|LEKTI\|LETKI\|NETS\|NS\|VAKTI | 5 | Antimicrobials |
| MARCO | 8685 | macrophage receptor with collagenous structure | SCARA2 | 2 | Antimicrobials |
| BECN1 | 8678 | beclin 1, autophagy related | ATG6\|VPS30\|beclin1 | 17 | Antimicrobials |
| TNFSF11 | 8600 | tumor necrosis factor (ligand) superfamily, member 11 | CD254\|ODF\|OPGL\|OPTB2\|RANKL\|TRANCE\|hRANKL2\|sOdf | 13 | Antimicrobials |
| KNG1 | 3827 | kininogen 1 | BDK\|KNG | 3 | Antimicrobials |
| CSK | 1445 | c-src tyrosine kinase | MGC117393 | 15 | Antimicrobials |
| KLRK1 | 22914 | killer cell lectin-like receptor subfamily K, member 1 | CD314\|D12S2489E\|FLJ17759\|FLJ75772\|KLR\|NKG2-D\|NKG2D | 12 | Antimicrobials |
| KCNH2 | 3757 | potassium voltage-gated channel, subfamily H (eag-related), member 2 | ERG1\|HERG\|HERG1\|Kv11.1\|LQT2\|SQT1 | 7 | Antimicrobials |
| JUND | 3727 | jun D proto-oncogene | AP-1 | 19 | Antimicrobials |
| JAK1 | 3716 | Janus kinase 1 | JAK1A\|JAK1B\|JTK3 | 1 | Antimicrobials |
| CREB1 | 1385 | cAMP responsive element binding protein 1 | CREB\|MGC9284 | 2 | Antimicrobials |
| CLDN4 | 1364 | claudin 4 | CPE-R\|CPER\|CPETR\|CPETR1\|WBSCR8\|hCPE-R | 7 | Antimicrobials |
| CCL28 | 56477 | chemokine (C-C motif) ligand 28 | CCK1\|MEC\|MGC71902\|SCYA28 | 5 | Antimicrobials |
| RNASE3 | 6037 | ribonuclease, RNase A family, 3 (eosinophil cationic protein) | ECP\|RNS3 | 14 | Antimicrobials |
| RN7SL1 | 6029 | RNA, 7SL, cytoplasmic 1 | 7L1a\|7SL\|RN7SL\|RNSRP1 | 14 | Antimicrobials |
| IRF7 | 3665 | interferon regulatory factor 7 | IRF-7H\|IRF7A | 11 | Antimicrobials |
| IREB2 | 3658 | iron-responsive element binding protein 2 | ACO3\|FLJ23381\|IRP2\|IRP2AD | 15 | Antimicrobials |
| ILK | 3611 | integrin-linked kinase | DKFZp686F1765\|P59 | 11 | Antimicrobials |
| IL18 | 3606 | interleukin 18 (interferon-gamma-inducing factor) | IGIF\|IL-18\|IL-1g\|IL1F4\|MGC12320 | 11 | Antimicrobials |
| IL17A | 3605 | interleukin 17A | CTLA8\|IL-17\|IL-17A\|IL17 | 6 | Antimicrobials |
| LTB4R | 1241 | leukotriene B4 receptor | BLT1\|BLTR\|CMKRL1\|GPR16\|LTB4R1\|LTBR1\|P2RY7\|P2Y7 | 14 | Antimicrobials |
| APOBEC3A | 200315 | apolipoprotein B mRNA editing enzyme, catalytic polypeptide-like 3A | ARP3\|PHRBN\|bK150C2.1 | 22 | Antimicrobials |
| MASP2 | 10747 | mannan-binding lectin serine peptidase 2 | MAP19\|MASP-2\|sMAP | 1 | Antimicrobials |
| TRIM27 | 5987 | tripartite motif-containing 27 | RFP\|RNF76 | 6 | Antimicrobials |
| RELA | 5970 | v-rel reticuloendotheliosis viral oncogene homolog A (avian) | MGC131774\|NFKB3\|p65 | 11 | Antimicrobials |
| IL7R | 3575 | interleukin 7 receptor | CD127\|CDW127\|IL-7R-alpha\|IL7RA\|ILRA | 5 | Antimicrobials |
| IL1A | 3552 | interleukin 1, alpha | IL-1A\|IL1\|IL1-ALPHA\|IL1F1 | 2 | Antimicrobials |
| PTX3 | 5806 | pentraxin-related gene, rapidly induced by IL-1 beta | TNFAIP5\|TSG-14 | 3 | Antimicrobials |
| IFNAR2 | 3455 | interferon (alpha, beta and omega) receptor 2 | IFN-R\|IFN-alpha-REC\|IFNABR\|IFNARB | 21 | Antimicrobials |
| IFN1@ | 3438 | interferon, type 1, cluster | IFNA | 9 | Antimicrobials |
| SYTL1 | 84958 | synaptotagmin-like 1 | FLJ14996\|JFC1\|SLP1 | 1 | Antimicrobials |
| APOBEC3C | 27350 | apolipoprotein B mRNA editing enzyme, catalytic polypeptide-like 3C | APOBEC1L\|ARDC2\|ARDC4\|ARP5\|MGC19485\|PBI\|bK150C2.3 | 22 | Antimicrobials |
| DDX17 | 10521 | DEAD (Asp-Glu-Ala-Asp) box polypeptide 17 | DKFZp761H2016\|P72\|RH70 | 22 | Antimicrobials |
| PTGS2 | 5743 | prostaglandin-endoperoxide synthase 2 (prostaglandin G/H synthase and cyclooxygenase) | COX-2\|COX2\|GRIPGHS\|PGG/HS\|PGHS-2\|PHS-2\|hCox-2 | 1 | Antimicrobials |
| HTR1A | 3350 | 5-hydroxytryptamine (serotonin) receptor 1A | 5-HT1A\|5HT1a\|ADRB2RL1\|ADRBRL1 | 5 | Antimicrobials |
| SEPT-7 | 989 | septin 7 | CDC10\|CDC3\|Nbla02942\|SEPT7A | 7 | Antimicrobials |
| CD40LG | 959 | CD40 ligand | CD154\|CD40L\|HIGM1\|IGM\|IMD3\|T-BAM\|TNFSF5\|TRAP\|gp39\|hCD40L | X | Antimicrobials |
| CD14 | 929 | CD14 molecule | - | 5 | Antimicrobials |
| CD8A | 925 | CD8a molecule | CD8\|Leu2\|MAL\|p32 | 2 | Antimicrobials |
| CD4 | 920 | CD4 molecule | CD4mut | 12 | Antimicrobials |
| MASP1 | 5648 | mannan-binding lectin serine peptidase 1 (C4/C2 activating component of Ra-reactive factor) | CRARF\|CRARF1\|DKFZp686I01199\|FLJ26383\|MASP\|MGC126283\|MGC126284\|PRSS5\|RaRF | 3 | Antimicrobials |
| PROC | 5624 | protein C (inactivator of coagulation factors Va and VIIIa) | PC\|PROC1 | 2 | Antimicrobials |
| MAP2K2 | 5605 | mitogen-activated protein kinase kinase 2 | FLJ26075\|MAPKK2\|MEK2\|MKK2\|PRKMK2 | 19 | Antimicrobials |
| MAP2K1 | 5604 | mitogen-activated protein kinase kinase 1 | MAPKK1\|MEK1\|MKK1\|PRKMK1 | 15 | Antimicrobials |
| HRG | 3273 | histidine-rich glycoprotein | DKFZp779H1622\|HPRG\|HRGP | 3 | Antimicrobials |
| NDRG1 | 10397 | N-myc downstream regulated 1 | CAP43\|CMT4D\|DRG1\|GC4\|HMSNL\|NDR1\|NMSL\|PROXY1\|RIT42\|RTP\|TARG1\|TDD5 | 8 | Antimicrobials |
| IRF9 | 10379 | interferon regulatory factor 9 | IRF-9\|ISGF3\|ISGF3G\|p48 | 14 | Antimicrobials |
| TRIM22 | 10346 | tripartite motif-containing 22 | GPSTAF50\|RNF94\|STAF50 | 11 | Antimicrobials |
| LANCL1 | 10314 | LanC lantibiotic synthetase component C-like 1 (bacterial) | GPR69A\|p40 | 2 | Antimicrobials |
| PPP4C | 5531 | protein phosphatase 4 (formerly X), catalytic subunit | PP4\|PPH3\|PPX | 16 | Antimicrobials |
| HMOX1 | 3162 | heme oxygenase (decycling) 1 | HO-1\|HSP32\|bK286B10 | 22 | Antimicrobials |
| HMGB1 | 3146 | high-mobility group box 1 | DKFZp686A04236\|HMG1\|HMG3\|SBP-1 | 13 | Antimicrobials |
| HLA-B | 3106 | major histocompatibility complex, class I, B | AS\|HLA-B-7301\|HLA-B73\|HLAB\|HLAC\|MGC111087\|SPDA1 | 6 | Antimicrobials |
| RNASE7 | 84659 | ribonuclease, RNase A family, 7 | MGC133220 | 14 | Antimicrobials |
| ABCC4 | 10257 | ATP-binding cassette, sub-family C (CFTR/MRP), member 4 | EST170205\|MOAT-B\|MOATB\|MRP4 | 13 | Antimicrobials |
| HGF | 3082 | hepatocyte growth factor (hepapoietin A; scatter factor) | F-TCF\|HGFB\|HPTA\|SF | 7 | Antimicrobials |
| HDAC1 | 3065 | histone deacetylase 1 | DKFZp686H12203\|GON-10\|HD1\|RPD3\|RPD3L1 | 1 | Antimicrobials |
| IL28RA | 163702 | interleukin 28 receptor, alpha (interferon, lambda receptor) | CRF2/12\|IFNLR\|IFNLR1\|IL-28R1\|LICR2 | 1 | Antimicrobials |
| PLSCR1 | 5359 | phospholipid scramblase 1 | MMTRA1B | 3 | Antimicrobials |
| B2M | 567 | beta-2-microglobulin | - | 15 | Antimicrobials |
| BACH2 | 60468 | BTB and CNC homology 1, basic leucine zipper transcription factor 2 | - | 6 | Antimicrobials |
| TANK | 10010 | TRAF family member-associated NFKB activator | I-TRAF\|TRAF2 | 2 | Antimicrobials |
| PIK3CG | 5294 | phosphoinositide-3-kinase, catalytic, gamma polypeptide | PI3CG\|PI3K\|PI3Kgamma\|PIK3 | 7 | Antimicrobials |
| ARRB1 | 408 | arrestin, beta 1 | ARB1\|ARR1 | 11 | Antimicrobials |
| RSAD2 | 91543 | radical S-adenosyl methionine domain containing 2 | 2510004L01Rik\|cig33\|cig5\|vig1 | 2 | Antimicrobials |
| STAB2 | 55576 | stabilin 2 | DKFZp434E0321\|FEEL-2\|FELE-2\|FELL\|FELL-2\|FEX2\|HARE\|STAB-2 | 12 | Antimicrobials |
| TBK1 | 29110 | TANK-binding kinase 1 | FLJ11330\|NAK\|T2K | 12 | Antimicrobials |
| PDYN | 5173 | prodynorphin | MGC26418\|PENKB | 20 | Antimicrobials |
| PDGFRB | 5159 | platelet-derived growth factor receptor, beta polypeptide | CD140B\|JTK12\|PDGF-R-beta\|PDGFR\|PDGFR1 | 5 | Antimicrobials |
| PDCD1 | 5133 | programmed cell death 1 | CD279\|PD1\|SLEB2\|hPD-1\|hPD-l | 2 | Antimicrobials |
| PCSK2 | 5126 | proprotein convertase subtilisin/kexin type 2 | NEC2\|PC2\|SPC2 | 20 | Antimicrobials |
| PCSK1 | 5122 | proprotein convertase subtilisin/kexin type 1 | BMIQ12\|NEC1\|PC1\|PC3\|SPC3 | 5 | Antimicrobials |
| ARG2 | 384 | arginase, type II | - | 14 | Antimicrobials |
| AQP9 | 366 | aquaporin 9 | HsT17287\|SSC1 | 15 | Antimicrobials |
| FASLG | 356 | Fas ligand (TNF superfamily, member 6) | APT1LG1\|CD178\|CD95L\|FASL\|TNFSF6 | 1 | Antimicrobials |
| APOH | 350 | apolipoprotein H (beta-2-glycoprotein I) | B2G1\|BG | 17 | Antimicrobials |
| BIRC5 | 332 | baculoviral IAP repeat-containing 5 | API4\|EPR-1 | 17 | Antimicrobials |
| ANXA6 | 309 | annexin A6 | ANX6\|CBP68 | 5 | Antimicrobials |
| IL22 | 50616 | interleukin 22 | IL-21\|IL-22\|IL-D110\|IL-TIF\|IL21\|ILTIF\|MGC79382\|MGC79384\|TIFIL-23\|TIFa\|zcyto18 | 12 | Antimicrobials |
| VTN | 7448 | vitronectin | V75\|VN\|VNT | 17 | Antimicrobials |
| VIM | 7431 | vimentin | FLJ36605 | 10 | Antimicrobials |
| VCAM1 | 7412 | vascular cell adhesion molecule 1 | CD106\|DKFZp779G2333\|INCAM-100\|MGC99561 | 1 | Antimicrobials |
| PRDX1 | 5052 | peroxiredoxin 1 | MSP23\|NKEFA\|PAG\|PAGA\|PAGB\|PRX1\|PRXI\|TDPX2 | 1 | Antimicrobials |
| GFAP | 2670 | glial fibrillary acidic protein | FLJ45472 | 17 | Antimicrobials |
| GBP2 | 2634 | guanylate binding protein 2, interferon-inducible | - | 1 | Antimicrobials |
| ALB | 213 | albumin | DKFZp779N1935\|PRO0883\|PRO0903\|PRO1341 | 4 | Antimicrobials |
| SLC29A3 | 55315 | solute carrier family 29 (nucleoside transporters), member 3 | ENT3\|FLJ11160 | 10 | Antimicrobials |
| OAS1 | 4938 | 2',5'-oligoadenylate synthetase 1, 40/46kDa | IFI-4\|OIAS\|OIASI | 12 | Antimicrobials |
| AGER | 177 | advanced glycosylation end product-specific receptor | MGC22357\|RAGE | 6 | Antimicrobials |
| UNC93B1 | 81622 | unc-93 homolog B1 (C. elegans) | MGC126617\|UNC93\|UNC93B | 11 | Antimicrobials |
| TNFSF4 | 7292 | tumor necrosis factor (ligand) superfamily, member 4 | CD134L\|CD252\|GP34\|OX-40L\|OX4OL\|TXGP1 | 1 | Antimicrobials |
| NOS1 | 4842 | nitric oxide synthase 1 (neuronal) | IHPS1\|NOS\|nNOS | 12 | Antimicrobials |
| ACTG1 | 71 | actin, gamma 1 | ACT\|ACTG\|DFNA20\|DFNA26 | 17 | Antimicrobials |
| ACTA1 | 58 | actin, alpha 1, skeletal muscle | ACTA\|ASMA\|CFTD\|CFTD1\|CFTDM\|MPFD\|NEM1\|NEM2\|NEM3 | 1 | Antimicrobials |
| ACO1 | 48 | aconitase 1, soluble | ACONS\|IREB1\|IREBP\|IREBP1\|IRP1 | 9 | Antimicrobials |
| SERPINA3 | 12 | serpin peptidase inhibitor, clade A (alpha-1 antiproteinase, antitrypsin), member 3 | AACT\|ACT\|GIG24\|GIG25\|MGC88254 | 14 | Antimicrobials |
| IL8RA | 3577 | interleukin 8 receptor, alpha | C-C\|C-C-CKR-1\|CD128\|CD181\|CDw128a\|CKR-1\|CMKAR1\|CXCR1\|IL8R1\|IL8RBA | 2 | Antimicrobials |
| CCL15 | 6359 | chemokine (C-C motif) ligand 15 | HCC-2\|HMRP-2B\|LKN1\|Lkn-1\|MIP-1d\|MIP-5\|NCC-3\|NCC3\|SCYA15\|SCYL3\|SY15 | 17 | Antimicrobials |
| CCL14 | 6358 | chemokine (C-C motif) ligand 14 | CC-1\|CC-3\|CKb1\|FLJ16015\|HCC-1\|HCC-3\|MCIF\|NCC-2\|NCC2\|SCYA14\|SCYL2\|SY14 | 17 | Antimicrobials |
| CCL4 | 6351 | chemokine (C-C motif) ligand 4 | ACT2\|AT744.1\|G-26\|LAG1\|MGC104418\|MGC126025\|MGC126026\|MIP-1-beta\|MIP1B\|MIP1B1\|SCYA2\|SCYA4 | 17 | Antimicrobials |
| CCL16 | 6360 | chemokine (C-C motif) ligand 16 | CKb12\|HCC-4\|ILINCK\|LCC-1\|LEC\|LMC\|MGC117051\|Mtn-1\|NCC-4\|NCC4\|SCYA16\|SCYL4 | 17 | Antimicrobials |
| CCL19 | 6363 | chemokine (C-C motif) ligand 19 | CKb11\|ELC\|MGC34433\|MIP-3b\|MIP3B\|SCYA19 | 9 | Antimicrobials |
| CCL13 | 6357 | chemokine (C-C motif) ligand 13 | CKb10\|MCP-4\|MGC17134\|NCC-1\|NCC1\|SCYA13\|SCYL1 | 17 | Antimicrobials |
| CCL18 | 6362 | chemokine (C-C motif) ligand 18 (pulmonary and activation-regulated) | AMAC-1\|AMAC1\|CKb7\|DC-CK1\|DCCK1\|MIP-4\|PARC\|SCYA18 | 17 | Antimicrobials |
| CCL17 | 6361 | chemokine (C-C motif) ligand 17 | A-152E5.3\|ABCD-2\|MGC138271\|MGC138273\|SCYA17\|TARC | 16 | Antimicrobials |
| CCL26 | 10344 | chemokine (C-C motif) ligand 26 | IMAC\|MGC126714\|MIP-4a\|MIP-4alpha\|SCYA26\|TSC-1 | 7 | Antimicrobials |
| CCL22 | 6367 | chemokine (C-C motif) ligand 22 | A-152E5.1\|ABCD-1\|DC/B-CK\|MDC\|MGC34554\|SCYA22\|STCP-1 | 16 | Antimicrobials |
| CCR3 | 1232 | chemokine (C-C motif) receptor 3 | CC-CKR-3\|CD193\|CKR3\|CMKBR3\|MGC102841 | 3 | Antimicrobials |
| CCL28 | 56477 | chemokine (C-C motif) ligand 28 | CCK1\|MEC\|MGC71902\|SCYA28 | 5 | Antimicrobials |
| CCL4L2 | 388372 | chemokine (C-C motif) ligand 4-like 2 | AT744.2\|CCL4L\|SCYA4L | 17 | Antimicrobials |
| CCBP2 | 1238 | chemokine binding protein 2 | CCR10\|CCR9\|CMKBR9\|D6\|MGC126678\|MGC138250\|hD6 | 3 | Antimicrobials |
| CCR7 | 1236 | chemokine (C-C motif) receptor 7 | BLR2\|CD197\|CDw197\|CMKBR7\|EBI1 | 17 | Antimicrobials |
| CCL27 | 10850 | chemokine (C-C motif) ligand 27 | ALP\|CTACK\|CTAK\|ESKINE\|ILC\|PESKY\|SCYA27 | 9 | Antimicrobials |
| CCR8 | 1237 | chemokine (C-C motif) receptor 8 | CDw198\|CKR-L1\|CKRL1\|CMKBR8\|CMKBRL2\|CY6\|GPR-CY6\|MGC129966\|MGC129973\|TER1 | 3 | Antimicrobials |
| CCRL1 | 51554 | chemokine (C-C motif) receptor-like 1 | CC-CKR-11\|CCBP2\|CCR10\|CCR11\|CCX-CKR\|CKR-11\|PPR1\|VSHK1 | 3 | Antimicrobials |
| CCR10 | 2826 | chemokine (C-C motif) receptor 10 | GPR2 | 17 | Antimicrobials |
| CCL2 | 6347 | chemokine (C-C motif) ligand 2 | GDCF-2\|HC11\|HSMCR30\|MCAF\|MCP-1\|MCP1\|MGC9434\|SCYA2\|SMC-CF | 17 | Antimicrobials |
| CCL21 | 6366 | chemokine (C-C motif) ligand 21 | 6Ckine\|CKb9\|ECL\|MGC34555\|SCYA21\|SLC\|TCA4 | 9 | Antimicrobials |
| CCL7 | 6354 | chemokine (C-C motif) ligand 7 | FIC\|MARC\|MCP-3\|MCP3\|MGC138463\|MGC138465\|NC28\|SCYA6\|SCYA7 | 17 | Antimicrobials |
| CCL5 | 6352 | chemokine (C-C motif) ligand 5 | D17S136E\|MGC17164\|RANTES\|SCYA5\|SISd\|TCP228 | 17 | Antimicrobials |
| CCL3 | 6348 | chemokine (C-C motif) ligand 3 | G0S19-1\|LD78ALPHA\|MIP-1-alpha\|MIP1A\|SCYA3 | 17 | Antimicrobials |
| CCL20 | 6364 | chemokine (C-C motif) ligand 20 | CKb4\|LARC\|MIP-3a\|MIP3A\|SCYA20\|ST38 | 2 | Antimicrobials |
| CCL11 | 6356 | chemokine (C-C motif) ligand 11 | MGC22554\|SCYA11 | 17 | Antimicrobials |
| CCR5 | 1234 | chemokine (C-C motif) receptor 5 | CC-CKR-5\|CCCKR5\|CD195\|CKR-5\|CKR5\|CMKBR5\|FLJ78003\|IDDM22 | 3 | Antimicrobials |
| CCL23 | 6368 | chemokine (C-C motif) ligand 23 | CK-BETA-8\|CKb8\|Ckb-8\|Ckb-8-1\|MIP-3\|MIP3\|MPIF-1\|SCYA23 | 17 | Antimicrobials |
| CCL25 | 6370 | chemokine (C-C motif) ligand 25 | Ckb15\|MGC150327\|SCYA25\|TECK | 19 | Antimicrobials |
| CCL1 | 6346 | chemokine (C-C motif) ligand 1 | I-309\|P500\|SCYA1\|SISe\|TCA3 | 17 | Antimicrobials |
| CCL3L3 | 414062 | chemokine (C-C motif) ligand 3-like 3 | 464.2\|D17S1718\|LD78\|LD78BETA\|MGC12815\|SCYA3L\|SCYA3L1 | 17 | Antimicrobials |
| CCL4L1 | 9560 | chemokine (C-C motif) ligand 4-like 1 | AT744.2\|CCL4L\|LAG-1\|LAG1\|SCYA4L | 17 | Antimicrobials |
| CXCL12 | 6387 | chemokine (C-X-C motif) ligand 12 (stromal cell-derived factor 1) | PBSF\|SCYB12\|SDF-1a\|SDF-1b\|SDF1\|SDF1A\|SDF1B\|TLSF-a\|TLSF-b\|TPAR1 | 10 | Antimicrobials |
| XCL1 | 6375 | chemokine (C motif) ligand 1 | ATAC\|LPTN\|LTN\|SCM-1\|SCM-1a\|SCM1\|SCYC1 | 1 | Antimicrobials |
| CCL8 | 6355 | chemokine (C-C motif) ligand 8 | HC14\|MCP-2\|MCP2\|SCYA10\|SCYA8 | 17 | Antimicrobials |
| CCL3L1 | 6349 | chemokine (C-C motif) ligand 3-like 1 | 464.2\|D17S1718\|G0S19-2\|LD78\|LD78BETA\|MGC104178\|MGC12815\|MGC182017\|MIP1AP\|SCYA3L\|SCYA3L1 | 17 | Antimicrobials |
| CCR1 | 1230 | chemokine (C-C motif) receptor 1 | CD191\|CKR-1\|CKR1\|CMKBR1\|HM145\|MIP1aR\|SCYAR1 | 3 | Antimicrobials |
| CCL24 | 6369 | chemokine (C-C motif) ligand 24 | Ckb-6\|MPIF-2\|MPIF2\|SCYA24 | 7 | Antimicrobials |
| XCL2 | 6846 | chemokine (C motif) ligand 2 | SCM-1b\|SCM1B\|SCYC2 | 1 | Antimicrobials |
| CXCL1 | 2919 | chemokine (C-X-C motif) ligand 1 (melanoma growth stimulating activity, alpha) | FSP\|GRO1\|GROa\|MGSA\|MGSA-a\|NAP-3\|SCYB1 | 4 | Antimicrobials |
| CXCL10 | 3627 | chemokine (C-X-C motif) ligand 10 | C7\|IFI10\|INP10\|IP-10\|SCYB10\|crg-2\|gIP-10\|mob-1 | 4 | Antimicrobials |
| CXCR4 | 7852 | chemokine (C-X-C motif) receptor 4 | CD184\|D2S201E\|FB22\|HM89\|HSY3RR\|LAP3\|LCR1\|LESTR\|NPY3R\|NPYR\|NPYRL\|NPYY3R\|WHIM | 2 | Antimicrobials |
| CXCL2 | 2920 | chemokine (C-X-C motif) ligand 2 | CINC-2a\|GRO2\|GROb\|MGSA-b\|MIP-2a\|MIP2\|MIP2A\|SCYB2 | 4 | Antimicrobials |
| CXCR6 | 10663 | chemokine (C-X-C motif) receptor 6 | BONZO\|CD186\|STRL33\|TYMSTR | 3 | Antimicrobials |
| CCR4 | 1233 | chemokine (C-C motif) receptor 4 | CC-CKR-4\|CD194\|CKR4\|CMKBR4\|ChemR13\|HGCN:14099\|K5-5\|MGC88293 | 3 | Antimicrobials |
| CXCL11 | 6373 | chemokine (C-X-C motif) ligand 11 | H174\|I-TAC\|IP-9\|IP9\|MGC102770\|SCYB11\|SCYB9B\|b-R1 | 4 | Antimicrobials |
| FAM19A5 | 25817 | family with sequence similarity 19 (chemokine (C-C motif)-like), member A5 | QLLK5208\|TAFA-5\|TAFA5\|UNQ5208 | 22 | Antimicrobials |
| FAM19A3 | 284467 | family with sequence similarity 19 (chemokine (C-C motif)-like), member A3 | MGC138473\|TAFA-3\|TAFA3 | 1 | Antimicrobials |
| FAM19A4 | 151647 | family with sequence similarity 19 (chemokine (C-C motif)-like), member A4 | FLJ25161\|TAFA-4\|TAFA4 | 3 | Antimicrobials |
| FAM19A1 | 407738 | family with sequence similarity 19 (chemokine (C-C motif)-like), member A1 | TAFA-1\|TAFA1 | 3 | Antimicrobials |
| FAM19A2 | 338811 | family with sequence similarity 19 (chemokine (C-C motif)-like), member A2 | DKFZp761E1217\|DKFZp781P0552\|MGC42403\|TAFA-2\|TAFA2 | 12 | Antimicrobials |
| CCL14-CCL15 | 348249 | chemokine ligand 14, chemokine ligand 15 transcription unit | - | 17 | Antimicrobials |
| IL6 | 3569 | interleukin 6 (interferon, beta 2) | BSF2\|HGF\|HSF\|IFNB2\|IL-6 | 7 | Antimicrobials |
| TNF | 7124 | tumor necrosis factor (TNF superfamily, member 2) | DIF\|TNF-alpha\|TNFA\|TNFSF2 | 6 | Antimicrobials |
| IL1B | 3553 | interleukin 1, beta | IL-1\|IL1-BETA\|IL1F2 | 2 | Antimicrobials |
| IL18 | 3606 | interleukin 18 (interferon-gamma-inducing factor) | IGIF\|IL-18\|IL-1g\|IL1F4\|MGC12320 | 11 | Antimicrobials |
| PTK2B | 2185 | PTK2B protein tyrosine kinase 2 beta | CADTK\|CAKB\|FADK2\|FAK2\|FRNK\|PKB\|PTK\|PYK2\|RAFTK | 8 | Antimicrobials |
| VEGFA | 7422 | vascular endothelial growth factor A | MGC70609\|MVCD1\|VEGF\|VEGF-A\|VPF | 6 | Antimicrobials |
| IL4 | 3565 | interleukin 4 | BCGF-1\|BCGF1\|BSF1\|IL-4\|MGC79402 | 5 | Antimicrobials |
| CDH1 | 999 | cadherin 1, type 1, E-cadherin (epithelial) | Arc-1\|CD324\|CDHE\|ECAD\|LCAM\|UVO | 16 | Antimicrobials |
| CD40 | 958 | CD40 molecule, TNF receptor superfamily member 5 | Bp50\|CDW40\|MGC9013\|TNFRSF5\|p50 | 20 | Antimicrobials |
| DEFB103A | 55894 | defensin, beta 103A | DEFB103\|DEFB3\|HBD-3\|HBD3\|HBP-3\|HBP3 | 8 | Antimicrobials |
| F2RL1 | 2150 | coagulation factor II (thrombin) receptor-like 1 | GPR11\|PAR2 | 5 | Antimicrobials |
| MMP9 | 4318 | matrix metallopeptidase 9 (gelatinase B, 92kDa gelatinase, 92kDa type IV collagenase) | CLG4B\|GELB\|MMP-9 | 20 | Antimicrobials |
| LTBP1 | 4052 | latent transforming growth factor beta binding protein 1 | MGC163161 | 2 | Antimicrobials |
| DEFB4 | 1673 | defensin, beta 4 | DEFB-2\|DEFB102\|DEFB2\|HBD-2\|SAP1 | 8 | Antimicrobials |
| TNFSF10 | 8743 | tumor necrosis factor (ligand) superfamily, member 10 | APO2L\|Apo-2L\|CD253\|TL2\|TRAIL | 3 | Antimicrobials |
| IL13 | 3596 | interleukin 13 | ALRH\|BHR1\|IL-13\|MGC116786\|MGC116788\|MGC116789\|P600 | 5 | Antimicrobials |
| IL10 | 3586 | interleukin 10 | CSIF\|IL-10\|IL10A\|MGC126450\|MGC126451\|TGIF | 1 | Antimicrobials |
| IL2 | 3558 | interleukin 2 | IL-2\|TCGF\|lymphokine | 4 | Antimicrobials |
| PPARG | 5468 | peroxisome proliferator-activated receptor gamma | CIMT1\|NR1C3\|PPARG1\|PPARG2\|PPARgamma | 3 | Antimicrobials |
| FGR | 2268 | Gardner-Rasheed feline sarcoma viral (v-fgr) oncogene homolog | FLJ43153\|MGC75096\|SRC2\|c-fgr\|c-src2\|p55c-fgr\|p58c-fgr | 1 | Antimicrobials |
| MIF | 4282 | macrophage migration inhibitory factor (glycosylation-inhibiting factor) | GIF\|GLIF\|MMIF | 22 | Antimicrobials |
| CRP | 1401 | C-reactive protein, pentraxin-related | MGC149895\|MGC88244\|PTX1 | 1 | Antimicrobials |
| JAK2 | 3717 | Janus kinase 2 | JTK10 | 9 | Antimicrobials |
| IL1A | 3552 | interleukin 1, alpha | IL-1A\|IL1\|IL1-ALPHA\|IL1F1 | 2 | Antimicrobials |
| PTK2 | 5747 | PTK2 protein tyrosine kinase 2 | FADK\|FAK\|FAK1\|pp125FAK | 8 | Antimicrobials |
| PTGDR | 5729 | prostaglandin D2 receptor (DP) | AS1\|ASRT1\|DP\|DP1\|MGC49004 | 14 | Antimicrobials |
| CD86 | 942 | CD86 molecule | B7-2\|B70\|CD28LG2\|LAB72\|MGC34413 | 3 | Antimicrobials |
| HCK | 3055 | hemopoietic cell kinase | JTK9 | 20 | Antimicrobials |
| ARRB1 | 408 | arrestin, beta 1 | ARB1\|ARR1 | 11 | Antimicrobials |
| GNAI1 | 2770 | guanine nucleotide binding protein (G protein), alpha inhibiting activity polypeptide 1 | Gi | 7 | Antimicrobials |
| VDR | 7421 | vitamin D (1,25- dihydroxyvitamin D3) receptor | NR1I1 | 12 | Antimicrobials |
| OLR1 | 4973 | oxidized low density lipoprotein (lectin-like) receptor 1 | CLEC8A\|LOX1\|SCARE1 | 12 | Antimicrobials |
| ADRBK1 | 156 | adrenergic, beta, receptor kinase 1 | BARK1\|BETA-ARK1\|FLJ16718\|GRK2 | 11 | Antimicrobials |
| TXK | 7294 | TXK tyrosine kinase | BTKL\|MGC22473\|PSCTK5\|PTK4\|RLK\|TKL | 4 | Antimicrobials |
| RNASE2 | 6036 | ribonuclease, RNase A family, 2 (liver, eosinophil-derived neurotoxin) | EDN\|RNS2 | 14 | Antimicrobials |
| CD79A | 973 | CD79a molecule, immunoglobulin-associated alpha | IGA\|MB-1 | 19 | BCRSignalingPathway |
| CD79B | 974 | CD79b molecule, immunoglobulin-associated beta | B29\|IGB | 17 | BCRSignalingPathway |
| LYN | 4067 | v-yes-1 Yamaguchi sarcoma viral related oncogene homolog | FLJ26625\|JTK8 | 8 | BCRSignalingPathway |
| SYK | 6850 | spleen tyrosine kinase | DKFZp313N1010\|FLJ25043\|FLJ37489 | 9 | BCRSignalingPathway |
| BTK | 695 | Bruton agammaglobulinemia tyrosine kinase | AGMX1\|AT\|ATK\|BPK\|IMD1\|MGC126261\|MGC126262\|PSCTK1\|XLA | X | BCRSignalingPathway |
| BLNK | 29760 | B-cell linker | BASH\|BLNK-S\|LY57\|MGC111051\|SLP-65\|SLP65 | 10 | BCRSignalingPathway |
| VAV3 | 10451 | vav 3 guanine nucleotide exchange factor | FLJ40431 | 1 | BCRSignalingPathway |
| VAV1 | 7409 | vav 1 guanine nucleotide exchange factor | VAV | 19 | BCRSignalingPathway |
| VAV2 | 7410 | vav 2 guanine nucleotide exchange factor | - | 9 | BCRSignalingPathway |
| RAC1 | 5879 | ras-related C3 botulinum toxin substrate 1 (rho family, small GTP binding protein Rac1) | MGC111543\|MIG5\|TC-25\|p21-Rac1 | 7 | BCRSignalingPathway |
| RAC2 | 5880 | ras-related C3 botulinum toxin substrate 2 (rho family, small GTP binding protein Rac2) | EN-7\|Gx\|HSPC022 | 22 | BCRSignalingPathway |
| RAC3 | 5881 | ras-related C3 botulinum toxin substrate 3 (rho family, small GTP binding protein Rac3) | - | 17 | BCRSignalingPathway |
| PPP3CA | 5530 | protein phosphatase 3 (formerly 2B), catalytic subunit, alpha isoform | CALN\|CALNA\|CALNA1\|CCN1\|CNA1\|PPP2B | 4 | BCRSignalingPathway |
| PPP3CB | 5532 | protein phosphatase 3 (formerly 2B), catalytic subunit, beta isoform | CALNA2\|CALNB | 10 | BCRSignalingPathway |
| PPP3CC | 5533 | protein phosphatase 3 (formerly 2B), catalytic subunit, gamma isoform | CALNA3 | 8 | BCRSignalingPathway |
| CHP | 11261 | calcium binding protein P22 | SLC9A1BP | 15 | BCRSignalingPathway |
| PPP3R1 | 5534 | protein phosphatase 3 (formerly 2B), regulatory subunit B, alpha isoform | CALNB1\|CNB\|CNB1 | 2 | BCRSignalingPathway |
| PPP3R2 | 5535 | protein phosphatase 3 (formerly 2B), regulatory subunit B, beta isoform | PPP3RL | 9 | BCRSignalingPathway |
| CHP2 | 63928 | calcineurin B homologous protein 2 | - | 16 | BCRSignalingPathway |
| NFAT5 | 10725 | nuclear factor of activated T-cells 5, tonicity-responsive | KIAA0827\|NF-AT5\|NFATL1\|NFATZ\|OREBP\|TONEBP | 16 | BCRSignalingPathway |
| NFATC1 | 4772 | nuclear factor of activated T-cells, cytoplasmic, calcineurin-dependent 1 | MGC138448\|NF-ATC\|NFAT2\|NFATc | 18 | BCRSignalingPathway |
| NFATC2 | 4773 | nuclear factor of activated T-cells, cytoplasmic, calcineurin-dependent 2 | NFAT1\|NFATP | 20 | BCRSignalingPathway |
| NFATC3 | 4775 | nuclear factor of activated T-cells, cytoplasmic, calcineurin-dependent 3 | NFAT4\|NFATX | 16 | BCRSignalingPathway |
| NFATC4 | 4776 | nuclear factor of activated T-cells, cytoplasmic, calcineurin-dependent 4 | NF-ATc4\|NFAT3 | 14 | BCRSignalingPathway |
| HRAS | 3265 | v-Ha-ras Harvey rat sarcoma viral oncogene homolog | C-BAS/HAS\|C-H-RAS\|C-HA-RAS1\|CTLO\|H-RASIDX\|HAMSV\|HRAS1\|K-RAS\|N-RAS\|RASH1 | 11 | BCRSignalingPathway |
| KRAS | 3845 | v-Ki-ras2 Kirsten rat sarcoma viral oncogene homolog | C-K-RAS\|K-RAS2A\|K-RAS2B\|K-RAS4A\|K-RAS4B\|KI-RAS\|KRAS1\|KRAS2\|NS3\|RASK2 | 12 | BCRSignalingPathway |
| NRAS | 4893 | neuroblastoma RAS viral (v-ras) oncogene homolog | ALPS4\|N-ras\|NRAS1 | 1 | BCRSignalingPathway |
| FOS | 2353 | v-fos FBJ murine osteosarcoma viral oncogene homolog | AP-1\|C-FOS | 14 | BCRSignalingPathway |
| JUN | 3725 | jun oncogene | AP-1\|AP1\|c-Jun | 1 | BCRSignalingPathway |
| CARD11 | 84433 | caspase recruitment domain family, member 11 | BIMP3\|CARMA1\|MGC133069 | 7 | BCRSignalingPathway |
| BCL10 | 8915 | B-cell CLL/lymphoma 10 | CARMEN\|CIPER\|CLAP\|c-E10\|mE10 | 1 | BCRSignalingPathway |
| MALT1 | 10892 | mucosa associated lymphoid tissue lymphoma translocation gene 1 | DKFZp434L132\|MLT\|MLT1 | 18 | BCRSignalingPathway |
| CHUK | 1147 | conserved helix-loop-helix ubiquitous kinase | IKBKA\|IKK-alpha\|IKK1\|IKKA\|NFKBIKA\|TCF16 | 10 | BCRSignalingPathway |
| IKBKB | 3551 | inhibitor of kappa light polypeptide gene enhancer in B-cells, kinase beta | FLJ40509\|IKK-beta\|IKK2\|IKKB\|MGC131801\|NFKBIKB | 8 | BCRSignalingPathway |
| IKBKG | 8517 | inhibitor of kappa light polypeptide gene enhancer in B-cells, kinase gamma | AMCBX1\|FIP-3\|FIP3\|Fip3p\|IKK-gamma\|IP\|IP1\|IP2\|IPD2\|NEMO | X | BCRSignalingPathway |
| NFKB1 | 4790 | nuclear factor of kappa light polypeptide gene enhancer in B-cells 1 | DKFZp686C01211\|EBP-1\|KBF1\|MGC54151\|NF-kappa-B\|NFKB-p105\|NFKB-p50\|p105\|p50 | 4 | BCRSignalingPathway |
| RELA | 5970 | v-rel reticuloendotheliosis viral oncogene homolog A (avian) | MGC131774\|NFKB3\|p65 | 11 | BCRSignalingPathway |
| NFKBIA | 4792 | nuclear factor of kappa light polypeptide gene enhancer in B-cells inhibitor, alpha | IKBA\|MAD-3\|NFKBI | 14 | BCRSignalingPathway |
| NFKBIB | 4793 | nuclear factor of kappa light polypeptide gene enhancer in B-cells inhibitor, beta | IKBB\|TRIP9 | 19 | BCRSignalingPathway |
| NFKBIE | 4794 | nuclear factor of kappa light polypeptide gene enhancer in B-cells inhibitor, epsilon | IKBE | 6 | BCRSignalingPathway |
| CD81 | 975 | CD81 molecule | S5.7\|TAPA1\|TSPAN28 | 11 | BCRSignalingPathway |
| CD19 | 930 | CD19 molecule | B4\|MGC12802 | 16 | BCRSignalingPathway |
| CR2 | 1380 | complement component (3d/Epstein Barr virus) receptor 2 | C3DR\|CD21\|SLEB9 | 1 | BCRSignalingPathway |
| PIK3R5 | 23533 | phosphoinositide-3-kinase, regulatory subunit 5 | F730038I15Rik\|FOAP-2\|P101-PI3K\|p101 | 17 | BCRSignalingPathway |
| PIK3R1 | 5295 | phosphoinositide-3-kinase, regulatory subunit 1 (alpha) | GRB1\|p85\|p85-ALPHA | 5 | BCRSignalingPathway |
| PIK3R2 | 5296 | phosphoinositide-3-kinase, regulatory subunit 2 (beta) | P85B\|p85\|p85-BETA | 19 | BCRSignalingPathway |
| PIK3R3 | 8503 | phosphoinositide-3-kinase, regulatory subunit 3 (gamma) | DKFZp686P05226\|FLJ41892\|p55\|p55-GAMMA | 1 | BCRSignalingPathway |
| PIK3CA | 5290 | phosphoinositide-3-kinase, catalytic, alpha polypeptide | MGC142161\|MGC142163\|PI3K\|p110-alpha | 3 | BCRSignalingPathway |
| PIK3CB | 5291 | phosphoinositide-3-kinase, catalytic, beta polypeptide | DKFZp779K1237\|MGC133043\|PI3K\|PI3KCB\|PI3Kbeta\|PIK3C1\|p110-BETA | 3 | BCRSignalingPathway |
| PIK3CD | 5293 | phosphoinositide-3-kinase, catalytic, delta polypeptide | p110D | 1 | BCRSignalingPathway |
| PIK3CG | 5294 | phosphoinositide-3-kinase, catalytic, gamma polypeptide | PI3CG\|PI3K\|PI3Kgamma\|PIK3 | 7 | BCRSignalingPathway |
| AKT3 | 10000 | v-akt murine thymoma viral oncogene homolog 3 (protein kinase B, gamma) | DKFZp434N0250\|PKB-GAMMA\|PKBG\|PRKBG\|RAC-PK-gamma\|RAC-gamma\|STK-2 | 1 | BCRSignalingPathway |
| AKT1 | 207 | v-akt murine thymoma viral oncogene homolog 1 | AKT\|MGC99656\|PKB\|PKB-ALPHA\|PRKBA\|RAC\|RAC-ALPHA | 14 | BCRSignalingPathway |
| AKT2 | 208 | v-akt murine thymoma viral oncogene homolog 2 | PKBB\|PKBBETA\|PRKBB\|RAC-BETA | 19 | BCRSignalingPathway |
| GSK3B | 2932 | glycogen synthase kinase 3 beta | - | 3 | BCRSignalingPathway |
| INPP5D | 3635 | inositol polyphosphate-5-phosphatase, 145kDa | MGC104855\|MGC142140\|MGC142142\|SHIP\|SHIP1\|SIP-145\|hp51CN | 2 | BCRSignalingPathway |
| CD22 | 933 | CD22 molecule | FLJ22814\|MGC130020\|SIGLEC-2\|SIGLEC2 | 19 | BCRSignalingPathway |
| CD72 | 971 | CD72 molecule | CD72b\|LYB2 | 9 | BCRSignalingPathway |
| PTPN6 | 5777 | protein tyrosine phosphatase, non-receptor type 6 | HCP\|HCPH\|HPTP1C\|PTP-1C\|SH-PTP1\|SHP-1\|SHP-1L\|SHP1 | 12 | BCRSignalingPathway |
| LILRB3 | 11025 | leukocyte immunoglobulin-like receptor, subfamily B (with TM and ITIM domains), member 3 | CD85A\|HL9\|ILT5\|LIR-3\|LIR3\|MGC138403\|PIRB | 19 | BCRSignalingPathway |
| FCGR2B | 2213 | Fc fragment of IgG, low affinity IIb, receptor (CD32) | CD32\|CD32B\|FCG2\|FCGR2\|IGFR2 | 1 | BCRSignalingPathway |
| RASGRP3 | 25780 | RAS guanyl releasing protein 3 (calcium and DAG-regulated) | GRP3\|KIAA0846 | 2 | BCRSignalingPathway |
| PLCG2 | 5336 | phospholipase C, gamma 2 (phosphatidylinositol-specific) | - | 16 | BCRSignalingPathway |
| PRKCB | 5579 | protein kinase C, beta | MGC41878\|PKC-beta\|PKCB\|PRKCB1\|PRKCB2 | 16 | BCRSignalingPathway |
| IFITM1 | 8519 | interferon induced transmembrane protein 1 (9-27) | 9-27\|CD225\|IFI17\|LEU13 | 11 | BCRSignalingPathway |
| IGH@ | 3492 | immunoglobulin heavy locus | DKFZp686C15213\|IGH\|IGH.1@\|IGHDY1\|MGC72071\|MGC88774 | 14 | BCRSignalingPathway |
| IGHA1 | 3493 | immunoglobulin heavy constant alpha 1 | FLJ14473\|FLJ35065\|FLJ35500\|FLJ36402\|FLJ39698\|FLJ40001\|FLJ41548\|FLJ41552\|FLJ41789\|FLJ43248\|FLJ43594\|FLJ44293\|FLJ46028\|FLJ46621\|FLJ46724\|FLJ46811\|FLJ46824\|FLJ90170\|IgA1\|MGC102857 | 14 | BCRSignalingPathway |
| IGHA2 | 3494 | immunoglobulin heavy constant alpha 2 (A2m marker) | - | 14 | BCRSignalingPathway |
| IGHD | 3495 | immunoglobulin heavy constant delta | FLJ00382\|FLJ46727\|MGC29633 | 14 | BCRSignalingPathway |
| IGHD@ | 50648 | immunoglobulin heavy diversity group | IGD1\|IGHDY1 | 14 | BCRSignalingPathway |
| IGHD1-1 | 28510 | immunoglobulin heavy diversity 1-1 | IGHD11 | 14 | BCRSignalingPathway |
| IGHD1-14 | 28508 | immunoglobulin heavy diversity 1-14 (non-functional) | DM2\|IGHD114 | 14 | BCRSignalingPathway |
| IGHD1-20 | 28507 | immunoglobulin heavy diversity 1-20 | IGHD120 | 14 | BCRSignalingPathway |
| IGHD1-26 | 28506 | immunoglobulin heavy diversity 1-26 | IGHD126 | 14 | BCRSignalingPathway |
| IGHD1-7 | 28509 | immunoglobulin heavy diversity 1-7 | DM1\|IGHD17 | 14 | BCRSignalingPathway |
| IGHD2-15 | 28503 | immunoglobulin heavy diversity 2-15 | D2\|IGHD215 | 14 | BCRSignalingPathway |
| IGHD2-2 | 28505 | immunoglobulin heavy diversity 2-2 | IGHD22 | 14 | BCRSignalingPathway |
| IGHD2-21 | 28502 | immunoglobulin heavy diversity 2-21 | IGHD221 | 14 | BCRSignalingPathway |
| IGHD2-8 | 28504 | immunoglobulin heavy diversity 2-8 | DLR1\|IGHD28 | 14 | BCRSignalingPathway |
| IGHD3-10 | 28499 | immunoglobulin heavy diversity 3-10 | DXP'1\|IGHD310 | 14 | BCRSignalingPathway |
| IGHD3-16 | 28498 | immunoglobulin heavy diversity 3-16 | IGHD316 | 14 | BCRSignalingPathway |
| IGHD3-22 | 28497 | immunoglobulin heavy diversity 3-22 | IGHD322 | 14 | BCRSignalingPathway |
| IGHD3-3 | 28501 | immunoglobulin heavy diversity 3-3 | DXP4\|IGHD33 | 14 | BCRSignalingPathway |
| IGHD3-9 | 28500 | immunoglobulin heavy diversity 3-9 | DXP1\|IGHD39 | 14 | BCRSignalingPathway |
| IGHD4-11 | 28495 | immunoglobulin heavy diversity 4-11 (non-functional) | DA1\|IGHD411 | 14 | BCRSignalingPathway |
| IGHD4-17 | 28494 | immunoglobulin heavy diversity 4-17 | IGHD417 | 14 | BCRSignalingPathway |
| IGHD4-23 | 28493 | immunoglobulin heavy diversity 4-23 (non-functional) | IGHD423 | 14 | BCRSignalingPathway |
| IGHD4-4 | 28496 | immunoglobulin heavy diversity 4-4 | DA4\|IGHD44 | 14 | BCRSignalingPathway |
| IGHD5-12 | 28491 | immunoglobulin heavy diversity 5-12 | DK1\|IGHD512 | 14 | BCRSignalingPathway |
| IGHD5-18 | 28490 | immunoglobulin heavy diversity 5-18 | IGHD518 | 14 | BCRSignalingPathway |
| IGHD5-24 | 28489 | immunoglobulin heavy diversity 5-24 (non-functional) | IGHD524 | 14 | BCRSignalingPathway |
| IGHD5-5 | 28492 | immunoglobulin heavy diversity 5-5 | DK4\|IGHD55 | 14 | BCRSignalingPathway |
| IGHD6-13 | 28487 | immunoglobulin heavy diversity 6-13 | DN1\|IGHD613 | 14 | BCRSignalingPathway |
| IGHD6-19 | 28486 | immunoglobulin heavy diversity 6-19 | IGHD619 | 14 | BCRSignalingPathway |
| IGHD6-25 | 28485 | immunoglobulin heavy diversity 6-25 | IGHD625 | 14 | BCRSignalingPathway |
| IGHD6-6 | 28488 | immunoglobulin heavy diversity 6-6 | D(N4)\|IGHD66 | 14 | BCRSignalingPathway |
| IGHD7-27 | 28484 | immunoglobulin heavy diversity 7-27 | DHQ52\|IGHD727 | 14 | BCRSignalingPathway |
| IGHE | 3497 | immunoglobulin heavy constant epsilon | IgE | 14 | BCRSignalingPathway |
| IGHG1 | 3500 | immunoglobulin heavy constant gamma 1 (G1m marker) | - | 14 | BCRSignalingPathway |
| IGHG2 | 3501 | immunoglobulin heavy constant gamma 2 (G2m marker) | DKFZp686I04196 | 14 | BCRSignalingPathway |
| IGHG3 | 3502 | immunoglobulin heavy constant gamma 3 (G3m marker) | DKFZp686H11213\|FLJ39988\|FLJ40036\|FLJ40253\|FLJ40587\|FLJ40789\|FLJ40834\|IgG3\|MGC45809 | 14 | BCRSignalingPathway |
| IGHG4 | 3503 | immunoglobulin heavy constant gamma 4 (G4m marker) | MGC117419 | 14 | BCRSignalingPathway |
| IGHJ@ | 3506 | immunoglobulin heavy joining group | IGHJ | 14 | BCRSignalingPathway |
| IGHJ1 | 28483 | immunoglobulin heavy joining 1 | JH1 | 14 | BCRSignalingPathway |
| IGHJ2 | 28481 | immunoglobulin heavy joining 2 | JH2 | 14 | BCRSignalingPathway |
| IGHJ3 | 28479 | immunoglobulin heavy joining 3 | JH3b | 14 | BCRSignalingPathway |
| IGHJ4 | 28477 | immunoglobulin heavy joining 4 | JH4b | 14 | BCRSignalingPathway |
| IGHJ5 | 28476 | immunoglobulin heavy joining 5 | JH5b | 14 | BCRSignalingPathway |
| IGHJ6 | 28475 | immunoglobulin heavy joining 6 | JH6b | 14 | BCRSignalingPathway |
| IGHM | 3507 | immunoglobulin heavy constant mu | DKFZp686I15196\|DKFZp686I15212\|FLJ00385\|MGC104996\|MGC52291\|MU\|VH | 14 | BCRSignalingPathway |
| IGHV@ | 3509 | immunoglobulin heavy variable group | IGHV | 14 | BCRSignalingPathway |
| IGHV1-18 | 28468 | immunoglobulin heavy variable 1-18 | IGHV118 | 14 | BCRSignalingPathway |
| IGHV1-2 | 28474 | immunoglobulin heavy variable 1-2 | IGHV12 | 14 | BCRSignalingPathway |
| IGHV1-24 | 28467 | immunoglobulin heavy variable 1-24 | IGHV124\|VH | 14 | BCRSignalingPathway |
| IGHV1-3 | 28473 | immunoglobulin heavy variable 1-3 | IGHV13 | 14 | BCRSignalingPathway |
| IGHV1-45 | 28466 | immunoglobulin heavy variable 1-45 | IGHV145\|VH | 14 | BCRSignalingPathway |
| IGHV1-46 | 28465 | immunoglobulin heavy variable 1-46 | IGHV146 | 14 | BCRSignalingPathway |
| IGHV1-58 | 28464 | immunoglobulin heavy variable 1-58 | IGHV158\|VH | 14 | BCRSignalingPathway |
| IGHV1-69 | 28461 | immunoglobulin heavy variable 1-69 | IGHV1-E\|IGHV169\|IGHV1E | 14 | BCRSignalingPathway |
| IGHV1-8 | 28472 | immunoglobulin heavy variable 1-8 | IGHV18 | 14 | BCRSignalingPathway |
| IGHV1-C | 28460 | immunoglobulin heavy variable 1-C (provisional, non-functional) | IGHV1C | 14 | BCRSignalingPathway |
| IGHV1-F | 28458 | immunoglobulin heavy variable 1-F (provisional) | IGHV1F | 14 | BCRSignalingPathway |
| IGHV2-26 | 28455 | immunoglobulin heavy variable 2-26 | IGHV226\|VH | 14 | BCRSignalingPathway |
| IGHV2-5 | 28457 | immunoglobulin heavy variable 2-5 | IGHV25\|VH | 14 | BCRSignalingPathway |
| IGHV2-70 | 28454 | immunoglobulin heavy variable 2-70 | IGHV270\|VH | 14 | BCRSignalingPathway |
| IGHV3-11 | 28450 | immunoglobulin heavy variable 3-11 (gene/pseudogene) | IGHV311\|VH | 14 | BCRSignalingPathway |
| IGHV3-13 | 28449 | immunoglobulin heavy variable 3-13 | IGHV313 | 14 | BCRSignalingPathway |
| IGHV3-15 | 28448 | immunoglobulin heavy variable 3-15 | IGHV315\|VH | 14 | BCRSignalingPathway |
| IGHV3-16 | 28447 | immunoglobulin heavy variable 3-16 (non-functional) | IGHV316\|VH | 14 | BCRSignalingPathway |
| IGHV3-20 | 28445 | immunoglobulin heavy variable 3-20 | IGHV320\|VH | 14 | BCRSignalingPathway |
| IGHV3-21 | 28444 | immunoglobulin heavy variable 3-21 | IGHV321\|VH | 14 | BCRSignalingPathway |
| IGHV3-23 | 28442 | immunoglobulin heavy variable 3-23 | DP47\|IGHV323\|V3-23\|VH26 | 14 | BCRSignalingPathway |
| IGHV3-30 | 28439 | immunoglobulin heavy variable 3-30 | IGHV330\|VH | 14 | BCRSignalingPathway |
| IGHV3-30-3 | 57290 | immunoglobulin heavy variable 3-30-3 | IGHV3-3\|IGHV3303 | 14 | BCRSignalingPathway |
| IGHV3-30-5 | 89770 | immunoglobulin heavy variable 3-30-5 | IGHV3-3\|IGHV3305 | 14 | BCRSignalingPathway |
| IGHV3-33 | 28434 | immunoglobulin heavy variable 3-33 | IGHV333\|VH | 14 | BCRSignalingPathway |
| IGHV3-35 | 28432 | immunoglobulin heavy variable 3-35 (non-functional) | IGHV335\|VH | 14 | BCRSignalingPathway |
| IGHV3-38 | 28429 | immunoglobulin heavy variable 3-38 (non-functional) | IGHV338\|VH | 14 | BCRSignalingPathway |
| IGHV3-43 | 28426 | immunoglobulin heavy variable 3-43 | IGHV343\|VH | 14 | BCRSignalingPathway |
| IGHV3-48 | 28424 | immunoglobulin heavy variable 3-48 | IGHV348\|VH | 14 | BCRSignalingPathway |
| IGHV3-49 | 28423 | immunoglobulin heavy variable 3-49 | IGHV349\|VH | 14 | BCRSignalingPathway |
| IGHV3-53 | 28420 | immunoglobulin heavy variable 3-53 | IGHV353\|VH | 14 | BCRSignalingPathway |
| IGHV3-64 | 28414 | immunoglobulin heavy variable 3-64 | IGHV364\|VH | 14 | BCRSignalingPathway |
| IGHV3-66 | 28412 | immunoglobulin heavy variable 3-66 | IGHV366\|VH | 14 | BCRSignalingPathway |
| IGHV3-7 | 28452 | immunoglobulin heavy variable 3-7 | IGHV37\|VH | 14 | BCRSignalingPathway |
| IGHV3-72 | 28410 | immunoglobulin heavy variable 3-72 | IGHV372\|VH | 14 | BCRSignalingPathway |
| IGHV3-73 | 28409 | immunoglobulin heavy variable 3-73 | IGHV373\|VH | 14 | BCRSignalingPathway |
| IGHV3-74 | 28408 | immunoglobulin heavy variable 3-74 | IGHV374\|VH | 14 | BCRSignalingPathway |
| IGHV3-9 | 28451 | immunoglobulin heavy variable 3-9 | IGHV39\|VH | 14 | BCRSignalingPathway |
| IGHV3-D | 28404 | immunoglobulin heavy variable 3-D (provisional) | IGHV3D | 14 | BCRSignalingPathway |
| IGHV3-H | 28402 | immunoglobulin heavy variable 3-H pseudogene (provisional) | IGHV3H | 14 | BCRSignalingPathway |
| IGHV4-28 | 28400 | immunoglobulin heavy variable 4-28 | IGHV428\|VH | 14 | BCRSignalingPathway |
| IGHV4-30-1 | 28399 | immunoglobulin heavy variable 4-30-1 | IGHV4-3 | 14 | BCRSignalingPathway |
| IGHV4-30-2 | 28398 | immunoglobulin heavy variable 4-30-2 | IGHV4-3\|IGHV4302 | 14 | BCRSignalingPathway |
| IGHV4-30-4 | 28397 | immunoglobulin heavy variable 4-30-4 | IGHV4-3\|IGHV4304 | 14 | BCRSignalingPathway |
| IGHV4-31 | 28396 | immunoglobulin heavy variable 4-31 | FLJ45507\|IGHV431 | 14 | BCRSignalingPathway |
| IGHV4-34 | 28395 | immunoglobulin heavy variable 4-34 | IGHV434\|VH | 14 | BCRSignalingPathway |
| IGHV4-39 | 28394 | immunoglobulin heavy variable 4-39 | IGHV439\|VH | 14 | BCRSignalingPathway |
| IGHV4-4 | 28401 | immunoglobulin heavy variable 4-4 | IGHV44\|VH | 14 | BCRSignalingPathway |
| IGHV4-59 | 28392 | immunoglobulin heavy variable 4-59 | IGHV459\|VH | 14 | BCRSignalingPathway |
| IGHV4-61 | 28391 | immunoglobulin heavy variable 4-61 | IGHV461\|VH | 14 | BCRSignalingPathway |
| IGHV4-B | 28389 | immunoglobulin heavy variable 4-B (provisional) | IGHV4B | 14 | BCRSignalingPathway |
| IGHV5-51 | 28388 | immunoglobulin heavy variable 5-51 | IGHV551\|VH | 14 | BCRSignalingPathway |
| IGHV5-A | 28386 | immunoglobulin heavy variable 5-A (provisional, gene/pseudogene) | IGHV5A | 14 | BCRSignalingPathway |
| IGHV6-1 | 28385 | immunoglobulin heavy variable 6-1 | IGHV61\|VH | 14 | BCRSignalingPathway |
| IGHV7-4-1 | 57289 | immunoglobulin heavy variable 7-4-1 | IGHV7-41\|IGHV741 | 14 | BCRSignalingPathway |
| IGHV7-81 | 28378 | immunoglobulin heavy variable 7-81 (non-functional) | IGHV781 | 14 | BCRSignalingPathway |
| IGK@ | 50802 | immunoglobulin kappa locus | FLJ26296\|IGK\|IGKC\|MGC22645\|MGC27376\|MGC40426\|MGC71990 | 2 | BCRSignalingPathway |
| IGKC | 3514 | immunoglobulin kappa constant | HCAK1\|Km\|MGC111575\|MGC62011\|MGC72072\|MGC88770\|MGC88771\|MGC88809 | 2 | BCRSignalingPathway |
| IGKDEL | 3515 | immunoglobulin kappa deleting element or like | IGKDE | 2 | BCRSignalingPathway |
| IGKJ@ | 7842 | immunoglobulin kappa joining group | IGKJ | 2 | BCRSignalingPathway |
| IGKJ1 | 28950 | immunoglobulin kappa joining 1 | J1 | 2 | BCRSignalingPathway |
| IGKJ2 | 28949 | immunoglobulin kappa joining 2 | J2 | 2 | BCRSignalingPathway |
| IGKJ3 | 28948 | immunoglobulin kappa joining 3 | J3 | 2 | BCRSignalingPathway |
| IGKJ4 | 28947 | immunoglobulin kappa joining 4 | J4 | 2 | BCRSignalingPathway |
| IGKJ5 | 28946 | immunoglobulin kappa joining 5 | J5 | 2 | BCRSignalingPathway |
| IGKV@ | 3519 | immunoglobulin kappa variable group | IGKV\|IGKV1\|IGKV1@\|IGKV2\|IGKV2@\|IGKV3\|IGKV3@ | 2 | BCRSignalingPathway |
| IGKV1-12 | 28940 | immunoglobulin kappa variable 1-12 | IGKV112\|L19 | 2 | BCRSignalingPathway |
| IGKV1-13 | 28939 | immunoglobulin kappa variable 1-13 (gene/pseudogene) | IGKV113\|L18 | 2 | BCRSignalingPathway |
| IGKV1-16 | 28938 | immunoglobulin kappa variable 1-16 | IGKV116\|L1 | 2 | BCRSignalingPathway |
| IGKV1-17 | 28937 | immunoglobulin kappa variable 1-17 | A30\|IGKV117 | 2 | BCRSignalingPathway |
| IGKV1-27 | 28935 | immunoglobulin kappa variable 1-27 | A20\|IGKV127 | 2 | BCRSignalingPathway |
| IGKV1-33 | 28933 | immunoglobulin kappa variable 1-33 | IGKV133\|O18 | 2 | BCRSignalingPathway |
| IGKV1-37 | 28931 | immunoglobulin kappa variable 1-37 (non-functional) | IGKV137\|O14 | 2 | BCRSignalingPathway |
| IGKV1-39 | 28930 | immunoglobulin kappa variable 1-39 (gene/pseudogene) | IGKV139\|O12\|O12a | 2 | BCRSignalingPathway |
| IGKV1-5 | 28299 | immunoglobulin kappa variable 1-5 | IGKV\|IGKV15\|L12\|L12a\|MGC22745\|MGC32715\|MGC88810\|V1 | 2 | BCRSignalingPathway |
| IGKV1-6 | 28943 | immunoglobulin kappa variable 1-6 | IGKV16\|L11 | 2 | BCRSignalingPathway |
| IGKV1-8 | 28942 | immunoglobulin kappa variable 1-8 | IGKV18\|L9 | 2 | BCRSignalingPathway |
| IGKV1-9 | 28941 | immunoglobulin kappa variable 1-9 | IGKV19\|L8 | 2 | BCRSignalingPathway |
| IGKV1D-12 | 28903 | immunoglobulin kappa variable 1D-12 | IGKV1D12\|L19 | 2 | BCRSignalingPathway |
| IGKV1D-13 | 28902 | immunoglobulin kappa variable 1D-13 | IGKV1D13\|L18 | 2 | BCRSignalingPathway |
| IGKV1D-16 | 28901 | immunoglobulin kappa variable 1D-16 | IGKV1D16\|L15\|L15a | 2 | BCRSignalingPathway |
| IGKV1D-17 | 28900 | immunoglobulin kappa variable 1D-17 | IGKV1D17\|L14 | 2 | BCRSignalingPathway |
| IGKV1D-33 | 28896 | immunoglobulin kappa variable 1D-33 | IGKV1D33\|O8 | 2 | BCRSignalingPathway |
| IGKV1D-37 | 28894 | immunoglobulin kappa variable 1D-37 (non-functional) | IGKV1D37\|O4 | 2 | BCRSignalingPathway |
| IGKV1D-39 | 28893 | immunoglobulin kappa variable 1D-39 | IGKV1D39\|O2 | 2 | BCRSignalingPathway |
| IGKV1D-42 | 28892 | immunoglobulin kappa variable 1D-42 (non-functional) | IGKV1D42\|L22 | 2 | BCRSignalingPathway |
| IGKV1D-43 | 28891 | immunoglobulin kappa variable 1D-43 | IGKV1D43\|L23\|L23a | 2 | BCRSignalingPathway |
| IGKV1D-8 | 28904 | immunoglobulin kappa variable 1D-8 | IGKV1D8\|L24\|L24a | 2 | BCRSignalingPathway |
| IGKV2-24 | 28923 | immunoglobulin kappa variable 2-24 | A23\|IGKV224 | 2 | BCRSignalingPathway |
| IGKV2-28 | 28921 | immunoglobulin kappa variable 2-28 | A19\|IGKV228 | 2 | BCRSignalingPathway |
| IGKV2-30 | 28919 | immunoglobulin kappa variable 2-30 | A17\|IGKV230 | 2 | BCRSignalingPathway |
| IGKV2-40 | 28916 | immunoglobulin kappa variable 2-40 | IGKV240\|O11\|O11a | 2 | BCRSignalingPathway |
| IGKV2D-24 | 28885 | immunoglobulin kappa variable 2D-24 (non-functional) | A7\|IGKV2D24 | 2 | BCRSignalingPathway |
| IGKV2D-28 | 28883 | immunoglobulin kappa variable 2D-28 | A3\|IGKV2D28 | 2 | BCRSignalingPathway |
| IGKV2D-29 | 28882 | immunoglobulin kappa variable 2D-29 | A2a\|A2c\|IGKV2D29 | 2 | BCRSignalingPathway |
| IGKV2D-30 | 28881 | immunoglobulin kappa variable 2D-30 | A1\|IGKV2D30 | 2 | BCRSignalingPathway |
| IGKV2D-40 | 28878 | immunoglobulin kappa variable 2D-40 | IGKV2D40\|O1 | 2 | BCRSignalingPathway |
| IGKV3-11 | 28914 | immunoglobulin kappa variable 3-11 | IGKV311\|L6 | 2 | BCRSignalingPathway |
| IGKV3-15 | 28913 | immunoglobulin kappa variable 3-15 | IGKV315\|L2 | 2 | BCRSignalingPathway |
| IGKV3-20 | 28912 | immunoglobulin kappa variable 3-20 | 13K18\|A27\|IGKV320 | 2 | BCRSignalingPathway |
| IGKV3-7 | 28915 | immunoglobulin kappa variable 3-7 (non-functional) | IGKV37\|L10\|L10a\|Vh | 2 | BCRSignalingPathway |
| IGKV3D-11 | 28876 | immunoglobulin kappa variable 3D-11 | IGKV3D11\|L20 | 2 | BCRSignalingPathway |
| IGKV3D-15 | 28875 | immunoglobulin kappa variable 3D-15 (gene/pseudogene) | IGKV3D15\|L16\|L16a\|L16b\|L16c | 2 | BCRSignalingPathway |
| IGKV3D-20 | 28874 | immunoglobulin kappa variable 3D-20 | A11\|A11a\|IGKV3D20 | 2 | BCRSignalingPathway |
| IGKV3D-7 | 28877 | immunoglobulin kappa variable 3D-7 | IGKV3D7\|L25 | 2 | BCRSignalingPathway |
| IGKV4-1 | 28908 | immunoglobulin kappa variable 4-1 | B3\|IGKV41 | 2 | BCRSignalingPathway |
| IGKV5-2 | 28907 | immunoglobulin kappa variable 5-2 | B2\|IGKV52 | 2 | BCRSignalingPathway |
| IGKV6-21 | 28906 | immunoglobulin kappa variable 6-21 (non-functional) | A26\|IGKV621 | 2 | BCRSignalingPathway |
| IGKV6D-21 | 28870 | immunoglobulin kappa variable 6D-21 (non-functional) | A10\|IGKV6D21 | 2 | BCRSignalingPathway |
| IGKV6D-41 | 28869 | immunoglobulin kappa variable 6D-41 (non-functional) | A14 | 2 | BCRSignalingPathway |
| IGL@ | 3535 | immunoglobulin lambda locus | IGL\|MGC88804 | 22 | BCRSignalingPathway |
| IGLC@ | 3536 | immunoglobulin lambda constant group | IGLC | 22 | BCRSignalingPathway |
| IGLC1 | 3537 | immunoglobulin lambda constant 1 (Mcg marker) | IGLC | 22 | BCRSignalingPathway |
| IGLC2 | 3538 | immunoglobulin lambda constant 2 (Kern-Oz- marker) | IGLC\|MGC20392\|MGC45681 | 22 | BCRSignalingPathway |
| IGLC3 | 3539 | immunoglobulin lambda constant 3 (Kern-Oz+ marker) | IGLC | 22 | BCRSignalingPathway |
| IGLC6 | 3542 | immunoglobulin lambda constant 6 (Kern+Oz- marker, gene/pseudogene) | IGLC | 22 | BCRSignalingPathway |
| IGLC7 | 28834 | immunoglobulin lambda constant 7 | C7 | 22 | BCRSignalingPathway |
| IGLJ@ | 8217 | immunoglobulin lambda joining group | IGLJ | 22 | BCRSignalingPathway |
| IGLJ1 | 28833 | immunoglobulin lambda joining 1 | J1 | 22 | BCRSignalingPathway |
| IGLJ2 | 28832 | immunoglobulin lambda joining 2 | J2 | 22 | BCRSignalingPathway |
| IGLJ3 | 28831 | immunoglobulin lambda joining 3 | J3 | 22 | BCRSignalingPathway |
| IGLJ4 | 28830 | immunoglobulin lambda joining 4 (non-functional) | - | 22 | BCRSignalingPathway |
| IGLJ5 | 28829 | immunoglobulin lambda joining 5 (non-functional) | - | 22 | BCRSignalingPathway |
| IGLJ6 | 28828 | immunoglobulin lambda joining 6 | - | 22 | BCRSignalingPathway |
| IGLJ7 | 28827 | immunoglobulin lambda joining 7 | J7 | 22 | BCRSignalingPathway |
| IGLV@ | 3546 | immunoglobulin lambda variable group | IGLV | 22 | BCRSignalingPathway |
| IGLV1-36 | 28826 | immunoglobulin lambda variable 1-36 | IGLV136\|V1-11 | 22 | BCRSignalingPathway |
| IGLV1-40 | 28825 | immunoglobulin lambda variable 1-40 | IGLV140\|V1-13 | 22 | BCRSignalingPathway |
| IGLV1-44 | 28823 | immunoglobulin lambda variable 1-44 | IGLV144\|V1-16 | 22 | BCRSignalingPathway |
| IGLV1-47 | 28822 | immunoglobulin lambda variable 1-47 | IGLV147\|V1-17 | 22 | BCRSignalingPathway |
| IGLV1-50 | 28821 | immunoglobulin lambda variable 1-50 (non-functional) | IGLV150\|V1-18 | 22 | BCRSignalingPathway |
| IGLV1-51 | 28820 | immunoglobulin lambda variable 1-51 | IGLV151\|V1-19 | 22 | BCRSignalingPathway |
| IGLV10-54 | 28772 | immunoglobulin lambda variable 10-54 | IGLV1054\|V1-20 | 22 | BCRSignalingPathway |
| IGLV11-55 | 28770 | immunoglobulin lambda variable 11-55 (non-functional) | IGLV1155\|V4-6 | 22 | BCRSignalingPathway |
| IGLV2-11 | 28816 | immunoglobulin lambda variable 2-11 | IGLV211\|V1-3 | 22 | BCRSignalingPathway |
| IGLV2-14 | 28815 | immunoglobulin lambda variable 2-14 | IGLV214\|V1-4 | 22 | BCRSignalingPathway |
| IGLV2-18 | 28814 | immunoglobulin lambda variable 2-18 | IGLV218\|V1-5 | 22 | BCRSignalingPathway |
| IGLV2-23 | 28813 | immunoglobulin lambda variable 2-23 | IGLV223\|V1-7 | 22 | BCRSignalingPathway |
| IGLV2-33 | 28811 | immunoglobulin lambda variable 2-33 (non-functional) | IGLV233\|V1-9 | 22 | BCRSignalingPathway |
| IGLV2-8 | 28817 | immunoglobulin lambda variable 2-8 | IGLV28\|V1-2 | 22 | BCRSignalingPathway |
| IGLV3-1 | 28809 | immunoglobulin lambda variable 3-1 | IGLV31\|V2-1 | 22 | BCRSignalingPathway |
| IGLV3-10 | 28803 | immunoglobulin lambda variable 3-10 | IGLV310\|V2-7 | 22 | BCRSignalingPathway |
| IGLV3-12 | 28802 | immunoglobulin lambda variable 3-12 | IGLV312\|V2-8 | 22 | BCRSignalingPathway |
| IGLV3-16 | 28799 | immunoglobulin lambda variable 3-16 | IGLV316\|V2-11 | 22 | BCRSignalingPathway |
| IGLV3-19 | 28797 | immunoglobulin lambda variable 3-19 | IGLV319\|V2-13\|VL3L | 22 | BCRSignalingPathway |
| IGLV3-21 | 28796 | immunoglobulin lambda variable 3-21 | IGLV321\|V2-14 | 22 | BCRSignalingPathway |
| IGLV3-22 | 28795 | immunoglobulin lambda variable 3-22 (gene/pseudogene) | IGLV322\|V2-15 | 22 | BCRSignalingPathway |
| IGLV3-25 | 28793 | immunoglobulin lambda variable 3-25 | IGLV325\|MGC105005\|V2-17 | 22 | BCRSignalingPathway |
| IGLV3-27 | 28791 | immunoglobulin lambda variable 3-27 | IGLV327\|V2-19 | 22 | BCRSignalingPathway |
| IGLV3-32 | 28787 | immunoglobulin lambda variable 3-32 (non-functional) | IGLV332\|V2-23P | 22 | BCRSignalingPathway |
| IGLV3-9 | 28804 | immunoglobulin lambda variable 3-9 (gene/pseudogene) | IGLV39\|V2-6 | 22 | BCRSignalingPathway |
| IGLV4-3 | 28786 | immunoglobulin lambda variable 4-3 | IGLV43\|V5-1 | 22 | BCRSignalingPathway |
| IGLV4-60 | 28785 | immunoglobulin lambda variable 4-60 | IGLV460\|V5-4 | 22 | BCRSignalingPathway |
| IGLV4-69 | 28784 | immunoglobulin lambda variable 4-69 | IGLV469\|V5-6 | 22 | BCRSignalingPathway |
| IGLV5-37 | 28783 | immunoglobulin lambda variable 5-37 | IGLV537\|V4-1 | 22 | BCRSignalingPathway |
| IGLV5-39 | 28782 | immunoglobulin lambda variable 5-39 | IGLV539 | 22 | BCRSignalingPathway |
| IGLV5-45 | 28781 | immunoglobulin lambda variable 5-45 | IGLV545\|V4-2 | 22 | BCRSignalingPathway |
| IGLV5-48 | 28780 | immunoglobulin lambda variable 5-48 (non-functional) | IGLV548\|V4-3 | 22 | BCRSignalingPathway |
| IGLV5-52 | 28779 | immunoglobulin lambda variable 5-52 | IGLV552\|V4-4 | 22 | BCRSignalingPathway |
| IGLV6-57 | 28778 | immunoglobulin lambda variable 6-57 | IGLV657\|MGC34845\|V1-22 | 22 | BCRSignalingPathway |
| IGLV7-43 | 28776 | immunoglobulin lambda variable 7-43 | IGLV743\|V3-2 | 22 | BCRSignalingPathway |
| IGLV7-46 | 28775 | immunoglobulin lambda variable 7-46 (gene/pseudogene) | IGLV746\|V3-3 | 22 | BCRSignalingPathway |
| IGLV8-61 | 28774 | immunoglobulin lambda variable 8-61 | IGLV861\|V3-4 | 22 | BCRSignalingPathway |
| IGLV9-49 | 28773 | immunoglobulin lambda variable 9-49 | IGLV949\|V5-2 | 22 | BCRSignalingPathway |
| C3 | 718 | complement component 3 | ARMD9\|ASP\|CPAMD1 | 19 | Chemokines |
| C5 | 727 | complement component 5 | CPAMD4\|FLJ17816\|FLJ17822\|MGC142298 | 9 | Chemokines |
| CAMP | 820 | cathelicidin antimicrobial peptide | CAP18\|CRAMP\|FALL-39\|FALL39\|HSD26\|LL37 | 3 | Chemokines |
| CCL1 | 6346 | chemokine (C-C motif) ligand 1 | I-309\|P500\|SCYA1\|SISe\|TCA3 | 17 | Chemokines |
| CCL11 | 6356 | chemokine (C-C motif) ligand 11 | MGC22554\|SCYA11 | 17 | Chemokines |
| CCL13 | 6357 | chemokine (C-C motif) ligand 13 | CKb10\|MCP-4\|MGC17134\|NCC-1\|NCC1\|SCYA13\|SCYL1 | 17 | Chemokines |
| CCL14 | 6358 | chemokine (C-C motif) ligand 14 | CC-1\|CC-3\|CKb1\|FLJ16015\|HCC-1\|HCC-3\|MCIF\|NCC-2\|NCC2\|SCYA14\|SCYL2\|SY14 | 17 | Chemokines |
| CCL14-CCL15 | 348249 | chemokine ligand 14, chemokine ligand 15 transcription unit | - | 17 | Chemokines |
| CCL15 | 6359 | chemokine (C-C motif) ligand 15 | HCC-2\|HMRP-2B\|LKN1\|Lkn-1\|MIP-1d\|MIP-5\|NCC-3\|NCC3\|SCYA15\|SCYL3\|SY15 | 17 | Chemokines |
| CCL16 | 6360 | chemokine (C-C motif) ligand 16 | CKb12\|HCC-4\|ILINCK\|LCC-1\|LEC\|LMC\|MGC117051\|Mtn-1\|NCC-4\|NCC4\|SCYA16\|SCYL4 | 17 | Chemokines |
| CCL17 | 6361 | chemokine (C-C motif) ligand 17 | A-152E5.3\|ABCD-2\|MGC138271\|MGC138273\|SCYA17\|TARC | 16 | Chemokines |
| CCL18 | 6362 | chemokine (C-C motif) ligand 18 (pulmonary and activation-regulated) | AMAC-1\|AMAC1\|CKb7\|DC-CK1\|DCCK1\|MIP-4\|PARC\|SCYA18 | 17 | Chemokines |
| CCL19 | 6363 | chemokine (C-C motif) ligand 19 | CKb11\|ELC\|MGC34433\|MIP-3b\|MIP3B\|SCYA19 | 9 | Chemokines |
| CCL2 | 6347 | chemokine (C-C motif) ligand 2 | GDCF-2\|HC11\|HSMCR30\|MCAF\|MCP-1\|MCP1\|MGC9434\|SCYA2\|SMC-CF | 17 | Chemokines |
| CCL20 | 6364 | chemokine (C-C motif) ligand 20 | CKb4\|LARC\|MIP-3a\|MIP3A\|SCYA20\|ST38 | 2 | Chemokines |
| CCL21 | 6366 | chemokine (C-C motif) ligand 21 | 6Ckine\|CKb9\|ECL\|MGC34555\|SCYA21\|SLC\|TCA4 | 9 | Chemokines |
| CCL22 | 6367 | chemokine (C-C motif) ligand 22 | A-152E5.1\|ABCD-1\|DC/B-CK\|MDC\|MGC34554\|SCYA22\|STCP-1 | 16 | Chemokines |
| CCL23 | 6368 | chemokine (C-C motif) ligand 23 | CK-BETA-8\|CKb8\|Ckb-8\|Ckb-8-1\|MIP-3\|MIP3\|MPIF-1\|SCYA23 | 17 | Chemokines |
| CCL24 | 6369 | chemokine (C-C motif) ligand 24 | Ckb-6\|MPIF-2\|MPIF2\|SCYA24 | 7 | Chemokines |
| CCL25 | 6370 | chemokine (C-C motif) ligand 25 | Ckb15\|MGC150327\|SCYA25\|TECK | 19 | Chemokines |
| CCL26 | 10344 | chemokine (C-C motif) ligand 26 | IMAC\|MGC126714\|MIP-4a\|MIP-4alpha\|SCYA26\|TSC-1 | 7 | Chemokines |
| CCL27 | 10850 | chemokine (C-C motif) ligand 27 | ALP\|CTACK\|CTAK\|ESKINE\|ILC\|PESKY\|SCYA27 | 9 | Chemokines |
| CCL28 | 56477 | chemokine (C-C motif) ligand 28 | CCK1\|MEC\|MGC71902\|SCYA28 | 5 | Chemokines |
| CCL3 | 6348 | chemokine (C-C motif) ligand 3 | G0S19-1\|LD78ALPHA\|MIP-1-alpha\|MIP1A\|SCYA3 | 17 | Chemokines |
| CCL3L1 | 6349 | chemokine (C-C motif) ligand 3-like 1 | 464.2\|D17S1718\|G0S19-2\|LD78\|LD78BETA\|MGC104178\|MGC12815\|MGC182017\|MIP1AP\|SCYA3L\|SCYA3L1 | 17 | Chemokines |
| CCL3L2 | 390788 | chemokine (C-C motif) ligand 3-like 2 | G0S19-3\|LD78gamma\|SCYA3L2 | 17 | Chemokines |
| CCL3L3 | 414062 | chemokine (C-C motif) ligand 3-like 3 | 464.2\|D17S1718\|LD78\|LD78BETA\|MGC12815\|SCYA3L\|SCYA3L1 | 17 | Chemokines |
| CCL4 | 6351 | chemokine (C-C motif) ligand 4 | ACT2\|AT744.1\|G-26\|LAG1\|MGC104418\|MGC126025\|MGC126026\|MIP-1-beta\|MIP1B\|MIP1B1\|SCYA2\|SCYA4 | 17 | Chemokines |
| CCL4L1 | 9560 | chemokine (C-C motif) ligand 4-like 1 | AT744.2\|CCL4L\|LAG-1\|LAG1\|SCYA4L | 17 | Chemokines |
| CCL4L2 | 388372 | chemokine (C-C motif) ligand 4-like 2 | AT744.2\|CCL4L\|SCYA4L | 17 | Chemokines |
| CCL5 | 6352 | chemokine (C-C motif) ligand 5 | D17S136E\|MGC17164\|RANTES\|SCYA5\|SISd\|TCP228 | 17 | Chemokines |
| CCL7 | 6354 | chemokine (C-C motif) ligand 7 | FIC\|MARC\|MCP-3\|MCP3\|MGC138463\|MGC138465\|NC28\|SCYA6\|SCYA7 | 17 | Chemokines |
| CCL8 | 6355 | chemokine (C-C motif) ligand 8 | HC14\|MCP-2\|MCP2\|SCYA10\|SCYA8 | 17 | Chemokines |
| CKLF | 51192 | chemokine-like factor | C32\|CKLF1\|CKLF2\|CKLF3\|CKLF4\|HSPC224\|UCK-1 | 16 | Chemokines |
| CMA1 | 1215 | chymase 1, mast cell | CYH\|MCT1\|MGC119890\|MGC119891\|chymase | 14 | Chemokines |
| CTSG | 1511 | cathepsin G | CG\|MGC23078 | 14 | Chemokines |
| CX3CL1 | 6376 | chemokine (C-X3-C motif) ligand 1 | ABCD-3\|C3Xkine\|CXC3\|CXC3C\|NTN\|NTT\|SCYD1\|fractalkine\|neurotactin | 16 | Chemokines |
| CXCL1 | 2919 | chemokine (C-X-C motif) ligand 1 (melanoma growth stimulating activity, alpha) | FSP\|GRO1\|GROa\|MGSA\|MGSA-a\|NAP-3\|SCYB1 | 4 | Chemokines |
| CXCL10 | 3627 | chemokine (C-X-C motif) ligand 10 | C7\|IFI10\|INP10\|IP-10\|SCYB10\|crg-2\|gIP-10\|mob-1 | 4 | Chemokines |
| CXCL11 | 6373 | chemokine (C-X-C motif) ligand 11 | H174\|I-TAC\|IP-9\|IP9\|MGC102770\|SCYB11\|SCYB9B\|b-R1 | 4 | Chemokines |
| CXCL12 | 6387 | chemokine (C-X-C motif) ligand 12 (stromal cell-derived factor 1) | PBSF\|SCYB12\|SDF-1a\|SDF-1b\|SDF1\|SDF1A\|SDF1B\|TLSF-a\|TLSF-b\|TPAR1 | 10 | Chemokines |
| CXCL13 | 10563 | chemokine (C-X-C motif) ligand 13 | ANGIE\|ANGIE2\|BCA-1\|BCA1\|BLC\|BLR1L\|SCYB13 | 4 | Chemokines |
| CXCL14 | 9547 | chemokine (C-X-C motif) ligand 14 | BMAC\|BRAK\|KS1\|Kec\|MGC10687\|MIP-2g\|NJAC\|SCYB14\|bolekine | 5 | Chemokines |
| CXCL16 | 58191 | chemokine (C-X-C motif) ligand 16 | CXCLG16\|SR-PSOX\|SRPSOX | 17 | Chemokines |
| CXCL17 | 284340 | chemokine (C-X-C motif) ligand 17 | DMC\|Dcip1\|MGC138300\|UNQ473\|VCC-1\|VCC1 | 19 | Chemokines |
| CXCL2 | 2920 | chemokine (C-X-C motif) ligand 2 | CINC-2a\|GRO2\|GROb\|MGSA-b\|MIP-2a\|MIP2\|MIP2A\|SCYB2 | 4 | Chemokines |
| CXCL3 | 2921 | chemokine (C-X-C motif) ligand 3 | CINC-2b\|GRO3\|GROg\|MIP-2b\|MIP2B\|SCYB3 | 4 | Chemokines |
| CXCL5 | 6374 | chemokine (C-X-C motif) ligand 5 | ENA-78\|SCYB5 | 4 | Chemokines |
| CXCL6 | 6372 | chemokine (C-X-C motif) ligand 6 (granulocyte chemotactic protein 2) | CKA-3\|GCP-2\|GCP2\|SCYB6 | 4 | Chemokines |
| CXCL9 | 4283 | chemokine (C-X-C motif) ligand 9 | CMK\|Humig\|MIG\|SCYB9\|crg-10 | 4 | Chemokines |
| CYR61 | 3491 | cysteine-rich, angiogenic inducer, 61 | CCN1\|GIG1\|IGFBP10 | 1 | Chemokines |
| DEFA1 | 1667 | defensin, alpha 1 | DEF1\|DEFA2\|HNP-1\|HP-1\|MGC138393\|MRS | 8 | Chemokines |
| DEFA3 | 1668 | defensin, alpha 3, neutrophil-specific | DEF3\|HNP-3\|HNP3\|HP-3 | 8 | Chemokines |
| DEFA5 | 1670 | defensin, alpha 5, Paneth cell-specific | DEF5\|HD-5\|MGC129728 | 8 | Chemokines |
| DEFB1 | 1672 | defensin, beta 1 | BD1\|DEFB-1\|DEFB101\|HBD1\|MGC51822 | 8 | Chemokines |
| DEFB103A | 55894 | defensin, beta 103A | DEFB103\|DEFB3\|HBD-3\|HBD3\|HBP-3\|HBP3 | 8 | Chemokines |
| DEFB104A | 140596 | defensin, beta 104A | BD-4\|DEFB-4\|DEFB104\|DEFB4\|MGC118942\|MGC118944\|MGC118945\|hBD-4 | 8 | Chemokines |
| DEFB4 | 1673 | defensin, beta 4 | DEFB-2\|DEFB102\|DEFB2\|HBD-2\|SAP1 | 8 | Chemokines |
| EDN1 | 1906 | endothelin 1 | ET1\|HDLCQ7 | 6 | Chemokines |
| EDN2 | 1907 | endothelin 2 | ET2\|PPET2 | 1 | Chemokines |
| EDN3 | 1908 | endothelin 3 | ET3\|MGC15067\|MGC61498 | 20 | Chemokines |
| FGF10 | 2255 | fibroblast growth factor 10 | - | 5 | Chemokines |
| FGF2 | 2247 | fibroblast growth factor 2 (basic) | BFGF\|FGFB\|HBGF-2 | 4 | Chemokines |
| HTN3 | 3347 | histatin 3 | HIS2\|HTN2\|HTN5 | 4 | Chemokines |
| IL8 | 3576 | interleukin 8 | CXCL8\|GCP-1\|GCP1\|LECT\|LUCT\|LYNAP\|MDNCF\|MONAP\|NAF\|NAP-1\|NAP1 | 4 | Chemokines |
| LECT2 | 3950 | leukocyte cell-derived chemotaxin 2 | MGC126628\|chm-II\|chm2 | 5 | Chemokines |
| PF4 | 5196 | platelet factor 4 | CXCL4\|MGC138298\|SCYB4 | 4 | Chemokines |
| PF4V1 | 5197 | platelet factor 4 variant 1 | CXCL4L1\|CXCL4V1\|PF4-ALT\|PF4A\|SCYB4V1 | 4 | Chemokines |
| PLAU | 5328 | plasminogen activator, urokinase | ATF\|UPA\|URK\|u-PA | 10 | Chemokines |
| PPBP | 5473 | pro-platelet basic protein (chemokine (C-X-C motif) ligand 7) | B-TG1\|Beta-TG\|CTAP-III\|CTAP3\|CTAPIII\|CXCL7\|LA-PF4\|LDGF\|MDGF\|NAP-2\|PBP\|SCYB7\|TC1\|TC2\|TGB\|TGB1\|THBGB\|THBGB1 | 4 | Chemokines |
| PPBPL1 | 728045 | pro-platelet basic protein-like 1 | TGB2 | 4 | Chemokines |
| PROK2 | 60675 | prokineticin 2 | BV8\|KAL4\|MIT1\|PK2 | 3 | Chemokines |
| RNASE2 | 6036 | ribonuclease, RNase A family, 2 (liver, eosinophil-derived neurotoxin) | EDN\|RNS2 | 14 | Chemokines |
| SAA1 | 6288 | serum amyloid A1 | MGC111216\|PIG4\|SAA\|TP53I4 | 11 | Chemokines |
| SAA2 | 6289 | serum amyloid A2 | - | 11 | Chemokines |
| SBDS | 51119 | Shwachman-Bodian-Diamond syndrome | CGI-97\|FLJ10917\|SDS\|SWDS | 7 | Chemokines |
| SEMA3A | 10371 | sema domain, immunoglobulin domain (Ig), short basic domain, secreted, (semaphorin) 3A | Hsema-I\|Hsema-III\|MGC133243\|SEMA1\|SEMAD\|SEMAIII\|SEMAL\|SemD\|coll-1 | 7 | Chemokines |
| SEMA3B | 7869 | sema domain, immunoglobulin domain (Ig), short basic domain, secreted, (semaphorin) 3B | FLJ34863\|LUCA-1\|SEMA5\|SEMAA\|SemA\|semaV | 3 | Chemokines |
| SEMA3C | 10512 | sema domain, immunoglobulin domain (Ig), short basic domain, secreted, (semaphorin) 3C | SEMAE\|SemE | 7 | Chemokines |
| SEMA3D | 223117 | sema domain, immunoglobulin domain (Ig), short basic domain, secreted, (semaphorin) 3D | MGC39708\|Sema-Z2\|coll-2 | 7 | Chemokines |
| SEMA3E | 9723 | sema domain, immunoglobulin domain (Ig), short basic domain, secreted, (semaphorin) 3E | KIAA0331\|M-SEMAH\|M-SemaK\|SEMAH\|coll-5 | 7 | Chemokines |
| SEMA3F | 6405 | sema domain, immunoglobulin domain (Ig), short basic domain, secreted, (semaphorin) 3F | SEMA-IV\|SEMA4\|SEMAK | 3 | Chemokines |
| SEMA3G | 56920 | sema domain, immunoglobulin domain (Ig), short basic domain, secreted, (semaphorin) 3G | FLJ00014\|MGC119473\|sem2 | 3 | Chemokines |
| SEMA4A | 64218 | sema domain, immunoglobulin domain (Ig), transmembrane domain (TM) and short cytoplasmic domain, (semaphorin) 4A | CORD10\|FLJ12287\|RP35\|SEMAB\|SEMB | 1 | Chemokines |
| SEMA4B | 10509 | sema domain, immunoglobulin domain (Ig), transmembrane domain (TM) and short cytoplasmic domain, (semaphorin) 4B | KIAA1745\|MGC131831\|SEMAC\|SemC | 15 | Chemokines |
| SEMA4C | 54910 | sema domain, immunoglobulin domain (Ig), transmembrane domain (TM) and short cytoplasmic domain, (semaphorin) 4C | FLJ20369\|KIAA1739\|M-SEMA-F\|MGC126382\|MGC126383\|SEMACL1\|SEMAF\|SEMAI | 2 | Chemokines |
| SEMA4D | 10507 | sema domain, immunoglobulin domain (Ig), transmembrane domain (TM) and short cytoplasmic domain, (semaphorin) 4D | C9orf164\|CD100\|FLJ33485\|FLJ34282\|FLJ39737\|FLJ46484\|M-sema-G\|MGC169138\|MGC169141\|SEMAJ\|coll-4 | 9 | Chemokines |
| SEMA4F | 10505 | sema domain, immunoglobulin domain (Ig), transmembrane domain (TM) and short cytoplasmic domain, (semaphorin) 4F | M-SEMA\|PRO2353\|SEMAM\|SEMAW\|m-Sema-M | 2 | Chemokines |
| SEMA4G | 57715 | sema domain, immunoglobulin domain (Ig), transmembrane domain (TM) and short cytoplasmic domain, (semaphorin) 4G | FLJ20590\|KIAA1619\|MGC102867 | 10 | Chemokines |
| SEMA5A | 9037 | sema domain, seven thrombospondin repeats (type 1 and type 1-like), transmembrane domain (TM) and short cytoplasmic domain, (semaphorin) 5A | FLJ12815\|SEMAF\|semF | 5 | Chemokines |
| SEMA5B | 54437 | sema domain, seven thrombospondin repeats (type 1 and type 1-like), transmembrane domain (TM) and short cytoplasmic domain, (semaphorin) 5B | FLJ10372\|KIAA1445\|SEMAG\|SemG | 3 | Chemokines |
| SEMA6A | 57556 | sema domain, transmembrane domain (TM), and cytoplasmic domain, (semaphorin) 6A | HT018\|KIAA1368\|SEMA\|SEMA6A1\|SEMAQ\|VIA | 5 | Chemokines |
| SEMA6B | 10501 | sema domain, transmembrane domain (TM), and cytoplasmic domain, (semaphorin) 6B | SEM-SEMA-Y\|SEMA-VIB\|SEMAN\|semaZ | 19 | Chemokines |
| SEMA6C | 10500 | sema domain, transmembrane domain (TM), and cytoplasmic domain, (semaphorin) 6C | SEMAY\|m-SemaY\|m-SemaY2 | 1 | Chemokines |
| SEMA6D | 80031 | sema domain, transmembrane domain (TM), and cytoplasmic domain, (semaphorin) 6D | FLJ11598\|KIAA1479 | 15 | Chemokines |
| SEMA7A | 8482 | semaphorin 7A, GPI membrane anchor (John Milton Hagen blood group) | CD108\|CDw108\|H-SEMA-K1\|H-Sema-L\|JMH\|MGC126692\|MGC126696\|SEMAK1\|SEMAL | 15 | Chemokines |
| SLIT1 | 6585 | slit homolog 1 (Drosophila) | MEGF4\|MGC164811\|SLIL1\|SLIT3\|Slit-1 | 10 | Chemokines |
| SLIT2 | 9353 | slit homolog 2 (Drosophila) | FLJ14420\|SLIL3\|Slit-2 | 4 | Chemokines |
| TNC | 3371 | tenascin C | HXB\|MGC167029\|TN | 9 | Chemokines |
| TYMP | 1890 | thymidine phosphorylase | ECGF1\|MNGIE\|PDECGF\|TP\|hPD-ECGF | 22 | Chemokines |
| XCL1 | 6375 | chemokine (C motif) ligand 1 | ATAC\|LPTN\|LTN\|SCM-1\|SCM-1a\|SCM1\|SCYC1 | 1 | Chemokines |
| XCL2 | 6846 | chemokine (C motif) ligand 2 | SCM-1b\|SCM1B\|SCYC2 | 1 | Chemokines |
| C5AR1 | 728 | complement component 5a receptor 1 | C5A\|C5AR\|C5R1\|CD88 | 19 | Chemokine Receptors |
| CCBP2 | 1238 | chemokine binding protein 2 | CCR10\|CCR9\|CMKBR9\|D6\|MGC126678\|MGC138250\|hD6 | 3 | Chemokine Receptors |
| CCR1 | 1230 | chemokine (C-C motif) receptor 1 | CD191\|CKR-1\|CKR1\|CMKBR1\|HM145\|MIP1aR\|SCYAR1 | 3 | Chemokine Receptors |
| CCR10 | 2826 | chemokine (C-C motif) receptor 10 | GPR2 | 17 | Chemokine Receptors |
| CCR3 | 1232 | chemokine (C-C motif) receptor 3 | CC-CKR-3\|CD193\|CKR3\|CMKBR3\|MGC102841 | 3 | Chemokine Receptors |
| CCR4 | 1233 | chemokine (C-C motif) receptor 4 | CC-CKR-4\|CD194\|CKR4\|CMKBR4\|ChemR13\|HGCN:14099\|K5-5\|MGC88293 | 3 | Chemokine Receptors |
| CCR5 | 1234 | chemokine (C-C motif) receptor 5 | CC-CKR-5\|CCCKR5\|CD195\|CKR-5\|CKR5\|CMKBR5\|FLJ78003\|IDDM22 | 3 | Chemokine Receptors |
| CCR6 | 1235 | chemokine (C-C motif) receptor 6 | BN-1\|CD196\|CKR-L3\|CKR6\|CKRL3\|CMKBR6\|DCR2\|DRY-6\|GPR-CY4\|GPR29\|GPRCY4\|STRL22 | 6 | Chemokine Receptors |
| CCR7 | 1236 | chemokine (C-C motif) receptor 7 | BLR2\|CD197\|CDw197\|CMKBR7\|EBI1 | 17 | Chemokine Receptors |
| CCR8 | 1237 | chemokine (C-C motif) receptor 8 | CDw198\|CKR-L1\|CKRL1\|CMKBR8\|CMKBRL2\|CY6\|GPR-CY6\|MGC129966\|MGC129973\|TER1 | 3 | Chemokine Receptors |
| CCR9 | 10803 | chemokine (C-C motif) receptor 9 | CDw199\|GPR-9-6\|GPR28 | 3 | Chemokine Receptors |
| CCRL1 | 51554 | chemokine (C-C motif) receptor-like 1 | CC-CKR-11\|CCBP2\|CCR10\|CCR11\|CCX-CKR\|CKR-11\|PPR1\|VSHK1 | 3 | Chemokine Receptors |
| CCRL2 | 9034 | chemokine (C-C motif) receptor-like 2 | CKRX\|CRAM-A\|CRAM-B\|FLJ55815\|HCR\|MGC116710\|MGC34104 | 3 | Chemokine Receptors |
| CMKLR1 | 1240 | chemokine-like receptor 1 | CHEMERINR\|ChemR23\|DEZ\|MGC126105\|MGC126106 | 12 | Chemokine Receptors |
| CX3CR1 | 1524 | chemokine (C-X3-C motif) receptor 1 | CCRL1\|CMKBRL1\|CMKDR1\|GPR13\|GPRV28\|V28 | 3 | Chemokine Receptors |
| CXCR3 | 2833 | chemokine (C-X-C motif) receptor 3 | CD182\|CD183\|CKR-L2\|CMKAR3\|GPR9\|IP10-R\|Mig-R\|MigR | X | Chemokine Receptors |
| CXCR4 | 7852 | chemokine (C-X-C motif) receptor 4 | CD184\|D2S201E\|FB22\|HM89\|HSY3RR\|LAP3\|LCR1\|LESTR\|NPY3R\|NPYR\|NPYRL\|NPYY3R\|WHIM | 2 | Chemokine Receptors |
| CXCR5 | 643 | chemokine (C-X-C motif) receptor 5 | BLR1\|CD185\|MDR15\|MGC117347 | 11 | Chemokine Receptors |
| CXCR6 | 10663 | chemokine (C-X-C motif) receptor 6 | BONZO\|CD186\|STRL33\|TYMSTR | 3 | Chemokine Receptors |
| CXCR7 | 57007 | chemokine (C-X-C motif) receptor 7 | CMKOR1\|GPR159\|RDC1 | 2 | Chemokine Receptors |
| CYSLTR1 | 10800 | cysteinyl leukotriene receptor 1 | CYSLT1\|CYSLT1R\|CYSLTR\|HG55\|HMTMF81\|MGC46139 | X | Chemokine Receptors |
| CYSLTR2 | 57105 | cysteinyl leukotriene receptor 2 | CYSLT2\|CYSLT2R\|GPCR\|HG57\|HPN321\|KPG_011\|PSEC0146\|hGPCR21 | 13 | Chemokine Receptors |
| DARC | 2532 | Duffy blood group, chemokine receptor | CCBP1\|CD234\|Dfy\|FY\|GPD\|GpFy\|WBCQ1 | 1 | Chemokine Receptors |
| EDNRA | 1909 | endothelin receptor type A | ETA\|ETRA | 4 | Chemokine Receptors |
| EDNRB | 1910 | endothelin receptor type B | ABCDS\|ETB\|ETBR\|ETRB\|HSCR\|HSCR2 | 13 | Chemokine Receptors |
| FPR1 | 2357 | formyl peptide receptor 1 | FMLP\|FPR | 19 | Chemokine Receptors |
| FPR2 | 2358 | formyl peptide receptor 2 | ALXR\|FMLP-R-II\|FMLPX\|FPR2A\|FPRH1\|FPRH2\|FPRL1\|HM63\|LXA4R | 19 | Chemokine Receptors |
| FPR2 | 2358 | formyl peptide receptor 2 | ALXR\|FMLP-R-II\|FMLPX\|FPR2A\|FPRH1\|FPRH2\|FPRL1\|HM63\|LXA4R | 19 | Chemokine Receptors |
| GPR17 | 2840 | G protein-coupled receptor 17 | DKFZp686M18273 | 2 | Chemokine Receptors |
| GPR32 | 2854 | G protein-coupled receptor 32 | - | 19 | Chemokine Receptors |
| GPR33 | 2856 | G protein-coupled receptor 33 (gene/pseudogene) | - | 14 | Chemokine Receptors |
| GPR44 | 11251 | G protein-coupled receptor 44 | CD294\|CRTH2\|DP2 | 11 | Chemokine Receptors |
| GPR77 | 27202 | G protein-coupled receptor 77 | C5L2\|GPF77 | 19 | Chemokine Receptors |
| IL8RA | 3577 | interleukin 8 receptor, alpha | C-C\|C-C-CKR-1\|CD128\|CD181\|CDw128a\|CKR-1\|CMKAR1\|CXCR1\|IL8R1\|IL8RBA | 2 | Chemokine Receptors |
| IL8RB | 3579 | interleukin 8 receptor, beta | CD182\|CDw128b\|CMKAR2\|CXCR2\|IL8R2\|IL8RA | 2 | Chemokine Receptors |
| LTB4R | 1241 | leukotriene B4 receptor | BLT1\|BLTR\|CMKRL1\|GPR16\|LTB4R1\|LTBR1\|P2RY7\|P2Y7 | 14 | Chemokine Receptors |
| LTB4R2 | 56413 | leukotriene B4 receptor 2 | BLT2\|BLTR2\|JULF2\|KPG_004\|NOP9 | 14 | Chemokine Receptors |
| PLAUR | 5329 | plasminogen activator, urokinase receptor | CD87\|UPAR\|URKR | 19 | Chemokine Receptors |
| PLXNA1 | 5361 | plexin A1 | NOV\|NOVP\|PLEXIN-A1\|PLXN1 | 3 | Chemokine Receptors |
| PLXNA2 | 5362 | plexin A2 | FLJ11751\|FLJ30634\|KIAA0463\|OCT\|PLXN2 | 1 | Chemokine Receptors |
| PLXNA3 | 55558 | plexin A3 | 6.3\|HSSEXGENE\|PLEXIN-A3\|PLXN3\|PLXN4\|SEX\|XAP-6 | X | Chemokine Receptors |
| PLXNA4 | 91584 | plexin A4 | DKFZp434G0625\|DKFZp566O0546\|FAYV2820\|FLJ35026\|FLJ38287\|KIAA1550\|PLEXA4\|PLXNA4A\|PLXNA4B\|PRO34003 | 7 | Chemokine Receptors |
| PLXNB1 | 5364 | plexin B1 | KIAA0407\|MGC149167\|PLEXIN-B1\|PLXN5\|SEP | 3 | Chemokine Receptors |
| PLXNB2 | 23654 | plexin B2 | KIAA0315\|MM1\|Nbla00445\|PLEXB2\|dJ402G11.3 | 22 | Chemokine Receptors |
| PLXNB3 | 5365 | plexin B3 | FLJ76953\|PLEXB3\|PLEXR\|PLXN6 | X | Chemokine Receptors |
| PLXNC1 | 10154 | plexin C1 | CD232\|PLXN-C1\|VESPR | 12 | Chemokine Receptors |
| PLXND1 | 23129 | plexin D1 | KIAA0620\|MGC75353\|PLEXD1 | 3 | Chemokine Receptors |
| PTAFR | 5724 | platelet-activating factor receptor | PAFR | 1 | Chemokine Receptors |
| ROBO1 | 6091 | roundabout, axon guidance receptor, homolog 1 (Drosophila) | DUTT1\|FLJ21882\|MGC131599\|MGC133277\|SAX3 | 3 | Chemokine Receptors |
| ROBO2 | 6092 | roundabout, axon guidance receptor, homolog 2 (Drosophila) | KIAA1568\|SAX3 | 3 | Chemokine Receptors |
| ROBO3 | 64221 | roundabout, axon guidance receptor, homolog 3 (Drosophila) | FLJ21044\|HGPPS\|HGPS\|RBIG1\|RIG1 | 11 | Chemokine Receptors |
| RXFP3 | 51289 | relaxin/insulin-like family peptide receptor 3 | GPCR135\|MGC141998\|MGC142000\|RLN3R1\|RXFPR3\|SALPR | 5 | Chemokine Receptors |
| XCR1 | 2829 | chemokine (C motif) receptor 1 | CCXCR1\|GPR5 | 3 | Chemokine Receptors |
| ADIPOQ | 9370 | adiponectin, C1Q and collagen domain containing | ACDC\|ACRP30\|ADIPQTL1\|ADPN\|APM-1\|APM1\|GBP28\|adiponectin | 3 | Cytokines |
| ADM | 133 | adrenomedullin | AM | 11 | Cytokines |
| ADM2 | 79924 | adrenomedullin 2 | AM2\|FLJ21135\|dJ579N16.4 | 22 | Cytokines |
| AGRP | 181 | agouti related protein homolog (mouse) | AGRT\|ART\|ASIP2\|MGC118963 | 16 | Cytokines |
| AGT | 183 | angiotensinogen (serpin peptidase inhibitor, clade A, member 8) | ANHU\|FLJ92595\|FLJ97926\|SERPINA8 | 1 | Cytokines |
| AMBN | 258 | ameloblastin (enamel matrix protein) | - | 4 | Cytokines |
| AMELX | 265 | amelogenin (amelogenesis imperfecta 1, X-linked) | AIH1\|ALGN\|AMG\|AMGL\|AMGX | X | Cytokines |
| AMH | 268 | anti-Mullerian hormone | MIF\|MIS | 19 | Cytokines |
| ANGPTL5 | 253935 | angiopoietin-like 5 | - | 11 | Cytokines |
| ANGPTL7 | 10218 | angiopoietin-like 7 | AngX\|CDT6\|RP4-647M16.2\|dJ647M16.1 | 1 | Cytokines |
| APLN | 8862 | apelin | XNPEP2 | X | Cytokines |
| AREG | 374 | amphiregulin | AR\|CRDGF\|MGC13647\|SDGF | 4 | Cytokines |
| ARMET | 7873 | arginine-rich, mutated in early stage tumors | ARP\|MANF\|MGC142148\|MGC142150 | 3 | Cytokines |
| ARMETL1 | 441549 | arginine-rich, mutated in early stage tumors-like 1 | cdnf | 10 | Cytokines |
| ARTN | 9048 | artemin | ENOVIN\|EVN\|NBN | 1 | Cytokines |
| AVP | 551 | arginine vasopressin | ADH\|ARVP\|AVP-NPII\|AVRP\|VP | 20 | Cytokines |
| AZU1 | 566 | azurocidin 1 | AZAMP\|AZU\|CAP37\|HBP\|HUMAZUR\|NAZC | 19 | Cytokines |
| BDNF | 627 | brain-derived neurotrophic factor | MGC34632 | 11 | Cytokines |
| BMP1 | 649 | bone morphogenetic protein 1 | FLJ44432\|PCOLC\|PCP\|TLD\|pCP-2 | 8 | Cytokines |
| BMP10 | 27302 | bone morphogenetic protein 10 | MGC126783 | 2 | Cytokines |
| BMP15 | 9210 | bone morphogenetic protein 15 | GDF9B\|ODG2\|POF4 | X | Cytokines |
| BMP2 | 650 | bone morphogenetic protein 2 | BMP2A | 20 | Cytokines |
| BMP3 | 651 | bone morphogenetic protein 3 | BMP-3A | 4 | Cytokines |
| BMP4 | 652 | bone morphogenetic protein 4 | BMP2B\|BMP2B1\|MCOPS6\|OFC11\|ZYME | 14 | Cytokines |
| BMP5 | 653 | bone morphogenetic protein 5 | MGC34244 | 6 | Cytokines |
| BMP6 | 654 | bone morphogenetic protein 6 | VGR\|VGR1 | 6 | Cytokines |
| BMP7 | 655 | bone morphogenetic protein 7 | OP-1 | 20 | Cytokines |
| BMP8A | 353500 | bone morphogenetic protein 8a | FLJ14351\|FLJ45264 | 1 | Cytokines |
| BMP8B | 656 | bone morphogenetic protein 8b | BMP8\|MGC131757\|OP2 | 1 | Cytokines |
| BTC | 685 | betacellulin | - | 4 | Cytokines |
| C19orf10 | 56005 | chromosome 19 open reading frame 10 | EUROIMAGE1875335\|IL25\|IL27\|IL27w\|R33729_1\|SF20 | 19 | Cytokines |
| C3 | 718 | complement component 3 | ARMD9\|ASP\|CPAMD1 | 19 | Cytokines |
| C5 | 727 | complement component 5 | CPAMD4\|FLJ17816\|FLJ17822\|MGC142298 | 9 | Cytokines |
| CALCA | 796 | calcitonin-related polypeptide alpha | CALC1\|CGRP\|CGRP-I\|CGRP1\|CT\|KC\|MGC126648 | 11 | Cytokines |
| CALCB | 797 | calcitonin-related polypeptide beta | CALC2\|CGRP-II\|CGRP2\|FLJ30166 | 11 | Cytokines |
| CAMP | 820 | cathelicidin antimicrobial peptide | CAP18\|CRAMP\|FALL-39\|FALL39\|HSD26\|LL37 | 3 | Cytokines |
| CAT | 847 | catalase | MGC138422\|MGC138424 | 11 | Cytokines |
| CCK | 885 | cholecystokinin | MGC117187 | 3 | Cytokines |
| CCL1 | 6346 | chemokine (C-C motif) ligand 1 | I-309\|P500\|SCYA1\|SISe\|TCA3 | 17 | Cytokines |
| CCL11 | 6356 | chemokine (C-C motif) ligand 11 | MGC22554\|SCYA11 | 17 | Cytokines |
| CCL13 | 6357 | chemokine (C-C motif) ligand 13 | CKb10\|MCP-4\|MGC17134\|NCC-1\|NCC1\|SCYA13\|SCYL1 | 17 | Cytokines |
| CCL14 | 6358 | chemokine (C-C motif) ligand 14 | CC-1\|CC-3\|CKb1\|FLJ16015\|HCC-1\|HCC-3\|MCIF\|NCC-2\|NCC2\|SCYA14\|SCYL2\|SY14 | 17 | Cytokines |
| CCL14-CCL15 | 348249 | chemokine ligand 14, chemokine ligand 15 transcription unit | - | 17 | Cytokines |
| CCL15 | 6359 | chemokine (C-C motif) ligand 15 | HCC-2\|HMRP-2B\|LKN1\|Lkn-1\|MIP-1d\|MIP-5\|NCC-3\|NCC3\|SCYA15\|SCYL3\|SY15 | 17 | Cytokines |
| CCL16 | 6360 | chemokine (C-C motif) ligand 16 | CKb12\|HCC-4\|ILINCK\|LCC-1\|LEC\|LMC\|MGC117051\|Mtn-1\|NCC-4\|NCC4\|SCYA16\|SCYL4 | 17 | Cytokines |
| CCL17 | 6361 | chemokine (C-C motif) ligand 17 | A-152E5.3\|ABCD-2\|MGC138271\|MGC138273\|SCYA17\|TARC | 16 | Cytokines |
| CCL18 | 6362 | chemokine (C-C motif) ligand 18 (pulmonary and activation-regulated) | AMAC-1\|AMAC1\|CKb7\|DC-CK1\|DCCK1\|MIP-4\|PARC\|SCYA18 | 17 | Cytokines |
| CCL19 | 6363 | chemokine (C-C motif) ligand 19 | CKb11\|ELC\|MGC34433\|MIP-3b\|MIP3B\|SCYA19 | 9 | Cytokines |
| CCL2 | 6347 | chemokine (C-C motif) ligand 2 | GDCF-2\|HC11\|HSMCR30\|MCAF\|MCP-1\|MCP1\|MGC9434\|SCYA2\|SMC-CF | 17 | Cytokines |
| CCL20 | 6364 | chemokine (C-C motif) ligand 20 | CKb4\|LARC\|MIP-3a\|MIP3A\|SCYA20\|ST38 | 2 | Cytokines |
| CCL21 | 6366 | chemokine (C-C motif) ligand 21 | 6Ckine\|CKb9\|ECL\|MGC34555\|SCYA21\|SLC\|TCA4 | 9 | Cytokines |
| CCL22 | 6367 | chemokine (C-C motif) ligand 22 | A-152E5.1\|ABCD-1\|DC/B-CK\|MDC\|MGC34554\|SCYA22\|STCP-1 | 16 | Cytokines |
| CCL23 | 6368 | chemokine (C-C motif) ligand 23 | CK-BETA-8\|CKb8\|Ckb-8\|Ckb-8-1\|MIP-3\|MIP3\|MPIF-1\|SCYA23 | 17 | Cytokines |
| CCL24 | 6369 | chemokine (C-C motif) ligand 24 | Ckb-6\|MPIF-2\|MPIF2\|SCYA24 | 7 | Cytokines |
| CCL25 | 6370 | chemokine (C-C motif) ligand 25 | Ckb15\|MGC150327\|SCYA25\|TECK | 19 | Cytokines |
| CCL26 | 10344 | chemokine (C-C motif) ligand 26 | IMAC\|MGC126714\|MIP-4a\|MIP-4alpha\|SCYA26\|TSC-1 | 7 | Cytokines |
| CCL27 | 10850 | chemokine (C-C motif) ligand 27 | ALP\|CTACK\|CTAK\|ESKINE\|ILC\|PESKY\|SCYA27 | 9 | Cytokines |
| CCL28 | 56477 | chemokine (C-C motif) ligand 28 | CCK1\|MEC\|MGC71902\|SCYA28 | 5 | Cytokines |
| CCL3 | 6348 | chemokine (C-C motif) ligand 3 | G0S19-1\|LD78ALPHA\|MIP-1-alpha\|MIP1A\|SCYA3 | 17 | Cytokines |
| CCL3L1 | 6349 | chemokine (C-C motif) ligand 3-like 1 | 464.2\|D17S1718\|G0S19-2\|LD78\|LD78BETA\|MGC104178\|MGC12815\|MGC182017\|MIP1AP\|SCYA3L\|SCYA3L1 | 17 | Cytokines |
| CCL3L2 | 390788 | chemokine (C-C motif) ligand 3-like 2 | G0S19-3\|LD78gamma\|SCYA3L2 | 17 | Cytokines |
| CCL3L3 | 414062 | chemokine (C-C motif) ligand 3-like 3 | 464.2\|D17S1718\|LD78\|LD78BETA\|MGC12815\|SCYA3L\|SCYA3L1 | 17 | Cytokines |
| CCL4 | 6351 | chemokine (C-C motif) ligand 4 | ACT2\|AT744.1\|G-26\|LAG1\|MGC104418\|MGC126025\|MGC126026\|MIP-1-beta\|MIP1B\|MIP1B1\|SCYA2\|SCYA4 | 17 | Cytokines |
| CCL4L1 | 9560 | chemokine (C-C motif) ligand 4-like 1 | AT744.2\|CCL4L\|LAG-1\|LAG1\|SCYA4L | 17 | Cytokines |
| CCL4L2 | 388372 | chemokine (C-C motif) ligand 4-like 2 | AT744.2\|CCL4L\|SCYA4L | 17 | Cytokines |
| CCL5 | 6352 | chemokine (C-C motif) ligand 5 | D17S136E\|MGC17164\|RANTES\|SCYA5\|SISd\|TCP228 | 17 | Cytokines |
| CCL7 | 6354 | chemokine (C-C motif) ligand 7 | FIC\|MARC\|MCP-3\|MCP3\|MGC138463\|MGC138465\|NC28\|SCYA6\|SCYA7 | 17 | Cytokines |
| CCL8 | 6355 | chemokine (C-C motif) ligand 8 | HC14\|MCP-2\|MCP2\|SCYA10\|SCYA8 | 17 | Cytokines |
| CD320 | 51293 | CD320 molecule | 8D6\|8D6A | 19 | Cytokines |
| CD40LG | 959 | CD40 ligand | CD154\|CD40L\|HIGM1\|IGM\|IMD3\|T-BAM\|TNFSF5\|TRAP\|gp39\|hCD40L | X | Cytokines |
| CD70 | 970 | CD70 molecule | CD27L\|CD27LG\|TNFSF7 | 19 | Cytokines |
| CECR1 | 51816 | cat eye syndrome chromosome region, candidate 1 | ADGF\|IDGFL | 22 | Cytokines |
| CER1 | 9350 | cerberus 1, cysteine knot superfamily, homolog (Xenopus laevis) | DAND4\|MGC119894\|MGC119895\|MGC96951 | 9 | Cytokines |
| CGA | 1081 | glycoprotein hormones, alpha polypeptide | CG-ALPHA\|FSHA\|GPHA1\|GPHa\|HCG\|LHA\|TSHA | 6 | Cytokines |
| CGB | 1082 | chorionic gonadotropin, beta polypeptide | CGB3\|hCGB | 19 | Cytokines |
| CGB1 | 114335 | chorionic gonadotropin, beta polypeptide 1 | - | 19 | Cytokines |
| CGB2 | 114336 | chorionic gonadotropin, beta polypeptide 2 | - | 19 | Cytokines |
| CGB5 | 93659 | chorionic gonadotropin, beta polypeptide 5 | HCG\|MGC119822 | 19 | Cytokines |
| CGB7 | 94027 | chorionic gonadotropin, beta polypeptide 7 | CG-beta-a\|FLJ35403\|FLJ43118 | 19 | Cytokines |
| CGB8 | 94115 | chorionic gonadotropin, beta polypeptide 8 | - | 19 | Cytokines |
| CHGA | 1113 | chromogranin A (parathyroid secretory protein 1) | CGA | 14 | Cytokines |
| CHGB | 1114 | chromogranin B (secretogranin 1) | SCG1 | 20 | Cytokines |
| CKLF | 51192 | chemokine-like factor | C32\|CKLF1\|CKLF2\|CKLF3\|CKLF4\|HSPC224\|UCK-1 | 16 | Cytokines |
| CLCF1 | 23529 | cardiotrophin-like cytokine factor 1 | BSF3\|CISS2\|CLC\|NNT1\|NR6 | 11 | Cytokines |
| CLEC11A | 6320 | C-type lectin domain family 11, member A | CLECSF3\|LSLCL\|P47\|SCGF | 19 | Cytokines |
| CMA1 | 1215 | chymase 1, mast cell | CYH\|MCT1\|MGC119890\|MGC119891\|chymase | 14 | Cytokines |
| CMTM1 | 113540 | CKLF-like MARVEL transmembrane domain containing 1 | CKLFH\|CKLFH1\|CKLFSF1\|MGC71870 | 16 | Cytokines |
| CMTM2 | 146225 | CKLF-like MARVEL transmembrane domain containing 2 | CKLFSF2\|MGC39436 | 16 | Cytokines |
| CMTM3 | 123920 | CKLF-like MARVEL transmembrane domain containing 3 | BNAS2\|CKLFSF3\|FLJ31762\|MGC51956 | 16 | Cytokines |
| CMTM4 | 146223 | CKLF-like MARVEL transmembrane domain containing 4 | CKLFSF4 | 16 | Cytokines |
| CMTM5 | 116173 | CKLF-like MARVEL transmembrane domain containing 5 | CKLFSF5\|FLJ37521 | 14 | Cytokines |
| CMTM6 | 54918 | CKLF-like MARVEL transmembrane domain containing 6 | CKLFSF6\|FLJ20396\|PRO2219 | 3 | Cytokines |
| CMTM7 | 112616 | CKLF-like MARVEL transmembrane domain containing 7 | CKLFSF7\|FLJ30992 | 3 | Cytokines |
| CMTM8 | 152189 | CKLF-like MARVEL transmembrane domain containing 8 | CKLFSF8\|CKLFSF8-V2 | 3 | Cytokines |
| CNTF | 1270 | ciliary neurotrophic factor | HCNTF | 11 | Cytokines |
| CORT | 1325 | cortistatin | CST-14\|CST-17\|CST-29 | 1 | Cytokines |
| CRH | 1392 | corticotropin releasing hormone | CRF | 8 | Cytokines |
| CSF1 | 1435 | colony stimulating factor 1 (macrophage) | MCSF\|MGC31930 | 1 | Cytokines |
| CSF2 | 1437 | colony stimulating factor 2 (granulocyte-macrophage) | GMCSF\|MGC131935\|MGC138897 | 5 | Cytokines |
| CSF3 | 1440 | colony stimulating factor 3 (granulocyte) | G-CSF\|GCSF\|MGC45931 | 17 | Cytokines |
| CSH1 | 1442 | chorionic somatomammotropin hormone 1 (placental lactogen) | CSA\|CSMT\|FLJ75407\|PL | 17 | Cytokines |
| CSH2 | 1443 | chorionic somatomammotropin hormone 2 | CS-2\|CSB\|hCS-B | 17 | Cytokines |
| CSHL1 | 1444 | chorionic somatomammotropin hormone-like 1 | CS-5\|CSHP1\|CSL\|MGC149868\|hCS-L | 17 | Cytokines |
| CSPG5 | 10675 | chondroitin sulfate proteoglycan 5 (neuroglycan C) | MGC44034\|NGC | 3 | Cytokines |
| CTF1 | 1489 | cardiotrophin 1 | CT-1\|CT1 | 16 | Cytokines |
| CTGF | 1490 | connective tissue growth factor | CCN2\|HCS24\|IGFBP8\|MGC102839\|NOV2 | 6 | Cytokines |
| CTSG | 1511 | cathepsin G | CG\|MGC23078 | 14 | Cytokines |
| CX3CL1 | 6376 | chemokine (C-X3-C motif) ligand 1 | ABCD-3\|C3Xkine\|CXC3\|CXC3C\|NTN\|NTT\|SCYD1\|fractalkine\|neurotactin | 16 | Cytokines |
| CXCL1 | 2919 | chemokine (C-X-C motif) ligand 1 (melanoma growth stimulating activity, alpha) | FSP\|GRO1\|GROa\|MGSA\|MGSA-a\|NAP-3\|SCYB1 | 4 | Cytokines |
| CXCL10 | 3627 | chemokine (C-X-C motif) ligand 10 | C7\|IFI10\|INP10\|IP-10\|SCYB10\|crg-2\|gIP-10\|mob-1 | 4 | Cytokines |
| CXCL11 | 6373 | chemokine (C-X-C motif) ligand 11 | H174\|I-TAC\|IP-9\|IP9\|MGC102770\|SCYB11\|SCYB9B\|b-R1 | 4 | Cytokines |
| CXCL12 | 6387 | chemokine (C-X-C motif) ligand 12 (stromal cell-derived factor 1) | PBSF\|SCYB12\|SDF-1a\|SDF-1b\|SDF1\|SDF1A\|SDF1B\|TLSF-a\|TLSF-b\|TPAR1 | 10 | Cytokines |
| CXCL13 | 10563 | chemokine (C-X-C motif) ligand 13 | ANGIE\|ANGIE2\|BCA-1\|BCA1\|BLC\|BLR1L\|SCYB13 | 4 | Cytokines |
| CXCL14 | 9547 | chemokine (C-X-C motif) ligand 14 | BMAC\|BRAK\|KS1\|Kec\|MGC10687\|MIP-2g\|NJAC\|SCYB14\|bolekine | 5 | Cytokines |
| CXCL16 | 58191 | chemokine (C-X-C motif) ligand 16 | CXCLG16\|SR-PSOX\|SRPSOX | 17 | Cytokines |
| CXCL17 | 284340 | chemokine (C-X-C motif) ligand 17 | DMC\|Dcip1\|MGC138300\|UNQ473\|VCC-1\|VCC1 | 19 | Cytokines |
| CXCL2 | 2920 | chemokine (C-X-C motif) ligand 2 | CINC-2a\|GRO2\|GROb\|MGSA-b\|MIP-2a\|MIP2\|MIP2A\|SCYB2 | 4 | Cytokines |
| CXCL3 | 2921 | chemokine (C-X-C motif) ligand 3 | CINC-2b\|GRO3\|GROg\|MIP-2b\|MIP2B\|SCYB3 | 4 | Cytokines |
| CXCL5 | 6374 | chemokine (C-X-C motif) ligand 5 | ENA-78\|SCYB5 | 4 | Cytokines |
| CXCL6 | 6372 | chemokine (C-X-C motif) ligand 6 (granulocyte chemotactic protein 2) | CKA-3\|GCP-2\|GCP2\|SCYB6 | 4 | Cytokines |
| CXCL9 | 4283 | chemokine (C-X-C motif) ligand 9 | CMK\|Humig\|MIG\|SCYB9\|crg-10 | 4 | Cytokines |
| CYR61 | 3491 | cysteine-rich, angiogenic inducer, 61 | CCN1\|GIG1\|IGFBP10 | 1 | Cytokines |
| DEFA1 | 1667 | defensin, alpha 1 | DEF1\|DEFA2\|HNP-1\|HP-1\|MGC138393\|MRS | 8 | Cytokines |
| DEFA3 | 1668 | defensin, alpha 3, neutrophil-specific | DEF3\|HNP-3\|HNP3\|HP-3 | 8 | Cytokines |
| DEFA5 | 1670 | defensin, alpha 5, Paneth cell-specific | DEF5\|HD-5\|MGC129728 | 8 | Cytokines |
| DEFB1 | 1672 | defensin, beta 1 | BD1\|DEFB-1\|DEFB101\|HBD1\|MGC51822 | 8 | Cytokines |
| DEFB103A | 55894 | defensin, beta 103A | DEFB103\|DEFB3\|HBD-3\|HBD3\|HBP-3\|HBP3 | 8 | Cytokines |
| DEFB104A | 140596 | defensin, beta 104A | BD-4\|DEFB-4\|DEFB104\|DEFB4\|MGC118942\|MGC118944\|MGC118945\|hBD-4 | 8 | Cytokines |
| DEFB4 | 1673 | defensin, beta 4 | DEFB-2\|DEFB102\|DEFB2\|HBD-2\|SAP1 | 8 | Cytokines |
| DKK1 | 22943 | dickkopf homolog 1 (Xenopus laevis) | DKK-1\|SK | 10 | Cytokines |
| EBI3 | 10148 | Epstein-Barr virus induced 3 | IL27B | 19 | Cytokines |
| EDN1 | 1906 | endothelin 1 | ET1\|HDLCQ7 | 6 | Cytokines |
| EDN2 | 1907 | endothelin 2 | ET2\|PPET2 | 1 | Cytokines |
| EDN3 | 1908 | endothelin 3 | ET3\|MGC15067\|MGC61498 | 20 | Cytokines |
| EGF | 1950 | epidermal growth factor (beta-urogastrone) | HOMG4\|URG | 4 | Cytokines |
| EPGN | 255324 | epithelial mitogen homolog (mouse) | ALGV3072\|EPG\|FLJ75542\|PRO9904\|epigen | 4 | Cytokines |
| EPO | 2056 | erythropoietin | EP\|MGC138142\|MVCD2 | 7 | Cytokines |
| EREG | 2069 | epiregulin | ER | 4 | Cytokines |
| ESM1 | 11082 | endothelial cell-specific molecule 1 | endocan | 5 | Cytokines |
| FAM3B | 54097 | family with sequence similarity 3, member B | 2-21\|C21orf11\|C21orf76\|ORF9\|PANDER\|PRED44 | 21 | Cytokines |
| FAM3C | 10447 | family with sequence similarity 3, member C | GS3786\|ILEI | 7 | Cytokines |
| FAM3D | 131177 | family with sequence similarity 3, member D | EF7\|OIT1 | 3 | Cytokines |
| FASLG | 356 | Fas ligand (TNF superfamily, member 6) | APT1LG1\|CD178\|CD95L\|FASL\|TNFSF6 | 1 | Cytokines |
| FGF1 | 2246 | fibroblast growth factor 1 (acidic) | AFGF\|ECGF\|ECGF-beta\|ECGFA\|ECGFB\|FGF-alpha\|FGFA\|GLIO703\|HBGF1 | 5 | Cytokines |
| FGF10 | 2255 | fibroblast growth factor 10 | - | 5 | Cytokines |
| FGF11 | 2256 | fibroblast growth factor 11 | FHF3\|FLJ16061\|MGC102953\|MGC45269 | 17 | Cytokines |
| FGF12 | 2257 | fibroblast growth factor 12 | FGF12B\|FHF1 | 3 | Cytokines |
| FGF13 | 2258 | fibroblast growth factor 13 | FGF2\|FHF-2\|FHF2 | X | Cytokines |
| FGF14 | 2259 | fibroblast growth factor 14 | FHF4\|MGC119129\|SCA27 | 13 | Cytokines |
| FGF16 | 8823 | fibroblast growth factor 16 | - | X | Cytokines |
| FGF17 | 8822 | fibroblast growth factor 17 | FGF-13 | 8 | Cytokines |
| FGF18 | 8817 | fibroblast growth factor 18 | FGF-18\|ZFGF5 | 5 | Cytokines |
| FGF19 | 9965 | fibroblast growth factor 19 | - | 11 | Cytokines |
| FGF2 | 2247 | fibroblast growth factor 2 (basic) | BFGF\|FGFB\|HBGF-2 | 4 | Cytokines |
| FGF20 | 26281 | fibroblast growth factor 20 | - | 8 | Cytokines |
| FGF21 | 26291 | fibroblast growth factor 21 | - | 19 | Cytokines |
| FGF22 | 27006 | fibroblast growth factor 22 | - | 19 | Cytokines |
| FGF23 | 8074 | fibroblast growth factor 23 | ADHR\|HPDR2\|HYPF\|PHPTC | 12 | Cytokines |
| FGF3 | 2248 | fibroblast growth factor 3 (murine mammary tumor virus integration site (v-int-2) oncogene homolog) | HBGF-3\|INT2 | 11 | Cytokines |
| FGF4 | 2249 | fibroblast growth factor 4 | HBGF-4\|HST\|HST-1\|HSTF1\|K-FGF\|KFGF | 11 | Cytokines |
| FGF5 | 2250 | fibroblast growth factor 5 | HBGF-5\|Smag-82 | 4 | Cytokines |
| FGF6 | 2251 | fibroblast growth factor 6 | HBGF-6\|HST2 | 12 | Cytokines |
| FGF7 | 2252 | fibroblast growth factor 7 (keratinocyte growth factor) | HBGF-7\|KGF | 15 | Cytokines |
| FGF8 | 2253 | fibroblast growth factor 8 (androgen-induced) | AIGF\|HBGF-8\|KAL6\|MGC149376 | 10 | Cytokines |
| FGF9 | 2254 | fibroblast growth factor 9 (glia-activating factor) | GAF\|HBFG-9\|MGC119914\|MGC119915 | 13 | Cytokines |
| FIGF | 2277 | c-fos induced growth factor (vascular endothelial growth factor D) | VEGF-D\|VEGFD | X | Cytokines |
| FIGNL2 | 401720 | fidgetin-like 2 | - | 12 | Cytokines |
| FLT3LG | 2323 | fms-related tyrosine kinase 3 ligand | FL | 19 | Cytokines |
| FSHB | 2488 | follicle stimulating hormone, beta polypeptide | - | 11 | Cytokines |
| GAL | 51083 | galanin prepropeptide | GALN\|GLNN\|GMAP\|MGC40167 | 11 | Cytokines |
| GALP | 85569 | galanin-like peptide | - | 19 | Cytokines |
| GAST | 2520 | gastrin | GAS | 17 | Cytokines |
| GCG | 2641 | glucagon | GLP1\|GLP2\|GRPP | 2 | Cytokines |
| GDF1 | 2657 | growth differentiation factor 1 | - | 19 | Cytokines |
| GDF10 | 2662 | growth differentiation factor 10 | BMP-3b\|BMP3B | 10 | Cytokines |
| GDF11 | 10220 | growth differentiation factor 11 | BMP-11\|BMP11 | 12 | Cytokines |
| GDF15 | 9518 | growth differentiation factor 15 | GDF-15\|MIC-1\|MIC1\|NAG-1\|PDF\|PLAB\|PTGFB | 19 | Cytokines |
| GDF2 | 2658 | growth differentiation factor 2 | BMP-9\|BMP9 | 10 | Cytokines |
| GDF3 | 9573 | growth differentiation factor 3 | - | 12 | Cytokines |
| GDF5 | 8200 | growth differentiation factor 5 | BMP14\|CDMP1\|LAP4\|OS5\|SYNS2 | 20 | Cytokines |
| GDF6 | 392255 | growth differentiation factor 6 | BMP13\|CDMP2\|KFS\|KFSL\|MGC158100\|MGC158101\|SGM1 | 8 | Cytokines |
| GDF7 | 151449 | growth differentiation factor 7 | BMP12 | 2 | Cytokines |
| GDF9 | 2661 | growth differentiation factor 9 | - | 5 | Cytokines |
| GDNF | 2668 | glial cell derived neurotrophic factor | ATF1\|ATF2\|HFB1-GDNF | 5 | Cytokines |
| GH1 | 2688 | growth hormone 1 | GH\|GH-N\|GHN\|hGH-N | 17 | Cytokines |
| GH2 | 2689 | growth hormone 2 | GH-V\|GHL\|GHV\|hGH-V | 17 | Cytokines |
| GHRH | 2691 | growth hormone releasing hormone | GHRF\|GRF\|MGC119781 | 20 | Cytokines |
| GHRL | 51738 | ghrelin/obestatin prepropeptide | MTLRP\|obestatin | 3 | Cytokines |
| GIP | 2695 | gastric inhibitory polypeptide | - | 17 | Cytokines |
| GKN1 | 56287 | gastrokine 1 | AMP18\|BRICD1\|CA11\|FOV\|MGC70354\|foveolin | 2 | Cytokines |
| GMFB | 2764 | glia maturation factor, beta | GMF | 14 | Cytokines |
| GMFG | 9535 | glia maturation factor, gamma | GMF-GAMMA\|MGC126867 | 19 | Cytokines |
| GNRH1 | 2796 | gonadotropin-releasing hormone 1 (luteinizing-releasing hormone) | GNRH\|GRH\|LHRH\|LNRH | 8 | Cytokines |
| GNRH2 | 2797 | gonadotropin-releasing hormone 2 | GnRH-II\|LH-RHII | 20 | Cytokines |
| GPHA2 | 170589 | glycoprotein hormone alpha 2 | A2\|GPA2\|MGC126572\|ZSIG51 | 11 | Cytokines |
| GPHB5 | 122876 | glycoprotein hormone beta 5 | B5\|GPB5\|ZLUT1 | 14 | Cytokines |
| GPI | 2821 | glucose phosphate isomerase | AMF\|GNPI\|NLK\|PGI\|PHI\|SA-36 | 19 | Cytokines |
| GREM1 | 26585 | gremlin 1, cysteine knot superfamily, homolog (Xenopus laevis) | CKTSF1B1\|DAND2\|DRM\|GREMLIN\|IHG-2\|MGC126660\|PIG2 | 15 | Cytokines |
| GREM2 | 64388 | gremlin 2, cysteine knot superfamily, homolog (Xenopus laevis) | CKTSF1B2\|DAND3\|PRDC | 1 | Cytokines |
| GRN | 2896 | granulin | GEP\|GP88\|PCDGF\|PEPI\|PGRN | 17 | Cytokines |
| GRP | 2922 | gastrin-releasing peptide | BN\|GRP-10\|preproGRP\|proGRP | 18 | Cytokines |
| GUCA2A | 2980 | guanylate cyclase activator 2A (guanylin) | GUANYLIN\|GUCA2\|STARA | 1 | Cytokines |
| HAMP | 57817 | hepcidin antimicrobial peptide | HEPC\|HEPCIDIN\|HFE2B\|LEAP-1\|LEAP1\|PLTR | 19 | Cytokines |
| HBEGF | 1839 | heparin-binding EGF-like growth factor | DTR\|DTS\|DTSF\|HEGFL | 5 | Cytokines |
| HDGF | 3068 | hepatoma-derived growth factor (high-mobility group protein 1-like) | DKFZp686J1764\|FLJ96580\|HMG1L2 | 1 | Cytokines |
| HDGFRP3 | 50810 | hepatoma-derived growth factor, related protein 3 | CGI-142\|HDGF2 | 15 | Cytokines |
| HGF | 3082 | hepatocyte growth factor (hepapoietin A; scatter factor) | F-TCF\|HGFB\|HPTA\|SF | 7 | Cytokines |
| HTN3 | 3347 | histatin 3 | HIS2\|HTN2\|HTN5 | 4 | Cytokines |
| IAPP | 3375 | islet amyloid polypeptide | AMYLIN\|DAP\|IAP | 12 | Cytokines |
| IFNA1 | 3439 | interferon, alpha 1 | IFL\|IFN\|IFN-ALPHA\|IFNA13\|IFNA@\|MGC138207\|MGC138505\|MGC138507 | 9 | Cytokines |
| IFNA10 | 3446 | interferon, alpha 10 | MGC119878\|MGC119879 | 9 | Cytokines |
| IFNA13 | 3447 | interferon, alpha 13 | - | 9 | Cytokines |
| IFNA14 | 3448 | interferon, alpha 14 | LEIF2H\|MGC125756\|MGC125757 | 9 | Cytokines |
| IFNA16 | 3449 | interferon, alpha 16 | - | 9 | Cytokines |
| IFNA17 | 3451 | interferon, alpha 17 | IFNA\|INFA\|LEIF2C1 | 9 | Cytokines |
| IFNA2 | 3440 | interferon, alpha 2 | IFNA\|INFA2\|MGC125764\|MGC125765 | 9 | Cytokines |
| IFNA21 | 3452 | interferon, alpha 21 | MGC126687\|MGC126689 | 9 | Cytokines |
| IFNA4 | 3441 | interferon, alpha 4 | INFA4\|MGC142200 | 9 | Cytokines |
| IFNA5 | 3442 | interferon, alpha 5 | INFA5 | 9 | Cytokines |
| IFNA6 | 3443 | interferon, alpha 6 | - | 9 | Cytokines |
| IFNA7 | 3444 | interferon, alpha 7 | IFNA-J | 9 | Cytokines |
| IFNA8 | 3445 | interferon, alpha 8 | - | 9 | Cytokines |
| IFNB1 | 3456 | interferon, beta 1, fibroblast | IFB\|IFF\|IFNB\|MGC96956 | 9 | Cytokines |
| IFNE | 338376 | interferon, epsilon | IFN-E\|IFNE1\|IFNT1\|MGC119018\|MGC119020\|PRO655 | 9 | Cytokines |
| IFNG | 3458 | interferon, gamma | IFG\|IFI | 12 | Cytokines |
| IFNK | 56832 | interferon, kappa | RP11-27J8.1 | 9 | Cytokines |
| IFNW1 | 3467 | interferon, omega 1 | - | 9 | Cytokines |
| IGF1 | 3479 | insulin-like growth factor 1 (somatomedin C) | IGF1A\|IGFI | 12 | Cytokines |
| IGF2 | 3481 | insulin-like growth factor 2 (somatomedin A) | C11orf43\|FLJ22066\|FLJ44734\|INSIGF\|pp9974 | 11 | Cytokines |
| IL10 | 3586 | interleukin 10 | CSIF\|IL-10\|IL10A\|MGC126450\|MGC126451\|TGIF | 1 | Cytokines |
| IL11 | 3589 | interleukin 11 | AGIF\|IL-11 | 19 | Cytokines |
| IL12A | 3592 | interleukin 12A (natural killer cell stimulatory factor 1, cytotoxic lymphocyte maturation factor 1, p35) | CLMF\|IL-12A\|NFSK\|NKSF1\|P35 | 3 | Cytokines |
| IL12B | 3593 | interleukin 12B (natural killer cell stimulatory factor 2, cytotoxic lymphocyte maturation factor 2, p40) | CLMF\|CLMF2\|IL-12B\|NKSF\|NKSF2 | 5 | Cytokines |
| IL13 | 3596 | interleukin 13 | ALRH\|BHR1\|IL-13\|MGC116786\|MGC116788\|MGC116789\|P600 | 5 | Cytokines |
| IL15 | 3600 | interleukin 15 | IL-15\|MGC9721 | 4 | Cytokines |
| IL16 | 3603 | interleukin 16 (lymphocyte chemoattractant factor) | FLJ16806\|FLJ42735\|FLJ44234\|HsT19289\|IL-16\|LCF\|prIL-16 | 15 | Cytokines |
| IL17A | 3605 | interleukin 17A | CTLA8\|IL-17\|IL-17A\|IL17 | 6 | Cytokines |
| IL17B | 27190 | interleukin 17B | IL-17B\|IL-20\|MGC138900\|MGC138901\|ZCYTO7 | 5 | Cytokines |
| IL17C | 27189 | interleukin 17C | CX2\|IL-17C\|IL-21\|MGC126884\|MGC138401 | 16 | Cytokines |
| IL17D | 53342 | interleukin 17D | FLJ30846\|IL-17D\|IL-22\|IL-27\|IL27 | 13 | Cytokines |
| IL17F | 112744 | interleukin 17F | IL-17F\|ML-1\|ML1 | 6 | Cytokines |
| IL18 | 3606 | interleukin 18 (interferon-gamma-inducing factor) | IGIF\|IL-18\|IL-1g\|IL1F4\|MGC12320 | 11 | Cytokines |
| IL19 | 29949 | interleukin 19 | IL-10C\|MDA1\|NG.1\|ZMDA1 | 1 | Cytokines |
| IL1A | 3552 | interleukin 1, alpha | IL-1A\|IL1\|IL1-ALPHA\|IL1F1 | 2 | Cytokines |
| IL1B | 3553 | interleukin 1, beta | IL-1\|IL1-BETA\|IL1F2 | 2 | Cytokines |
| IL1F10 | 84639 | interleukin 1 family, member 10 (theta) | FIL1-theta\|FKSG75\|IL-1HY2\|IL1-theta\|MGC119831\|MGC119832\|MGC119833 | 2 | Cytokines |
| IL1F5 | 26525 | interleukin 1 family, member 5 (delta) | FIL1\|FIL1(DELTA)\|FIL1D\|IL1HY1\|IL1L1\|IL1RP3\|MGC29840 | 2 | Cytokines |
| IL1F6 | 27179 | interleukin 1 family, member 6 (epsilon) | FIL1\|FIL1(EPSILON)\|FIL1E\|IL-1F6\|IL1(EPSILON)\|MGC129552\|MGC129553 | 2 | Cytokines |
| IL1F7 | 27178 | interleukin 1 family, member 7 (zeta) | FIL1\|FIL1(ZETA)\|FIL1Z\|IL-1F7\|IL-1H4\|IL-1RP1\|IL1H4\|IL1RP1 | 2 | Cytokines |
| IL1F8 | 27177 | interleukin 1 family, member 8 (eta) | FIL1\|FIL1-(ETA)\|FIL1H\|IL-1F8\|IL-1H2\|IL1-ETA\|IL1H2\|MGC126880\|MGC126882 | 2 | Cytokines |
| IL1F9 | 56300 | interleukin 1 family, member 9 | IL-1F9\|IL-1H1\|IL-1RP2\|IL1E\|IL1H1\|IL1RP2 | 2 | Cytokines |
| IL1RN | 3557 | interleukin 1 receptor antagonist | ICIL-1RA\|IL-1ra3\|IL1F3\|IL1RA\|IRAP\|MGC10430 | 2 | Cytokines |
| IL2 | 3558 | interleukin 2 | IL-2\|TCGF\|lymphokine | 4 | Cytokines |
| IL20 | 50604 | interleukin 20 | IL-20\|IL10D\|MGC96907\|ZCYTO10 | 1 | Cytokines |
| IL21 | 59067 | interleukin 21 | IL-21\|Za11 | 4 | Cytokines |
| IL22 | 50616 | interleukin 22 | IL-21\|IL-22\|IL-D110\|IL-TIF\|IL21\|ILTIF\|MGC79382\|MGC79384\|TIFIL-23\|TIFa\|zcyto18 | 12 | Cytokines |
| IL23A | 51561 | interleukin 23, alpha subunit p19 | IL-23\|IL-23A\|IL23P19\|MGC79388\|P19\|SGRF | 12 | Cytokines |
| IL24 | 11009 | interleukin 24 | C49A\|FISP\|IL-24\|IL10B\|MDA7\|Mob-5\|ST16\|mda-7 | 1 | Cytokines |
| IL25 | 64806 | interleukin 25 | IL-17E\|IL-25\|IL17E | 14 | Cytokines |
| IL26 | 55801 | interleukin 26 | AK155\|IL-26 | 12 | Cytokines |
| IL27 | 246778 | interleukin 27 | IL-27\|IL-27A\|IL27p28\|IL30\|MGC71873\|p28 | 16 | Cytokines |
| IL28A | 282616 | interleukin 28A (interferon, lambda 2) | IFNL2\|IL-28A | 19 | Cytokines |
| IL28B | 282617 | interleukin 28B (interferon, lambda 3) | IFNL3\|IL-28B\|IL28C | 19 | Cytokines |
| IL29 | 282618 | interleukin 29 (interferon, lambda 1) | IFNL1\|IL-29 | 19 | Cytokines |
| IL3 | 3562 | interleukin 3 (colony-stimulating factor, multiple) | IL-3\|MCGF\|MGC79398\|MGC79399\|MULTI-CSF | 5 | Cytokines |
| IL31 | 386653 | interleukin 31 | IL-31 | 12 | Cytokines |
| IL32 | 9235 | interleukin 32 | IL-32alpha\|IL-32beta\|IL-32delta\|IL-32gamma\|NK4\|TAIF\|TAIFa\|TAIFb\|TAIFc\|TAIFd | 16 | Cytokines |
| IL33 | 90865 | interleukin 33 | C9orf26\|DKFZp586H0523\|DVS27\|NF-HEV\|NFEHEV\|RP11-575C20.2 | 9 | Cytokines |
| IL34 | 146433 | interleukin 34 | C16orf77\|IL-34\|MGC34647 | 16 | Cytokines |
| IL4 | 3565 | interleukin 4 | BCGF-1\|BCGF1\|BSF1\|IL-4\|MGC79402 | 5 | Cytokines |
| IL5 | 3567 | interleukin 5 (colony-stimulating factor, eosinophil) | EDF\|IL-5\|TRF | 5 | Cytokines |
| IL6 | 3569 | interleukin 6 (interferon, beta 2) | BSF2\|HGF\|HSF\|IFNB2\|IL-6 | 7 | Cytokines |
| IL6ST | 3572 | interleukin 6 signal transducer (gp130, oncostatin M receptor) | CD130\|CDw130\|GP130\|GP130-RAPS\|IL6R-beta | 5 | Cytokines |
| IL7 | 3574 | interleukin 7 | IL-7 | 8 | Cytokines |
| IL8 | 3576 | interleukin 8 | CXCL8\|GCP-1\|GCP1\|LECT\|LUCT\|LYNAP\|MDNCF\|MONAP\|NAF\|NAP-1\|NAP1 | 4 | Cytokines |
| IL9 | 3578 | interleukin 9 | HP40\|IL-9\|P40 | 5 | Cytokines |
| INHA | 3623 | inhibin, alpha | - | 2 | Cytokines |
| INHBA | 3624 | inhibin, beta A | EDF\|FRP | 7 | Cytokines |
| INHBB | 3625 | inhibin, beta B | MGC157939 | 2 | Cytokines |
| INHBC | 3626 | inhibin, beta C | IHBC | 12 | Cytokines |
| INHBE | 83729 | inhibin, beta E | MGC4638 | 12 | Cytokines |
| INS | 3630 | insulin | ILPR\|IRDN | 11 | Cytokines |
| INS-IGF2 | 723961 | INS-IGF2 readthrough transcript | - | 11 | Cytokines |
| INSL3 | 3640 | insulin-like 3 (Leydig cell) | MGC119818\|MGC119819\|RLF\|RLNL | 19 | Cytokines |
| INSL4 | 3641 | insulin-like 4 (placenta) | EPIL\|PLACENTIN | 9 | Cytokines |
| INSL5 | 10022 | insulin-like 5 | MGC126695\|MGC126697\|PRO182\|UNQ156 | 1 | Cytokines |
| INSL6 | 11172 | insulin-like 6 | RIF1 | 9 | Cytokines |
| JAG1 | 182 | jagged 1 (Alagille syndrome) | AGS\|AHD\|AWS\|CD339\|HJ1\|JAGL1\|MGC104644 | 20 | Cytokines |
| JAG2 | 3714 | jagged 2 | HJ2\|SER2 | 14 | Cytokines |
| KGFLP1 | 387628 | keratinocyte growth factor-like protein 1 | MGC125746\|MGC125747\|MGC126891 | 9 | Cytokines |
| KGFLP2 | 654466 | keratinocyte growth factor-like protein 2 | - | 9 | Cytokines |
| KITLG | 4254 | KIT ligand | DKFZp686F2250\|KL-1\|Kitl\|MGF\|SCF\|SF\|SHEP7 | 12 | Cytokines |
| KL | 9365 | klotho | - | 13 | Cytokines |
| LACRT | 90070 | lacritin | MGC71934 | 12 | Cytokines |
| LECT2 | 3950 | leukocyte cell-derived chemotaxin 2 | MGC126628\|chm-II\|chm2 | 5 | Cytokines |
| LEFTY1 | 10637 | left-right determination factor 1 | LEFTB\|LEFTYB | 1 | Cytokines |
| LEFTY2 | 7044 | left-right determination factor 2 | EBAF\|LEFTA\|LEFTYA\|MGC46222\|TGFB4 | 1 | Cytokines |
| LEP | 3952 | leptin | FLJ94114\|OB\|OBS | 7 | Cytokines |
| LHB | 3972 | luteinizing hormone beta polypeptide | CGB4\|LSH-B\|hLHB | 19 | Cytokines |
| LIF | 3976 | leukemia inhibitory factor (cholinergic differentiation factor) | CDF\|DIA\|HILDA | 22 | Cytokines |
| LRSAM1 | 90678 | leucine rich repeat and sterile alpha motif containing 1 | FLJ31641\|RIFLE\|TAL | 9 | Cytokines |
| LTA | 4049 | lymphotoxin alpha (TNF superfamily, member 1) | LT\|TNFB\|TNFSF1 | 6 | Cytokines |
| LTB | 4050 | lymphotoxin beta (TNF superfamily, member 3) | TNFC\|TNFSF3\|p33 | 6 | Cytokines |
| LTBP1 | 4052 | latent transforming growth factor beta binding protein 1 | MGC163161 | 2 | Cytokines |
| LTBP2 | 4053 | latent transforming growth factor beta binding protein 2 | C14orf141\|LTBP3\|MSTP031 | 14 | Cytokines |
| LTBP3 | 4054 | latent transforming growth factor beta binding protein 3 | DKFZp586M2123\|FLJ33431\|FLJ39893\|FLJ42533\|FLJ44138\|FLJ45576\|LTBP-3\|LTBP2\|pp6425 | 11 | Cytokines |
| LTBP4 | 8425 | latent transforming growth factor beta binding protein 4 | FLJ46318\|FLJ90018\|LTBP-4\|LTBP-4L | 19 | Cytokines |
| MDK | 4192 | midkine (neurite growth-promoting factor 2) | FLJ27379\|MK\|NEGF2 | 11 | Cytokines |
| MIA | 8190 | melanoma inhibitory activity | CD-RAP | 19 | Cytokines |
| MIF | 4282 | macrophage migration inhibitory factor (glycosylation-inhibiting factor) | GIF\|GLIF\|MMIF | 22 | Cytokines |
| MLN | 4295 | motilin | MGC138519 | 6 | Cytokines |
| MSTN | 2660 | myostatin | GDF8 | 2 | Cytokines |
| NAMPT | 10135 | nicotinamide phosphoribosyltransferase | 1110035O14Rik\|DKFZp666B131\|MGC117256\|PBEF\|PBEF1\|VF\|VISFATIN | 7 | Cytokines |
| NDP | 4693 | Norrie disease (pseudoglioma) | EVR2\|FEVR\|ND | X | Cytokines |
| NENF | 29937 | neuron derived neurotrophic factor | CIR2\|NEUDESIN\|SCIRP10\|SPUF | 1 | Cytokines |
| NGF | 4803 | nerve growth factor (beta polypeptide) | Beta-NGF\|HSAN5\|MGC161426\|MGC161428\|NGFB | 1 | Cytokines |
| NMB | 4828 | neuromedin B | MGC17211\|MGC2277\|MGC3936 | 15 | Cytokines |
| NODAL | 4838 | nodal homolog (mouse) | MGC138230 | 10 | Cytokines |
| NOV | 4856 | nephroblastoma overexpressed gene | CCN3\|IGFBP9 | 8 | Cytokines |
| NPFF | 8620 | neuropeptide FF-amide peptide precursor | FMRFAL | 12 | Cytokines |
| NPPA | 4878 | natriuretic peptide precursor A | ANF\|ANP\|ATFB6\|CDD-ANF\|PND | 1 | Cytokines |
| NPPB | 4879 | natriuretic peptide precursor B | BNP | 1 | Cytokines |
| NPPC | 4880 | natriuretic peptide precursor C | CNP | 2 | Cytokines |
| NPY | 4852 | neuropeptide Y | PYY4 | 7 | Cytokines |
| NRG1 | 3084 | neuregulin 1 | ARIA\|GGF\|GGF2\|HGL\|HRG\|HRG1\|HRGA\|NDF\|SMDF | 8 | Cytokines |
| NRG2 | 9542 | neuregulin 2 | Don-1\|HRG2\|NTAK | 5 | Cytokines |
| NRG3 | 10718 | neuregulin 3 | HRG3\|pro-NRG3 | 10 | Cytokines |
| NRG4 | 145957 | neuregulin 4 | DKFZp779N0541\|DKFZp779N1944\|HRG4 | 15 | Cytokines |
| NRTN | 4902 | neurturin | NTN | 19 | Cytokines |
| NTF3 | 4908 | neurotrophin 3 | HDNF\|MGC129711\|NGF-2\|NGF2\|NT3 | 12 | Cytokines |
| NTF4 | 4909 | neurotrophin 4 | NT-4/5\|NT4\|NT5\|NTF5 | 19 | Cytokines |
| NTS | 4922 | neurotensin | NMN-125\|NN\|NT\|NT/N\|NTS1 | 12 | Cytokines |
| NUDT6 | 11162 | nudix (nucleoside diphosphate linked moiety X)-type motif 6 | ASFGF2\|FGF-2\|FGF-AS\|FGF2AS\|bFGF\|gfg\|gfg-1 | 4 | Cytokines |
| OGN | 4969 | osteoglycin | DKFZp586P2421\|OG\|OIF\|SLRR3A | 9 | Cytokines |
| OSGIN1 | 29948 | oxidative stress induced growth inhibitor 1 | BDGI\|OKL38 | 16 | Cytokines |
| OSM | 5008 | oncostatin M | MGC20461 | 22 | Cytokines |
| OSTN | 344901 | osteocrin | MUSCLIN | 3 | Cytokines |
| OXT | 5020 | oxytocin, prepropeptide | MGC126890\|MGC126892\|OT\|OT-NPI | 20 | Cytokines |
| P11 | 8909 | 26 serine protease | MGC133268\|PP11\|PRSS26 | 12 | Cytokines |
| PDGFA | 5154 | platelet-derived growth factor alpha polypeptide | PDGF-A\|PDGF1 | 7 | Cytokines |
| PDGFB | 5155 | platelet-derived growth factor beta polypeptide (simian sarcoma viral (v-sis) oncogene homolog) | FLJ12858\|PDGF2\|SIS\|SSV\|c-sis | 22 | Cytokines |
| PDGFC | 56034 | platelet derived growth factor C | FALLOTEIN\|SCDGF | 4 | Cytokines |
| PDGFD | 80310 | platelet derived growth factor D | IEGF\|MGC26867\|MSTP036\|SCDGF-B\|SCDGFB | 11 | Cytokines |
| PDGFRA | 5156 | platelet-derived growth factor receptor, alpha polypeptide | CD140A\|MGC74795\|PDGFR2\|Rhe-PDGFRA | 4 | Cytokines |
| PDGFRB | 5159 | platelet-derived growth factor receptor, beta polypeptide | CD140B\|JTK12\|PDGF-R-beta\|PDGFR\|PDGFR1 | 5 | Cytokines |
| PDGFRL | 5157 | platelet-derived growth factor receptor-like | PDGRL\|PRLTS | 8 | Cytokines |
| PDYN | 5173 | prodynorphin | MGC26418\|PENKB | 20 | Cytokines |
| PENK | 5179 | proenkephalin | - | 8 | Cytokines |
| PF4 | 5196 | platelet factor 4 | CXCL4\|MGC138298\|SCYB4 | 4 | Cytokines |
| PF4V1 | 5197 | platelet factor 4 variant 1 | CXCL4L1\|CXCL4V1\|PF4-ALT\|PF4A\|SCYB4V1 | 4 | Cytokines |
| PGF | 5228 | placental growth factor | D12S1900\|PGFL\|PLGF\|PlGF-2\|SHGC-10760 | 14 | Cytokines |
| PLAU | 5328 | plasminogen activator, urokinase | ATF\|UPA\|URK\|u-PA | 10 | Cytokines |
| PMCH | 5367 | pro-melanin-concentrating hormone | MCH | 12 | Cytokines |
| PNOC | 5368 | prepronociceptin | PPNOC | 8 | Cytokines |
| POMC | 5443 | proopiomelanocortin | ACTH\|CLIP\|LPH\|MSH\|NPP\|POC | 2 | Cytokines |
| PPBP | 5473 | pro-platelet basic protein (chemokine (C-X-C motif) ligand 7) | B-TG1\|Beta-TG\|CTAP-III\|CTAP3\|CTAPIII\|CXCL7\|LA-PF4\|LDGF\|MDGF\|NAP-2\|PBP\|SCYB7\|TC1\|TC2\|TGB\|TGB1\|THBGB\|THBGB1 | 4 | Cytokines |
| PPBPL1 | 728045 | pro-platelet basic protein-like 1 | TGB2 | 4 | Cytokines |
| PPBPL2 | 10895 | pro-platelet basic protein-like 2 | SPBPBP | 4 | Cytokines |
| PPY | 5539 | pancreatic polypeptide | PNP | 17 | Cytokines |
| PRL | 5617 | prolactin | - | 6 | Cytokines |
| PRLH | 51052 | prolactin releasing hormone | PRH\|PRRP | 2 | Cytokines |
| PROK1 | 84432 | prokineticin 1 | EGVEGF\|PK1\|PRK1 | 1 | Cytokines |
| PROK2 | 60675 | prokineticin 2 | BV8\|KAL4\|MIT1\|PK2 | 3 | Cytokines |
| PSPN | 5623 | persephin | PSP | 19 | Cytokines |
| PTH | 5741 | parathyroid hormone | PTH1 | 11 | Cytokines |
| PTH2 | 113091 | parathyroid hormone 2 | TIP39 | 19 | Cytokines |
| PTHLH | 5744 | parathyroid hormone-like hormone | HHM\|MGC14611\|PLP\|PTHR\|PTHRP | 12 | Cytokines |
| PTN | 5764 | pleiotrophin | HARP\|HBGF8\|HBNF\|NEGF1 | 7 | Cytokines |
| PYY | 5697 | peptide YY | PYY1 | 17 | Cytokines |
| QRFP | 347148 | pyroglutamylated RFamide peptide | 26RFa\|MGC119794\|P518 | 9 | Cytokines |
| RABEP1 | 9135 | rabaptin, RAB GTPase binding effector protein 1 | RAB5EP\|RABPT5 | 17 | Cytokines |
| RABEP2 | 79874 | rabaptin, RAB GTPase binding effector protein 2 | FLJ23282\|FRA | 16 | Cytokines |
| REG1A | 5967 | regenerating islet-derived 1 alpha | ICRF\|MGC12447\|P19\|PSP\|PSPS\|PSPS1\|PTP\|REG | 2 | Cytokines |
| RETN | 56729 | resistin | ADSF\|FIZZ3\|MGC126603\|MGC126609\|RETN1\|RSTN\|XCP1 | 19 | Cytokines |
| RETNLB | 84666 | resistin like beta | FIZZ1\|FIZZ2\|HXCP2\|RELM-beta\|RELMb\|RELMbeta\|XCP2 | 3 | Cytokines |
| RLN1 | 6013 | relaxin 1 | H1\|RLXH1\|bA12D24.3.1\|bA12D24.3.2 | 9 | Cytokines |
| RLN2 | 6019 | relaxin 2 | H2\|RLXH2\|bA12D24.1.1\|bA12D24.1.2 | 9 | Cytokines |
| RLN3 | 117579 | relaxin 3 | H3\|RXN3\|ZINS4\|insl7 | 19 | Cytokines |
| RNASE2 | 6036 | ribonuclease, RNase A family, 2 (liver, eosinophil-derived neurotoxin) | EDN\|RNS2 | 14 | Cytokines |
| S100A6 | 6277 | S100 calcium binding protein A6 | 2A9\|5B10\|CABP\|CACY\|PRA | 1 | Cytokines |
| SAA1 | 6288 | serum amyloid A1 | MGC111216\|PIG4\|SAA\|TP53I4 | 11 | Cytokines |
| SAA2 | 6289 | serum amyloid A2 | - | 11 | Cytokines |
| SBDS | 51119 | Shwachman-Bodian-Diamond syndrome | CGI-97\|FLJ10917\|SDS\|SWDS | 7 | Cytokines |
| SCG2 | 7857 | secretogranin II (chromogranin C) | CHGC\|SN\|SgII | 2 | Cytokines |
| SCGB3A1 | 92304 | secretoglobin, family 3A, member 1 | HIN-1\|HIN1\|LU105\|MGC87867\|PnSP-2\|UGRP2 | 5 | Cytokines |
| SCT | 6343 | secretin | - | 11 | Cytokines |
| SCYE1 | 9255 | small inducible cytokine subfamily E, member 1 (endothelial monocyte-activating) | AIMP1\|EMAP2\|EMAPII\|p43 | 4 | Cytokines |
| SECTM1 | 6398 | secreted and transmembrane 1 | K12 | 17 | Cytokines |
| SEMA3A | 10371 | sema domain, immunoglobulin domain (Ig), short basic domain, secreted, (semaphorin) 3A | Hsema-I\|Hsema-III\|MGC133243\|SEMA1\|SEMAD\|SEMAIII\|SEMAL\|SemD\|coll-1 | 7 | Cytokines |
| SEMA3B | 7869 | sema domain, immunoglobulin domain (Ig), short basic domain, secreted, (semaphorin) 3B | FLJ34863\|LUCA-1\|SEMA5\|SEMAA\|SemA\|semaV | 3 | Cytokines |
| SEMA3C | 10512 | sema domain, immunoglobulin domain (Ig), short basic domain, secreted, (semaphorin) 3C | SEMAE\|SemE | 7 | Cytokines |
| SEMA3D | 223117 | sema domain, immunoglobulin domain (Ig), short basic domain, secreted, (semaphorin) 3D | MGC39708\|Sema-Z2\|coll-2 | 7 | Cytokines |
| SEMA3E | 9723 | sema domain, immunoglobulin domain (Ig), short basic domain, secreted, (semaphorin) 3E | KIAA0331\|M-SEMAH\|M-SemaK\|SEMAH\|coll-5 | 7 | Cytokines |
| SEMA3F | 6405 | sema domain, immunoglobulin domain (Ig), short basic domain, secreted, (semaphorin) 3F | SEMA-IV\|SEMA4\|SEMAK | 3 | Cytokines |
| SEMA3G | 56920 | sema domain, immunoglobulin domain (Ig), short basic domain, secreted, (semaphorin) 3G | FLJ00014\|MGC119473\|sem2 | 3 | Cytokines |
| SEMA4A | 64218 | sema domain, immunoglobulin domain (Ig), transmembrane domain (TM) and short cytoplasmic domain, (semaphorin) 4A | CORD10\|FLJ12287\|RP35\|SEMAB\|SEMB | 1 | Cytokines |
| SEMA4B | 10509 | sema domain, immunoglobulin domain (Ig), transmembrane domain (TM) and short cytoplasmic domain, (semaphorin) 4B | KIAA1745\|MGC131831\|SEMAC\|SemC | 15 | Cytokines |
| SEMA4C | 54910 | sema domain, immunoglobulin domain (Ig), transmembrane domain (TM) and short cytoplasmic domain, (semaphorin) 4C | FLJ20369\|KIAA1739\|M-SEMA-F\|MGC126382\|MGC126383\|SEMACL1\|SEMAF\|SEMAI | 2 | Cytokines |
| SEMA4D | 10507 | sema domain, immunoglobulin domain (Ig), transmembrane domain (TM) and short cytoplasmic domain, (semaphorin) 4D | C9orf164\|CD100\|FLJ33485\|FLJ34282\|FLJ39737\|FLJ46484\|M-sema-G\|MGC169138\|MGC169141\|SEMAJ\|coll-4 | 9 | Cytokines |
| SEMA4F | 10505 | sema domain, immunoglobulin domain (Ig), transmembrane domain (TM) and short cytoplasmic domain, (semaphorin) 4F | M-SEMA\|PRO2353\|SEMAM\|SEMAW\|m-Sema-M | 2 | Cytokines |
| SEMA4G | 57715 | sema domain, immunoglobulin domain (Ig), transmembrane domain (TM) and short cytoplasmic domain, (semaphorin) 4G | FLJ20590\|KIAA1619\|MGC102867 | 10 | Cytokines |
| SEMA5A | 9037 | sema domain, seven thrombospondin repeats (type 1 and type 1-like), transmembrane domain (TM) and short cytoplasmic domain, (semaphorin) 5A | FLJ12815\|SEMAF\|semF | 5 | Cytokines |
| SEMA5B | 54437 | sema domain, seven thrombospondin repeats (type 1 and type 1-like), transmembrane domain (TM) and short cytoplasmic domain, (semaphorin) 5B | FLJ10372\|KIAA1445\|SEMAG\|SemG | 3 | Cytokines |
| SEMA6A | 57556 | sema domain, transmembrane domain (TM), and cytoplasmic domain, (semaphorin) 6A | HT018\|KIAA1368\|SEMA\|SEMA6A1\|SEMAQ\|VIA | 5 | Cytokines |
| SEMA6B | 10501 | sema domain, transmembrane domain (TM), and cytoplasmic domain, (semaphorin) 6B | SEM-SEMA-Y\|SEMA-VIB\|SEMAN\|semaZ | 19 | Cytokines |
| SEMA6C | 10500 | sema domain, transmembrane domain (TM), and cytoplasmic domain, (semaphorin) 6C | SEMAY\|m-SemaY\|m-SemaY2 | 1 | Cytokines |
| SEMA6D | 80031 | sema domain, transmembrane domain (TM), and cytoplasmic domain, (semaphorin) 6D | FLJ11598\|KIAA1479 | 15 | Cytokines |
| SEMA7A | 8482 | semaphorin 7A, GPI membrane anchor (John Milton Hagen blood group) | CD108\|CDw108\|H-SEMA-K1\|H-Sema-L\|JMH\|MGC126692\|MGC126696\|SEMAK1\|SEMAL | 15 | Cytokines |
| SLIT1 | 6585 | slit homolog 1 (Drosophila) | MEGF4\|MGC164811\|SLIL1\|SLIT3\|Slit-1 | 10 | Cytokines |
| SLIT2 | 9353 | slit homolog 2 (Drosophila) | FLJ14420\|SLIL3\|Slit-2 | 4 | Cytokines |
| SLURP1 | 57152 | secreted LY6/PLAUR domain containing 1 | ANUP\|ARS\|ArsB\|LY6LS\|MDM | 8 | Cytokines |
| SPP1 | 6696 | secreted phosphoprotein 1 | BNSP\|BSPI\|ETA-1\|MGC110940\|OPN | 4 | Cytokines |
| SST | 6750 | somatostatin | SMST | 3 | Cytokines |
| STC1 | 6781 | stanniocalcin 1 | STC | 8 | Cytokines |
| STC2 | 8614 | stanniocalcin 2 | STC-2\|STCRP | 5 | Cytokines |
| TAC1 | 6863 | tachykinin, precursor 1 | Hs.2563\|NK2\|NKNA\|NPK\|TAC2 | 7 | Cytokines |
| TDGF1 | 6997 | teratocarcinoma-derived growth factor 1 | CR\|CRGF\|CRIPTO\|Cripto-1 | 3 | Cytokines |
| TDGF3 | 6998 | teratocarcinoma-derived growth factor 3, pseudogene | CR-3\|CRIPTO\|CRIPTO-3\|TDGF1\|TDGF2 | X | Cytokines |
| TG | 7038 | thyroglobulin | AITD3\|TGN | 8 | Cytokines |
| TGFA | 7039 | transforming growth factor, alpha | TFGA | 2 | Cytokines |
| TGFB1 | 7040 | transforming growth factor, beta 1 | CED\|DPD1\|TGFB\|TGFbeta | 19 | Cytokines |
| TGFB2 | 7042 | transforming growth factor, beta 2 | MGC116892\|TGF-beta2 | 1 | Cytokines |
| TGFB3 | 7043 | transforming growth factor, beta 3 | ARVD\|FLJ16571\|TGF-beta3 | 14 | Cytokines |
| THPO | 7066 | thrombopoietin | MGC163194\|MGDF\|MKCSF\|ML\|MPLLG\|TPO | 3 | Cytokines |
| TNC | 3371 | tenascin C | HXB\|MGC167029\|TN | 9 | Cytokines |
| TNF | 7124 | tumor necrosis factor (TNF superfamily, member 2) | DIF\|TNF-alpha\|TNFA\|TNFSF2 | 6 | Cytokines |
| TNFRSF11B | 4982 | tumor necrosis factor receptor superfamily, member 11b | MGC29565\|OCIF\|OPG\|TR1 | 8 | Cytokines |
| TNFSF10 | 8743 | tumor necrosis factor (ligand) superfamily, member 10 | APO2L\|Apo-2L\|CD253\|TL2\|TRAIL | 3 | Cytokines |
| TNFSF11 | 8600 | tumor necrosis factor (ligand) superfamily, member 11 | CD254\|ODF\|OPGL\|OPTB2\|RANKL\|TRANCE\|hRANKL2\|sOdf | 13 | Cytokines |
| TNFSF12 | 8742 | tumor necrosis factor (ligand) superfamily, member 12 | APO3L\|DR3LG\|MGC129581\|MGC20669\|TWEAK | 17 | Cytokines |
| TNFSF13 | 8741 | tumor necrosis factor (ligand) superfamily, member 13 | APRIL\|CD256\|TALL2\|TRDL-1\|UNQ383/PRO715\|ligand | 17 | Cytokines |
| TNFSF13B | 10673 | tumor necrosis factor (ligand) superfamily, member 13b | BAFF\|BLYS\|CD257\|DTL\|TALL-1\|TALL1\|THANK\|TNFSF20\|ZTNF4 | 13 | Cytokines |
| TNFSF14 | 8740 | tumor necrosis factor (ligand) superfamily, member 14 | CD258\|HVEML\|LIGHT\|LTg\|TR2 | 19 | Cytokines |
| TNFSF15 | 9966 | tumor necrosis factor (ligand) superfamily, member 15 | MGC129934\|MGC129935\|TL1\|TL1A\|VEGI\|VEGI192A | 9 | Cytokines |
| TNFSF18 | 8995 | tumor necrosis factor (ligand) superfamily, member 18 | AITRL\|GITRL\|MGC138237\|TL6\|hGITRL | 1 | Cytokines |
| TNFSF4 | 7292 | tumor necrosis factor (ligand) superfamily, member 4 | CD134L\|CD252\|GP34\|OX-40L\|OX4OL\|TXGP1 | 1 | Cytokines |
| TNFSF8 | 944 | tumor necrosis factor (ligand) superfamily, member 8 | CD153\|CD30L\|CD30LG\|MGC138144 | 9 | Cytokines |
| TNFSF9 | 8744 | tumor necrosis factor (ligand) superfamily, member 9 | 4-1BB-L\|CD137L | 19 | Cytokines |
| TOR2A | 27433 | torsin family 2, member A | FLJ14771\|MGC99558\|TORP1 | 9 | Cytokines |
| TRH | 7200 | thyrotropin-releasing hormone | MGC125964\|MGC125965 | 3 | Cytokines |
| TSHB | 7252 | thyroid stimulating hormone, beta | CHNG4\|TSH-BETA | 1 | Cytokines |
| TSLP | 85480 | thymic stromal lymphopoietin | - | 5 | Cytokines |
| TXLNA | 200081 | taxilin alpha | DKFZp451J0118\|IL14\|MGC118870\|MGC118871\|RP4-622L5.4\|TXLN | 1 | Cytokines |
| TYMP | 1890 | thymidine phosphorylase | ECGF1\|MNGIE\|PDECGF\|TP\|hPD-ECGF | 22 | Cytokines |
| UCN | 7349 | urocortin | MGC129974\|MGC129975\|UI\|UROC | 2 | Cytokines |
| UCN2 | 90226 | urocortin 2 | SRP\|UCN-II\|UCNI\|UR\|URP | 3 | Cytokines |
| UCN3 | 114131 | urocortin 3 (stresscopin) | MGC119002\|SCP\|SPC\|UCNIII | 10 | Cytokines |
| UTS2 | 10911 | urotensin 2 | PRO1068\|U-II\|UCN2\|UII | 1 | Cytokines |
| UTS2D | 257313 | urotensin 2 domain containing | MGC138371\|U2B\|URP | 3 | Cytokines |
| VEGFA | 7422 | vascular endothelial growth factor A | MGC70609\|MVCD1\|VEGF\|VEGF-A\|VPF | 6 | Cytokines |
| VEGFB | 7423 | vascular endothelial growth factor B | VEGFL\|VRF | 11 | Cytokines |
| VEGFC | 7424 | vascular endothelial growth factor C | Flt4-L\|VRP | 4 | Cytokines |
| VGF | 7425 | VGF nerve growth factor inducible | - | 7 | Cytokines |
| VIP | 7432 | vasoactive intestinal peptide | MGC13587\|PHM27 | 6 | Cytokines |
| XCL1 | 6375 | chemokine (C motif) ligand 1 | ATAC\|LPTN\|LTN\|SCM-1\|SCM-1a\|SCM1\|SCYC1 | 1 | Cytokines |
| XCL2 | 6846 | chemokine (C motif) ligand 2 | SCM-1b\|SCM1B\|SCYC2 | 1 | Cytokines |
| ACVR1B | 91 | activin A receptor, type IB | ACTRIB\|ACVRLK4\|ALK4\|SKR2 | 12 | Cytokine Receptors |
| ACVR1C | 130399 | activin A receptor, type IC | ACVRLK7\|ALK7 | 2 | Cytokine Receptors |
| ACVR2A | 92 | activin A receptor, type IIA | ACTRII\|ACVR2 | 2 | Cytokine Receptors |
| ACVR2B | 93 | activin A receptor, type IIB | ACTRIIB\|ActR-IIB\|MGC116908 | 3 | Cytokine Receptors |
| ACVRL1 | 94 | activin A receptor type II-like 1 | ACVRLK1\|ALK-1\|ALK1\|HHT\|HHT2\|ORW2\|SKR3\|TSR-I | 12 | Cytokine Receptors |
| ADCYAP1R1 | 117 | adenylate cyclase activating polypeptide 1 (pituitary) receptor type I | PAC1\|PACAPR\|PACAPRI | 7 | Cytokine Receptors |
| ADIPOR1 | 51094 | adiponectin receptor 1 | ACDCR1\|CGI-45\|CGI45\|FLJ25385\|FLJ42464\|PAQR1\|TESBP1A | 1 | Cytokine Receptors |
| ADIPOR2 | 79602 | adiponectin receptor 2 | ACDCR2\|FLJ21432\|MGC4640\|PAQR2 | 12 | Cytokine Receptors |
| ADRB1 | 153 | adrenergic, beta-1-, receptor | ADRB1R\|B1AR\|BETA1AR\|RHR | 10 | Cytokine Receptors |
| ADRB2 | 154 | adrenergic, beta-2-, receptor, surface | ADRB2R\|ADRBR\|B2AR\|BAR\|BETA2AR | 5 | Cytokine Receptors |
| AGTR1 | 185 | angiotensin II receptor, type 1 | AG2S\|AGTR1A\|AGTR1B\|AT1\|AT1B\|AT1R\|AT2R1\|AT2R1A\|AT2R1B\|HAT1R | 3 | Cytokine Receptors |
| AGTR2 | 186 | angiotensin II receptor, type 2 | AT2\|ATGR2\|MRX88 | X | Cytokine Receptors |
| AMHR2 | 269 | anti-Mullerian hormone receptor, type II | AMHR\|MISR2\|MISRII | 12 | Cytokine Receptors |
| ANGPT1 | 284 | angiopoietin 1 | AGP1\|AGPT\|ANG1 | 8 | Cytokine Receptors |
| ANGPT4 | 51378 | angiopoietin 4 | AGP4\|ANG-3\|ANG4\|MGC138181\|MGC138183 | 20 | Cytokine Receptors |
| ANGPTL1 | 9068 | angiopoietin-like 1 | ANG3\|ANGPT3\|ARP1\|AngY\|KIAA0351\|UNQ162\|dJ595C2.2 | 1 | Cytokine Receptors |
| ANGPTL2 | 23452 | angiopoietin-like 2 | ARP2\|HARP\|MGC8889 | 9 | Cytokine Receptors |
| ANGPTL3 | 27329 | angiopoietin-like 3 | ANGPT5 | 1 | Cytokine Receptors |
| ANGPTL4 | 51129 | angiopoietin-like 4 | ANGPTL2\|ARP4\|FIAF\|HFARP\|NL2\|PGAR\|pp1158 | 19 | Cytokine Receptors |
| ANGPTL6 | 83854 | angiopoietin-like 6 | AGF\|ARP5 | 19 | Cytokine Receptors |
| APLNR | 187 | apelin receptor | AGTRL1\|APJ\|APJR\|FLJ90771\|MGC45246 | 11 | Cytokine Receptors |
| AR | 367 | androgen receptor | AIS\|DHTR\|HUMARA\|HYSP1\|KD\|NR3C4\|SBMA\|SMAX1\|TFM | X | Cytokine Receptors |
| AVPR1A | 552 | arginine vasopressin receptor 1A | AVPR1 | 12 | Cytokine Receptors |
| AVPR1B | 553 | arginine vasopressin receptor 1B | AVPR3 | 1 | Cytokine Receptors |
| AVPR2 | 554 | arginine vasopressin receptor 2 | ADHR\|DI1\|DIR\|DIR3\|MGC126533\|MGC138386\|NDI\|V2R | X | Cytokine Receptors |
| BMPR1A | 657 | bone morphogenetic protein receptor, type IA | 10q23del\|ACVRLK3\|ALK3\|CD292\|SKR5 | 10 | Cytokine Receptors |
| BMPR1B | 658 | bone morphogenetic protein receptor, type IB | ALK-6\|ALK6\|CDw293 | 4 | Cytokine Receptors |
| BMPR2 | 659 | bone morphogenetic protein receptor, type II (serine/threonine kinase) | BMPR-II\|BMPR3\|BMR2\|BRK-3\|FLJ41585\|FLJ76945\|PPH1\|T-ALK | 2 | Cytokine Receptors |
| BRD8 | 10902 | bromodomain containing 8 | SMAP\|SMAP2\|p120 | 5 | Cytokine Receptors |
| C3AR1 | 719 | complement component 3a receptor 1 | AZ3B\|C3AR\|HNFAG09 | 12 | Cytokine Receptors |
| C5AR1 | 728 | complement component 5a receptor 1 | C5A\|C5AR\|C5R1\|CD88 | 19 | Cytokine Receptors |
| CALCR | 799 | calcitonin receptor | CRT\|CTR\|CTR1 | 7 | Cytokine Receptors |
| CALCRL | 10203 | calcitonin receptor-like | CGRPR\|CRLR | 2 | Cytokine Receptors |
| CCBP2 | 1238 | chemokine binding protein 2 | CCR10\|CCR9\|CMKBR9\|D6\|MGC126678\|MGC138250\|hD6 | 3 | Cytokine Receptors |
| CCR1 | 1230 | chemokine (C-C motif) receptor 1 | CD191\|CKR-1\|CKR1\|CMKBR1\|HM145\|MIP1aR\|SCYAR1 | 3 | Cytokine Receptors |
| CCR10 | 2826 | chemokine (C-C motif) receptor 10 | GPR2 | 17 | Cytokine Receptors |
| CCR3 | 1232 | chemokine (C-C motif) receptor 3 | CC-CKR-3\|CD193\|CKR3\|CMKBR3\|MGC102841 | 3 | Cytokine Receptors |
| CCR4 | 1233 | chemokine (C-C motif) receptor 4 | CC-CKR-4\|CD194\|CKR4\|CMKBR4\|ChemR13\|HGCN:14099\|K5-5\|MGC88293 | 3 | Cytokine Receptors |
| CCR5 | 1234 | chemokine (C-C motif) receptor 5 | CC-CKR-5\|CCCKR5\|CD195\|CKR-5\|CKR5\|CMKBR5\|FLJ78003\|IDDM22 | 3 | Cytokine Receptors |
| CCR6 | 1235 | chemokine (C-C motif) receptor 6 | BN-1\|CD196\|CKR-L3\|CKR6\|CKRL3\|CMKBR6\|DCR2\|DRY-6\|GPR-CY4\|GPR29\|GPRCY4\|STRL22 | 6 | Cytokine Receptors |
| CCR7 | 1236 | chemokine (C-C motif) receptor 7 | BLR2\|CD197\|CDw197\|CMKBR7\|EBI1 | 17 | Cytokine Receptors |
| CCR8 | 1237 | chemokine (C-C motif) receptor 8 | CDw198\|CKR-L1\|CKRL1\|CMKBR8\|CMKBRL2\|CY6\|GPR-CY6\|MGC129966\|MGC129973\|TER1 | 3 | Cytokine Receptors |
| CCR9 | 10803 | chemokine (C-C motif) receptor 9 | CDw199\|GPR-9-6\|GPR28 | 3 | Cytokine Receptors |
| CCRL1 | 51554 | chemokine (C-C motif) receptor-like 1 | CC-CKR-11\|CCBP2\|CCR10\|CCR11\|CCX-CKR\|CKR-11\|PPR1\|VSHK1 | 3 | Cytokine Receptors |
| CCRL2 | 9034 | chemokine (C-C motif) receptor-like 2 | CKRX\|CRAM-A\|CRAM-B\|FLJ55815\|HCR\|MGC116710\|MGC34104 | 3 | Cytokine Receptors |
| CD40 | 958 | CD40 molecule, TNF receptor superfamily member 5 | Bp50\|CDW40\|MGC9013\|TNFRSF5\|p50 | 20 | Cytokine Receptors |
| CMKLR1 | 1240 | chemokine-like receptor 1 | CHEMERINR\|ChemR23\|DEZ\|MGC126105\|MGC126106 | 12 | Cytokine Receptors |
| CNTFR | 1271 | ciliary neurotrophic factor receptor | MGC1774 | 9 | Cytokine Receptors |
| CRHR1 | 1394 | corticotropin releasing hormone receptor 1 | CRF-R\|CRF1\|CRFR1\|CRH-R1h\|CRHR\|CRHR1f | 17 | Cytokine Receptors |
| CRHR2 | 1395 | corticotropin releasing hormone receptor 2 | CRFR2 | 7 | Cytokine Receptors |
| CRIM1 | 51232 | cysteine rich transmembrane BMP regulator 1 (chordin-like) | MGC138194\|S52 | 2 | Cytokine Receptors |
| CRLF1 | 9244 | cytokine receptor-like factor 1 | CISS\|CISS1\|CLF\|CLF-1\|NR6 | 19 | Cytokine Receptors |
| CRLF2 | 64109 | cytokine receptor-like factor 2 | CRL2\|CRLF2Y\|TSLPR | X\|Y | Cytokine Receptors |
| CRLF3 | 51379 | cytokine receptor-like factor 3 | CREME9\|CYTOR4\|FRWS\|MGC20661 | 17 | Cytokine Receptors |
| CSF1R | 1436 | colony stimulating factor 1 receptor | C-FMS\|CD115\|CSFR\|FIM2\|FMS | 5 | Cytokine Receptors |
| CSF2RA | 1438 | colony stimulating factor 2 receptor, alpha, low-affinity (granulocyte-macrophage) | CD116\|CDw116\|CSF2R\|CSF2RAX\|CSF2RAY\|CSF2RX\|CSF2RY\|GM-CSF-R-alpha\|GMCSFR\|GMR\|MGC3848\|MGC4838 | X\|Y | Cytokine Receptors |
| CSF2RB | 1439 | colony stimulating factor 2 receptor, beta, low-affinity (granulocyte-macrophage) | CD131\|CDw131\|IL3RB\|IL5RB | 22 | Cytokine Receptors |
| CSF3R | 1441 | colony stimulating factor 3 receptor (granulocyte) | CD114\|GCSFR | 1 | Cytokine Receptors |
| CX3CR1 | 1524 | chemokine (C-X3-C motif) receptor 1 | CCRL1\|CMKBRL1\|CMKDR1\|GPR13\|GPRV28\|V28 | 3 | Cytokine Receptors |
| CXCR3 | 2833 | chemokine (C-X-C motif) receptor 3 | CD182\|CD183\|CKR-L2\|CMKAR3\|GPR9\|IP10-R\|Mig-R\|MigR | X | Cytokine Receptors |
| CXCR4 | 7852 | chemokine (C-X-C motif) receptor 4 | CD184\|D2S201E\|FB22\|HM89\|HSY3RR\|LAP3\|LCR1\|LESTR\|NPY3R\|NPYR\|NPYRL\|NPYY3R\|WHIM | 2 | Cytokine Receptors |
| CXCR5 | 643 | chemokine (C-X-C motif) receptor 5 | BLR1\|CD185\|MDR15\|MGC117347 | 11 | Cytokine Receptors |
| CXCR6 | 10663 | chemokine (C-X-C motif) receptor 6 | BONZO\|CD186\|STRL33\|TYMSTR | 3 | Cytokine Receptors |
| CXCR7 | 57007 | chemokine (C-X-C motif) receptor 7 | CMKOR1\|GPR159\|RDC1 | 2 | Cytokine Receptors |
| CYSLTR1 | 10800 | cysteinyl leukotriene receptor 1 | CYSLT1\|CYSLT1R\|CYSLTR\|HG55\|HMTMF81\|MGC46139 | X | Cytokine Receptors |
| CYSLTR2 | 57105 | cysteinyl leukotriene receptor 2 | CYSLT2\|CYSLT2R\|GPCR\|HG57\|HPN321\|KPG_011\|PSEC0146\|hGPCR21 | 13 | Cytokine Receptors |
| DARC | 2532 | Duffy blood group, chemokine receptor | CCBP1\|CD234\|Dfy\|FY\|GPD\|GpFy\|WBCQ1 | 1 | Cytokine Receptors |
| EDNRA | 1909 | endothelin receptor type A | ETA\|ETRA | 4 | Cytokine Receptors |
| EDNRB | 1910 | endothelin receptor type B | ABCDS\|ETB\|ETBR\|ETRB\|HSCR\|HSCR2 | 13 | Cytokine Receptors |
| EGFR | 1956 | epidermal growth factor receptor (erythroblastic leukemia viral (v-erb-b) oncogene homolog, avian) | ERBB\|ERBB1\|HER1\|PIG61\|mENA | 7 | Cytokine Receptors |
| ENG | 2022 | endoglin | CD105\|END\|FLJ41744\|HHT1\|ORW\|ORW1 | 9 | Cytokine Receptors |
| EPOR | 2057 | erythropoietin receptor | MGC138358 | 19 | Cytokine Receptors |
| ESR1 | 2099 | estrogen receptor 1 | DKFZp686N23123\|ER\|ESR\|ESRA\|Era\|NR3A1 | 6 | Cytokine Receptors |
| ESR2 | 2100 | estrogen receptor 2 (ER beta) | ER-BETA\|ESR-BETA\|ESRB\|ESTRB\|Erb\|NR3A2 | 14 | Cytokine Receptors |
| ESRRA | 2101 | estrogen-related receptor alpha | ERR1\|ERRa\|ERRalpha\|ESRL1\|NR3B1 | 11 | Cytokine Receptors |
| ESRRB | 2103 | estrogen-related receptor beta | DFNB35\|ERR2\|ERRb\|ERRbeta\|ERRbeta-2\|ESRL2\|NR3B2 | 14 | Cytokine Receptors |
| ESRRG | 2104 | estrogen-related receptor gamma | DKFZp781L1617\|ERR3\|FLJ16023\|KIAA0832\|NR3B3 | 1 | Cytokine Receptors |
| FGFR1 | 2260 | fibroblast growth factor receptor 1 | BFGFR\|CD331\|CEK\|FGFBR\|FLG\|FLJ99988\|FLT2\|HBGFR\|KAL2\|N-SAM\|OGD | 8 | Cytokine Receptors |
| FGFR2 | 2263 | fibroblast growth factor receptor 2 | BEK\|BFR-1\|CD332\|CEK3\|CFD1\|ECT1\|FLJ98662\|JWS\|K-SAM\|KGFR\|TK14\|TK25 | 10 | Cytokine Receptors |
| FGFR3 | 2261 | fibroblast growth factor receptor 3 | ACH\|CD333\|CEK2\|HSFGFR3EX\|JTK4 | 4 | Cytokine Receptors |
| FGFR4 | 2264 | fibroblast growth factor receptor 4 | CD334\|JTK2\|MGC20292\|TKF | 5 | Cytokine Receptors |
| FGFRL1 | 53834 | fibroblast growth factor receptor-like 1 | FGFR5\|FHFR | 4 | Cytokine Receptors |
| FLT1 | 2321 | fms-related tyrosine kinase 1 (vascular endothelial growth factor/vascular permeability factor receptor) | FLT\|VEGFR1 | 13 | Cytokine Receptors |
| FLT3 | 2322 | fms-related tyrosine kinase 3 | CD135\|FLK2\|STK1 | 13 | Cytokine Receptors |
| FLT4 | 2324 | fms-related tyrosine kinase 4 | FLT41\|LMPH1A\|PCL\|VEGFR3 | 5 | Cytokine Receptors |
| FPR1 | 2357 | formyl peptide receptor 1 | FMLP\|FPR | 19 | Cytokine Receptors |
| FPR2 | 2358 | formyl peptide receptor 2 | ALXR\|FMLP-R-II\|FMLPX\|FPR2A\|FPRH1\|FPRH2\|FPRL1\|HM63\|LXA4R | 19 | Cytokine Receptors |
| FPR2 | 2358 | formyl peptide receptor 2 | ALXR\|FMLP-R-II\|FMLPX\|FPR2A\|FPRH1\|FPRH2\|FPRL1\|HM63\|LXA4R | 19 | Cytokine Receptors |
| FSHR | 2492 | follicle stimulating hormone receptor | FSHRO\|LGR1\|MGC141667\|MGC141668\|ODG1 | 2 | Cytokine Receptors |
| GALR2 | 8811 | galanin receptor 2 | GALNR2\|MGC125983\|MGC125984 | 17 | Cytokine Receptors |
| GALR3 | 8484 | galanin receptor 3 | - | 22 | Cytokine Receptors |
| GCGR | 2642 | glucagon receptor | GGR\|MGC138246 | 17 | Cytokine Receptors |
| GHR | 2690 | growth hormone receptor | GHBP | 5 | Cytokine Receptors |
| GHRHR | 2692 | growth hormone releasing hormone receptor | GHRFR\|GHRHRpsv\|GRFR | 7 | Cytokine Receptors |
| GHSR | 2693 | growth hormone secretagogue receptor | - | 3 | Cytokine Receptors |
| GIPR | 2696 | gastric inhibitory polypeptide receptor | MGC126722 | 19 | Cytokine Receptors |
| GLP1R | 2740 | glucagon-like peptide 1 receptor | MGC138331 | 6 | Cytokine Receptors |
| GLP2R | 9340 | glucagon-like peptide 2 receptor | - | 17 | Cytokine Receptors |
| GNRHR | 2798 | gonadotropin-releasing hormone receptor | GNRHR1\|GRHR\|LHRHR\|LRHR | 4 | Cytokine Receptors |
| GPER | 2852 | G protein-coupled estrogen receptor 1 | CEPR\|CMKRL2\|DRY12\|FEG-1\|GPCR-Br\|GPR30\|LERGU\|LERGU2\|LyGPR\|MGC99678 | 7 | Cytokine Receptors |
| GPR17 | 2840 | G protein-coupled receptor 17 | DKFZp686M18273 | 2 | Cytokine Receptors |
| GPR32 | 2854 | G protein-coupled receptor 32 | - | 19 | Cytokine Receptors |
| GPR33 | 2856 | G protein-coupled receptor 33 (gene/pseudogene) | - | 14 | Cytokine Receptors |
| GPR44 | 11251 | G protein-coupled receptor 44 | CD294\|CRTH2\|DP2 | 11 | Cytokine Receptors |
| GPR77 | 27202 | G protein-coupled receptor 77 | C5L2\|GPF77 | 19 | Cytokine Receptors |
| HNF4A | 3172 | hepatocyte nuclear factor 4, alpha | FLJ39654\|HNF4\|HNF4a7\|HNF4a8\|HNF4a9\|HNF4alpha\|MODY\|MODY1\|NR2A1\|NR2A21\|TCF\|TCF14 | 20 | Cytokine Receptors |
| HNF4G | 3174 | hepatocyte nuclear factor 4, gamma | NR2A2\|NR2A3 | 8 | Cytokine Receptors |
| HTR3A | 3359 | 5-hydroxytryptamine (serotonin) receptor 3A | 5-HT-3\|5-HT3A\|5-HT3R\|5HT3R\|HTR3 | 11 | Cytokine Receptors |
| HTR3B | 9177 | 5-hydroxytryptamine (serotonin) receptor 3B | 5-HT3B | 11 | Cytokine Receptors |
| HTR3C | 170572 | 5-hydroxytryptamine (serotonin) receptor 3, family member C | - | 3 | Cytokine Receptors |
| HTR3D | 200909 | 5-hydroxytryptamine (serotonin) receptor 3 family member D | MGC119636\|MGC119637 | 3 | Cytokine Receptors |
| HTR3E | 285242 | 5-hydroxytryptamine (serotonin) receptor 3, family member E | 5-HT3c1\|MGC120035\|MGC120036\|MGC120037 | 3 | Cytokine Receptors |
| IFNAR1 | 3454 | interferon (alpha, beta and omega) receptor 1 | AVP\|IFN-alpha-REC\|IFNAR\|IFNBR\|IFRC | 21 | Cytokine Receptors |
| IFNAR2 | 3455 | interferon (alpha, beta and omega) receptor 2 | IFN-R\|IFN-alpha-REC\|IFNABR\|IFNARB | 21 | Cytokine Receptors |
| IFNGR1 | 3459 | interferon gamma receptor 1 | CD119\|FLJ45734\|IFNGR | 6 | Cytokine Receptors |
| IFNGR2 | 3460 | interferon gamma receptor 2 (interferon gamma transducer 1) | AF-1\|IFGR2\|IFNGT1 | 21 | Cytokine Receptors |
| IGF1R | 3480 | insulin-like growth factor 1 receptor | CD221\|IGFIR\|IGFR\|JTK13\|MGC142170\|MGC142172\|MGC18216 | 15 | Cytokine Receptors |
| IGF2R | 3482 | insulin-like growth factor 2 receptor | CD222\|CIMPR\|M6P-R\|MPR1\|MPRI | 6 | Cytokine Receptors |
| IL10RA | 3587 | interleukin 10 receptor, alpha | CDW210A\|HIL-10R\|IL-10R1\|IL10R | 11 | Cytokine Receptors |
| IL10RB | 3588 | interleukin 10 receptor, beta | CDW210B\|CRF2-4\|CRFB4\|D21S58\|D21S66\|IL-10R2 | 21 | Cytokine Receptors |
| IL11RA | 3590 | interleukin 11 receptor, alpha | MGC2146 | 9 | Cytokine Receptors |
| IL11RB | 3591 | interleukin 11 receptor, beta | - | - | Cytokine Receptors |
| IL12RB1 | 3594 | interleukin 12 receptor, beta 1 | CD212\|IL-12R-BETA1\|IL12RB\|MGC34454 | 19 | Cytokine Receptors |
| IL12RB2 | 3595 | interleukin 12 receptor, beta 2 | - | 1 | Cytokine Receptors |
| IL13RA1 | 3597 | interleukin 13 receptor, alpha 1 | CD213A1\|IL-13Ra\|NR4 | X | Cytokine Receptors |
| IL13RA2 | 3598 | interleukin 13 receptor, alpha 2 | CD213A2\|CT19\|IL-13R\|IL13BP | X | Cytokine Receptors |
| IL15RA | 3601 | interleukin 15 receptor, alpha | MGC104179 | 10 | Cytokine Receptors |
| IL15RB | 3602 | interleukin 15 receptor, beta | - | - | Cytokine Receptors |
| IL17RA | 23765 | interleukin 17 receptor A | CD217\|CDw217\|IL-17RA\|IL17R\|MGC10262\|hIL-17R | 22 | Cytokine Receptors |
| IL17RB | 55540 | interleukin 17 receptor B | CRL4\|EVI27\|IL17BR\|IL17RH1\|MGC5245 | 3 | Cytokine Receptors |
| IL17RC | 84818 | interleukin 17 receptor C | FLJ95963\|FLJ96005\|IL17-RL\|IL17RL\|MGC10763 | 3 | Cytokine Receptors |
| IL17RD | 54756 | interleukin 17 receptor D | DKFZp434N1928\|FLJ35755\|IL-17RD\|IL17RLM\|MGC133309\|SEF | 3 | Cytokine Receptors |
| IL17RE | 132014 | interleukin 17 receptor E | FLJ23658\|MGC71884 | 3 | Cytokine Receptors |
| IL18R1 | 8809 | interleukin 18 receptor 1 | CD218a\|CDw218a\|IL-1Rrp\|IL18RA\|IL1RRP | 2 | Cytokine Receptors |
| IL18RAP | 8807 | interleukin 18 receptor accessory protein | ACPL\|CD218b\|CDw218b\|IL18RB\|MGC120589\|MGC120590 | 2 | Cytokine Receptors |
| IL1R1 | 3554 | interleukin 1 receptor, type I | CD121A\|D2S1473\|IL-1R-alpha\|IL1R\|IL1RA\|P80 | 2 | Cytokine Receptors |
| IL1R2 | 7850 | interleukin 1 receptor, type II | CD121b\|IL1RB\|MGC47725 | 2 | Cytokine Receptors |
| IL1RAP | 3556 | interleukin 1 receptor accessory protein | C3orf13\|FLJ37788\|IL-1RAcP\|IL1R3 | 3 | Cytokine Receptors |
| IL1RL1 | 9173 | interleukin 1 receptor-like 1 | DER4\|FIT-1\|MGC32623\|ST2\|ST2L\|ST2V\|T1 | 2 | Cytokine Receptors |
| IL1RL2 | 8808 | interleukin 1 receptor-like 2 | IL1R-rp2\|IL1RRP2 | 2 | Cytokine Receptors |
| IL20RA | 53832 | interleukin 20 receptor, alpha | FLJ40993\|IL-20R1\|ZCYTOR7 | 6 | Cytokine Receptors |
| IL20RB | 53833 | interleukin 20 receptor beta | DIRS1\|FNDC6\|IL-20R2\|MGC34923 | 3 | Cytokine Receptors |
| IL21R | 50615 | interleukin 21 receptor | MGC10967\|NILR | 16 | Cytokine Receptors |
| IL22RA1 | 58985 | interleukin 22 receptor, alpha 1 | CRF2-9\|IL22R\|IL22R1 | 1 | Cytokine Receptors |
| IL22RA2 | 116379 | interleukin 22 receptor, alpha 2 | CRF2-10\|CRF2-S1\|CRF2X\|IL-22BP\|MGC150509\|MGC150510 | 6 | Cytokine Receptors |
| IL23R | 149233 | interleukin 23 receptor | - | 1 | Cytokine Receptors |
| IL27RA | 9466 | interleukin 27 receptor, alpha | CRL1\|IL27R\|TCCR\|WSX1\|zcytor1 | 19 | Cytokine Receptors |
| IL28RA | 163702 | interleukin 28 receptor, alpha (interferon, lambda receptor) | CRF2/12\|IFNLR\|IFNLR1\|IL-28R1\|LICR2 | 1 | Cytokine Receptors |
| IL2RA | 3559 | interleukin 2 receptor, alpha | CD25\|IDDM10\|IL2R\|TCGFR | 10 | Cytokine Receptors |
| IL2RB | 3560 | interleukin 2 receptor, beta | CD122\|P70-75 | 22 | Cytokine Receptors |
| IL2RG | 3561 | interleukin 2 receptor, gamma (severe combined immunodeficiency) | CD132\|IMD4\|SCIDX\|SCIDX1 | X | Cytokine Receptors |
| IL31RA | 133396 | interleukin 31 receptor A | CRL\|CRL3\|GLM-R\|GLMR\|GPL\|IL-31RA\|MGC125346\|PRO21384 | 5 | Cytokine Receptors |
| IL3RA | 3563 | interleukin 3 receptor, alpha (low affinity) | CD123\|IL3R\|IL3RAY\|IL3RX\|IL3RY\|MGC34174\|hIL-3Ra | X\|Y | Cytokine Receptors |
| IL4R | 3566 | interleukin 4 receptor | CD124\|IL4RA | 16 | Cytokine Receptors |
| IL5RA | 3568 | interleukin 5 receptor, alpha | CD125\|CDw125\|HSIL5R3\|IL5R\|MGC26560 | 3 | Cytokine Receptors |
| IL6R | 3570 | interleukin 6 receptor | CD126\|IL-6R-1\|IL-6R-alpha\|IL6RA\|MGC104991 | 1 | Cytokine Receptors |
| IL7R | 3575 | interleukin 7 receptor | CD127\|CDW127\|IL-7R-alpha\|IL7RA\|ILRA | 5 | Cytokine Receptors |
| IL8RA | 3577 | interleukin 8 receptor, alpha | C-C\|C-C-CKR-1\|CD128\|CD181\|CDw128a\|CKR-1\|CMKAR1\|CXCR1\|IL8R1\|IL8RBA | 2 | Cytokine Receptors |
| IL8RB | 3579 | interleukin 8 receptor, beta | CD182\|CDw128b\|CMKAR2\|CXCR2\|IL8R2\|IL8RA | 2 | Cytokine Receptors |
| IL9R | 3581 | interleukin 9 receptor | CD129 | X\|Y | Cytokine Receptors |
| INSR | 3643 | insulin receptor | CD220\|HHF5 | 19 | Cytokine Receptors |
| KDR | 3791 | kinase insert domain receptor (a type III receptor tyrosine kinase) | CD309\|FLK1\|VEGFR\|VEGFR2 | 4 | Cytokine Receptors |
| LEPR | 3953 | leptin receptor | CD295\|OBR | 1 | Cytokine Receptors |
| LGR4 | 55366 | leucine-rich repeat-containing G protein-coupled receptor 4 | GPR48 | 11 | Cytokine Receptors |
| LGR5 | 8549 | leucine-rich repeat-containing G protein-coupled receptor 5 | FEX\|GPR49\|GPR67\|GRP49\|HG38\|MGC117008 | 12 | Cytokine Receptors |
| LGR6 | 59352 | leucine-rich repeat-containing G protein-coupled receptor 6 | FLJ14471\|GPCR\|VTS20631 | 1 | Cytokine Receptors |
| LHCGR | 3973 | luteinizing hormone/choriogonadotropin receptor | FLJ41504\|LCGR\|LGR2\|LH/CG-R\|LH/CGR\|LHR\|LHRHR\|LSH-R | 2 | Cytokine Receptors |
| LIFR | 3977 | leukemia inhibitory factor receptor alpha | CD118\|FLJ98106\|FLJ99923\|LIF-R\|SJS2\|STWS\|SWS | 5 | Cytokine Receptors |
| LTB4R | 1241 | leukotriene B4 receptor | BLT1\|BLTR\|CMKRL1\|GPR16\|LTB4R1\|LTBR1\|P2RY7\|P2Y7 | 14 | Cytokine Receptors |
| LTB4R2 | 56413 | leukotriene B4 receptor 2 | BLT2\|BLTR2\|JULF2\|KPG_004\|NOP9 | 14 | Cytokine Receptors |
| LTBR | 4055 | lymphotoxin beta receptor (TNFR superfamily, member 3) | CD18\|D12S370\|LT-BETA-R\|TNF-R-III\|TNFCR\|TNFR-RP\|TNFR2-RP\|TNFRSF3 | 12 | Cytokine Receptors |
| MC1R | 4157 | melanocortin 1 receptor (alpha melanocyte stimulating hormone receptor) | MGC14337\|MSH-R\|SHEP2 | 16 | Cytokine Receptors |
| MC2R | 4158 | melanocortin 2 receptor (adrenocorticotropic hormone) | ACTHR\|MGC125798 | 18 | Cytokine Receptors |
| MC3R | 4159 | melanocortin 3 receptor | BMIQ9\|MC3\|MC3-R\|OB20\|OQTL | 20 | Cytokine Receptors |
| MC4R | 4160 | melanocortin 4 receptor | MGC126851\|MGC138197 | 18 | Cytokine Receptors |
| MCHR1 | 2847 | melanin-concentrating hormone receptor 1 | GPR24\|MCH1R\|MGC32129\|SLC1 | 22 | Cytokine Receptors |
| MCHR2 | 84539 | melanin-concentrating hormone receptor 2 | GPR145\|MCH2\|MCH2R\|SLT | 6 | Cytokine Receptors |
| MET | 4233 | met proto-oncogene (hepatocyte growth factor receptor) | AUTS9\|HGFR\|RCCP2\|c-Met | 7 | Cytokine Receptors |
| MLNR | 2862 | motilin receptor | GPR38\|MTLR1 | 13 | Cytokine Receptors |
| MPL | 4352 | myeloproliferative leukemia virus oncogene | C-MPL\|CD110\|MPLV\|TPOR | 1 | Cytokine Receptors |
| MTNR1A | 4543 | melatonin receptor 1A | MEL-1A-R\|MT1 | 4 | Cytokine Receptors |
| MTNR1B | 4544 | melatonin receptor 1B | MEL-1B-R\|MT2 | 11 | Cytokine Receptors |
| NGFR | 4804 | nerve growth factor receptor (TNFR superfamily, member 16) | CD271\|Gp80-LNGFR\|TNFRSF16\|p75(NTR)\|p75NTR | 17 | Cytokine Receptors |
| NMBR | 4829 | neuromedin B receptor | - | 6 | Cytokine Receptors |
| NPR1 | 4881 | natriuretic peptide receptor A/guanylate cyclase A (atrionatriuretic peptide receptor A) | ANPRA\|ANPa\|GUC2A\|GUCY2A\|NPRA | 1 | Cytokine Receptors |
| NPR3 | 4883 | natriuretic peptide receptor C/guanylate cyclase C (atrionatriuretic peptide receptor C) | ANPRC\|GUCY2B\|NPRC | 5 | Cytokine Receptors |
| NR0B1 | 190 | nuclear receptor subfamily 0, group B, member 1 | AHC\|AHCH\|AHX\|DAX-1\|DAX1\|DSS\|GTD\|HHG\|NROB1 | X | Cytokine Receptors |
| NR0B2 | 8431 | nuclear receptor subfamily 0, group B, member 2 | FLJ17090\|SHP\|SHP1 | 1 | Cytokine Receptors |
| NR1D1 | 9572 | nuclear receptor subfamily 1, group D, member 1 | EAR1\|THRA1\|THRAL\|ear-1\|hRev | 17 | Cytokine Receptors |
| NR1D2 | 9975 | nuclear receptor subfamily 1, group D, member 2 | BD73\|EAR-1R\|RVR | 3 | Cytokine Receptors |
| NR1H2 | 7376 | nuclear receptor subfamily 1, group H, member 2 | LXR-b\|LXRB\|NER\|NER-I\|RIP15\|UNR | 19 | Cytokine Receptors |
| NR1H3 | 10062 | nuclear receptor subfamily 1, group H, member 3 | LXR-a\|LXRA\|RLD-1 | 11 | Cytokine Receptors |
| NR1H4 | 9971 | nuclear receptor subfamily 1, group H, member 4 | BAR\|FXR\|HRR-1\|HRR1\|MGC163445\|RIP14 | 12 | Cytokine Receptors |
| NR1I2 | 8856 | nuclear receptor subfamily 1, group I, member 2 | BXR\|ONR1\|PAR\|PAR1\|PAR2\|PARq\|PRR\|PXR\|SAR\|SXR | 3 | Cytokine Receptors |
| NR1I3 | 9970 | nuclear receptor subfamily 1, group I, member 3 | CAR\|CAR1\|MB67\|MGC150433\|MGC97144\|MGC97209 | 1 | Cytokine Receptors |
| NR2C1 | 7181 | nuclear receptor subfamily 2, group C, member 1 | TR2 | 12 | Cytokine Receptors |
| NR2C2 | 7182 | nuclear receptor subfamily 2, group C, member 2 | TAK1\|TR2R1\|TR4\|hTAK1 | 3 | Cytokine Receptors |
| NR2E1 | 7101 | nuclear receptor subfamily 2, group E, member 1 | TLL\|TLX\|XTLL | 6 | Cytokine Receptors |
| NR2E3 | 10002 | nuclear receptor subfamily 2, group E, member 3 | ESCS\|MGC49976\|PNR\|RNR\|RP37\|rd7 | 15 | Cytokine Receptors |
| NR2F1 | 7025 | nuclear receptor subfamily 2, group F, member 1 | COUP-TFI\|EAR-3\|EAR3\|ERBAL3\|NR2F2\|SVP44\|TCFCOUP1\|TFCOUP1 | 5 | Cytokine Receptors |
| NR2F2 | 7026 | nuclear receptor subfamily 2, group F, member 2 | ARP1\|COUP-TFII\|COUPTFB\|MGC117452\|SVP40\|TFCOUP2 | 15 | Cytokine Receptors |
| NR2F6 | 2063 | nuclear receptor subfamily 2, group F, member 6 | EAR-2\|EAR2\|ERBAL2 | 19 | Cytokine Receptors |
| NR3C1 | 2908 | nuclear receptor subfamily 3, group C, member 1 (glucocorticoid receptor) | GCCR\|GCR\|GR\|GRL | 5 | Cytokine Receptors |
| NR3C2 | 4306 | nuclear receptor subfamily 3, group C, member 2 | MCR\|MGC133092\|MLR\|MR | 4 | Cytokine Receptors |
| NR4A1 | 3164 | nuclear receptor subfamily 4, group A, member 1 | GFRP1\|HMR\|MGC9485\|N10\|NAK-1\|NGFIB\|NP10\|NUR77\|TR3 | 12 | Cytokine Receptors |
| NR4A2 | 4929 | nuclear receptor subfamily 4, group A, member 2 | HZF-3\|NOT\|NURR1\|RNR1\|TINUR | 2 | Cytokine Receptors |
| NR4A3 | 8013 | nuclear receptor subfamily 4, group A, member 3 | CHN\|CSMF\|MINOR\|NOR1\|TEC | 9 | Cytokine Receptors |
| NR5A1 | 2516 | nuclear receptor subfamily 5, group A, member 1 | AD4BP\|ELP\|FTZ1\|FTZF1\|SF-1\|SF1 | 9 | Cytokine Receptors |
| NR5A2 | 2494 | nuclear receptor subfamily 5, group A, member 2 | B1F\|B1F2\|CPF\|FTF\|FTZ-F1\|FTZ-F1beta\|LRH-1\|hB1F\|hB1F-2 | 1 | Cytokine Receptors |
| NR6A1 | 2649 | nuclear receptor subfamily 6, group A, member 1 | GCNF\|GCNF1\|NR61\|RTR | 9 | Cytokine Receptors |
| NRP1 | 8829 | neuropilin 1 | BDCA4\|CD304\|DKFZp686A03134\|DKFZp781F1414\|NP1\|NRP\|VEGF165R | 10 | Cytokine Receptors |
| NRP2 | 8828 | neuropilin 2 | MGC126574\|NP2\|NPN2\|PRO2714\|VEGF165R2 | 2 | Cytokine Receptors |
| OGFR | 11054 | opioid growth factor receptor | - | 20 | Cytokine Receptors |
| OPRD1 | 4985 | opioid receptor, delta 1 | OPRD | 1 | Cytokine Receptors |
| OPRK1 | 4986 | opioid receptor, kappa 1 | KOR\|OPRK | 8 | Cytokine Receptors |
| OPRL1 | 4987 | opiate receptor-like 1 | KOR-3\|MGC34578\|NOCIR\|OOR\|ORL1 | 20 | Cytokine Receptors |
| OPRM1 | 4988 | opioid receptor, mu 1 | KIAA0403\|LMOR\|MOR\|MOR1\|OPRM | 6 | Cytokine Receptors |
| OSMR | 9180 | oncostatin M receptor | MGC150626\|MGC150627\|MGC75127\|OSMRB | 5 | Cytokine Receptors |
| OXTR | 5021 | oxytocin receptor | OT-R | 3 | Cytokine Receptors |
| PGR | 5241 | progesterone receptor | NR3C3\|PR | 11 | Cytokine Receptors |
| PGRMC2 | 10424 | progesterone receptor membrane component 2 | DG6\|PMBP | 4 | Cytokine Receptors |
| PLAUR | 5329 | plasminogen activator, urokinase receptor | CD87\|UPAR\|URKR | 19 | Cytokine Receptors |
| PLXNA1 | 5361 | plexin A1 | NOV\|NOVP\|PLEXIN-A1\|PLXN1 | 3 | Cytokine Receptors |
| PLXNA2 | 5362 | plexin A2 | FLJ11751\|FLJ30634\|KIAA0463\|OCT\|PLXN2 | 1 | Cytokine Receptors |
| PLXNA3 | 55558 | plexin A3 | 6.3\|HSSEXGENE\|PLEXIN-A3\|PLXN3\|PLXN4\|SEX\|XAP-6 | X | Cytokine Receptors |
| PLXNA4 | 91584 | plexin A4 | DKFZp434G0625\|DKFZp566O0546\|FAYV2820\|FLJ35026\|FLJ38287\|KIAA1550\|PLEXA4\|PLXNA4A\|PLXNA4B\|PRO34003 | 7 | Cytokine Receptors |
| PLXNB1 | 5364 | plexin B1 | KIAA0407\|MGC149167\|PLEXIN-B1\|PLXN5\|SEP | 3 | Cytokine Receptors |
| PLXNB2 | 23654 | plexin B2 | KIAA0315\|MM1\|Nbla00445\|PLEXB2\|dJ402G11.3 | 22 | Cytokine Receptors |
| PLXNB3 | 5365 | plexin B3 | FLJ76953\|PLEXB3\|PLEXR\|PLXN6 | X | Cytokine Receptors |
| PLXNC1 | 10154 | plexin C1 | CD232\|PLXN-C1\|VESPR | 12 | Cytokine Receptors |
| PLXND1 | 23129 | plexin D1 | KIAA0620\|MGC75353\|PLEXD1 | 3 | Cytokine Receptors |
| PPARA | 5465 | peroxisome proliferator-activated receptor alpha | MGC2237\|MGC2452\|NR1C1\|PPAR\|hPPAR | 22 | Cytokine Receptors |
| PPARD | 5467 | peroxisome proliferator-activated receptor delta | FAAR\|MGC3931\|NR1C2\|NUC1\|NUCI\|NUCII\|PPAR-beta\|PPARB | 6 | Cytokine Receptors |
| PPARG | 5468 | peroxisome proliferator-activated receptor gamma | CIMT1\|NR1C3\|PPARG1\|PPARG2\|PPARgamma | 3 | Cytokine Receptors |
| PRLHR | 2834 | prolactin releasing hormone receptor | GPR10\|GR3\|MGC126539\|MGC126541\|PrRPR | 10 | Cytokine Receptors |
| PRLR | 5618 | prolactin receptor | hPRLrI | 5 | Cytokine Receptors |
| PTAFR | 5724 | platelet-activating factor receptor | PAFR | 1 | Cytokine Receptors |
| PTGDR | 5729 | prostaglandin D2 receptor (DP) | AS1\|ASRT1\|DP\|DP1\|MGC49004 | 14 | Cytokine Receptors |
| PTGDS | 5730 | prostaglandin D2 synthase 21kDa (brain) | LPGDS\|PDS\|PGD2\|PGDS\|PGDS2 | 9 | Cytokine Receptors |
| PTGER1 | 5731 | prostaglandin E receptor 1 (subtype EP1), 42kDa | EP1 | 19 | Cytokine Receptors |
| PTGER2 | 5732 | prostaglandin E receptor 2 (subtype EP2), 53kDa | EP2 | 14 | Cytokine Receptors |
| PTGER3 | 5733 | prostaglandin E receptor 3 (subtype EP3) | EP3\|EP3-I\|EP3-II\|EP3-III\|EP3-IV\|EP3e\|MGC141828\|MGC141829\|MGC27302 | 1 | Cytokine Receptors |
| PTGER4 | 5734 | prostaglandin E receptor 4 (subtype EP4) | EP4\|EP4R\|MGC126583 | 5 | Cytokine Receptors |
| PTGFR | 5737 | prostaglandin F receptor (FP) | FP\|MGC120498\|MGC46203 | 1 | Cytokine Receptors |
| PTH1R | 5745 | parathyroid hormone 1 receptor | MGC138426\|MGC138452\|PFE\|PTHR\|PTHR1 | 3 | Cytokine Receptors |
| PTH2R | 5746 | parathyroid hormone 2 receptor | PTHR2 | 2 | Cytokine Receptors |
| RARA | 5914 | retinoic acid receptor, alpha | NR1B1\|RAR | 17 | Cytokine Receptors |
| RARB | 5915 | retinoic acid receptor, beta | HAP\|NR1B2\|RRB2 | 3 | Cytokine Receptors |
| RARG | 5916 | retinoic acid receptor, gamma | NR1B3\|RARC | 12 | Cytokine Receptors |
| ROBO1 | 6091 | roundabout, axon guidance receptor, homolog 1 (Drosophila) | DUTT1\|FLJ21882\|MGC131599\|MGC133277\|SAX3 | 3 | Cytokine Receptors |
| ROBO2 | 6092 | roundabout, axon guidance receptor, homolog 2 (Drosophila) | KIAA1568\|SAX3 | 3 | Cytokine Receptors |
| ROBO3 | 64221 | roundabout, axon guidance receptor, homolog 3 (Drosophila) | FLJ21044\|HGPPS\|HGPS\|RBIG1\|RIG1 | 11 | Cytokine Receptors |
| RORA | 6095 | RAR-related orphan receptor A | DKFZp686M2414\|MGC119326\|MGC119329\|NR1F1\|ROR1\|ROR2\|ROR3\|RZR-ALPHA\|RZRA | 15 | Cytokine Receptors |
| RORB | 6096 | RAR-related orphan receptor B | NR1F2\|ROR-BETA\|RZR-BETA\|RZRB\|bA133M9.1 | 9 | Cytokine Receptors |
| RORC | 6097 | RAR-related orphan receptor C | MGC129539\|NR1F3\|RORG\|RZR-GAMMA\|RZRG\|TOR | 1 | Cytokine Receptors |
| RXFP1 | 59350 | relaxin/insulin-like family peptide receptor 1 | LGR7\|LGR7.1\|LGR7.10\|LGR7.2\|MGC138347\|MGC142177\|RXFPR1 | 4 | Cytokine Receptors |
| RXFP2 | 122042 | relaxin/insulin-like family peptide receptor 2 | GPR106\|GREAT\|INSL3R\|LGR8\|LGR8.1\|RXFPR2 | 13 | Cytokine Receptors |
| RXFP3 | 51289 | relaxin/insulin-like family peptide receptor 3 | GPCR135\|MGC141998\|MGC142000\|RLN3R1\|RXFPR3\|SALPR | 5 | Cytokine Receptors |
| RXRA | 6256 | retinoid X receptor, alpha | FLJ00280\|FLJ00318\|FLJ16020\|FLJ16733\|MGC102720\|NR2B1 | 9 | Cytokine Receptors |
| RXRB | 6257 | retinoid X receptor, beta | DAUDI6\|H-2RIIBP\|MGC1831\|NR2B2\|RCoR-1 | 6 | Cytokine Receptors |
| RXRG | 6258 | retinoid X receptor, gamma | NR2B3\|RXRC | 1 | Cytokine Receptors |
| S1PR1 | 1901 | sphingosine-1-phosphate receptor 1 | CHEDG1\|D1S3362\|ECGF1\|EDG-1\|EDG1\|FLJ58121\|S1P1 | 1 | Cytokine Receptors |
| S1PR2 | 9294 | sphingosine-1-phosphate receptor 2 | AGR16\|EDG-5\|EDG5\|Gpcr13\|H218\|LPB2\|S1P2 | 19 | Cytokine Receptors |
| SCTR | 6344 | secretin receptor | SR | 2 | Cytokine Receptors |
| SDC1 | 6382 | syndecan 1 | CD138\|SDC\|SYND1\|syndecan | 2 | Cytokine Receptors |
| SDC2 | 6383 | syndecan 2 | HSPG\|HSPG1\|SYND2 | 8 | Cytokine Receptors |
| SDC3 | 9672 | syndecan 3 | N-syndecan\|SDCN\|SYND3 | 1 | Cytokine Receptors |
| SDC4 | 6385 | syndecan 4 | MGC22217\|SYND4 | 20 | Cytokine Receptors |
| SORT1 | 6272 | sortilin 1 | Gp95\|NT3 | 1 | Cytokine Receptors |
| SSTR1 | 6751 | somatostatin receptor 1 | SRIF-2 | 14 | Cytokine Receptors |
| SSTR2 | 6752 | somatostatin receptor 2 | - | 17 | Cytokine Receptors |
| SSTR5 | 6755 | somatostatin receptor 5 | - | 16 | Cytokine Receptors |
| ST2 | 6761 | suppression of tumorigenicity 2 | - | 11 | Cytokine Receptors |
| TACR1 | 6869 | tachykinin receptor 1 | NK1R\|NKIR\|SPR\|TAC1R | 2 | Cytokine Receptors |
| TEK | 7010 | TEK tyrosine kinase, endothelial | CD202B\|TIE-2\|TIE2\|VMCM\|VMCM1 | 9 | Cytokine Receptors |
| TGFBR1 | 7046 | transforming growth factor, beta receptor 1 | AAT5\|ACVRLK4\|ALK-5\|ALK5\|LDS1A\|LDS2A\|SKR4\|TGFR-1 | 9 | Cytokine Receptors |
| TGFBR2 | 7048 | transforming growth factor, beta receptor II (70/80kDa) | AAT3\|FAA3\|LDS1B\|LDS2B\|MFS2\|RIIC\|TAAD2\|TGFR-2\|TGFbeta-RII | 3 | Cytokine Receptors |
| TGFBR3 | 7049 | transforming growth factor, beta receptor III | BGCAN\|betaglycan | 1 | Cytokine Receptors |
| THRA | 7067 | thyroid hormone receptor, alpha (erythroblastic leukemia viral (v-erb-a) oncogene homolog, avian) | AR7\|EAR7\|ERB-T-1\|ERBA\|ERBA1\|MGC000261\|MGC43240\|NR1A1\|THRA1\|THRA2\|c-ERBA-1 | 17 | Cytokine Receptors |
| THRB | 7068 | thyroid hormone receptor, beta (erythroblastic leukemia viral (v-erb-a) oncogene homolog 2, avian) | ERBA-BETA\|ERBA2\|GRTH\|MGC126109\|MGC126110\|NR1A2\|PRTH\|THR1\|THRB1\|THRB2 | 3 | Cytokine Receptors |
| TIE1 | 7075 | tyrosine kinase with immunoglobulin-like and EGF-like domains 1 | JTK14\|TIE | 1 | Cytokine Receptors |
| TNFRSF10A | 8797 | tumor necrosis factor receptor superfamily, member 10a | APO2\|CD261\|DR4\|MGC9365\|TRAILR-1\|TRAILR1 | 8 | Cytokine Receptors |
| TNFRSF10B | 8795 | tumor necrosis factor receptor superfamily, member 10b | CD262\|DR5\|KILLER\|KILLER/DR5\|TRAIL-R2\|TRAILR2\|TRICK2\|TRICK2A\|TRICK2B\|TRICKB\|ZTNFR9 | 8 | Cytokine Receptors |
| TNFRSF10C | 8794 | tumor necrosis factor receptor superfamily, member 10c, decoy without an intracellular domain | CD263\|DCR1\|LIT\|MGC149501\|MGC149502\|TRAILR3\|TRID | 8 | Cytokine Receptors |
| TNFRSF10D | 8793 | tumor necrosis factor receptor superfamily, member 10d, decoy with truncated death domain | CD264\|DCR2\|TRAILR4\|TRUNDD | 8 | Cytokine Receptors |
| TNFRSF11A | 8792 | tumor necrosis factor receptor superfamily, member 11a, NFKB activator | CD265\|FEO\|LOH18CR1\|ODFR\|OFE\|OPTB7\|OSTS\|PDB2\|RANK\|TRANCER | 18 | Cytokine Receptors |
| TNFRSF12A | 51330 | tumor necrosis factor receptor superfamily, member 12A | CD266\|FN14\|TWEAKR | 16 | Cytokine Receptors |
| TNFRSF13B | 23495 | tumor necrosis factor receptor superfamily, member 13B | CD267\|CVID\|FLJ39942\|MGC133214\|MGC39952\|TACI\|TNFRSF14B | 17 | Cytokine Receptors |
| TNFRSF13C | 115650 | tumor necrosis factor receptor superfamily, member 13C | BAFF-R\|BAFFR\|CD268\|MGC138235 | 22 | Cytokine Receptors |
| TNFRSF14 | 8764 | tumor necrosis factor receptor superfamily, member 14 (herpesvirus entry mediator) | ATAR\|HVEA\|HVEM\|LIGHTR\|TR2 | 1 | Cytokine Receptors |
| TNFRSF17 | 608 | tumor necrosis factor receptor superfamily, member 17 | BCM\|BCMA\|CD269 | 16 | Cytokine Receptors |
| TNFRSF18 | 8784 | tumor necrosis factor receptor superfamily, member 18 | AITR\|GITR\|GITR-D | 1 | Cytokine Receptors |
| TNFRSF19 | 55504 | tumor necrosis factor receptor superfamily, member 19 | TAJ\|TAJ-alpha\|TRADE\|TROY | 13 | Cytokine Receptors |
| TNFRSF1A | 7132 | tumor necrosis factor receptor superfamily, member 1A | CD120a\|FPF\|MGC19588\|TBP1\|TNF-R\|TNF-R-I\|TNF-R55\|TNFAR\|TNFR1\|TNFR55\|TNFR60\|p55\|p55-R\|p60 | 12 | Cytokine Receptors |
| TNFRSF1B | 7133 | tumor necrosis factor receptor superfamily, member 1B | CD120b\|TBPII\|TNF-R-II\|TNF-R75\|TNFBR\|TNFR1B\|TNFR2\|TNFR80\|p75\|p75TNFR | 1 | Cytokine Receptors |
| TNFRSF21 | 27242 | tumor necrosis factor receptor superfamily, member 21 | BM-018\|DR6\|MGC31965 | 6 | Cytokine Receptors |
| TNFRSF25 | 8718 | tumor necrosis factor receptor superfamily, member 25 | APO-3\|DDR3\|DR3\|LARD\|TNFRSF12\|TR3\|TRAMP\|WSL-1\|WSL-LR | 1 | Cytokine Receptors |
| TNFRSF4 | 7293 | tumor necrosis factor receptor superfamily, member 4 | ACT35\|CD134\|OX40\|TXGP1L | 1 | Cytokine Receptors |
| TNFRSF6B | 8771 | tumor necrosis factor receptor superfamily, member 6b, decoy | DCR3\|DJ583P15.1.1\|M68\|TR6 | 20 | Cytokine Receptors |
| TNFRSF8 | 943 | tumor necrosis factor receptor superfamily, member 8 | CD30\|D1S166E\|Ki-1 | 1 | Cytokine Receptors |
| TNFRSF9 | 3604 | tumor necrosis factor receptor superfamily, member 9 | 4-1BB\|CD137\|CDw137\|ILA\|MGC2172 | 1 | Cytokine Receptors |
| TRHR | 7201 | thyrotropin-releasing hormone receptor | MGC141920 | 8 | Cytokine Receptors |
| TSHR | 7253 | thyroid stimulating hormone receptor | CHNG1\|LGR3\|MGC75129\|hTSHR-I | 14 | Cytokine Receptors |
| TUBB3 | 10381 | tubulin, beta 3 | MC1R\|TUBB4\|beta-4 | 16 | Cytokine Receptors |
| VDR | 7421 | vitamin D (1,25- dihydroxyvitamin D3) receptor | NR1I1 | 12 | Cytokine Receptors |
| VIPR1 | 7433 | vasoactive intestinal peptide receptor 1 | FLJ41949\|HVR1\|II\|PACAP-R-2\|RDC1\|VAPC1\|VIPR\|VIRG\|VPAC1\|VPCAP1R | 3 | Cytokine Receptors |
| VIPR2 | 7434 | vasoactive intestinal peptide receptor 2 | FLJ16511\|VPAC2\|VPCAP2R | 7 | Cytokine Receptors |
| XCR1 | 2829 | chemokine (C motif) receptor 1 | CCXCR1\|GPR5 | 3 | Cytokine Receptors |
| IFNA10 | 3446 | interferon, alpha 10 | MGC119878\|MGC119879 | 9 | Interferons |
| IFNA13 | 3447 | interferon, alpha 13 | - | 9 | Interferons |
| IFNA14 | 3448 | interferon, alpha 14 | LEIF2H\|MGC125756\|MGC125757 | 9 | Interferons |
| IFNA16 | 3449 | interferon, alpha 16 | - | 9 | Interferons |
| IFNA17 | 3451 | interferon, alpha 17 | IFNA\|INFA\|LEIF2C1 | 9 | Interferons |
| IFNA2 | 3440 | interferon, alpha 2 | IFNA\|INFA2\|MGC125764\|MGC125765 | 9 | Interferons |
| IFNA21 | 3452 | interferon, alpha 21 | MGC126687\|MGC126689 | 9 | Interferons |
| IFNA4 | 3441 | interferon, alpha 4 | INFA4\|MGC142200 | 9 | Interferons |
| IFNA5 | 3442 | interferon, alpha 5 | INFA5 | 9 | Interferons |
| IFNA6 | 3443 | interferon, alpha 6 | - | 9 | Interferons |
| IFNA7 | 3444 | interferon, alpha 7 | IFNA-J | 9 | Interferons |
| IFNA8 | 3445 | interferon, alpha 8 | - | 9 | Interferons |
| IFNB1 | 3456 | interferon, beta 1, fibroblast | IFB\|IFF\|IFNB\|MGC96956 | 9 | Interferons |
| IFNE | 338376 | interferon, epsilon | IFN-E\|IFNE1\|IFNT1\|MGC119018\|MGC119020\|PRO655 | 9 | Interferons |
| IFNG | 3458 | interferon, gamma | IFG\|IFI | 12 | Interferons |
| IFNK | 56832 | interferon, kappa | RP11-27J8.1 | 9 | Interferons |
| IFNW1 | 3467 | interferon, omega 1 | - | 9 | Interferons |
| IFNAR2 | 3455 | interferon (alpha, beta and omega) receptor 2 | IFN-R\|IFN-alpha-REC\|IFNABR\|IFNARB | 21 | Interferon Receptor |
| IFNGR1 | 3459 | interferon gamma receptor 1 | CD119\|FLJ45734\|IFNGR | 6 | Interferon Receptor |
| IFNGR2 | 3460 | interferon gamma receptor 2 (interferon gamma transducer 1) | AF-1\|IFGR2\|IFNGT1 | 21 | Interferon Receptor |
| IL11 | 3589 | interleukin 11 | AGIF\|IL-11 | 19 | Interleukins |
| IL12A | 3592 | interleukin 12A (natural killer cell stimulatory factor 1, cytotoxic lymphocyte maturation factor 1, p35) | CLMF\|IL-12A\|NFSK\|NKSF1\|P35 | 3 | Interleukins |
| IL12B | 3593 | interleukin 12B (natural killer cell stimulatory factor 2, cytotoxic lymphocyte maturation factor 2, p40) | CLMF\|CLMF2\|IL-12B\|NKSF\|NKSF2 | 5 | Interleukins |
| IL13 | 3596 | interleukin 13 | ALRH\|BHR1\|IL-13\|MGC116786\|MGC116788\|MGC116789\|P600 | 5 | Interleukins |
| IL15 | 3600 | interleukin 15 | IL-15\|MGC9721 | 4 | Interleukins |
| IL16 | 3603 | interleukin 16 (lymphocyte chemoattractant factor) | FLJ16806\|FLJ42735\|FLJ44234\|HsT19289\|IL-16\|LCF\|prIL-16 | 15 | Interleukins |
| IL17A | 3605 | interleukin 17A | CTLA8\|IL-17\|IL-17A\|IL17 | 6 | Interleukins |
| IL17B | 27190 | interleukin 17B | IL-17B\|IL-20\|MGC138900\|MGC138901\|ZCYTO7 | 5 | Interleukins |
| IL17C | 27189 | interleukin 17C | CX2\|IL-17C\|IL-21\|MGC126884\|MGC138401 | 16 | Interleukins |
| IL17D | 53342 | interleukin 17D | FLJ30846\|IL-17D\|IL-22\|IL-27\|IL27 | 13 | Interleukins |
| IL17F | 112744 | interleukin 17F | IL-17F\|ML-1\|ML1 | 6 | Interleukins |
| IL18 | 3606 | interleukin 18 (interferon-gamma-inducing factor) | IGIF\|IL-18\|IL-1g\|IL1F4\|MGC12320 | 11 | Interleukins |
| IL19 | 29949 | interleukin 19 | IL-10C\|MDA1\|NG.1\|ZMDA1 | 1 | Interleukins |
| IL1A | 3552 | interleukin 1, alpha | IL-1A\|IL1\|IL1-ALPHA\|IL1F1 | 2 | Interleukins |
| IL1B | 3553 | interleukin 1, beta | IL-1\|IL1-BETA\|IL1F2 | 2 | Interleukins |
| IL1F10 | 84639 | interleukin 1 family, member 10 (theta) | FIL1-theta\|FKSG75\|IL-1HY2\|IL1-theta\|MGC119831\|MGC119832\|MGC119833 | 2 | Interleukins |
| IL1F5 | 26525 | interleukin 1 family, member 5 (delta) | FIL1\|FIL1(DELTA)\|FIL1D\|IL1HY1\|IL1L1\|IL1RP3\|MGC29840 | 2 | Interleukins |
| IL1F6 | 27179 | interleukin 1 family, member 6 (epsilon) | FIL1\|FIL1(EPSILON)\|FIL1E\|IL-1F6\|IL1(EPSILON)\|MGC129552\|MGC129553 | 2 | Interleukins |
| IL1F7 | 27178 | interleukin 1 family, member 7 (zeta) | FIL1\|FIL1(ZETA)\|FIL1Z\|IL-1F7\|IL-1H4\|IL-1RP1\|IL1H4\|IL1RP1 | 2 | Interleukins |
| IL1F8 | 27177 | interleukin 1 family, member 8 (eta) | FIL1\|FIL1-(ETA)\|FIL1H\|IL-1F8\|IL-1H2\|IL1-ETA\|IL1H2\|MGC126880\|MGC126882 | 2 | Interleukins |
| IL1F9 | 56300 | interleukin 1 family, member 9 | IL-1F9\|IL-1H1\|IL-1RP2\|IL1E\|IL1H1\|IL1RP2 | 2 | Interleukins |
| IL1RN | 3557 | interleukin 1 receptor antagonist | ICIL-1RA\|IL-1ra3\|IL1F3\|IL1RA\|IRAP\|MGC10430 | 2 | Interleukins |
| IL2 | 3558 | interleukin 2 | IL-2\|TCGF\|lymphokine | 4 | Interleukins |
| IL20 | 50604 | interleukin 20 | IL-20\|IL10D\|MGC96907\|ZCYTO10 | 1 | Interleukins |
| IL21 | 59067 | interleukin 21 | IL-21\|Za11 | 4 | Interleukins |
| IL22 | 50616 | interleukin 22 | IL-21\|IL-22\|IL-D110\|IL-TIF\|IL21\|ILTIF\|MGC79382\|MGC79384\|TIFIL-23\|TIFa\|zcyto18 | 12 | Interleukins |
| IL23A | 51561 | interleukin 23, alpha subunit p19 | IL-23\|IL-23A\|IL23P19\|MGC79388\|P19\|SGRF | 12 | Interleukins |
| IL24 | 11009 | interleukin 24 | C49A\|FISP\|IL-24\|IL10B\|MDA7\|Mob-5\|ST16\|mda-7 | 1 | Interleukins |
| IL25 | 64806 | interleukin 25 | IL-17E\|IL-25\|IL17E | 14 | Interleukins |
| IL26 | 55801 | interleukin 26 | AK155\|IL-26 | 12 | Interleukins |
| IL27 | 246778 | interleukin 27 | IL-27\|IL-27A\|IL27p28\|IL30\|MGC71873\|p28 | 16 | Interleukins |
| IL28A | 282616 | interleukin 28A (interferon, lambda 2) | IFNL2\|IL-28A | 19 | Interleukins |
| IL28B | 282617 | interleukin 28B (interferon, lambda 3) | IFNL3\|IL-28B\|IL28C | 19 | Interleukins |
| IL29 | 282618 | interleukin 29 (interferon, lambda 1) | IFNL1\|IL-29 | 19 | Interleukins |
| IL3 | 3562 | interleukin 3 (colony-stimulating factor, multiple) | IL-3\|MCGF\|MGC79398\|MGC79399\|MULTI-CSF | 5 | Interleukins |
| IL31 | 386653 | interleukin 31 | IL-31 | 12 | Interleukins |
| IL32 | 9235 | interleukin 32 | IL-32alpha\|IL-32beta\|IL-32delta\|IL-32gamma\|NK4\|TAIF\|TAIFa\|TAIFb\|TAIFc\|TAIFd | 16 | Interleukins |
| IL33 | 90865 | interleukin 33 | C9orf26\|DKFZp586H0523\|DVS27\|NF-HEV\|NFEHEV\|RP11-575C20.2 | 9 | Interleukins |
| IL34 | 146433 | interleukin 34 | C16orf77\|IL-34\|MGC34647 | 16 | Interleukins |
| IL4 | 3565 | interleukin 4 | BCGF-1\|BCGF1\|BSF1\|IL-4\|MGC79402 | 5 | Interleukins |
| IL5 | 3567 | interleukin 5 (colony-stimulating factor, eosinophil) | EDF\|IL-5\|TRF | 5 | Interleukins |
| IL6 | 3569 | interleukin 6 (interferon, beta 2) | BSF2\|HGF\|HSF\|IFNB2\|IL-6 | 7 | Interleukins |
| IL6ST | 3572 | interleukin 6 signal transducer (gp130, oncostatin M receptor) | CD130\|CDw130\|GP130\|GP130-RAPS\|IL6R-beta | 5 | Interleukins |
| IL7 | 3574 | interleukin 7 | IL-7 | 8 | Interleukins |
| IL8 | 3576 | interleukin 8 | CXCL8\|GCP-1\|GCP1\|LECT\|LUCT\|LYNAP\|MDNCF\|MONAP\|NAF\|NAP-1\|NAP1 | 4 | Interleukins |
| IL9 | 3578 | interleukin 9 | HP40\|IL-9\|P40 | 5 | Interleukins |
| TXLNA | 200081 | taxilin alpha | DKFZp451J0118\|IL14\|MGC118870\|MGC118871\|RP4-622L5.4\|TXLN | 1 | Interleukins |
| IL10RA | 3587 | interleukin 10 receptor, alpha | CDW210A\|HIL-10R\|IL-10R1\|IL10R | 11 | Interleukins Receptor |
| IL10RB | 3588 | interleukin 10 receptor, beta | CDW210B\|CRF2-4\|CRFB4\|D21S58\|D21S66\|IL-10R2 | 21 | Interleukins Receptor |
| IL11RA | 3590 | interleukin 11 receptor, alpha | MGC2146 | 9 | Interleukins Receptor |
| IL11RB | 3591 | interleukin 11 receptor, beta | - | - | Interleukins Receptor |
| IL12RB1 | 3594 | interleukin 12 receptor, beta 1 | CD212\|IL-12R-BETA1\|IL12RB\|MGC34454 | 19 | Interleukins Receptor |
| IL12RB2 | 3595 | interleukin 12 receptor, beta 2 | - | 1 | Interleukins Receptor |
| IL13RA1 | 3597 | interleukin 13 receptor, alpha 1 | CD213A1\|IL-13Ra\|NR4 | X | Interleukins Receptor |
| IL13RA2 | 3598 | interleukin 13 receptor, alpha 2 | CD213A2\|CT19\|IL-13R\|IL13BP | X | Interleukins Receptor |
| IL15RA | 3601 | interleukin 15 receptor, alpha | MGC104179 | 10 | Interleukins Receptor |
| IL15RB | 3602 | interleukin 15 receptor, beta | - | - | Interleukins Receptor |
| IL17RA | 23765 | interleukin 17 receptor A | CD217\|CDw217\|IL-17RA\|IL17R\|MGC10262\|hIL-17R | 22 | Interleukins Receptor |
| IL17RB | 55540 | interleukin 17 receptor B | CRL4\|EVI27\|IL17BR\|IL17RH1\|MGC5245 | 3 | Interleukins Receptor |
| IL17RC | 84818 | interleukin 17 receptor C | FLJ95963\|FLJ96005\|IL17-RL\|IL17RL\|MGC10763 | 3 | Interleukins Receptor |
| IL17RD | 54756 | interleukin 17 receptor D | DKFZp434N1928\|FLJ35755\|IL-17RD\|IL17RLM\|MGC133309\|SEF | 3 | Interleukins Receptor |
| IL17RE | 132014 | interleukin 17 receptor E | FLJ23658\|MGC71884 | 3 | Interleukins Receptor |
| IL18R1 | 8809 | interleukin 18 receptor 1 | CD218a\|CDw218a\|IL-1Rrp\|IL18RA\|IL1RRP | 2 | Interleukins Receptor |
| IL18RAP | 8807 | interleukin 18 receptor accessory protein | ACPL\|CD218b\|CDw218b\|IL18RB\|MGC120589\|MGC120590 | 2 | Interleukins Receptor |
| IL1R1 | 3554 | interleukin 1 receptor, type I | CD121A\|D2S1473\|IL-1R-alpha\|IL1R\|IL1RA\|P80 | 2 | Interleukins Receptor |
| IL1R2 | 7850 | interleukin 1 receptor, type II | CD121b\|IL1RB\|MGC47725 | 2 | Interleukins Receptor |
| IL1RAP | 3556 | interleukin 1 receptor accessory protein | C3orf13\|FLJ37788\|IL-1RAcP\|IL1R3 | 3 | Interleukins Receptor |
| IL1RL1 | 9173 | interleukin 1 receptor-like 1 | DER4\|FIT-1\|MGC32623\|ST2\|ST2L\|ST2V\|T1 | 2 | Interleukins Receptor |
| IL1RL2 | 8808 | interleukin 1 receptor-like 2 | IL1R-rp2\|IL1RRP2 | 2 | Interleukins Receptor |
| IL20RA | 53832 | interleukin 20 receptor, alpha | FLJ40993\|IL-20R1\|ZCYTOR7 | 6 | Interleukins Receptor |
| IL20RB | 53833 | interleukin 20 receptor beta | DIRS1\|FNDC6\|IL-20R2\|MGC34923 | 3 | Interleukins Receptor |
| IL21R | 50615 | interleukin 21 receptor | MGC10967\|NILR | 16 | Interleukins Receptor |
| IL22RA1 | 58985 | interleukin 22 receptor, alpha 1 | CRF2-9\|IL22R\|IL22R1 | 1 | Interleukins Receptor |
| IL22RA2 | 116379 | interleukin 22 receptor, alpha 2 | CRF2-10\|CRF2-S1\|CRF2X\|IL-22BP\|MGC150509\|MGC150510 | 6 | Interleukins Receptor |
| IL23R | 149233 | interleukin 23 receptor | - | 1 | Interleukins Receptor |
| IL27RA | 9466 | interleukin 27 receptor, alpha | CRL1\|IL27R\|TCCR\|WSX1\|zcytor1 | 19 | Interleukins Receptor |
| IL28RA | 163702 | interleukin 28 receptor, alpha (interferon, lambda receptor) | CRF2/12\|IFNLR\|IFNLR1\|IL-28R1\|LICR2 | 1 | Interleukins Receptor |
| IL2RA | 3559 | interleukin 2 receptor, alpha | CD25\|IDDM10\|IL2R\|TCGFR | 10 | Interleukins Receptor |
| IL2RB | 3560 | interleukin 2 receptor, beta | CD122\|P70-75 | 22 | Interleukins Receptor |
| IL2RG | 3561 | interleukin 2 receptor, gamma (severe combined immunodeficiency) | CD132\|IMD4\|SCIDX\|SCIDX1 | X | Interleukins Receptor |
| IL31RA | 133396 | interleukin 31 receptor A | CRL\|CRL3\|GLM-R\|GLMR\|GPL\|IL-31RA\|MGC125346\|PRO21384 | 5 | Interleukins Receptor |
| IL3RA | 3563 | interleukin 3 receptor, alpha (low affinity) | CD123\|IL3R\|IL3RAY\|IL3RX\|IL3RY\|MGC34174\|hIL-3Ra | X\|Y | Interleukins Receptor |
| IL4R | 3566 | interleukin 4 receptor | CD124\|IL4RA | 16 | Interleukins Receptor |
| IL5RA | 3568 | interleukin 5 receptor, alpha | CD125\|CDw125\|HSIL5R3\|IL5R\|MGC26560 | 3 | Interleukins Receptor |
| IL6R | 3570 | interleukin 6 receptor | CD126\|IL-6R-1\|IL-6R-alpha\|IL6RA\|MGC104991 | 1 | Interleukins Receptor |
| IL7R | 3575 | interleukin 7 receptor | CD127\|CDW127\|IL-7R-alpha\|IL7RA\|ILRA | 5 | Interleukins Receptor |
| IL8RA | 3577 | interleukin 8 receptor, alpha | C-C\|C-C-CKR-1\|CD128\|CD181\|CDw128a\|CKR-1\|CMKAR1\|CXCR1\|IL8R1\|IL8RBA | 2 | Interleukins Receptor |
| IL8RB | 3579 | interleukin 8 receptor, beta | CD182\|CDw128b\|CMKAR2\|CXCR2\|IL8R2\|IL8RA | 2 | Interleukins Receptor |
| IL9R | 3581 | interleukin 9 receptor | CD129 | X\|Y | Interleukins Receptor |
| ST2 | 6761 | suppression of tumorigenicity 2 | - | 11 | Interleukins Receptor |
| HLA-A | 3105 | major histocompatibility complex, class I, A | FLJ26655\|HLAA | 6 | NaturalKiller Cell Cytotoxicity |
| HLA-B | 3106 | major histocompatibility complex, class I, B | AS\|HLA-B-7301\|HLA-B73\|HLAB\|HLAC\|MGC111087\|SPDA1 | 6 | NaturalKiller Cell Cytotoxicity |
| HLA-C | 3107 | major histocompatibility complex, class I, C | D6S204\|FLJ27082\|HLA-Cw\|HLA-Cw12\|HLA-JY3\|HLC-C\|PSORS1 | 6 | NaturalKiller Cell Cytotoxicity |
| HLA-E | 3133 | major histocompatibility complex, class I, E | DKFZp686P19218\|EA1.2\|EA2.1\|HLA-6.2\|MHC\|QA1 | 6 | NaturalKiller Cell Cytotoxicity |
| HLA-G | 3135 | major histocompatibility complex, class I, G | MHC-G | 6 | NaturalKiller Cell Cytotoxicity |
| KIR3DL1 | 3811 | killer cell immunoglobulin-like receptor, three domains, long cytoplasmic tail, 1 | CD158E1\|KIR\|MGC119726\|MGC119728\|MGC126589\|MGC126591\|NKAT3\|NKB1\|NKB1B | 19 | NaturalKiller Cell Cytotoxicity |
| KIR3DL2 | 3812 | killer cell immunoglobulin-like receptor, three domains, long cytoplasmic tail, 2 | CD158K\|MGC125321\|NKAT4\|NKAT4B\|p140 | 19 | NaturalKiller Cell Cytotoxicity |
| KIR2DL1 | 3802 | killer cell immunoglobulin-like receptor, two domains, long cytoplasmic tail, 1 | CD158A\|KIR-K64\|KIR221\|NKAT\|NKAT1\|p58.1 | 19 | NaturalKiller Cell Cytotoxicity |
| KIR2DL2 | 3803 | killer cell immunoglobulin-like receptor, two domains, long cytoplasmic tail, 2 | CD158B1\|CD158b\|NKAT6\|p58.2 | 19 | NaturalKiller Cell Cytotoxicity |
| KIR2DL3 | 3804 | killer cell immunoglobulin-like receptor, two domains, long cytoplasmic tail, 3 | CD158B2\|CD158b\|GL183\|KIR-023GB\|KIR-K7b\|KIR-K7c\|KIRCL23\|MGC129943\|NKAT\|NKAT2\|NKAT2A\|NKAT2B\|p58 | 19 | NaturalKiller Cell Cytotoxicity |
| KIR2DL4 | 3805 | killer cell immunoglobulin-like receptor, two domains, long cytoplasmic tail, 4 | CD158D\|G9P\|KIR103\|KIR103AS | 19 | NaturalKiller Cell Cytotoxicity |
| KIR2DL5A | 57292 | killer cell immunoglobulin-like receptor, two domains, long cytoplasmic tail, 5A | CD158F\|KIR2DL5\|KIR2DL5.1\|KIR2DL5.3 | 19 | NaturalKiller Cell Cytotoxicity |
| KLRC1 | 3821 | killer cell lectin-like receptor subfamily C, member 1 | CD159A\|MGC13374\|MGC59791\|NKG2\|NKG2A | 12 | NaturalKiller Cell Cytotoxicity |
| KLRC2 | 3822 | killer cell lectin-like receptor subfamily C, member 2 | CD159c\|MGC138244\|NKG2-C\|NKG2C | 12 | NaturalKiller Cell Cytotoxicity |
| KLRC3 | 3823 | killer cell lectin-like receptor subfamily C, member 3 | NKG2-E\|NKG2E | 12 | NaturalKiller Cell Cytotoxicity |
| KLRD1 | 3824 | killer cell lectin-like receptor subfamily D, member 1 | CD94 | 12 | NaturalKiller Cell Cytotoxicity |
| PTPN6 | 5777 | protein tyrosine phosphatase, non-receptor type 6 | HCP\|HCPH\|HPTP1C\|PTP-1C\|SH-PTP1\|SHP-1\|SHP-1L\|SHP1 | 12 | NaturalKiller Cell Cytotoxicity |
| PTPN11 | 5781 | protein tyrosine phosphatase, non-receptor type 11 | BPTP3\|CFC\|MGC14433\|NS1\|PTP-1D\|PTP2C\|SH-PTP2\|SH-PTP3\|SHP2 | 12 | NaturalKiller Cell Cytotoxicity |
| ICAM1 | 3383 | intercellular adhesion molecule 1 | BB2\|CD54\|P3.58 | 19 | NaturalKiller Cell Cytotoxicity |
| ICAM2 | 3384 | intercellular adhesion molecule 2 | CD102 | 17 | NaturalKiller Cell Cytotoxicity |
| ITGAL | 3683 | integrin, alpha L (antigen CD11A (p180), lymphocyte function-associated antigen 1; alpha polypeptide) | CD11A\|LFA-1\|LFA1A | 16 | NaturalKiller Cell Cytotoxicity |
| ITGB2 | 3689 | integrin, beta 2 (complement component 3 receptor 3 and 4 subunit) | CD18\|LAD\|LCAMB\|LFA-1\|MAC-1\|MF17\|MFI7 | 21 | NaturalKiller Cell Cytotoxicity |
| PTK2B | 2185 | PTK2B protein tyrosine kinase 2 beta | CADTK\|CAKB\|FADK2\|FAK2\|FRNK\|PKB\|PTK\|PYK2\|RAFTK | 8 | NaturalKiller Cell Cytotoxicity |
| VAV3 | 10451 | vav 3 guanine nucleotide exchange factor | FLJ40431 | 1 | NaturalKiller Cell Cytotoxicity |
| VAV1 | 7409 | vav 1 guanine nucleotide exchange factor | VAV | 19 | NaturalKiller Cell Cytotoxicity |
| VAV2 | 7410 | vav 2 guanine nucleotide exchange factor | - | 9 | NaturalKiller Cell Cytotoxicity |
| RAC1 | 5879 | ras-related C3 botulinum toxin substrate 1 (rho family, small GTP binding protein Rac1) | MGC111543\|MIG5\|TC-25\|p21-Rac1 | 7 | NaturalKiller Cell Cytotoxicity |
| RAC2 | 5880 | ras-related C3 botulinum toxin substrate 2 (rho family, small GTP binding protein Rac2) | EN-7\|Gx\|HSPC022 | 22 | NaturalKiller Cell Cytotoxicity |
| RAC3 | 5881 | ras-related C3 botulinum toxin substrate 3 (rho family, small GTP binding protein Rac3) | - | 17 | NaturalKiller Cell Cytotoxicity |
| PAK1 | 5058 | p21 protein (Cdc42/Rac)-activated kinase 1 | MGC130000\|MGC130001\|PAKalpha | 11 | NaturalKiller Cell Cytotoxicity |
| MAP2K1 | 5604 | mitogen-activated protein kinase kinase 1 | MAPKK1\|MEK1\|MKK1\|PRKMK1 | 15 | NaturalKiller Cell Cytotoxicity |
| MAP2K2 | 5605 | mitogen-activated protein kinase kinase 2 | FLJ26075\|MAPKK2\|MEK2\|MKK2\|PRKMK2 | 19 | NaturalKiller Cell Cytotoxicity |
| MAPK1 | 5594 | mitogen-activated protein kinase 1 | ERK\|ERK2\|ERT1\|MAPK2\|P42MAPK\|PRKM1\|PRKM2\|p38\|p40\|p41\|p41mapk | 22 | NaturalKiller Cell Cytotoxicity |
| MAPK3 | 5595 | mitogen-activated protein kinase 3 | ERK1\|HS44KDAP\|HUMKER1A\|MGC20180\|P44ERK1\|P44MAPK\|PRKM3 | 16 | NaturalKiller Cell Cytotoxicity |
| TNF | 7124 | tumor necrosis factor (TNF superfamily, member 2) | DIF\|TNF-alpha\|TNFA\|TNFSF2 | 6 | NaturalKiller Cell Cytotoxicity |
| CSF2 | 1437 | colony stimulating factor 2 (granulocyte-macrophage) | GMCSF\|MGC131935\|MGC138897 | 5 | NaturalKiller Cell Cytotoxicity |
| IFNG | 3458 | interferon, gamma | IFG\|IFI | 12 | NaturalKiller Cell Cytotoxicity |
| KIR2DS1 | 3806 | killer cell immunoglobulin-like receptor, two domains, short cytoplasmic tail, 1 | CD158H\|CD158a\|p50.1 | 19 | NaturalKiller Cell Cytotoxicity |
| KIR2DS3 | 3808 | killer cell immunoglobulin-like receptor, two domains, short cytoplasmic tail, 3 | NKAT7 | 19 | NaturalKiller Cell Cytotoxicity |
| KIR2DS4 | 3809 | killer cell immunoglobulin-like receptor, two domains, short cytoplasmic tail, 4 | CD158I\|KIR1D\|KIR412\|KKA3\|MGC120019\|MGC125315\|MGC125317\|NKAT8 | 19 | NaturalKiller Cell Cytotoxicity |
| KIR2DS5 | 3810 | killer cell immunoglobulin-like receptor, two domains, short cytoplasmic tail, 5 | CD158G\|NKAT9 | 19 | NaturalKiller Cell Cytotoxicity |
| NCR2 | 9436 | natural cytotoxicity triggering receptor 2 | CD336\|LY95\|NK-p44\|NKP44\|dJ149M18.1 | 6 | NaturalKiller Cell Cytotoxicity |
| TYROBP | 7305 | TYRO protein tyrosine kinase binding protein | DAP12\|KARAP\|PLOSL | 19 | NaturalKiller Cell Cytotoxicity |
| LCK | 3932 | lymphocyte-specific protein tyrosine kinase | YT16\|p56lck\|pp58lck | 1 | NaturalKiller Cell Cytotoxicity |
| FCGR3A | 2214 | Fc fragment of IgG, low affinity IIIa, receptor (CD16a) | CD16\|CD16A\|FCG3\|FCGR3\|FCGRIII\|FCR-10\|FCRIII\|FCRIIIA\|IGFR3 | 1 | NaturalKiller Cell Cytotoxicity |
| FCGR3B | 2215 | Fc fragment of IgG, low affinity IIIb, receptor (CD16b) | CD16\|CD16b\|FCG3\|FCGR3 | 1 | NaturalKiller Cell Cytotoxicity |
| LOC652578 | 652578 | similar to Fc fragment of IgG, low affinity IIIa, receptor for (CD16) | - | Un | NaturalKiller Cell Cytotoxicity |
| NCR1 | 9437 | natural cytotoxicity triggering receptor 1 | CD335\|FLJ99094\|LY94\|NK-p46\|NKP46 | 19 | NaturalKiller Cell Cytotoxicity |
| NCR3 | 259197 | natural cytotoxicity triggering receptor 3 | 1C7\|CD337\|LY117\|MALS\|NKp30 | 6 | NaturalKiller Cell Cytotoxicity |
| FCER1G | 2207 | Fc fragment of IgE, high affinity I, receptor for; gamma polypeptide | FCRG | 1 | NaturalKiller Cell Cytotoxicity |
| CD247 | 919 | CD247 molecule | CD3-ZETA\|CD3H\|CD3Q\|CD3Z\|T3Z\|TCRZ | 1 | NaturalKiller Cell Cytotoxicity |
| ZAP70 | 7535 | zeta-chain (TCR) associated protein kinase 70kDa | FLJ17670\|FLJ17679\|SRK\|STD\|TZK\|ZAP-70 | 2 | NaturalKiller Cell Cytotoxicity |
| SYK | 6850 | spleen tyrosine kinase | DKFZp313N1010\|FLJ25043\|FLJ37489 | 9 | NaturalKiller Cell Cytotoxicity |
| LCP2 | 3937 | lymphocyte cytosolic protein 2 (SH2 domain containing leukocyte protein of 76kDa) | SLP-76\|SLP76 | 5 | NaturalKiller Cell Cytotoxicity |
| LAT | 27040 | linker for activation of T cells | LAT1\|pp36 | 16 | NaturalKiller Cell Cytotoxicity |
| PLCG1 | 5335 | phospholipase C, gamma 1 | PLC-II\|PLC1\|PLC148\|PLCgamma1 | 20 | NaturalKiller Cell Cytotoxicity |
| PLCG2 | 5336 | phospholipase C, gamma 2 (phosphatidylinositol-specific) | - | 16 | NaturalKiller Cell Cytotoxicity |
| SH3BP2 | 6452 | SH3-domain binding protein 2 | 3BP2\|CRBM\|CRPM\|FLJ42079\|FLJ54978\|RES4-23 | 4 | NaturalKiller Cell Cytotoxicity |
| PIK3CA | 5290 | phosphoinositide-3-kinase, catalytic, alpha polypeptide | MGC142161\|MGC142163\|PI3K\|p110-alpha | 3 | NaturalKiller Cell Cytotoxicity |
| PIK3CB | 5291 | phosphoinositide-3-kinase, catalytic, beta polypeptide | DKFZp779K1237\|MGC133043\|PI3K\|PI3KCB\|PI3Kbeta\|PIK3C1\|p110-BETA | 3 | NaturalKiller Cell Cytotoxicity |
| PIK3CD | 5293 | phosphoinositide-3-kinase, catalytic, delta polypeptide | p110D | 1 | NaturalKiller Cell Cytotoxicity |
| PIK3CG | 5294 | phosphoinositide-3-kinase, catalytic, gamma polypeptide | PI3CG\|PI3K\|PI3Kgamma\|PIK3 | 7 | NaturalKiller Cell Cytotoxicity |
| PIK3R5 | 23533 | phosphoinositide-3-kinase, regulatory subunit 5 | F730038I15Rik\|FOAP-2\|P101-PI3K\|p101 | 17 | NaturalKiller Cell Cytotoxicity |
| PIK3R1 | 5295 | phosphoinositide-3-kinase, regulatory subunit 1 (alpha) | GRB1\|p85\|p85-ALPHA | 5 | NaturalKiller Cell Cytotoxicity |
| PIK3R2 | 5296 | phosphoinositide-3-kinase, regulatory subunit 2 (beta) | P85B\|p85\|p85-BETA | 19 | NaturalKiller Cell Cytotoxicity |
| PIK3R3 | 8503 | phosphoinositide-3-kinase, regulatory subunit 3 (gamma) | DKFZp686P05226\|FLJ41892\|p55\|p55-GAMMA | 1 | NaturalKiller Cell Cytotoxicity |
| FYN | 2534 | FYN oncogene related to SRC, FGR, YES | MGC45350\|SLK\|SYN | 6 | NaturalKiller Cell Cytotoxicity |
| SHC2 | 25759 | SHC (Src homology 2 domain containing) transforming protein 2 | SCK\|SHCB\|SLI | 19 | NaturalKiller Cell Cytotoxicity |
| SHC4 | 399694 | SHC (Src homology 2 domain containing) family, member 4 | MGC34023\|RaLP\|SHCD | 15 | NaturalKiller Cell Cytotoxicity |
| SHC3 | 53358 | SHC (Src homology 2 domain containing) transforming protein 3 | N-Shc\|NSHC\|RAI\|SHCC | 9 | NaturalKiller Cell Cytotoxicity |
| SHC1 | 6464 | SHC (Src homology 2 domain containing) transforming protein 1 | FLJ26504\|SHC\|SHCA | 1 | NaturalKiller Cell Cytotoxicity |
| GRB2 | 2885 | growth factor receptor-bound protein 2 | ASH\|EGFRBP-GRB2\|Grb3-3\|MST084\|MSTP084 | 17 | NaturalKiller Cell Cytotoxicity |
| SOS1 | 6654 | son of sevenless homolog 1 (Drosophila) | GF1\|GGF1\|GINGF\|HGF\|NS4 | 2 | NaturalKiller Cell Cytotoxicity |
| SOS2 | 6655 | son of sevenless homolog 2 (Drosophila) | FLJ25596 | 14 | NaturalKiller Cell Cytotoxicity |
| HRAS | 3265 | v-Ha-ras Harvey rat sarcoma viral oncogene homolog | C-BAS/HAS\|C-H-RAS\|C-HA-RAS1\|CTLO\|H-RASIDX\|HAMSV\|HRAS1\|K-RAS\|N-RAS\|RASH1 | 11 | NaturalKiller Cell Cytotoxicity |
| KRAS | 3845 | v-Ki-ras2 Kirsten rat sarcoma viral oncogene homolog | C-K-RAS\|K-RAS2A\|K-RAS2B\|K-RAS4A\|K-RAS4B\|KI-RAS\|KRAS1\|KRAS2\|NS3\|RASK2 | 12 | NaturalKiller Cell Cytotoxicity |
| NRAS | 4893 | neuroblastoma RAS viral (v-ras) oncogene homolog | ALPS4\|N-ras\|NRAS1 | 1 | NaturalKiller Cell Cytotoxicity |
| ARAF | 369 | v-raf murine sarcoma 3611 viral oncogene homolog | A-RAF\|ARAF1\|PKS2\|RAFA1 | X | NaturalKiller Cell Cytotoxicity |
| BRAF | 673 | v-raf murine sarcoma viral oncogene homolog B1 | B-RAF1\|BRAF1\|FLJ95109\|MGC126806\|MGC138284\|RAFB1 | 7 | NaturalKiller Cell Cytotoxicity |
| RAF1 | 5894 | v-raf-1 murine leukemia viral oncogene homolog 1 | CRAF\|NS5\|Raf-1\|c-Raf | 3 | NaturalKiller Cell Cytotoxicity |
| MICA | 4276 | MHC class I polypeptide-related sequence A | FLJ60820\|MGC111087\|PERB11.1 | 6 | NaturalKiller Cell Cytotoxicity |
| MICB | 4277 | MHC class I polypeptide-related sequence B | PERB11.2 | 6 | NaturalKiller Cell Cytotoxicity |
| ULBP3 | 79465 | UL16 binding protein 3 | RAET1N | 6 | NaturalKiller Cell Cytotoxicity |
| ULBP2 | 80328 | UL16 binding protein 2 | N2DL2\|RAET1H | 6 | NaturalKiller Cell Cytotoxicity |
| ULBP1 | 80329 | UL16 binding protein 1 | RAET1I | 6 | NaturalKiller Cell Cytotoxicity |
| KLRK1 | 22914 | killer cell lectin-like receptor subfamily K, member 1 | CD314\|D12S2489E\|FLJ17759\|FLJ75772\|KLR\|NKG2-D\|NKG2D | 12 | NaturalKiller Cell Cytotoxicity |
| HCST | 10870 | hematopoietic cell signal transducer | DAP10\|DKFZP586C1522\|KAP10\|PIK3AP | 19 | NaturalKiller Cell Cytotoxicity |
| CD48 | 962 | CD48 molecule | BCM1\|BLAST\|BLAST1\|MEM-102\|SLAMF2\|hCD48\|mCD48 | 1 | NaturalKiller Cell Cytotoxicity |
| CD244 | 51744 | CD244 molecule, natural killer cell receptor 2B4 | 2B4\|NAIL\|NKR2B4\|Nmrk\|SLAMF4 | 1 | NaturalKiller Cell Cytotoxicity |
| PPP3CA | 5530 | protein phosphatase 3 (formerly 2B), catalytic subunit, alpha isoform | CALN\|CALNA\|CALNA1\|CCN1\|CNA1\|PPP2B | 4 | NaturalKiller Cell Cytotoxicity |
| PPP3CB | 5532 | protein phosphatase 3 (formerly 2B), catalytic subunit, beta isoform | CALNA2\|CALNB | 10 | NaturalKiller Cell Cytotoxicity |
| PPP3CC | 5533 | protein phosphatase 3 (formerly 2B), catalytic subunit, gamma isoform | CALNA3 | 8 | NaturalKiller Cell Cytotoxicity |
| CHP | 11261 | calcium binding protein P22 | SLC9A1BP | 15 | NaturalKiller Cell Cytotoxicity |
| PPP3R1 | 5534 | protein phosphatase 3 (formerly 2B), regulatory subunit B, alpha isoform | CALNB1\|CNB\|CNB1 | 2 | NaturalKiller Cell Cytotoxicity |
| PPP3R2 | 5535 | protein phosphatase 3 (formerly 2B), regulatory subunit B, beta isoform | PPP3RL | 9 | NaturalKiller Cell Cytotoxicity |
| CHP2 | 63928 | calcineurin B homologous protein 2 | - | 16 | NaturalKiller Cell Cytotoxicity |
| NFAT5 | 10725 | nuclear factor of activated T-cells 5, tonicity-responsive | KIAA0827\|NF-AT5\|NFATL1\|NFATZ\|OREBP\|TONEBP | 16 | NaturalKiller Cell Cytotoxicity |
| NFATC1 | 4772 | nuclear factor of activated T-cells, cytoplasmic, calcineurin-dependent 1 | MGC138448\|NF-ATC\|NFAT2\|NFATc | 18 | NaturalKiller Cell Cytotoxicity |
| NFATC2 | 4773 | nuclear factor of activated T-cells, cytoplasmic, calcineurin-dependent 2 | NFAT1\|NFATP | 20 | NaturalKiller Cell Cytotoxicity |
| NFATC3 | 4775 | nuclear factor of activated T-cells, cytoplasmic, calcineurin-dependent 3 | NFAT4\|NFATX | 16 | NaturalKiller Cell Cytotoxicity |
| NFATC4 | 4776 | nuclear factor of activated T-cells, cytoplasmic, calcineurin-dependent 4 | NF-ATc4\|NFAT3 | 14 | NaturalKiller Cell Cytotoxicity |
| PRKCA | 5578 | protein kinase C, alpha | AAG6\|MGC129900\|MGC129901\|PKC-alpha\|PKCA\|PRKACA | 17 | NaturalKiller Cell Cytotoxicity |
| PRKCB | 5579 | protein kinase C, beta | MGC41878\|PKC-beta\|PKCB\|PRKCB1\|PRKCB2 | 16 | NaturalKiller Cell Cytotoxicity |
| PRKCG | 5582 | protein kinase C, gamma | MGC57564\|PKC-gamma\|PKCC\|PKCG\|SCA14 | 19 | NaturalKiller Cell Cytotoxicity |
| SH2D1B | 117157 | SH2 domain containing 1B | EAT2 | 1 | NaturalKiller Cell Cytotoxicity |
| SH2D1A | 4068 | SH2 domain protein 1A | DSHP\|EBVS\|FLJ18687\|FLJ92177\|IMD5\|LYP\|MTCP1\|SAP\|XLP\|XLPD | X | NaturalKiller Cell Cytotoxicity |
| IFNGR1 | 3459 | interferon gamma receptor 1 | CD119\|FLJ45734\|IFNGR | 6 | NaturalKiller Cell Cytotoxicity |
| IFNGR2 | 3460 | interferon gamma receptor 2 (interferon gamma transducer 1) | AF-1\|IFGR2\|IFNGT1 | 21 | NaturalKiller Cell Cytotoxicity |
| IFNA1 | 3439 | interferon, alpha 1 | IFL\|IFN\|IFN-ALPHA\|IFNA13\|IFNA@\|MGC138207\|MGC138505\|MGC138507 | 9 | NaturalKiller Cell Cytotoxicity |
| IFNA2 | 3440 | interferon, alpha 2 | IFNA\|INFA2\|MGC125764\|MGC125765 | 9 | NaturalKiller Cell Cytotoxicity |
| IFNA4 | 3441 | interferon, alpha 4 | INFA4\|MGC142200 | 9 | NaturalKiller Cell Cytotoxicity |
| IFNA5 | 3442 | interferon, alpha 5 | INFA5 | 9 | NaturalKiller Cell Cytotoxicity |
| IFNA6 | 3443 | interferon, alpha 6 | - | 9 | NaturalKiller Cell Cytotoxicity |
| IFNA7 | 3444 | interferon, alpha 7 | IFNA-J | 9 | NaturalKiller Cell Cytotoxicity |
| IFNA8 | 3445 | interferon, alpha 8 | - | 9 | NaturalKiller Cell Cytotoxicity |
| IFNA10 | 3446 | interferon, alpha 10 | MGC119878\|MGC119879 | 9 | NaturalKiller Cell Cytotoxicity |
| IFNA13 | 3447 | interferon, alpha 13 | - | 9 | NaturalKiller Cell Cytotoxicity |
| IFNA14 | 3448 | interferon, alpha 14 | LEIF2H\|MGC125756\|MGC125757 | 9 | NaturalKiller Cell Cytotoxicity |
| IFNA16 | 3449 | interferon, alpha 16 | - | 9 | NaturalKiller Cell Cytotoxicity |
| IFNA17 | 3451 | interferon, alpha 17 | IFNA\|INFA\|LEIF2C1 | 9 | NaturalKiller Cell Cytotoxicity |
| IFNA21 | 3452 | interferon, alpha 21 | MGC126687\|MGC126689 | 9 | NaturalKiller Cell Cytotoxicity |
| IFNB1 | 3456 | interferon, beta 1, fibroblast | IFB\|IFF\|IFNB\|MGC96956 | 9 | NaturalKiller Cell Cytotoxicity |
| IFNAR1 | 3454 | interferon (alpha, beta and omega) receptor 1 | AVP\|IFN-alpha-REC\|IFNAR\|IFNBR\|IFRC | 21 | NaturalKiller Cell Cytotoxicity |
| IFNAR2 | 3455 | interferon (alpha, beta and omega) receptor 2 | IFN-R\|IFN-alpha-REC\|IFNABR\|IFNARB | 21 | NaturalKiller Cell Cytotoxicity |
| TNFSF10 | 8743 | tumor necrosis factor (ligand) superfamily, member 10 | APO2L\|Apo-2L\|CD253\|TL2\|TRAIL | 3 | NaturalKiller Cell Cytotoxicity |
| TNFRSF10D | 8793 | tumor necrosis factor receptor superfamily, member 10d, decoy with truncated death domain | CD264\|DCR2\|TRAILR4\|TRUNDD | 8 | NaturalKiller Cell Cytotoxicity |
| TNFRSF10C | 8794 | tumor necrosis factor receptor superfamily, member 10c, decoy without an intracellular domain | CD263\|DCR1\|LIT\|MGC149501\|MGC149502\|TRAILR3\|TRID | 8 | NaturalKiller Cell Cytotoxicity |
| TNFRSF10B | 8795 | tumor necrosis factor receptor superfamily, member 10b | CD262\|DR5\|KILLER\|KILLER/DR5\|TRAIL-R2\|TRAILR2\|TRICK2\|TRICK2A\|TRICK2B\|TRICKB\|ZTNFR9 | 8 | NaturalKiller Cell Cytotoxicity |
| TNFRSF10A | 8797 | tumor necrosis factor receptor superfamily, member 10a | APO2\|CD261\|DR4\|MGC9365\|TRAILR-1\|TRAILR1 | 8 | NaturalKiller Cell Cytotoxicity |
| FASLG | 356 | Fas ligand (TNF superfamily, member 6) | APT1LG1\|CD178\|CD95L\|FASL\|TNFSF6 | 1 | NaturalKiller Cell Cytotoxicity |
| FAS | 355 | Fas (TNF receptor superfamily, member 6) | ALPS1A\|APO-1\|APT1\|CD95\|FAS1\|FASTM\|TNFRSF6 | 10 | NaturalKiller Cell Cytotoxicity |
| GZMB | 3002 | granzyme B (granzyme 2, cytotoxic T-lymphocyte-associated serine esterase 1) | CCPI\|CGL-1\|CGL1\|CSP-B\|CSPB\|CTLA1\|CTSGL1\|HLP\|SECT | 14 | NaturalKiller Cell Cytotoxicity |
| PRF1 | 5551 | perforin 1 (pore forming protein) | FLH2\|HPLH2\|MGC65093\|P1\|PFN1\|PFP | 10 | NaturalKiller Cell Cytotoxicity |
| CASP3 | 836 | caspase 3, apoptosis-related cysteine peptidase | CPP32\|CPP32B\|SCA-1 | 4 | NaturalKiller Cell Cytotoxicity |
| BID | 637 | BH3 interacting domain death agonist | FP497\|MGC15319\|MGC42355 | 22 | NaturalKiller Cell Cytotoxicity |
| CD3D | 915 | CD3d molecule, delta (CD3-TCR complex) | CD3-DELTA\|T3D | 11 | TCRsignalingPathway |
| CD3E | 916 | CD3e molecule, epsilon (CD3-TCR complex) | FLJ18683\|T3E\|TCRE | 11 | TCRsignalingPathway |
| CD3G | 917 | CD3g molecule, gamma (CD3-TCR complex) | CD3-GAMMA\|FLJ17620\|FLJ17664\|FLJ79544\|FLJ94613\|MGC138597\|T3G | 11 | TCRsignalingPathway |
| CD247 | 919 | CD247 molecule | CD3-ZETA\|CD3H\|CD3Q\|CD3Z\|T3Z\|TCRZ | 1 | TCRsignalingPathway |
| CD4 | 920 | CD4 molecule | CD4mut | 12 | TCRsignalingPathway |
| CD8A | 925 | CD8a molecule | CD8\|Leu2\|MAL\|p32 | 2 | TCRsignalingPathway |
| CD8B | 926 | CD8b molecule | CD8B1\|LYT3\|Leu2\|Ly3\|MGC119115 | 2 | TCRsignalingPathway |
| PTPRC | 5788 | protein tyrosine phosphatase, receptor type, C | B220\|CD45\|CD45R\|GP180\|LCA\|LY5\|T200 | 1 | TCRsignalingPathway |
| LCK | 3932 | lymphocyte-specific protein tyrosine kinase | YT16\|p56lck\|pp58lck | 1 | TCRsignalingPathway |
| FYN | 2534 | FYN oncogene related to SRC, FGR, YES | MGC45350\|SLK\|SYN | 6 | TCRsignalingPathway |
| ZAP70 | 7535 | zeta-chain (TCR) associated protein kinase 70kDa | FLJ17670\|FLJ17679\|SRK\|STD\|TZK\|ZAP-70 | 2 | TCRsignalingPathway |
| LCP2 | 3937 | lymphocyte cytosolic protein 2 (SH2 domain containing leukocyte protein of 76kDa) | SLP-76\|SLP76 | 5 | TCRsignalingPathway |
| LAT | 27040 | linker for activation of T cells | LAT1\|pp36 | 16 | TCRsignalingPathway |
| ITK | 3702 | IL2-inducible T-cell kinase | EMT\|LYK\|MGC126257\|MGC126258\|PSCTK2 | 5 | TCRsignalingPathway |
| TEC | 7006 | tec protein tyrosine kinase | MGC126760\|MGC126762\|PSCTK4 | 4 | TCRsignalingPathway |
| NCK1 | 4690 | NCK adaptor protein 1 | MGC12668\|NCK\|NCKalpha | 3 | TCRsignalingPathway |
| NCK2 | 8440 | NCK adaptor protein 2 | GRB4\|NCKbeta | 2 | TCRsignalingPathway |
| VAV3 | 10451 | vav 3 guanine nucleotide exchange factor | FLJ40431 | 1 | TCRsignalingPathway |
| VAV1 | 7409 | vav 1 guanine nucleotide exchange factor | VAV | 19 | TCRsignalingPathway |
| VAV2 | 7410 | vav 2 guanine nucleotide exchange factor | - | 9 | TCRsignalingPathway |
| GRAP2 | 9402 | GRB2-related adaptor protein 2 | GADS\|GRAP-2\|GRB2L\|GRBLG\|GRID\|GRPL\|GrbX\|Grf40\|Mona\|P38 | 22 | TCRsignalingPathway |
| GRB2 | 2885 | growth factor receptor-bound protein 2 | ASH\|EGFRBP-GRB2\|Grb3-3\|MST084\|MSTP084 | 17 | TCRsignalingPathway |
| PAK1 | 5058 | p21 protein (Cdc42/Rac)-activated kinase 1 | MGC130000\|MGC130001\|PAKalpha | 11 | TCRsignalingPathway |
| PAK2 | 5062 | p21 protein (Cdc42/Rac)-activated kinase 2 | PAK65\|PAKgamma | 3 | TCRsignalingPathway |
| PAK3 | 5063 | p21 protein (Cdc42/Rac)-activated kinase 3 | CDKN1A\|MRX30\|MRX47\|OPHN3\|PAK3beta\|bPAK\|hPAK3 | X | TCRsignalingPathway |
| PAK4 | 10298 | p21 protein (Cdc42/Rac)-activated kinase 4 | - | 19 | TCRsignalingPathway |
| PAK6 | 56924 | p21 protein (Cdc42/Rac)-activated kinase 6 | PAK5 | 15 | TCRsignalingPathway |
| PAK7 | 57144 | p21 protein (Cdc42/Rac)-activated kinase 7 | KIAA1264\|MGC26232\|PAK5 | 20 | TCRsignalingPathway |
| RHOA | 387 | ras homolog gene family, member A | ARH12\|ARHA\|RHO12\|RHOH12 | 3 | TCRsignalingPathway |
| CDC42 | 998 | cell division cycle 42 (GTP binding protein, 25kDa) | CDC42Hs\|G25K | 1 | TCRsignalingPathway |
| PPP3CA | 5530 | protein phosphatase 3 (formerly 2B), catalytic subunit, alpha isoform | CALN\|CALNA\|CALNA1\|CCN1\|CNA1\|PPP2B | 4 | TCRsignalingPathway |
| PPP3CB | 5532 | protein phosphatase 3 (formerly 2B), catalytic subunit, beta isoform | CALNA2\|CALNB | 10 | TCRsignalingPathway |
| PPP3CC | 5533 | protein phosphatase 3 (formerly 2B), catalytic subunit, gamma isoform | CALNA3 | 8 | TCRsignalingPathway |
| CHP | 11261 | calcium binding protein P22 | SLC9A1BP | 15 | TCRsignalingPathway |
| PPP3R1 | 5534 | protein phosphatase 3 (formerly 2B), regulatory subunit B, alpha isoform | CALNB1\|CNB\|CNB1 | 2 | TCRsignalingPathway |
| PPP3R2 | 5535 | protein phosphatase 3 (formerly 2B), regulatory subunit B, beta isoform | PPP3RL | 9 | TCRsignalingPathway |
| CHP2 | 63928 | calcineurin B homologous protein 2 | - | 16 | TCRsignalingPathway |
| NFAT5 | 10725 | nuclear factor of activated T-cells 5, tonicity-responsive | KIAA0827\|NF-AT5\|NFATL1\|NFATZ\|OREBP\|TONEBP | 16 | TCRsignalingPathway |
| NFATC1 | 4772 | nuclear factor of activated T-cells, cytoplasmic, calcineurin-dependent 1 | MGC138448\|NF-ATC\|NFAT2\|NFATc | 18 | TCRsignalingPathway |
| NFATC2 | 4773 | nuclear factor of activated T-cells, cytoplasmic, calcineurin-dependent 2 | NFAT1\|NFATP | 20 | TCRsignalingPathway |
| NFATC3 | 4775 | nuclear factor of activated T-cells, cytoplasmic, calcineurin-dependent 3 | NFAT4\|NFATX | 16 | TCRsignalingPathway |
| NFATC4 | 4776 | nuclear factor of activated T-cells, cytoplasmic, calcineurin-dependent 4 | NF-ATc4\|NFAT3 | 14 | TCRsignalingPathway |
| SOS1 | 6654 | son of sevenless homolog 1 (Drosophila) | GF1\|GGF1\|GINGF\|HGF\|NS4 | 2 | TCRsignalingPathway |
| SOS2 | 6655 | son of sevenless homolog 2 (Drosophila) | FLJ25596 | 14 | TCRsignalingPathway |
| HRAS | 3265 | v-Ha-ras Harvey rat sarcoma viral oncogene homolog | C-BAS/HAS\|C-H-RAS\|C-HA-RAS1\|CTLO\|H-RASIDX\|HAMSV\|HRAS1\|K-RAS\|N-RAS\|RASH1 | 11 | TCRsignalingPathway |
| KRAS | 3845 | v-Ki-ras2 Kirsten rat sarcoma viral oncogene homolog | C-K-RAS\|K-RAS2A\|K-RAS2B\|K-RAS4A\|K-RAS4B\|KI-RAS\|KRAS1\|KRAS2\|NS3\|RASK2 | 12 | TCRsignalingPathway |
| NRAS | 4893 | neuroblastoma RAS viral (v-ras) oncogene homolog | ALPS4\|N-ras\|NRAS1 | 1 | TCRsignalingPathway |
| FOS | 2353 | v-fos FBJ murine osteosarcoma viral oncogene homolog | AP-1\|C-FOS | 14 | TCRsignalingPathway |
| JUN | 3725 | jun oncogene | AP-1\|AP1\|c-Jun | 1 | TCRsignalingPathway |
| CARD11 | 84433 | caspase recruitment domain family, member 11 | BIMP3\|CARMA1\|MGC133069 | 7 | TCRsignalingPathway |
| BCL10 | 8915 | B-cell CLL/lymphoma 10 | CARMEN\|CIPER\|CLAP\|c-E10\|mE10 | 1 | TCRsignalingPathway |
| MALT1 | 10892 | mucosa associated lymphoid tissue lymphoma translocation gene 1 | DKFZp434L132\|MLT\|MLT1 | 18 | TCRsignalingPathway |
| CHUK | 1147 | conserved helix-loop-helix ubiquitous kinase | IKBKA\|IKK-alpha\|IKK1\|IKKA\|NFKBIKA\|TCF16 | 10 | TCRsignalingPathway |
| IKBKB | 3551 | inhibitor of kappa light polypeptide gene enhancer in B-cells, kinase beta | FLJ40509\|IKK-beta\|IKK2\|IKKB\|MGC131801\|NFKBIKB | 8 | TCRsignalingPathway |
| IKBKG | 8517 | inhibitor of kappa light polypeptide gene enhancer in B-cells, kinase gamma | AMCBX1\|FIP-3\|FIP3\|Fip3p\|IKK-gamma\|IP\|IP1\|IP2\|IPD2\|NEMO | X | TCRsignalingPathway |
| NFKB1 | 4790 | nuclear factor of kappa light polypeptide gene enhancer in B-cells 1 | DKFZp686C01211\|EBP-1\|KBF1\|MGC54151\|NF-kappa-B\|NFKB-p105\|NFKB-p50\|p105\|p50 | 4 | TCRsignalingPathway |
| RELA | 5970 | v-rel reticuloendotheliosis viral oncogene homolog A (avian) | MGC131774\|NFKB3\|p65 | 11 | TCRsignalingPathway |
| NFKBIA | 4792 | nuclear factor of kappa light polypeptide gene enhancer in B-cells inhibitor, alpha | IKBA\|MAD-3\|NFKBI | 14 | TCRsignalingPathway |
| NFKBIB | 4793 | nuclear factor of kappa light polypeptide gene enhancer in B-cells inhibitor, beta | IKBB\|TRIP9 | 19 | TCRsignalingPathway |
| NFKBIE | 4794 | nuclear factor of kappa light polypeptide gene enhancer in B-cells inhibitor, epsilon | IKBE | 6 | TCRsignalingPathway |
| CD28 | 940 | CD28 molecule | MGC138290\|Tp44 | 2 | TCRsignalingPathway |
| ICOS | 29851 | inducible T-cell co-stimulator | AILIM\|CD278\|MGC39850 | 2 | TCRsignalingPathway |
| CD40LG | 959 | CD40 ligand | CD154\|CD40L\|HIGM1\|IGM\|IMD3\|T-BAM\|TNFSF5\|TRAP\|gp39\|hCD40L | X | TCRsignalingPathway |
| PIK3R5 | 23533 | phosphoinositide-3-kinase, regulatory subunit 5 | F730038I15Rik\|FOAP-2\|P101-PI3K\|p101 | 17 | TCRsignalingPathway |
| PIK3R1 | 5295 | phosphoinositide-3-kinase, regulatory subunit 1 (alpha) | GRB1\|p85\|p85-ALPHA | 5 | TCRsignalingPathway |
| PIK3R2 | 5296 | phosphoinositide-3-kinase, regulatory subunit 2 (beta) | P85B\|p85\|p85-BETA | 19 | TCRsignalingPathway |
| PIK3R3 | 8503 | phosphoinositide-3-kinase, regulatory subunit 3 (gamma) | DKFZp686P05226\|FLJ41892\|p55\|p55-GAMMA | 1 | TCRsignalingPathway |
| PIK3CA | 5290 | phosphoinositide-3-kinase, catalytic, alpha polypeptide | MGC142161\|MGC142163\|PI3K\|p110-alpha | 3 | TCRsignalingPathway |
| PIK3CB | 5291 | phosphoinositide-3-kinase, catalytic, beta polypeptide | DKFZp779K1237\|MGC133043\|PI3K\|PI3KCB\|PI3Kbeta\|PIK3C1\|p110-BETA | 3 | TCRsignalingPathway |
| PIK3CD | 5293 | phosphoinositide-3-kinase, catalytic, delta polypeptide | p110D | 1 | TCRsignalingPathway |
| PIK3CG | 5294 | phosphoinositide-3-kinase, catalytic, gamma polypeptide | PI3CG\|PI3K\|PI3Kgamma\|PIK3 | 7 | TCRsignalingPathway |
| AKT3 | 10000 | v-akt murine thymoma viral oncogene homolog 3 (protein kinase B, gamma) | DKFZp434N0250\|PKB-GAMMA\|PKBG\|PRKBG\|RAC-PK-gamma\|RAC-gamma\|STK-2 | 1 | TCRsignalingPathway |
| AKT1 | 207 | v-akt murine thymoma viral oncogene homolog 1 | AKT\|MGC99656\|PKB\|PKB-ALPHA\|PRKBA\|RAC\|RAC-ALPHA | 14 | TCRsignalingPathway |
| AKT2 | 208 | v-akt murine thymoma viral oncogene homolog 2 | PKBB\|PKBBETA\|PRKBB\|RAC-BETA | 19 | TCRsignalingPathway |
| MAP3K8 | 1326 | mitogen-activated protein kinase kinase kinase 8 | COT\|EST\|ESTF\|FLJ10486\|TPL2\|Tpl-2\|c-COT | 10 | TCRsignalingPathway |
| MAP3K14 | 9020 | mitogen-activated protein kinase kinase kinase 14 | FTDCR1B\|HS\|HSNIK\|NIK | 17 | TCRsignalingPathway |
| PDCD1 | 5133 | programmed cell death 1 | CD279\|PD1\|SLEB2\|hPD-1\|hPD-l | 2 | TCRsignalingPathway |
| CTLA4 | 1493 | cytotoxic T-lymphocyte-associated protein 4 | CD\|CD152\|CELIAC3\|CTLA-4\|GSE\|ICOS\|IDDM12 | 2 | TCRsignalingPathway |
| PTPN6 | 5777 | protein tyrosine phosphatase, non-receptor type 6 | HCP\|HCPH\|HPTP1C\|PTP-1C\|SH-PTP1\|SHP-1\|SHP-1L\|SHP1 | 12 | TCRsignalingPathway |
| CBLC | 23624 | Cas-Br-M (murine) ecotropic retroviral transforming sequence c | CBL-3\|CBL-SL\|RNF57 | 19 | TCRsignalingPathway |
| CBL | 867 | Cas-Br-M (murine) ecotropic retroviral transforming sequence | C-CBL\|CBL2\|RNF55 | 11 | TCRsignalingPathway |
| CBLB | 868 | Cas-Br-M (murine) ecotropic retroviral transforming sequence b | DKFZp686J10223\|DKFZp779A0729\|DKFZp779F1443\|FLJ36865\|FLJ41152\|Nbla00127\|RNF56 | 3 | TCRsignalingPathway |
| IL2 | 3558 | interleukin 2 | IL-2\|TCGF\|lymphokine | 4 | TCRsignalingPathway |
| IL4 | 3565 | interleukin 4 | BCGF-1\|BCGF1\|BSF1\|IL-4\|MGC79402 | 5 | TCRsignalingPathway |
| IL5 | 3567 | interleukin 5 (colony-stimulating factor, eosinophil) | EDF\|IL-5\|TRF | 5 | TCRsignalingPathway |
| IL10 | 3586 | interleukin 10 | CSIF\|IL-10\|IL10A\|MGC126450\|MGC126451\|TGIF | 1 | TCRsignalingPathway |
| IFNG | 3458 | interferon, gamma | IFG\|IFI | 12 | TCRsignalingPathway |
| CSF2 | 1437 | colony stimulating factor 2 (granulocyte-macrophage) | GMCSF\|MGC131935\|MGC138897 | 5 | TCRsignalingPathway |
| TNF | 7124 | tumor necrosis factor (TNF superfamily, member 2) | DIF\|TNF-alpha\|TNFA\|TNFSF2 | 6 | TCRsignalingPathway |
| CDK4 | 1019 | cyclin-dependent kinase 4 | CMM3\|MGC14458\|PSK-J3 | 12 | TCRsignalingPathway |
| RASGRP1 | 10125 | RAS guanyl releasing protein 1 (calcium and DAG-regulated) | CALDAG-GEFI\|CALDAG-GEFII\|MGC129998\|MGC129999\|RASGRP\|V\|hRasGRP1 | 15 | TCRsignalingPathway |
| PDK1 | 5163 | pyruvate dehydrogenase kinase, isozyme 1 | - | 2 | TCRsignalingPathway |
| PLCG1 | 5335 | phospholipase C, gamma 1 | PLC-II\|PLC1\|PLC148\|PLCgamma1 | 20 | TCRsignalingPathway |
| PRKCQ | 5588 | protein kinase C, theta | MGC126514\|MGC141919\|PRKCT\|nPKC-theta | 10 | TCRsignalingPathway |
| TRAC | 28755 | T cell receptor alpha constant | - | 14 | TCRsignalingPathway |
| TRAJ1 | 28754 | T cell receptor alpha joining 1 (non-functional) | - | 14 | TCRsignalingPathway |
| TRAJ2 | 28753 | T cell receptor alpha joining 2 (non-functional) | - | 14 | TCRsignalingPathway |
| TRAJ3 | 28752 | T cell receptor alpha joining 3 | - | 14 | TCRsignalingPathway |
| TRAJ4 | 28751 | T cell receptor alpha joining 4 | - | 14 | TCRsignalingPathway |
| TRAJ5 | 28750 | T cell receptor alpha joining 5 | - | 14 | TCRsignalingPathway |
| TRAJ6 | 28749 | T cell receptor alpha joining 6 | - | 14 | TCRsignalingPathway |
| TRAJ7 | 28748 | T cell receptor alpha joining 7 | - | 14 | TCRsignalingPathway |
| TRAJ8 | 28747 | T cell receptor alpha joining 8 | - | 14 | TCRsignalingPathway |
| TRAJ9 | 28746 | T cell receptor alpha joining 9 | - | 14 | TCRsignalingPathway |
| TRAJ10 | 28745 | T cell receptor alpha joining 10 | - | 14 | TCRsignalingPathway |
| TRAJ11 | 28744 | T cell receptor alpha joining 11 | - | 14 | TCRsignalingPathway |
| TRAJ12 | 28743 | T cell receptor alpha joining 12 | - | 14 | TCRsignalingPathway |
| TRAJ13 | 28742 | T cell receptor alpha joining 13 | - | 14 | TCRsignalingPathway |
| TRAJ14 | 28741 | T cell receptor alpha joining 14 | - | 14 | TCRsignalingPathway |
| TRAJ15 | 28740 | T cell receptor alpha joining 15 | - | 14 | TCRsignalingPathway |
| TRAJ16 | 28739 | T cell receptor alpha joining 16 | - | 14 | TCRsignalingPathway |
| TRAJ17 | 28738 | T cell receptor alpha joining 17 | - | 14 | TCRsignalingPathway |
| TRAJ18 | 28737 | T cell receptor alpha joining 18 | - | 14 | TCRsignalingPathway |
| TRAJ19 | 28736 | T cell receptor alpha joining 19 (non-functional) | - | 14 | TCRsignalingPathway |
| TRAJ20 | 28735 | T cell receptor alpha joining 20 | - | 14 | TCRsignalingPathway |
| TRAJ21 | 28734 | T cell receptor alpha joining 21 | - | 14 | TCRsignalingPathway |
| TRAJ22 | 28733 | T cell receptor alpha joining 22 | - | 14 | TCRsignalingPathway |
| TRAJ23 | 28732 | T cell receptor alpha joining 23 | - | 14 | TCRsignalingPathway |
| TRAJ24 | 28731 | T cell receptor alpha joining 24 | - | 14 | TCRsignalingPathway |
| TRAJ25 | 28730 | T cell receptor alpha joining 25 (non-functional) | - | 14 | TCRsignalingPathway |
| TRAJ26 | 28729 | T cell receptor alpha joining 26 | - | 14 | TCRsignalingPathway |
| TRAJ27 | 28728 | T cell receptor alpha joining 27 | - | 14 | TCRsignalingPathway |
| TRAJ28 | 28727 | T cell receptor alpha joining 28 | - | 14 | TCRsignalingPathway |
| TRAJ29 | 28726 | T cell receptor alpha joining 29 | - | 14 | TCRsignalingPathway |
| TRAJ30 | 28725 | T cell receptor alpha joining 30 | - | 14 | TCRsignalingPathway |
| TRAJ31 | 28724 | T cell receptor alpha joining 31 | - | 14 | TCRsignalingPathway |
| TRAJ32 | 28723 | T cell receptor alpha joining 32 | - | 14 | TCRsignalingPathway |
| TRAJ33 | 28722 | T cell receptor alpha joining 33 | - | 14 | TCRsignalingPathway |
| TRAJ34 | 28721 | T cell receptor alpha joining 34 | - | 14 | TCRsignalingPathway |
| TRAJ35 | 28720 | T cell receptor alpha joining 35 (non-functional) | - | 14 | TCRsignalingPathway |
| TRAJ36 | 28719 | T cell receptor alpha joining 36 | - | 14 | TCRsignalingPathway |
| TRAJ37 | 28718 | T cell receptor alpha joining 37 | - | 14 | TCRsignalingPathway |
| TRAJ38 | 28717 | T cell receptor alpha joining 38 | - | 14 | TCRsignalingPathway |
| TRAJ39 | 28716 | T cell receptor alpha joining 39 | - | 14 | TCRsignalingPathway |
| TRAJ40 | 28715 | T cell receptor alpha joining 40 | - | 14 | TCRsignalingPathway |
| TRAJ41 | 28714 | T cell receptor alpha joining 41 | - | 14 | TCRsignalingPathway |
| TRAJ42 | 28713 | T cell receptor alpha joining 42 | - | 14 | TCRsignalingPathway |
| TRAJ43 | 28712 | T cell receptor alpha joining 43 | - | 14 | TCRsignalingPathway |
| TRAJ44 | 28711 | T cell receptor alpha joining 44 | - | 14 | TCRsignalingPathway |
| TRAJ45 | 28710 | T cell receptor alpha joining 45 | - | 14 | TCRsignalingPathway |
| TRAJ46 | 28709 | T cell receptor alpha joining 46 | - | 14 | TCRsignalingPathway |
| TRAJ47 | 28708 | T cell receptor alpha joining 47 | - | 14 | TCRsignalingPathway |
| TRAJ48 | 28707 | T cell receptor alpha joining 48 | - | 14 | TCRsignalingPathway |
| TRAJ49 | 28706 | T cell receptor alpha joining 49 | - | 14 | TCRsignalingPathway |
| TRAJ50 | 28705 | T cell receptor alpha joining 50 | - | 14 | TCRsignalingPathway |
| TRAJ52 | 28703 | T cell receptor alpha joining 52 | - | 14 | TCRsignalingPathway |
| TRAJ53 | 28702 | T cell receptor alpha joining 53 | - | 14 | TCRsignalingPathway |
| TRAJ54 | 28701 | T cell receptor alpha joining 54 | - | 14 | TCRsignalingPathway |
| TRAJ56 | 28699 | T cell receptor alpha joining 56 | - | 14 | TCRsignalingPathway |
| TRAJ57 | 28698 | T cell receptor alpha joining 57 | - | 14 | TCRsignalingPathway |
| TRAJ58 | 28697 | T cell receptor alpha joining 58 (non-functional) | - | 14 | TCRsignalingPathway |
| TRAJ59 | 28696 | T cell receptor alpha joining 59 (non-functional) | - | 14 | TCRsignalingPathway |
| TRAJ61 | 28694 | T cell receptor alpha joining 61 (non-functional) | - | 14 | TCRsignalingPathway |
| TRAV1-1 | 28693 | T cell receptor alpha variable 1-1 | TCRAV1S1\|TCRAV7S1\|TRAV11 | 14 | TCRsignalingPathway |
| TRAV1-2 | 28692 | T cell receptor alpha variable 1-2 | TCRAV1S2\|TCRAV7S2\|TRAV12 | 14 | TCRsignalingPathway |
| TRAV2 | 28691 | T cell receptor alpha variable 2 | TCRAV11S1\|TCRAV2S1 | 14 | TCRsignalingPathway |
| TRAV3 | 28690 | T cell receptor alpha variable 3 (gene/pseudogene) | TCRAV16S1\|TCRAV3S1 | 14 | TCRsignalingPathway |
| TRAV4 | 28689 | T cell receptor alpha variable 4 | TCRAV20S1\|TCRAV4S1 | 14 | TCRsignalingPathway |
| TRAV5 | 28688 | T cell receptor alpha variable 5 | TCRAV15S1\|TCRAV5S1 | 14 | TCRsignalingPathway |
| TRAV7 | 28686 | T cell receptor alpha variable 7 | TCRAV7S1 | 14 | TCRsignalingPathway |
| TRAV8-1 | 28685 | T cell receptor alpha variable 8-1 | TCRAV1S1\|TCRAV8S1\|TRAV81 | 14 | TCRsignalingPathway |
| TRAV8-2 | 28684 | T cell receptor alpha variable 8-2 | TCRAV1S5\|TCRAV8S2\|TRAV82 | 14 | TCRsignalingPathway |
| TRAV8-3 | 28683 | T cell receptor alpha variable 8-3 | TCRAV1S4\|TCRAV8S3\|TRAV83 | 14 | TCRsignalingPathway |
| TRAV8-4 | 28682 | T cell receptor alpha variable 8-4 | TCRAV1S2\|TCRAV8S4\|TRAV84 | 14 | TCRsignalingPathway |
| TRAV8-6 | 28680 | T cell receptor alpha variable 8-6 | TCRAV1S3\|TCRAV8S6\|TRAV86 | 14 | TCRsignalingPathway |
| TRAV8-7 | 28679 | T cell receptor alpha variable 8-7 (non-functional) | TCRAV8S7\|TRAV87 | 14 | TCRsignalingPathway |
| TRAV9-1 | 28678 | T cell receptor alpha variable 9-1 | TCRAV9S1\|TRAV91 | 14 | TCRsignalingPathway |
| TRAV9-2 | 28677 | T cell receptor alpha variable 9-2 | TCRAV22S1\|TCRAV9S2\|TRAV92 | 14 | TCRsignalingPathway |
| TRAV10 | 28676 | T cell receptor alpha variable 10 | TCRAV10S1\|TCRAV24S1 | 14 | TCRsignalingPathway |
| TRAV12-1 | 28674 | T cell receptor alpha variable 12-1 | TCRAV12S1\|TCRAV2S3\|TRAV121 | 14 | TCRsignalingPathway |
| TRAV12-2 | 28673 | T cell receptor alpha variable 12-2 | TCRAV12S2\|TCRAV2S1\|TRAV122 | 14 | TCRsignalingPathway |
| TRAV12-3 | 28672 | T cell receptor alpha variable 12-3 | TCRAV12S3\|TCRAV2S2\|TRAV123 | 14 | TCRsignalingPathway |
| TRAV13-1 | 28671 | T cell receptor alpha variable 13-1 | TCRAV13S1\|TCRAV8S1\|TRAV131 | 14 | TCRsignalingPathway |
| TRAV13-2 | 28670 | T cell receptor alpha variable 13-2 | TCRAV13S2\|TCRAV8S2\|TRAV132 | 14 | TCRsignalingPathway |
| TRAV14DV4 | 28669 | T cell receptor alpha variable 14/delta variable 4 | TCRAV6S1-hDV104S1\|TRAV14/DV4\|hADV14S1 | 14 | TCRsignalingPathway |
| TRAV16 | 28667 | T cell receptor alpha variable 16 | TCRAV16S1\|TCRAV9S1 | 14 | TCRsignalingPathway |
| TRAV17 | 28666 | T cell receptor alpha variable 17 | TCRAV17S1\|TCRAV3S1 | 14 | TCRsignalingPathway |
| TRAV18 | 28665 | T cell receptor alpha variable 18 | TCRAV18S1 | 14 | TCRsignalingPathway |
| TRAV19 | 28664 | T cell receptor alpha variable 19 | TCRAV12S1\|TCRAV19S1 | 14 | TCRsignalingPathway |
| TRAV20 | 28663 | T cell receptor alpha variable 20 | TCRAV20S1\|TCRAV30S1 | 14 | TCRsignalingPathway |
| TRAV21 | 28662 | T cell receptor alpha variable 21 | TCRAV21S1\|TCRAV23S1 | 14 | TCRsignalingPathway |
| TRAV22 | 28661 | T cell receptor alpha variable 22 | TCRAV13S1\|TCRAV22S1 | 14 | TCRsignalingPathway |
| TRAV23DV6 | 28660 | T cell receptor alpha variable 23/delta variable 6 | TCRAV17S1\|TRAV23/DV6\|hADV23S1 | 14 | TCRsignalingPathway |
| TRAV24 | 28659 | T cell receptor alpha variable 24 | TCRAV18S1\|TCRAV24S1 | 14 | TCRsignalingPathway |
| TRAV25 | 28658 | T cell receptor alpha variable 25 | TCRAV25S1\|TCRAV32S1 | 14 | TCRsignalingPathway |
| TRAV26-1 | 28657 | T cell receptor alpha variable 26-1 | TCRAV26S1\|TCRAV4S2\|TRAV261 | 14 | TCRsignalingPathway |
| TRAV26-2 | 28656 | T cell receptor alpha variable 26-2 | TCRAV26S2\|TCRAV4S1\|TRAV262 | 14 | TCRsignalingPathway |
| TRAV27 | 28655 | T cell receptor alpha variable 27 | TCRAV10S1\|TCRAV27S1 | 14 | TCRsignalingPathway |
| TRAV29DV5 | 28653 | T cell receptor alpha variable 29/delta variable 5 (gene/pseudogene) | TCRAV21S1\|TRAV29/DV5\|hADV29S1 | 14 | TCRsignalingPathway |
| TRAV30 | 28652 | T cell receptor alpha variable 30 | TCRAV29S1\|TCRAV30S1 | 14 | TCRsignalingPathway |
| TRAV34 | 28648 | T cell receptor alpha variable 34 | TCRAV26S1\|TCRAV34S1 | 14 | TCRsignalingPathway |
| TRAV35 | 28647 | T cell receptor alpha variable 35 | TCRAV25S1\|TCRAV35S1 | 14 | TCRsignalingPathway |
| TRAV36DV7 | 28646 | T cell receptor alpha variable 36/delta variable 7 | TCRAV28S1\|TRAV36/DV7\|hADV36S1 | 14 | TCRsignalingPathway |
| TRAV38-1 | 28644 | T cell receptor alpha variable 38-1 | TCRAV14S2\|TCRAV38S1\|TRAV381 | 14 | TCRsignalingPathway |
| TRAV38-2DV8 | 28643 | T cell receptor alpha variable 38-2/delta variable 8 | TCRAV14S1\|TRAV382DV8\|hADV38S2 | 14 | TCRsignalingPathway |
| TRAV39 | 28642 | T cell receptor alpha variable 39 | TCRAV27S1\|TCRAV39S1 | 14 | TCRsignalingPathway |
| TRAV40 | 28641 | T cell receptor alpha variable 40 | TCRAV31S1\|TCRAV40S1 | 14 | TCRsignalingPathway |
| TRAV41 | 28640 | T cell receptor alpha variable 41 | TCRAV19S1\|TCRAV41S1 | 14 | TCRsignalingPathway |
| TRBC1 | 28639 | T cell receptor beta constant 1 | BV05S1J2.2\|MGC88817\|TCRBC1 | 7 | TCRsignalingPathway |
| TRBC2 | 28638 | T cell receptor beta constant 2 | TCRBC2 | 7 | TCRsignalingPathway |
| TRBD1 | 28637 | T cell receptor beta diversity 1 | TCRBD1 | 7 | TCRsignalingPathway |
| TRBD2 | 28636 | T cell receptor beta diversity 2 | TCRBD2 | 7 | TCRsignalingPathway |
| TRBJ1-1 | 28635 | T cell receptor beta joining 1-1 | TCRBJ1S1\|TRBJ11 | 7 | TCRsignalingPathway |
| TRBJ1-2 | 28634 | T cell receptor beta joining 1-2 | TCRBJ1S2\|TRBJ12 | 7 | TCRsignalingPathway |
| TRBJ1-3 | 28633 | T cell receptor beta joining 1-3 | TCRBJ1S3\|TRBJ13 | 7 | TCRsignalingPathway |
| TRBJ1-4 | 28632 | T cell receptor beta joining 1-4 | TCRBJ1S4\|TRBJ14 | 7 | TCRsignalingPathway |
| TRBJ1-5 | 28631 | T cell receptor beta joining 1-5 | TCRBJ1S5\|TRBJ15 | 7 | TCRsignalingPathway |
| TRBJ1-6 | 28630 | T cell receptor beta joining 1-6 | TCRBJ1S6\|TRBJ16 | 7 | TCRsignalingPathway |
| TRBJ2-1 | 28629 | T cell receptor beta joining 2-1 | TCRBJ2S1\|TRBJ21 | 7 | TCRsignalingPathway |
| TRBJ2-2 | 28628 | T cell receptor beta joining 2-2 | TCRBJ2S2\|TRBJ22 | 7 | TCRsignalingPathway |
| TRBJ2-3 | 28626 | T cell receptor beta joining 2-3 | TCRBJ2S3\|TRBJ23 | 7 | TCRsignalingPathway |
| TRBJ2-4 | 28625 | T cell receptor beta joining 2-4 | TCRBJ2S4\|TRBJ24 | 7 | TCRsignalingPathway |
| TRBJ2-5 | 28624 | T cell receptor beta joining 2-5 | TCRBJ2S5\|TRBJ25 | 7 | TCRsignalingPathway |
| TRBJ2-6 | 28623 | T cell receptor beta joining 2-6 | TCRBJ2S6\|TRBJ26 | 7 | TCRsignalingPathway |
| TRBJ2-7 | 28622 | T cell receptor beta joining 2-7 | TCRBJ2S7\|TRBJ27 | 7 | TCRsignalingPathway |
| TRBV2 | 28620 | T cell receptor beta variable 2 | TCRBV22S1A2N1T\|TCRBV2S1 | 7 | TCRsignalingPathway |
| TRBV3-1 | 28619 | T cell receptor beta variable 3-1 | TCRBV3S1\|TCRBV9S1A1T\|TRBV31 | 7 | TCRsignalingPathway |
| TRBV4-1 | 28617 | T cell receptor beta variable 4-1 | BV07S1J2.7\|TCRBV4S1\|TCRBV7S1A1N2T\|TRBV41 | 7 | TCRsignalingPathway |
| TRBV4-2 | 28616 | T cell receptor beta variable 4-2 | TCRBV4S2\|TCRBV7S3A2T\|TRBV42 | 7 | TCRsignalingPathway |
| TRBV4-3 | 28615 | T cell receptor beta variable 4-3 | TCRBV4S3\|TCRBV7S2A1N4T\|TRBV43 | 7 | TCRsignalingPathway |
| TRBV5-1 | 28614 | T cell receptor beta variable 5-1 | TCRBV5S1\|TCRBV5S1A1T\|TRBV51 | 7 | TCRsignalingPathway |
| TRBV5-4 | 28611 | T cell receptor beta variable 5-4 | TCRBV5S4\|TCRBV5S6A3N2T\|TRBV54 | 7 | TCRsignalingPathway |
| TRBV5-5 | 28610 | T cell receptor beta variable 5-5 | TCRBV5S3A2T\|TCRBV5S5\|TRBV55 | 7 | TCRsignalingPathway |
| TRBV5-6 | 28609 | T cell receptor beta variable 5-6 | TCRBV5S2\|TCRBV5S6\|TRBV56 | 7 | TCRsignalingPathway |
| TRBV5-7 | 28608 | T cell receptor beta variable 5-7 (non-functional) | TCRBV5S7\|TCRBV5S7P\|TRBV57 | 7 | TCRsignalingPathway |
| TRBV5-8 | 28607 | T cell receptor beta variable 5-8 | TCRBV5S4A2T\|TCRBV5S8\|TRBV58 | 7 | TCRsignalingPathway |
| TRBV6-1 | 28606 | T cell receptor beta variable 6-1 | TCRBV13S3\|TCRBV6S1\|TRBV61 | 7 | TCRsignalingPathway |
| TRBV6-2 | 28605 | T cell receptor beta variable 6-2 (gene/pseudogene) | TCRBV13S2A1T\|TCRBV6S2\|TRBV62 | 7 | TCRsignalingPathway |
| TRBV6-3 | 28604 | T cell receptor beta variable 6-3 | TCRBV13S9/13S2A1T\|TCRBV6S3\|TRBV63 | 7 | TCRsignalingPathway |
| TRBV6-4 | 28603 | T cell receptor beta variable 6-4 | TCRBV13S5\|TCRBV6S4\|TRBV64 | 7 | TCRsignalingPathway |
| TRBV6-5 | 28602 | T cell receptor beta variable 6-5 | TCRBV13S1\|TCRBV6S5\|TRBV65 | 7 | TCRsignalingPathway |
| TRBV6-6 | 28601 | T cell receptor beta variable 6-6 | TCRBV13S6A2T\|TCRBV6S6\|TRBV66 | 7 | TCRsignalingPathway |
| TRBV6-7 | 28600 | T cell receptor beta variable 6-7 (non-functional) | TCRBV13S8P\|TCRBV6S7\|TRBV67 | 7 | TCRsignalingPathway |
| TRBV6-8 | 28599 | T cell receptor beta variable 6-8 | TCRBV13S7P\|TCRBV6S8\|TRBV68 | 7 | TCRsignalingPathway |
| TRBV6-9 | 28598 | T cell receptor beta variable 6-9 | TCRBV13S4\|TCRBV6S9\|TRBV69 | 7 | TCRsignalingPathway |
| TRBV7-2 | 28596 | T cell receptor beta variable 7-2 | MGC117435\|TCRBV6S5A1N1\|TCRBV7S2\|TRBV72 | 7 | TCRsignalingPathway |
| TRBV7-3 | 28595 | T cell receptor beta variable 7-3 | TCRBV6S1A1N1\|TCRBV7S3\|TRBV73 | 7 | TCRsignalingPathway |
| TRBV7-4 | 28594 | T cell receptor beta variable 7-4 (gene/pseudogene) | TCRBV6S8A2T\|TCRBV7S4\|TRBV74 | 7 | TCRsignalingPathway |
| TRBV7-6 | 28592 | T cell receptor beta variable 7-6 | TCRBV6S3A1N1T\|TCRBV7S6\|TRBV76 | 7 | TCRsignalingPathway |
| TRBV7-7 | 28591 | T cell receptor beta variable 7-7 | TCRBV6S6A2T\|TCRBV7S7\|TRBV77 | 7 | TCRsignalingPathway |
| TRBV7-8 | 28590 | T cell receptor beta variable 7-8 | TCRBV6S2A1N1T\|TCRBV7S8\|TRBV78 | 7 | TCRsignalingPathway |
| TRBV7-9 | 28589 | T cell receptor beta variable 7-9 | TCRBV6S4A1\|TCRBV7S9\|TRBV79 | 7 | TCRsignalingPathway |
| TRBV9 | 28586 | T cell receptor beta variable 9 | TCRBV1S1A1N1\|TCRBV9S1 | 7 | TCRsignalingPathway |
| TRBV10-1 | 28585 | T cell receptor beta variable 10-1(gene/pseudogene) | TCRBV10S1\|TCRBV12S2\|TCRBV12S2A1T\|TRBV101 | 7 | TCRsignalingPathway |
| TRBV10-2 | 28584 | T cell receptor beta variable 10-2 | TCRBV10S2\|TCRBV12S3\|TRBV102 | 7 | TCRsignalingPathway |
| TRBV10-3 | 28583 | T cell receptor beta variable 10-3 | TCRBV10S3\|TCRBV12S1A1N2\|TRBV103 | 7 | TCRsignalingPathway |
| TRBV11-1 | 28582 | T cell receptor beta variable 11-1 | TCRBV11S1\|TCRBV21S1\|TRBV111 | 7 | TCRsignalingPathway |
| TRBV11-2 | 28581 | T cell receptor beta variable 11-2 | TCRBV11S2\|TCRBV21S3A2N2T\|TRBV112 | 7 | TCRsignalingPathway |
| TRBV11-3 | 28580 | T cell receptor beta variable 11-3 | TCRBV11S3\|TCRBV21S2A2\|TRBV113 | 7 | TCRsignalingPathway |
| TRBV12-3 | 28577 | T cell receptor beta variable 12-3 | TCRBV12S3\|TCRBV8S1\|TRBV123 | 7 | TCRsignalingPathway |
| TRBV12-4 | 28576 | T cell receptor beta variable 12-4 | TCRBV12S4\|TCRBV8S2A1T\|TRBV124 | 7 | TCRsignalingPathway |
| TRBV12-5 | 28575 | T cell receptor beta variable 12-5 | TCRBV12S5\|TCRBV8S3\|TRBV125 | 7 | TCRsignalingPathway |
| TRBV13 | 28574 | T cell receptor beta variable 13 | TCRBV13S1\|TCRBV23S1A2T | 7 | TCRsignalingPathway |
| TRBV14 | 28573 | T cell receptor beta variable 14 | TCRBV14S1\|TCRBV16S1A1N1 | 7 | TCRsignalingPathway |
| TRBV15 | 28572 | T cell receptor beta variable 15 | TCRBV15S1\|TCRBV24S1A3T | 7 | TCRsignalingPathway |
| TRBV16 | 28571 | T cell receptor beta variable 16 (gene/pseudogene) | TCRBV16S1\|TCRBV25S1A2PT | 7 | TCRsignalingPathway |
| TRBV17 | 28570 | T cell receptor beta variable 17 (non-functional) | TCRBV17S1\|TCRBV26S1P | 7 | TCRsignalingPathway |
| TRBV18 | 28569 | T cell receptor beta variable 18 | TCRBV18S1 | 7 | TCRsignalingPathway |
| TRBV19 | 28568 | T cell receptor beta variable 19 | TCRBV17S1A1T\|TCRBV19S1 | 7 | TCRsignalingPathway |
| TRBV20-1 | 28567 | T cell receptor beta variable 20-1 | TCRBV20S1\|TCRBV2S1\|TRBV201 | 7 | TCRsignalingPathway |
| TRBV24-1 | 28563 | T cell receptor beta variable 24-1 | TCRBV15S1\|TCRBV24S1\|TRBV241 | 7 | TCRsignalingPathway |
| TRBV25-1 | 28562 | T cell receptor beta variable 25-1 | TCRBV11S1A1T\|TCRBV25S1\|TRBV251 | 7 | TCRsignalingPathway |
| TRBV27 | 28560 | T cell receptor beta variable 27 | FLJ35984\|TCRBV14S1\|TCRBV27S1 | 7 | TCRsignalingPathway |
| TRBV28 | 28559 | T cell receptor beta variable 28 | TCRBV28S1\|TCRBV3S1 | 7 | TCRsignalingPathway |
| TRBV29-1 | 28558 | T cell receptor beta variable 29-1 | TCRBV29S1\|TCRBV4S1A1T\|TRBV291 | 7 | TCRsignalingPathway |
| TRBV30 | 28557 | T cell receptor beta variable 30 (gene/pseudogene) | TCRBV20S1A1N2\|TCRBV30S1 | 7 | TCRsignalingPathway |
| TRDC | 28526 | T cell receptor delta constant | - | 14 | TCRsignalingPathway |
| TRDD1 | 28525 | T cell receptor delta diversity 1 | - | 14 | TCRsignalingPathway |
| TRDD2 | 28524 | T cell receptor delta diversity 2 | - | 14 | TCRsignalingPathway |
| TRDD3 | 28523 | T cell receptor delta diversity 3 | TCRD | 14 | TCRsignalingPathway |
| TRDJ1 | 28522 | T cell receptor delta joining 1 | TCRD | 14 | TCRsignalingPathway |
| TRDJ2 | 28521 | T cell receptor delta joining 2 | - | 14 | TCRsignalingPathway |
| TRDJ3 | 28520 | T cell receptor delta joining 3 | - | 14 | TCRsignalingPathway |
| TRDJ4 | 28519 | T cell receptor delta joining 4 | - | 14 | TCRsignalingPathway |
| TRDV1 | 28518 | T cell receptor delta variable 1 | hDV101S1 | 14 | TCRsignalingPathway |
| TRDV2 | 28517 | T cell receptor delta variable 2 | MGC117421\|hDV102S1 | 14 | TCRsignalingPathway |
| TRDV3 | 28516 | T cell receptor delta variable 3 | hDV103S1 | 14 | TCRsignalingPathway |
| TRGV9 | 6983 | T cell receptor gamma variable 9 | MGC47828\|TCRGV9\|V2 | 7 | TCRsignalingPathway |
| TRGV8 | 6982 | T cell receptor gamma variable 8 | TCRGV8\|V1S8 | 7 | TCRsignalingPathway |
| TRGV5 | 6978 | T cell receptor gamma variable 5 | TCRGV5\|V1S5 | 7 | TCRsignalingPathway |
| TRGV4 | 6977 | T cell receptor gamma variable 4 | TCRGV4\|V1S4 | 7 | TCRsignalingPathway |
| TRGV3 | 6976 | T cell receptor gamma variable 3 | TCRGV3\|V1S3 | 7 | TCRsignalingPathway |
| TRGV2 | 6974 | T cell receptor gamma variable 2 | MGC42817\|TCRGV2\|VIS2 | 7 | TCRsignalingPathway |
| TRGJP2 | 6972 | T cell receptor gamma joining P2 | JP2\|TCRGJP2 | 7 | TCRsignalingPathway |
| TRGJP1 | 6971 | T cell receptor gamma joining P1 | JP1\|TCRGJP1 | 7 | TCRsignalingPathway |
| TRGJP | 6970 | T cell receptor gamma joining P | JP\|TCRGJP | 7 | TCRsignalingPathway |
| TRGJ2 | 6969 | T cell receptor gamma joining 2 | J2\|TCRGJ2 | 7 | TCRsignalingPathway |
| TRGJ1 | 6968 | T cell receptor gamma joining 1 | J1\|TCRGJ1 | 7 | TCRsignalingPathway |
| TRGC2 | 6967 | T cell receptor gamma constant 2 | TCRGC2\|TRGC2(2X)\|TRGC2(3X) | 7 | TCRsignalingPathway |
| TRGC1 | 6966 | T cell receptor gamma constant 1 | C1\|TCRGC1 | 7 | TCRsignalingPathway |
| TRAV6 | 6956 | T cell receptor alpha variable 6 | TCRAV5S1\|TCRAV6S1 | 14 | TCRsignalingPathway |
| BMP1 | 649 | bone morphogenetic protein 1 | FLJ44432\|PCOLC\|PCP\|TLD\|pCP-2 | 8 | TGFb Family Member |
| BMP10 | 27302 | bone morphogenetic protein 10 | MGC126783 | 2 | TGFb Family Member |
| BMP15 | 9210 | bone morphogenetic protein 15 | GDF9B\|ODG2\|POF4 | X | TGFb Family Member |
| BMP2 | 650 | bone morphogenetic protein 2 | BMP2A | 20 | TGFb Family Member |
| BMP3 | 651 | bone morphogenetic protein 3 | BMP-3A | 4 | TGFb Family Member |
| BMP4 | 652 | bone morphogenetic protein 4 | BMP2B\|BMP2B1\|MCOPS6\|OFC11\|ZYME | 14 | TGFb Family Member |
| BMP5 | 653 | bone morphogenetic protein 5 | MGC34244 | 6 | TGFb Family Member |
| BMP6 | 654 | bone morphogenetic protein 6 | VGR\|VGR1 | 6 | TGFb Family Member |
| BMP7 | 655 | bone morphogenetic protein 7 | OP-1 | 20 | TGFb Family Member |
| BMP8A | 353500 | bone morphogenetic protein 8a | FLJ14351\|FLJ45264 | 1 | TGFb Family Member |
| BMP8B | 656 | bone morphogenetic protein 8b | BMP8\|MGC131757\|OP2 | 1 | TGFb Family Member |
| GDF1 | 2657 | growth differentiation factor 1 | - | 19 | TGFb Family Member |
| GDF10 | 2662 | growth differentiation factor 10 | BMP-3b\|BMP3B | 10 | TGFb Family Member |
| GDF11 | 10220 | growth differentiation factor 11 | BMP-11\|BMP11 | 12 | TGFb Family Member |
| GDF15 | 9518 | growth differentiation factor 15 | GDF-15\|MIC-1\|MIC1\|NAG-1\|PDF\|PLAB\|PTGFB | 19 | TGFb Family Member |
| GDF2 | 2658 | growth differentiation factor 2 | BMP-9\|BMP9 | 10 | TGFb Family Member |
| GDF3 | 9573 | growth differentiation factor 3 | - | 12 | TGFb Family Member |
| GDF5 | 8200 | growth differentiation factor 5 | BMP14\|CDMP1\|LAP4\|OS5\|SYNS2 | 20 | TGFb Family Member |
| GDF6 | 392255 | growth differentiation factor 6 | BMP13\|CDMP2\|KFS\|KFSL\|MGC158100\|MGC158101\|SGM1 | 8 | TGFb Family Member |
| GDF7 | 151449 | growth differentiation factor 7 | BMP12 | 2 | TGFb Family Member |
| GDF9 | 2661 | growth differentiation factor 9 | - | 5 | TGFb Family Member |
| GDNF | 2668 | glial cell derived neurotrophic factor | ATF1\|ATF2\|HFB1-GDNF | 5 | TGFb Family Member |
| INHA | 3623 | inhibin, alpha | - | 2 | TGFb Family Member |
| INHBA | 3624 | inhibin, beta A | EDF\|FRP | 7 | TGFb Family Member |
| INHBB | 3625 | inhibin, beta B | MGC157939 | 2 | TGFb Family Member |
| INHBC | 3626 | inhibin, beta C | IHBC | 12 | TGFb Family Member |
| INHBE | 83729 | inhibin, beta E | MGC4638 | 12 | TGFb Family Member |
| LEFTY1 | 10637 | left-right determination factor 1 | LEFTB\|LEFTYB | 1 | TGFb Family Member |
| LEFTY2 | 7044 | left-right determination factor 2 | EBAF\|LEFTA\|LEFTYA\|MGC46222\|TGFB4 | 1 | TGFb Family Member |
| NODAL | 4838 | nodal homolog (mouse) | MGC138230 | 10 | TGFb Family Member |
| TGFB1 | 7040 | transforming growth factor, beta 1 | CED\|DPD1\|TGFB\|TGFbeta | 19 | TGFb Family Member |
| TGFB2 | 7042 | transforming growth factor, beta 2 | MGC116892\|TGF-beta2 | 1 | TGFb Family Member |
| TGFB3 | 7043 | transforming growth factor, beta 3 | ARVD\|FLJ16571\|TGF-beta3 | 14 | TGFb Family Member |
| ACVR1B | 91 | activin A receptor, type IB | ACTRIB\|ACVRLK4\|ALK4\|SKR2 | 12 | TGFb Family Member Receptor |
| ACVR1C | 130399 | activin A receptor, type IC | ACVRLK7\|ALK7 | 2 | TGFb Family Member Receptor |
| ACVR2A | 92 | activin A receptor, type IIA | ACTRII\|ACVR2 | 2 | TGFb Family Member Receptor |
| ACVR2B | 93 | activin A receptor, type IIB | ACTRIIB\|ActR-IIB\|MGC116908 | 3 | TGFb Family Member Receptor |
| ACVRL1 | 94 | activin A receptor type II-like 1 | ACVRLK1\|ALK-1\|ALK1\|HHT\|HHT2\|ORW2\|SKR3\|TSR-I | 12 | TGFb Family Member Receptor |
| AMHR2 | 269 | anti-Mullerian hormone receptor, type II | AMHR\|MISR2\|MISRII | 12 | TGFb Family Member Receptor |
| BMPR1A | 657 | bone morphogenetic protein receptor, type IA | 10q23del\|ACVRLK3\|ALK3\|CD292\|SKR5 | 10 | TGFb Family Member Receptor |
| BMPR1B | 658 | bone morphogenetic protein receptor, type IB | ALK-6\|ALK6\|CDw293 | 4 | TGFb Family Member Receptor |
| BMPR2 | 659 | bone morphogenetic protein receptor, type II (serine/threonine kinase) | BMPR-II\|BMPR3\|BMR2\|BRK-3\|FLJ41585\|FLJ76945\|PPH1\|T-ALK | 2 | TGFb Family Member Receptor |
| TGFBR1 | 7046 | transforming growth factor, beta receptor 1 | AAT5\|ACVRLK4\|ALK-5\|ALK5\|LDS1A\|LDS2A\|SKR4\|TGFR-1 | 9 | TGFb Family Member Receptor |
| TGFBR2 | 7048 | transforming growth factor, beta receptor II (70/80kDa) | AAT3\|FAA3\|LDS1B\|LDS2B\|MFS2\|RIIC\|TAAD2\|TGFR-2\|TGFbeta-RII | 3 | TGFb Family Member Receptor |
| TGFBR3 | 7049 | transforming growth factor, beta receptor III | BGCAN\|betaglycan | 1 | TGFb Family Member Receptor |
| TNFRSF11B | 4982 | tumor necrosis factor receptor superfamily, member 11b | MGC29565\|OCIF\|OPG\|TR1 | 8 | TNF Family Members |
| TNFSF10 | 8743 | tumor necrosis factor (ligand) superfamily, member 10 | APO2L\|Apo-2L\|CD253\|TL2\|TRAIL | 3 | TNF Family Members |
| TNFSF11 | 8600 | tumor necrosis factor (ligand) superfamily, member 11 | CD254\|ODF\|OPGL\|OPTB2\|RANKL\|TRANCE\|hRANKL2\|sOdf | 13 | TNF Family Members |
| TNFSF12 | 8742 | tumor necrosis factor (ligand) superfamily, member 12 | APO3L\|DR3LG\|MGC129581\|MGC20669\|TWEAK | 17 | TNF Family Members |
| TNFSF13 | 8741 | tumor necrosis factor (ligand) superfamily, member 13 | APRIL\|CD256\|TALL2\|TRDL-1\|UNQ383/PRO715\|ligand | 17 | TNF Family Members |
| TNFSF13B | 10673 | tumor necrosis factor (ligand) superfamily, member 13b | BAFF\|BLYS\|CD257\|DTL\|TALL-1\|TALL1\|THANK\|TNFSF20\|ZTNF4 | 13 | TNF Family Members |
| TNFSF14 | 8740 | tumor necrosis factor (ligand) superfamily, member 14 | CD258\|HVEML\|LIGHT\|LTg\|TR2 | 19 | TNF Family Members |
| TNFSF15 | 9966 | tumor necrosis factor (ligand) superfamily, member 15 | MGC129934\|MGC129935\|TL1\|TL1A\|VEGI\|VEGI192A | 9 | TNF Family Members |
| TNFSF18 | 8995 | tumor necrosis factor (ligand) superfamily, member 18 | AITRL\|GITRL\|MGC138237\|TL6\|hGITRL | 1 | TNF Family Members |
| TNFSF4 | 7292 | tumor necrosis factor (ligand) superfamily, member 4 | CD134L\|CD252\|GP34\|OX-40L\|OX4OL\|TXGP1 | 1 | TNF Family Members |
| TNFSF8 | 944 | tumor necrosis factor (ligand) superfamily, member 8 | CD153\|CD30L\|CD30LG\|MGC138144 | 9 | TNF Family Members |
| TNFSF9 | 8744 | tumor necrosis factor (ligand) superfamily, member 9 | 4-1BB-L\|CD137L | 19 | TNF Family Members |
| TNFRSF10B | 8795 | tumor necrosis factor receptor superfamily, member 10b | CD262\|DR5\|KILLER\|KILLER/DR5\|TRAIL-R2\|TRAILR2\|TRICK2\|TRICK2A\|TRICK2B\|TRICKB\|ZTNFR9 | 8 | TNF Family Members Receptors |
| TNFRSF10C | 8794 | tumor necrosis factor receptor superfamily, member 10c, decoy without an intracellular domain | CD263\|DCR1\|LIT\|MGC149501\|MGC149502\|TRAILR3\|TRID | 8 | TNF Family Members Receptors |
| TNFRSF10D | 8793 | tumor necrosis factor receptor superfamily, member 10d, decoy with truncated death domain | CD264\|DCR2\|TRAILR4\|TRUNDD | 8 | TNF Family Members Receptors |
| TNFRSF11A | 8792 | tumor necrosis factor receptor superfamily, member 11a, NFKB activator | CD265\|FEO\|LOH18CR1\|ODFR\|OFE\|OPTB7\|OSTS\|PDB2\|RANK\|TRANCER | 18 | TNF Family Members Receptors |
| TNFRSF12A | 51330 | tumor necrosis factor receptor superfamily, member 12A | CD266\|FN14\|TWEAKR | 16 | TNF Family Members Receptors |
| TNFRSF13B | 23495 | tumor necrosis factor receptor superfamily, member 13B | CD267\|CVID\|FLJ39942\|MGC133214\|MGC39952\|TACI\|TNFRSF14B | 17 | TNF Family Members Receptors |
| TNFRSF13C | 115650 | tumor necrosis factor receptor superfamily, member 13C | BAFF-R\|BAFFR\|CD268\|MGC138235 | 22 | TNF Family Members Receptors |
| TNFRSF14 | 8764 | tumor necrosis factor receptor superfamily, member 14 (herpesvirus entry mediator) | ATAR\|HVEA\|HVEM\|LIGHTR\|TR2 | 1 | TNF Family Members Receptors |
| TNFRSF17 | 608 | tumor necrosis factor receptor superfamily, member 17 | BCM\|BCMA\|CD269 | 16 | TNF Family Members Receptors |
| TNFRSF18 | 8784 | tumor necrosis factor receptor superfamily, member 18 | AITR\|GITR\|GITR-D | 1 | TNF Family Members Receptors |
| TNFRSF19 | 55504 | tumor necrosis factor receptor superfamily, member 19 | TAJ\|TAJ-alpha\|TRADE\|TROY | 13 | TNF Family Members Receptors |
| TNFRSF1A | 7132 | tumor necrosis factor receptor superfamily, member 1A | CD120a\|FPF\|MGC19588\|TBP1\|TNF-R\|TNF-R-I\|TNF-R55\|TNFAR\|TNFR1\|TNFR55\|TNFR60\|p55\|p55-R\|p60 | 12 | TNF Family Members Receptors |
| TNFRSF1B | 7133 | tumor necrosis factor receptor superfamily, member 1B | CD120b\|TBPII\|TNF-R-II\|TNF-R75\|TNFBR\|TNFR1B\|TNFR2\|TNFR80\|p75\|p75TNFR | 1 | TNF Family Members Receptors |
| TNFRSF21 | 27242 | tumor necrosis factor receptor superfamily, member 21 | BM-018\|DR6\|MGC31965 | 6 | TNF Family Members Receptors |
| TNFRSF25 | 8718 | tumor necrosis factor receptor superfamily, member 25 | APO-3\|DDR3\|DR3\|LARD\|TNFRSF12\|TR3\|TRAMP\|WSL-1\|WSL-LR | 1 | TNF Family Members Receptors |
| TNFRSF4 | 7293 | tumor necrosis factor receptor superfamily, member 4 | ACT35\|CD134\|OX40\|TXGP1L | 1 | TNF Family Members Receptors |
| TNFRSF6B | 8771 | tumor necrosis factor receptor superfamily, member 6b, decoy | DCR3\|DJ583P15.1.1\|M68\|TR6 | 20 | TNF Family Members Receptors |
| TNFRSF8 | 943 | tumor necrosis factor receptor superfamily, member 8 | CD30\|D1S166E\|Ki-1 | 1 | TNF Family Members Receptors |
| TNFRSF9 | 3604 | tumor necrosis factor receptor superfamily, member 9 | 4-1BB\|CD137\|CDw137\|ILA\|MGC2172 | 1 | TNF Family Members Receptors |
